# Toward Larger Cyclo‐9,10‐Anthryleneparaphenylenes

**DOI:** 10.1002/chem.70878

**Published:** 2026-03-27

**Authors:** Moritz P. Schuldt, Frank Rominger, Sven M. Elbert, Michael Mastalerz

**Affiliations:** ^1^ Organisch‐Chemisches Institut Ruprecht‐Karls‐Universität Heidelberg Heidelberg Germany

**Keywords:** anthracene, endoperoxides, macrocycles, nanohoops, oligomers

## Abstract

Nanohoops of different sizes can be generated from various aromatic building blocks. By a cross‐coupling strategy of an anthracene‐based kinked precursor and 9,10‐diborylated anthracene, nanohoops based on diphenylanthracene units were synthesized and isolated up to the nonamer with 36 para‐connected aromatic rings. Their three‐dimensional structures were investigated by X‐ray diffraction, and their reactivity against oxygen was studied. The trimeric macrocycle was rearomatized to the corresponding cyclo‐9,10‐anthryleneparaphenylene (CAPP) in solution, which turned out to be prone to oxidation.

## Graphical abstract

During a macrocyclization with a dihydroanthracene diol as corner unit, larger oligomers than the trimer and tetramer were formed and isolated up to the nonamer. Their three‐dimensional structures were studied by X‐ray diffraction. The most abundant macrocycles were rearomatized to the corresponding CPP derivatives.

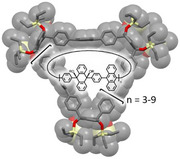

## 1 Introduction

**FIGURE 1:**
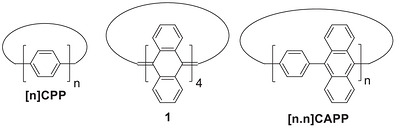
Structures of **[n]CPPs**, picotube **1**, and **[n.n]CAPPs**.

Since the initial synthesis of cycloparaphenylenes ([n]CPPs, see Figure 1), new methods for their synthesis as well as a plethora of new derivatives with varying properties have been reported. These include the parent [n]CPPs with *n* = 5‐16, 18, 20, and 21 [1, 2, 3, 4, 5, 6, 7, 8, 9, 10, 11, 12] as well as derivatives with various functional groups and nanohoops with various aromatic units [13, 14, 15, 16].

While CPPs consisting only of phenylene units are the simplest type of nanohoops, multiple π‐extended derivatives have been reported in recent years [13, 14]. Among these are nanohhoops based on naphthalenes with 1,4‐ [17, 18, 19, 20, 21, 22, 23] and 2,6‐connectivity [24, 25, 26], or larger anthracenes with 1,4‐ [27], 2,6‐ [28, 29] or 9,10‐connectivity [30, 31, 32, 33, 34]. For anthracene‐based nanohoops, the lower stabilization of the anthracene's central ring has two effects. On one hand, this ring is prone to oxidation to, for example, endoperoxides [35, 36, 37], or to cycloadditions [38, 39, 40], and to the reduction to dihydroanthracenes [41]. Therefore only few 9,10‐anthrylene containing nanohoops are known [30, 31, 32, 33, 34]. On the other hand, its reduced aromatic stabilization has allowed the synthesis of even smaller nanohoops than the smallest CPP ([5]CPP), such as the picotube **1** (see Figure 1), in which all phenylenes of [4]CPP are replaced by 9,10‐anthrylenes, which adopt a quinoidal structure and are bent [34]. Recently, this concept of incorporating anthrylenes in small CPP derivatives to reduce strain was used to obtain a nanohoop closer to the elusive [4]CPP, the [2.2]cyclo‐9,10‐anthryleneparaphenylene (**[2.2]CAPP**, see Figure 1), which contains two anthracene and two phenylene units [30]. For its synthesis, a dihydroanthracene diol was used as building block and successfully rearomatized by a new approach based on bromination, followed by reduction with metallic zinc. The high strain of 88 kcal/mol and low stability of the resulting **[2.2]CAPP** made purification and follow‐up chemistry impossible. Nevertheless, a single crystal X‐ray structure of **[2.2]CAPP** was obtained, confirming its quinoidal structure [30]. Although the pattern of alternating phenylene and 9,10‐anthrylene units in **[2.2]CAPP** makes it a potential precursor toward pentagon embedded nanobelts, its small size and high strain energy make such a synthesis unfeasible.

At the other end of the size spectrum, the number of corner units in a nanohoop precursor is a limiting factor as most synthetic approaches give three, four, five, and, rarely, six corner units [4, 22, 32, 42, 43, 44, 45, 46, 47, 48, 49]. Therefore, the largest CPP derivatives have been synthesized by three main strategies. Either by extending the building blocks such as phenylenes to biphenyles or even terphenylenes [2], by cross‐coupling them with linear linkers [9] or, perhaps more elegantly, by widening the internal angle of the bent building block [32]. For the last approach nanohoop precursors with up to seven corner units were reported, using a 9,10‐epoxyanthracene with an internal angle of 126° as the building block [32]. These precursors were successfully aromatized to derivatives of the largest CPP reported so far ([21]CPP) [2, 32]. Unlike their smaller congeners, large nanohoops have lower strain energies [50, 51], therefore we envisioned an approach toward pentagon embedded nanobelts based on larger **[n.n]CAPPs**, for example, **[6.6]CAPP** (with a strain energy of 59 kcal/mol calculated at B3LYP/6‐311G(d) level of theory). The resulting nanobelts are not only conjugated, non‐alternant nanobelts [52], but also cutouts of Haeckelite nanotubes [53].

Herein we report the synthesis of a series of precursors for very large **[n.n]CAPPs** (with up to 36 aromatic units). Their three‐dimensional conformations were analyzed by single crystal X‐ray diffraction up to the cyclic octamer. Furthermore, conditions to realize reductive aromatization of the trimeric and tetrameric precursors **[6.6]CAPP‐OH** and **[8.8]CAPP‐OH** to the respective nanohoops were tested.

## 2 Results and Discussion

### 2.1 Synthesis and Characterization

**SCHEME 1:**
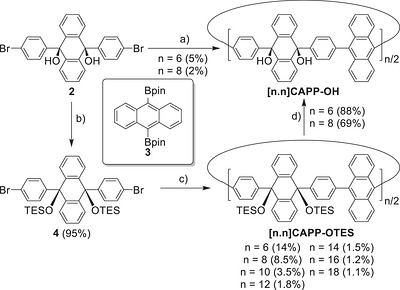
Synthesis of **[n.n]CAPP‐OTES** and **[n.n]CAPP‐OH**. (a) **3** (1 equiv), Pd_2_dba_3_ (10 mol%), XPhos (30 mol%), THF/2 M K_2_CO_3_, 80°C, o.n. (b) (i) NaH (60% wt., 4 equiv), THF, 0°C→50°C, 40 min, (ii) TESCl (6 equiv), THF, 50°C, o.n., (c) **3** (1 equiv), SPhos Pd G3 (10 mol%), THF/1 M K_3_PO_4_, 80°C, o.n., (d) TBAF·3H_2_O (3.1 equiv per diol), THF, r.t., 2.5 h.

To synthesize the outlined larger **[6.6]CAPP** and **[8.8]CAPP**, a direct synthesis of the corresponding non‐aromatic diol precursors **[n.n]CAPP‐OHs** (Scheme 1) was first conducted via a Suzuki–Miyaura cross‐coupling of diol **2** [54] and bisboronic ester **3** [55] using Pd_2_dba_3_ with XPhos as catalyst. Although the resulting trimeric **[6.6]CAPP‐OH** and tetrameric **[8.8]CAPP‐OH** were isolated by HPLC (with 50% THF/heptane as eluent) in 5% and 2% yields, their separation remained challenging due to their limited solubility in the eluent (1.4 mg/mL in pure THF for **[6.6]CAPP‐OH**).

**FIGURE 2:**
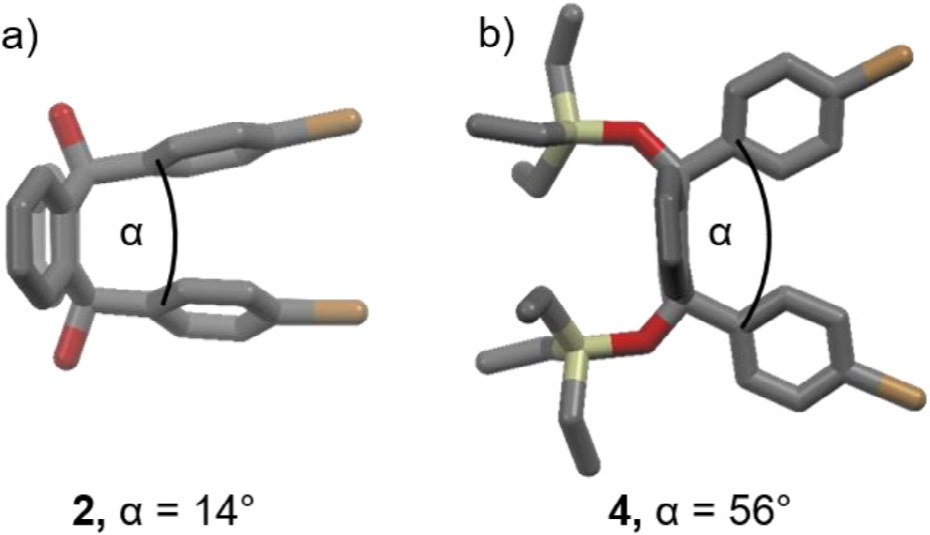
Single crystal X‐ray structure of (a) **2** and (b) **4** with their internal angles. Grey: carbon, red: oxygen, brown: bromine, beige: silicon. Hydrogens were omitted for clarity.

To increase the solubility of the resulting macrocycles, protection of the hydroxyl groups of the diol **2** was achieved with NaH and triethylsilyl chloride (TESCl) to give the silyl ether **4** in 95% yield. While the internal angle (of 14°) in the diol **2** (see Figure 2a) is identical to that of the diiodo derivative used in the synthesis of **[2.2]CAPP** [30], it is very different from the internal angle (of 66°) reported for a similar diol with chlorine substituents in the 1,5‐positions of the dihydroanthracene unit. The silylation changes its internal angle to 56° (for **4**, see Figure 2b), which is comparable to the 60° reported for the silyl ether of the chlorinated derivative [56].

**FIGURE 3:**
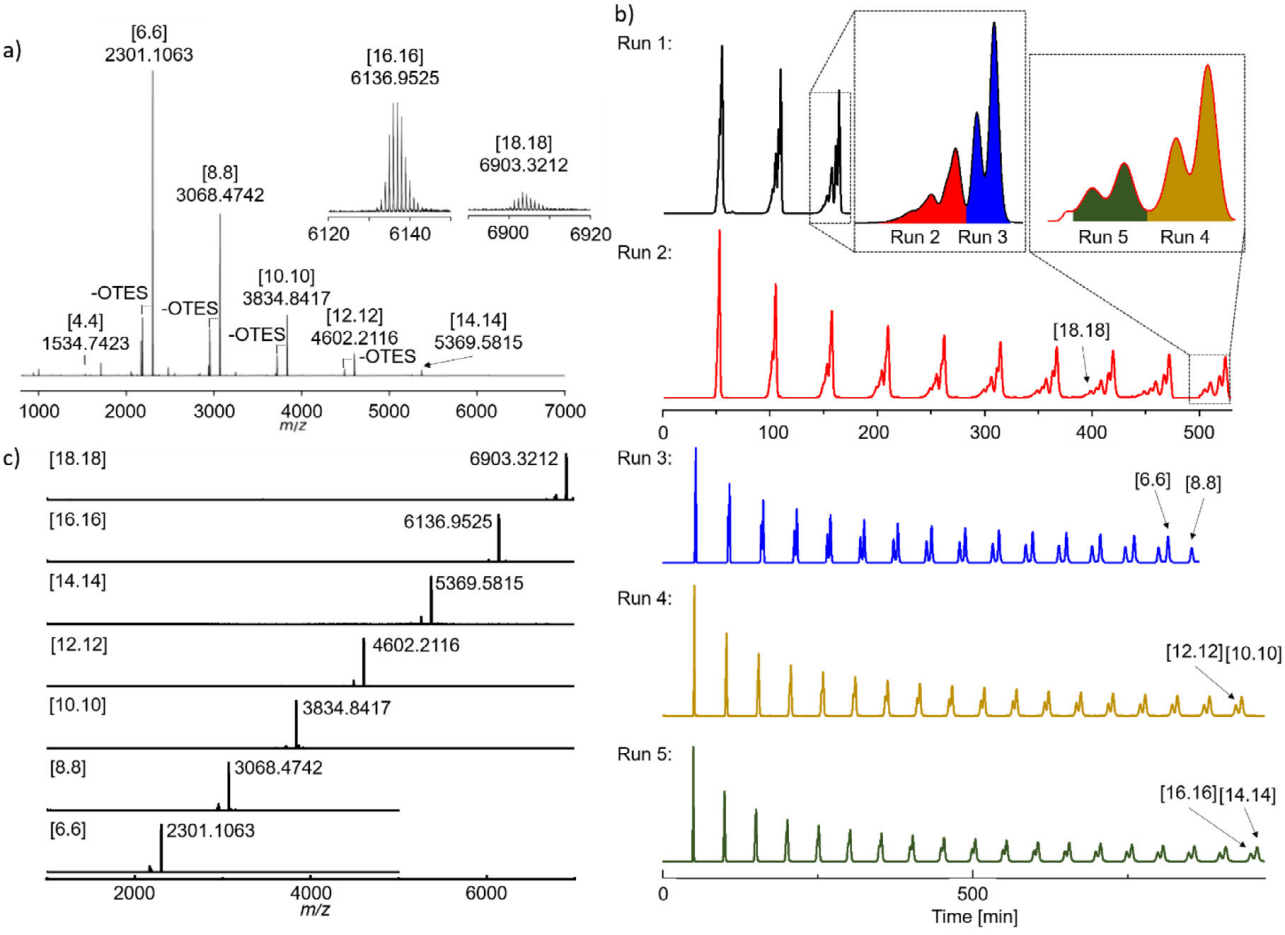
(a) HR‐MALDI‐TOF (DCTB matrix, pos. mode) of the crude reaction mixture of the **[n.n]CAPP‐OTES** synthesis. Due to the low intensity of **[16.16]CAPP‐OTES** and **[18.18]CAPP‐OTES** oligomers, zoomed in regions are shown for these compounds. The most abundant isotopic peak was picked for each compound. (b) Pre‐separation and separation of different **[n.n]CAPP‐OTES** oligomers by gel permeation chromatography. The fractions collected during the pre‐separations are highlighted in the color of the chromatograms of the corresponding separation. (c) HR‐MALDI‐TOF (DCTB matrix, pos. mode) of the **[n.n]CAPP‐OTES** after their separation (for larger spectra, see Figures S75–S81).

For the following Suzuki–Miyaura cross‐coupling with the silyl ether **4**, the previously used catalyst system (Pd_2_dba_3_ with XPhos) showed no macrocycle formation. Instead, SPhos Pd G3 was chosen as catalyst (see Figure S87 for comparable experiments with the unprotected diol **2**) [57, 58], and the formation of different macrocycles ranging from the dimeric **[4.4]CAPP‐OTES** (*m/z* 1534.7423, calcd. for [M+H]^+^:*m/z *1534.7431) up to the nonameric **[18.18]CAPP‐OTES** (*m/z* 6903.3212, calcd. for [M]^+^:*m/z *6903.3099, see Figure 3a) was found in the MALDI TOF MS of the crude product mixture, while peaks for the smaller **[2.2]CAPP‐OTES** were not observed. By GPC separation macrocycles from the trimeric **[6.6]CAPP‐OTES** up to the nonameric **[18.18]CAPP‐OTES** were isolated in decreasing yields from 14% for the trimer to 1.1% for the nonamer (For details, see Figure 3b, c). Subsequently, the most abundant, trimeric, and tetrameric macrocycles **[6.6]CAPP‐OTES** and **[8.8]CAPP‐OTES** were treated with tetrabutyl‐ammonium fluoride to obtain the corresponding polyols **[6.6]CAPP‐OH** and **[8.8]CAPP‐OH** in 88% and 69% yield. This route gives 12% and 6% yield over three steps and allows for better scalability, especially of the separation, due to the increased solubility of the compounds compared to those of the direct route (Scheme 1, path a).

**FIGURE 4:**
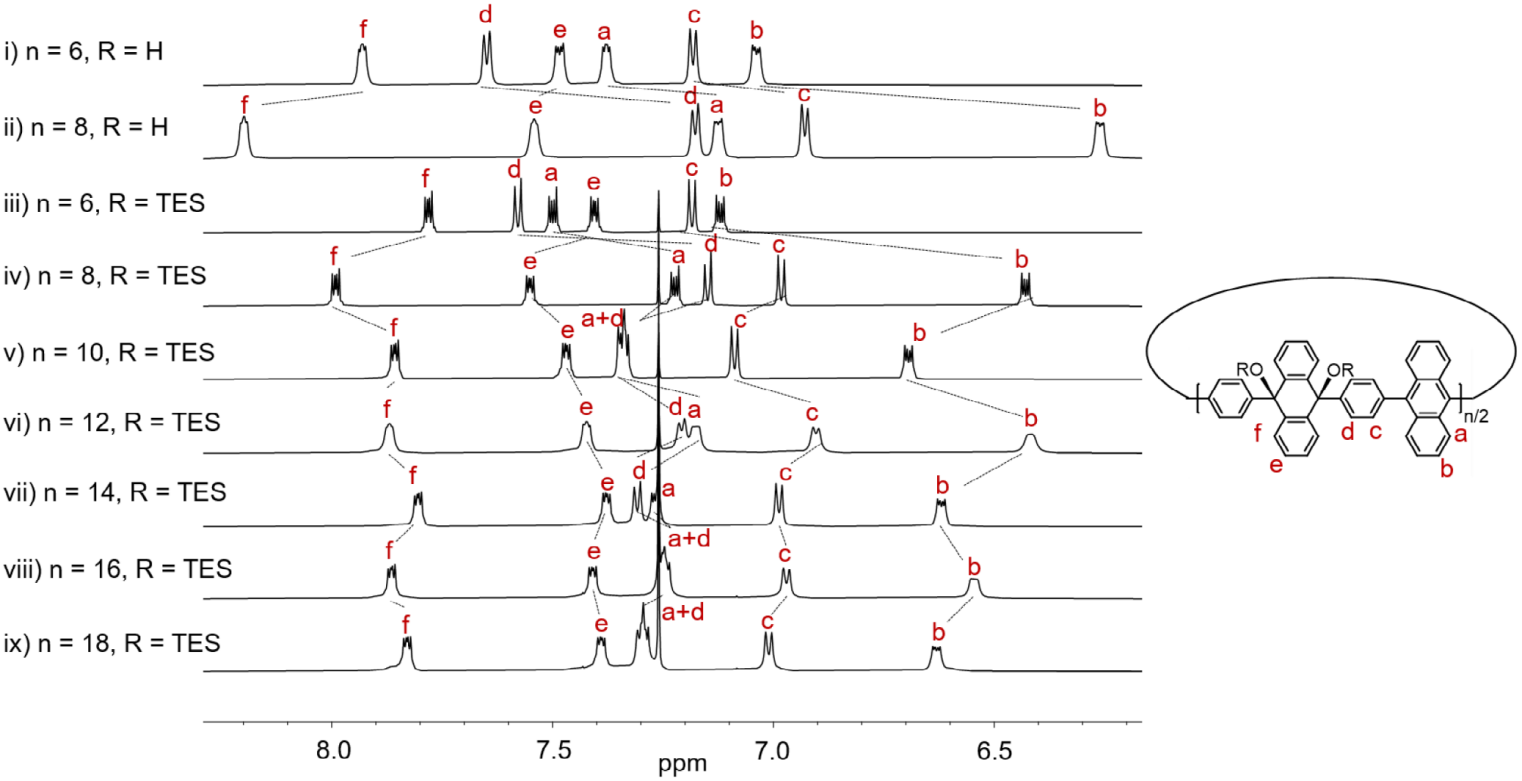
Aromatic regions of the ^1^H NMR spectra (600 MHz, 300 K) of (i and ii) **[6.6]CAPP‐OH** and **[8.8]CAPP‐OH** (THF‐d_8_) and (iii–ix) **[6.6]CAPP‐OTES** to **[18.18]CAPP‐OTES** (CDCl_3_) with proton assignments.

A comparison of the ^1^H NMR spectra of the various **CAPP‐OHs** and **CAPP‐OTES** (see Figure 4) showed a small effect of the silyl ethers. For the trimers (see spectra i and iii), only proton H^f^ shows a shift > 0.1 ppm of Δ*δ* = −0.16 ppm from **[6.6]CAPP‐OH** to **[6.6]CAPP‐OTES** and for the tetramers **[8.8]CAPP‐OH** and **[8.8]CAPP‐OTES** (see spectra ii and iv) only protons H^b^ and H^f^ change by Δ*δ* = +0.17 and −0.21 ppm. Within the series from **[6.6]CAPP‐OTES** to **[18.18]CAPP‐OTES** an even–odd trend can be observed, which is especially pronounced for protons H^b^, H^c^, and H^f^. This trend is the strongest for the smaller cycles, where proton H^b^ shifts from *δ* = 7.11 ppm (in **[6.6]CAPP‐OTES**) to *δ* = 6.43 ppm (in **[8.8]CAPP‐OTES**) to *δ* = 6.69 ppm (in **[10.10]CAPP‐OTES**). In contrast, a much weaker shift (of Δ*δ *= +0.08 ppm) is observed for H^b^ between the two largest cycles **[16.16]CAPP‐OTES** and **[18.18]CAPP‐OTES** (see spectra viii and ix).

**FIGURE 5:**
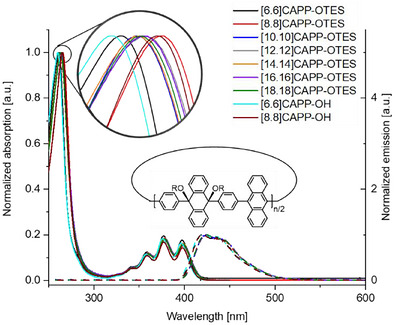
Absorption (solid lines) and emission (dashed lines, excitation wavelength: 253 nm) spectra of **[6.6]CAPP‐OTES**‐**[18.18]CAPP‐OTES** in dichloromethane and **[6.6]CAPP‐OH** and **[8.8]CAPP‐OH** in tetrahydrofuran. The peaks between 261 and 265 nm are enlarged to highlight their shift with the size of the macrocycle.

UV/Vis absorption spectroscopy shows very similar spectra for all **[n.n]CAPP‐OTES** and **[n.n]CAPP‐OH** with several peaks between *λ*_abs_ = 340 and 400 nm (see Figure 5), which are close to the ones typically observed for 9,10‐diphenylanthracene (between *λ*_abs_ = 338 and 394 nm) [59]. Upon closer inspection, the highest energy peak of **[6.6]CAPP‐OTES** and **[6.6]CAPP‐OH** is slightly blue‐shifted (at *λ*_abs_ = 261 and 260 nm) and the one of **[8.8]CAPP‐OTES** and **[8.8]CAPP‐OH** is slightly red‐shifted (both at *λ*_abs_ = 265 nm) compared to all other members of this series (at *λ*_abs_ = 263–264 nm). This is similar to the trend discussed above for the ^1^H NMR spectra, where the smallest macrocycles showed the strongest even–odd trend. In the emission spectra, **[8.8]CAPP‐OH** and all **[n.n]CAPP‐OTES** except **[6.6]CAPP‐OTES** have two peaks at *λ*_em_ = 424 and 438–441 nm. The smallest cycles **[6.6]CAPP‐OTES** and **[6.6]CAPP‐OH** are an exception, as the emission is slightly red‐shifted to *λ*_em_ = 419 or 420 and 436 or 437 nm and thereby closer to the emission of 9,10‐diphenylanthracene (at *λ*_em_ = 407 and 430 nm) [59]. The fluorescence quantum yields are between *Φ* = 56% for **[8.8]CAPP‐OH** and 27% for **[16.16]CAPP‐OTES** (see Table 1).

**TABLE 1:** Comparison of key data of **[n.n]CAPP‐OTES**, **[n.n]CAPP‐OH**, and **[6.6]CAPP‐OTES‐O_2_**.

Compound	*λ*_max_ (nm)	*ν*_Stokes_ (cm^−1^)	*λ*_em_ (nm)	*Φ*_F_ (%)	*d*_Hb‐anthracene_ (Å)^a^	*δ*(H^b^) (ppm)
**[6.6]CAPP‐OTES**	396	1386	419, 436	54 ± 10	8.2, 9.3	7.11
**[8.8]CAPP‐OTES**	397	1604	424, 439	33 ± 8	3.7	6.43
**[10.10]CAPP‐OTES**	398	1541	424, 439	30 ± 9	2.7, 3.1, 4.2	6.69
**[12.12]CAPP‐OTES**	400	1415	424, 439	45 ± 9	2.8, 3.0, 3.8	6.41
**[14.14]CAPP‐OTES**	399	1478	424, 439	38 ± 9	2.5, 2.9, 3.1, 3.3	6.62
**[16.16]CAPP‐OTES**	399	1478	424, 441	27 ± 8	2.9, 3.1	6.55
**[18.18]CAPP‐OTES**	399	1478	424, 439	29 ± 7	—	6.63
**[6.6]CAPP‐OH**	396	1443	420, 437	45 ± 10	—	7.04
**[8.8]CAPP‐OH**	397	1436	421, 438	56 ± 7	—	6.26
**[6.6]CAPP‐OTES‐O_2_**	263	—	—	—	—	6.96

^a^ Distances of proton H^b^ and the closest anthracene in the single crystal X‐ray structure, either H^b^‐π or H^b^‐C, dependent on the relative angles of the anthracene units (see also Figure 6).

### 2.2 Single Crystal X‐Ray Diffraction

**FIGURE 6:**
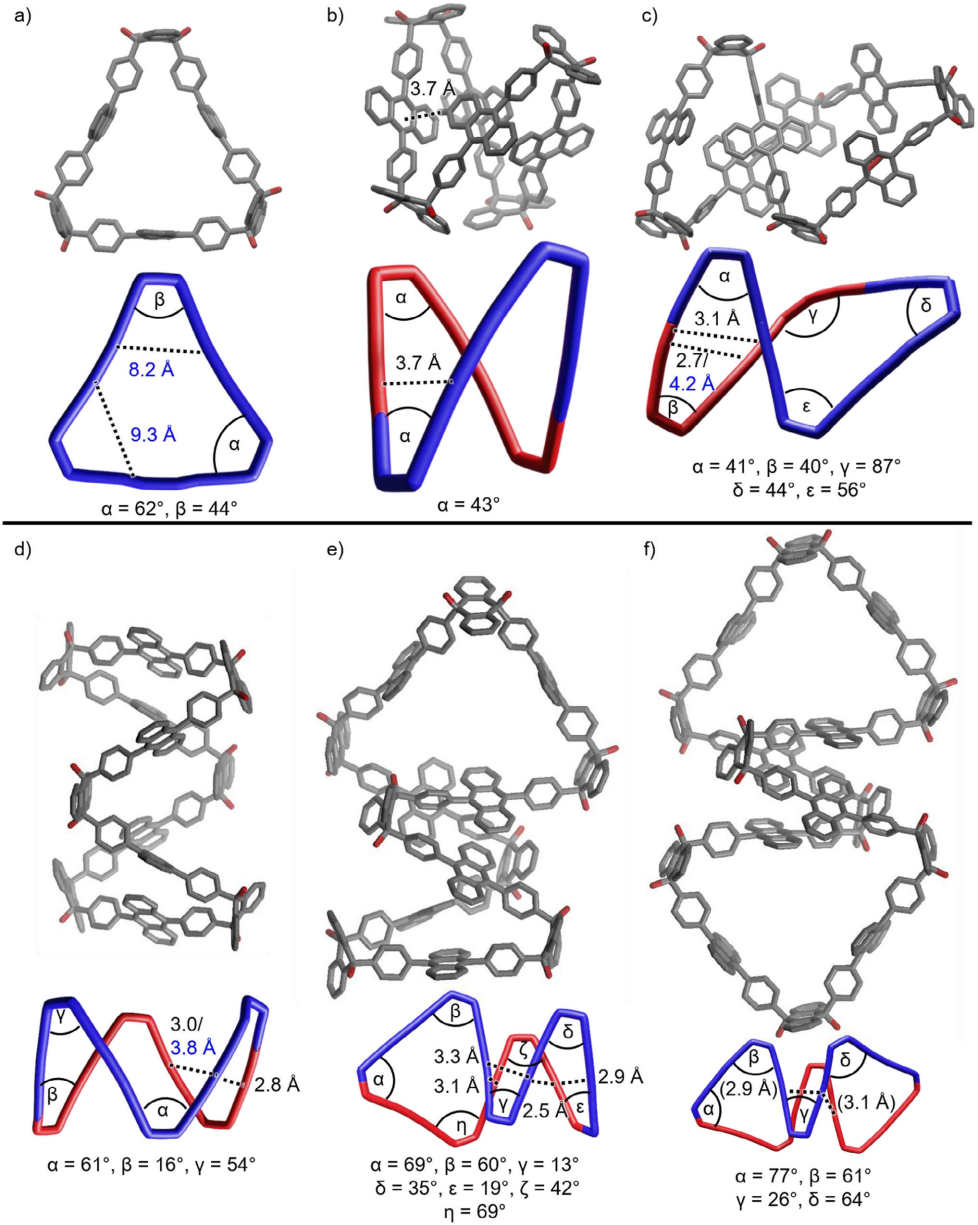
Single crystal X‐ray structures of (a) **[6.6]CAPP‐OTES**—(f) **[16.16]CAPP‐OTES** as capped stick models. Hydrogens were omitted for clarity. Grey: carbon, red: oxygen, beige: silicon, white: hydrogen. Additionally, all macrocycles are shown schematically to visualize the internal angles of the corner units and the intramolecular distances of proton H^b^ and the closest anthracene (either H^b^–π (black) or H^b^–C (blue, closest carbon), dependent on the relative angles of the anthracenes). This distance is exemplarily shown in the structure of **[8.8]CAPP‐OTES** in (b). Due to the lower crystal quality of **[16.16]CAPP‐OTES**, only approximate distances can be given. The parts of the molecule closer to the viewer are shown in blue and the parts in the background in red.

Crystals suitable for single crystal X‐ray diffraction analyses were grown by diffusion of methanol into *o*DCB solutions of **[6.6]CAPP‐OTES—[16.16]CAPP‐OTES** (see Figure 6). The smallest congener **[6.6]CAPP‐OTES** crystallized together with its one fold peroxidized form in the space group *C*2/c with *Z* = 4. The peroxide occupies 59% of the anthracene units parallel to the crystallographic *a*‐axis (for crystallographic details see Figures S103 and S104, for details regarding the peroxidation see below). While **[6.6]CAPP‐OTES** showed the expected planar triangular geometry, the larger cycles adopted nonplanar geometries, as their internal angles are much smaller than the internal angles of the corresponding regular polygons. The tetramer **[8.8]‐CAPP‐OTES**, which crystallizes in the space group $I\bar{4}$ with *Z* = 2, consequently has a tub conformation, similar to the one reported for tetrameric porphyrin nanorings [60]. Thus, all anthracene units of a molecule are in close proximity (with a distance of 3.7 Å between H^b^ in one and the π‐plane in another anthracene unit of the same molecule), which probably causes the strong change in chemical shift for H^b^ (see ^1^H NMR discussion above). Similarly to **[6.6]CAPP‐OTES**, the larger pentamer **[10.10]CAPP‐OTES** crystallized together with its one‐fold peroxide (modelled with 48% occupancy, for details see Supporting Information page S75) in the $P\bar{1}$ space group with *Z* = 2 and adopts a figure of eight conformation, in which two of the anthracene units are in close proximity to a third anthracene (with intramolecular H^b^‐π distances of 2.7 and 3.1 Å), while the remaining anthracenes are further apart. Assuming the conformation in the solid state is the most abundant one in solution, this explains the less pronounced upfield shift of the corresponding proton H^b^ (see above), compared to the smaller **[8.8]CAPP‐OTES**. The hexameric **[12.12]CAPP‐OTES** crystallizes in the $P\bar{1}$ space group with *Z* = 1. An inversion center in the molecule, leads to a zigzag conformation in its central part, in which the upper half crosses the lower half twice. This results in small H^b^–π or H^b^–C distances (of 2.8 to 3.8 Å) for all anthracene units, which is similar to the distance observed for **[8.8]CAPP‐OTES** and corresponds well with the similar chemical shift of H^b^ in **[8.8]CAPP‐OTES** and **[12.12]CAPP‐OTES** under the assumption of similar conformations in the solid state and solution. The heptameric **[14.14]CAPP‐OTES** crystallizes in the $P\bar{1}$ space group with *Z* = 2. Its conformation increases further in complexity and can be described as a combination of the trimeric, triangular structure of **[6.6]CAPP‐OTES** and the zigzag structure observed for **[12.12]CAPP‐OTES**. Consequently, short intramolecular H^b^–π distances (between 2.5 and 3.3 Å) are observed near the crossing points and larger ones in the triangular section, which results in similar chemical shift of proton H^b^ compared to the one in **[10.10]CAPP‐OTES**. Similarly, the octamer **[16.16]CAPP‐OTES** crystallizes in the $P\bar{1}$ space group with *Z* = 1 and can be described as a combination of two triangular structures with the zigzag motif observed for **[12.12]CAPP‐OTES** and **[14.14]CAPP‐OTES**. This conformation results in close intramolecular H^b^–π distances of approximately 2.9 and 3.1 Å in the center of the molecule, similar to the ones in the hexameric **[12.12]CAPP‐OTES** and heptameric **[14.14]CAPP‐OTES**, while the triangular sections show much larger distances. As there are two triangular sections, this results in a lower, averaged chemical shift of proton H^b^ compared to both **[10.10]CAPP‐OTES** and **[14.14]CAPP‐OTES**.

Generally, the strong changes in chemical shift for H^b^ observed in ^1^H NMR spectroscopy show a correlation with the average H^b^‐anthracene distances in the solid state, which are dependent on the molecules folding pattern. This folding pattern strongly influences the internal angles of the corner units as well. While the smaller cycles (**[6.6]CAPP‐OTES** and **[8.8]CAPP‐OTES**) adopt simple shapes, their internal angles are between 43° and 62° (see Figure 6 and Table 1) and thereby similar to the one in the building block (at 56°). Meanwhile the larger cycles show a much higher flexibility. There, most angles fall in a range from 40° to 61°, but there are outliers, which range from 13° to 87°. While **[10.10]CAPP‐OTES**, as the first bridged molecule, has one outlier widened to 87°, the zigzag motive observed for **[12.12]CAPP‐OTES**—**[16.16]CAPP‐OTES** reduces the internal angle of two corner units to 13°–26° and at the same time widens two angles to 64°–77° in **[14.14]CAPP‐OTES** and **[16.16]CAPP‐OTES**.

**FIGURE 7:**
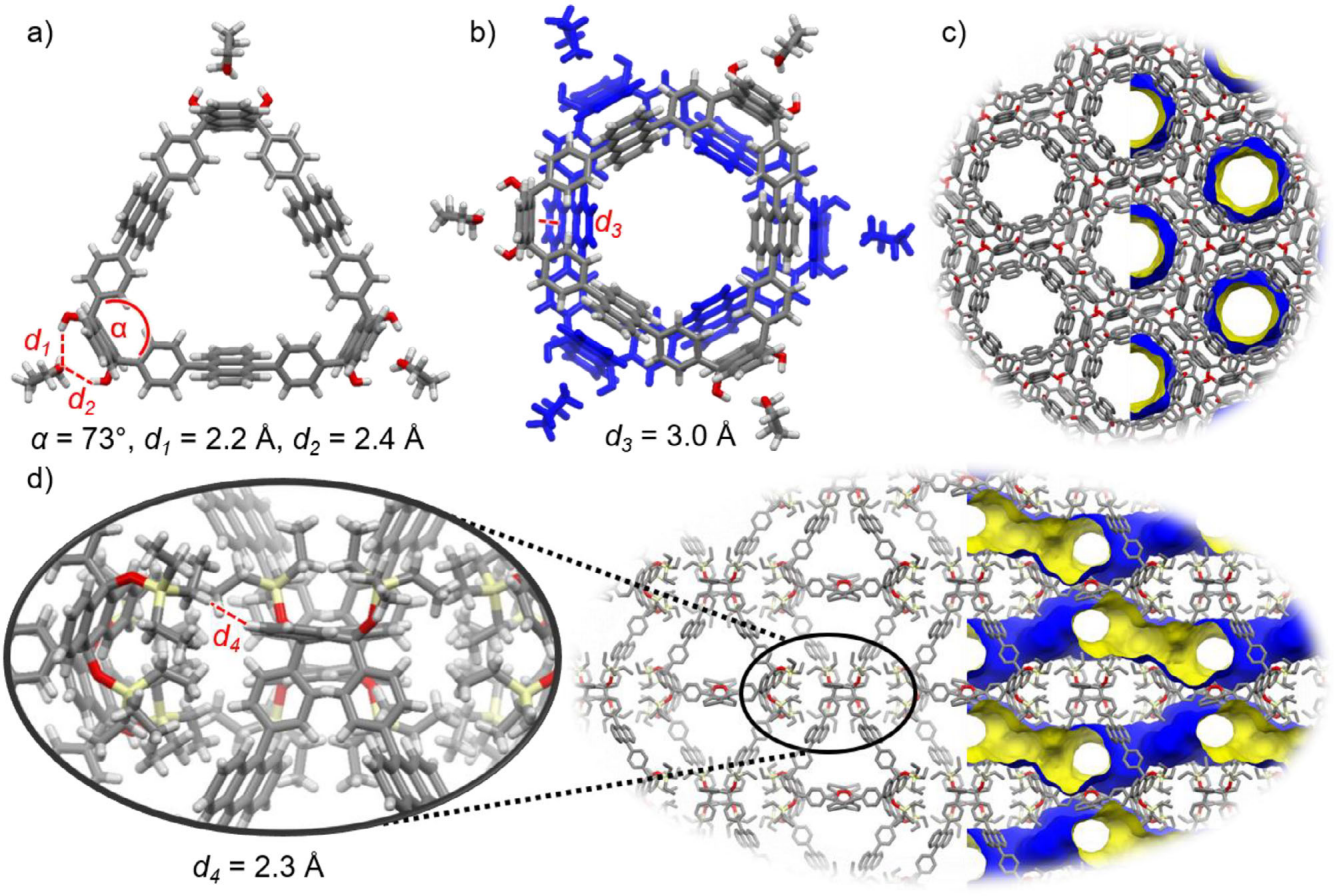
Single crystal X‐ray structures of **[6.6]CAPP‐OH** as capped stick model viewed along the crystallographic *c*‐axis. (a) one **[6.6]CAPP‐OH** with internal angle and the hydrogen bonds to the surrounding tetrahydrofuran molecules, (b) **[6.6]CAPP‐OH** dimer with one molecule highlighted in blue, to show the dimeric structure, packing, and voids (probe radius 1.82 Å, grid spacing 0.5 Å) of (c) **[6.6]CAPP‐OH** and (d) porous structure of **[6.6]CAPP‐OTES** (with zoom in on the intermolecular van der Waals interactions). Grey: carbon, red: oxygen, beige: silicon, white: hydrogen. Hydrogens were omitted for clarity in the subfigures depicting the packing.

In contrast to the TES protected **[6.6]CAPP‐OTES**, which crystalizes in the space group *C*2/c with *Z *= 4, the deprotected **[6.6]CAPP‐OH** (see Figure 7a–c) crystalizes in the space group $R\bar{3}$ with *Z *= 3. The diol has similar intramolecular distances of the anthracene units to the ones in its silyl ether congener, which corresponds well with the strong similarities of these compounds in both UV/vis (with Δ*λ*_max_ = 1 nm, see discussion above) and ^1^H NMR spectroscopy (with Δ*δ*_max_ = 0.16 ppm, see discussion above). In the solid state, three tetrahydrofuran molecules form hydrogen bonds with the diol units of **[6.6]CAPP‐OH** (with O‐H···O distances of 2.2 and 2.4 Å). The resulting reduced steric demand compared to the silyl ethers in **[6.6]CAPP‐OTES** enlarges the internal angles in **[6.6]CAPP‐OH** (*α* = 73°) compared to **[6.6]CAPP‐OTES** (*α* = 62° and *β =* 42°). This reduced steric demand also strongly influences the packing. **[6.6]CAPP‐OH** forms dimeric structures in the crystal (see Figure 7b), which are arranged similar to a hexagonal closest packing (see Figure 7c), where the surrounding dimers are shifted slightly up or down along the crystallographic *c*‐axis. The larger steric repulsion of the silyl ethers of **[6.6]CAPP‐OTES** prevents the dimer formation. Instead, van der Waals interactions between the ethyl chains and dihydroanthracene units dominate the packing (see Figure 7d). This difference in packing strongly influences the voids of the structure as well. **[6.6]CAPP‐OH** has tubular voids along the crystallographic *c*‐axis (see Figure 7c). Contrary to this, the silyl ether **[6.6]CAPP‐OTES** crystallizes with a two‐dimensional network of voids with an opening along the *c*‐axis (see Figure 7d). Although both structures show very large calculated BET surface areas of 1440 and 1905 m^2^g^−1^, preliminary measurements by nitrogen sorption at 77 K gave negligible experimental BET surface areas of 4 m^2^g for **[6.6]CAPP‐OTES** and 34 m^2^g^−1^ for **[6.6]CAPP‐OH**. Although the porosity of these structures is potentially interesting, it is not focused herein and will be further investigated in due course.

### 2.3 Peroxidation

**FIGURE 8:**
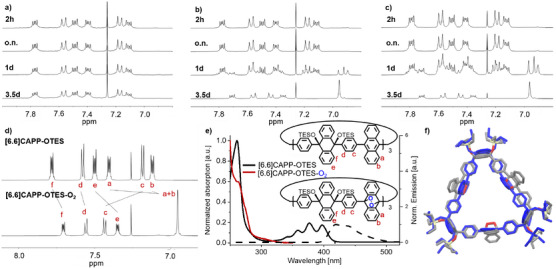
Aromatic regions of the ^1^H NMR spectra (300 MHz, CDCl_3_, 300 K) of **[6.6]CAPP‐OTES** at various times while exposed to sunlight under (a) argon atmosphere, (b) air, and (c) oxygen atmosphere. (d) Aromatic regions of the ^1^H NMR spectra (600 MHz, CDCl_3_, 300 K) of **[6.6]CAPP‐OTES** and threefold endoperoxide **[6.6]CAPP‐OTES‐O_2_** with signal assignments. (e) Absorption spectra of **[6.6]CAPP‐OTES** and **[6.6]CAPP‐OTES‐O_2_** and emission spectrum of **[6.6]CAPP‐OTES** (dotted line) in dichloromethane. (f) Single crystal x‐ray structure of **[6.6]CAPP‐OTES‐O_2_**.(in element colors) overlayed with the structure of **[6.6]CAPP‐OTES** (in blue). Gray: carbon, red: oxygen, brown: bromine, beige: silicon. Hydrogens were omitted for clarity.

All members of the **[n.n]CAPP‐OTES** series were oxidized when exposed to air (as was seen by the co‐crystallization of **[6.6]CAPP‐OTES** and **[10.10]CAPP‐OTES** with their one‐fold peroxidized congeners; see above). Consequently, this was studied by placing a sample of **[6.6]CAPP‐OTES** dissolved in CDCl_3_ under either an argon, an air or a pure oxygen atmosphere and monitoring the ^1^H NMR spectra of these samples over time (see Figure 8a–c). While the sample under argon showed no new NMR signals between *δ = *6.8 and 7.0 ppm, the samples under air and oxygen showed new signals in this region, which merged into only one singlet after 3.5 days. This suggests the full conversion to the endoperoxide **[6.6]CAPP‐OTES‐O_2_**. Similarly, endoperoxide formation was previously reported for 9,10‐diphenylanthracenes [35, 36, 37]. This is likely due to both the aromatic stabilization energy per π‐electron and the hydrogenation energy gradually decreasing in the series from benzene to anthracene and the resulting low stability of the central anthracene ring [61, 62]. The conversion to the peroxide was repeated on a larger scale to obtain analytically pure samples of **[6.6]CAPP‐OTES‐O_2_** in 97% yield. The protons close to the silyl ether (H^d^–H^f^) of **[6.6]CAPP‐OTES‐O_2_** show only small changes in chemical shielding (Δ*δ* ≤ 0.06 ppm) compared to **[6.6]CAPP‐OTES** (see Figure 8d), while the protons closer to the endoperoxide experience a stronger shift, for example, H^c^ (from *δ* = 7.18 to 7.44 ppm) or H^a^ (from *δ =* 7.49 to 6.96 ppm). The peroxides UV/Vis absorption spectrum is simplified to only a single peak at *λ*_abs_ = 263 nm and the compounds fluorescence is quenched (see Figure 8e), likely due to the largest aromatic subunits of **[6.6]CAPP‐OTES** being reduced in size to only benzene moieties in the peroxide. Interestingly, all dioxo bridges of **[6.6]CAPP‐OTES‐O_2_** point into the cycle as confirmed by single crystal X‐ray diffraction analysis (see Figure 8f). This leads to larger internal angles compared to the nonoxidized **[6.6]CAPP‐OTES** (shown in blue).

## 3 Reductive Aromatization

**FIGURE 9:**
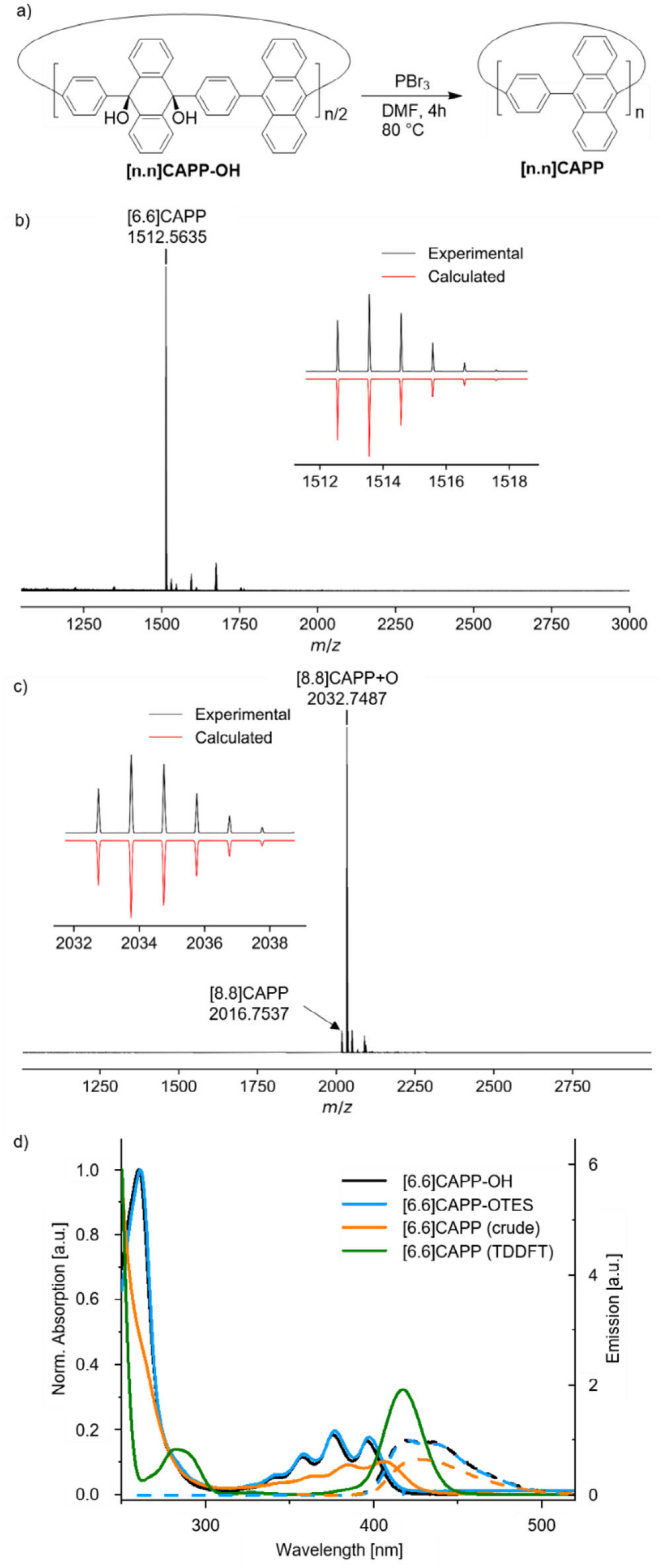
(a) Rearomatization of **[6.6]CAPP‐OH** and **[8.8]CAPP‐OH** to **[6.6]CAPP** and **[8.8]CAPP**. (b and c) HR MALDI‐TOF MS (DCTB matrix, pos. mode) of (b) **[6.6]CAPP** and (c) **[8.8]CAPP**. (d) Absorption (solid lines) and emission (dashed lines) spectra of **[6.6]CAPP‐OH** (in tetrahydrofuran, black) **[6.6]CAPP‐OTES** (in dichloromethane, blue) and crude **[6.6]CAPP** (in dichloromethane, orange). The TDDFT calculated spectrum (PBE0‐D3BJ/6‐311G(d) with dichloromethane solvation) of **[6.6]CAPP** is shown in green.

As the route via the silyl ethers allowed the synthesis of suitable amounts of **[6.6]CAPP‐OH** and **[8.8]CAPP‐OH** for further experiments (see above for synthetic details), the reduction to the aromatic **[6.6]CAPP** and **[8.8]CAPP** was attempted using sodium hypophosphite as reducing agent [33], giving only a one‐fold hydrogenated species of **[6.6]CAPP** as determined by MALDI‐TOF MS (*m/z* 1516.885, calcd. for [M+3H]^+^:1516.590) as well as for **[8.8]CAPP** (at *m/z* 2020.704, calcd. for for [M+3H]^+^: 2020.778). This observation is similar to the overreduction described for other highly strained 9,10‐anthracene based nanohoops [33] and likely due to the lower aromatization energy of anthracene compared to benzene [61, 62]. Recently, an alternative strategy, based on a bromination with PBr_3_ followed by a reduction with zinc, was reported for the smaller **[2.2]CAPP** [30]. To our delight, the first step (see Figure 9a) leads already to direct formation of the aromatic **[6.6]CAPP** and **[8.8]CAPP** instead of bromination, as confirmed by MALDI‐TOF MS. For **[6.6]CAPP** the molecular ion peak (at *m/z* 1512.5635 (calcd. for [M]^+^: 1512.5629) was the main peak (see Figure 9b). In contrast, for **[8.8]CAPP** (see Figure 9c) only a small peak was detected for the rearomatized product (at *m/z* 2016.7537, calcd. for [M]^+^: 2016.7507) and a larger signal was found for a species containing one additional oxygen atom (at *m/z* 2032.7487, calcd. for [M+O]^+^: 2032.7456). Unfortunately, the isolation of the aromatic compounds proved unsuccessful due to their chemical instability. As soon as the compound is taken from the reaction mixture, decomposition products are observed (see Supporting Information S88).

In UV/Vis absorption spectroscopy the resulting crude **[6.6]CAPP** (see Figure 9d) has no peak corresponding to the planar phenylene units (e.g., at *λ*_abs_ = 261 nm for **[6.6]CAPP‐OTES**), which is also observed for CPPs, for example, [9]CPP, [12]CPP, and [18]CPP, where the absorption maximum for the curved phenylenes is shifted to *λ*_abs_ = 340 nm from *λ*_abs_ = 255 nm in benzene [1, 59]. This change from planar to curved structure corresponds well with the strain energy of 59.0 kcal/mol for **[6.6]CAPP**, which is much higher than for the nearly unstrained **[6.6]CAPP‐OH** (with 1.3 kcal/mol, both calculated at B3LYP/6‐311G(d) level of theory) and comparable to that of [10]CPP (at 57.7 kcal/mol) [50]. The lowest energy band of **[6.6]CAPP** is shifted from *λ*_abs_ = 396 nm in its precursors to *λ*_abs_ = 406 nm likely due to the higher conjugation in the system. TDDFT calculations (at PBE0‐D3BJ/6‐311G(d) level of theory with DCM or THF solvation) for the most red‐shifted peak match well with the experimentally observed values for both **[6.6]CAPP** (*λ*_abs_: calcd.: 417 nm, exp.: 406 nm, see Figure 9d) and **[6.6]CAPP‐OH** (*λ*_abs_: calcd.: 401 nm, exp.: 396 nm). A similar shift can be observed for the emission spectrum, where the highest energy maximum is red‐shifted from *λ*_em_ = 420 nm in **[6.6]CAPP‐OH** to *λ*_em_ = 427 nm by the rearomatization. This results in a slightly reduced Stokes shift of *ṽ*_Stokes_ = 1211 cm^−1^, which can be explained by the higher rigidity due to the higher strain in the rearomatized **[6.6]CAPP**.

## 4 Conclusion

In summary, a series of silyl protected macrocycles (**[6.6]CAPP‐OTES—[18.18]CAPP‐OTES**) has been synthesized by a one‐pot reaction and studied by single crystal X‐ray diffraction. It has been found that these new CAPPs are prone to the formation of endoperoxides, making a generation of the corresponding aromatic nanohoops very challenging, which has been achieved only for the **[6.6]CAPP** in solution so far. The small intramolecular distances between diphenylanthracene units could be beneficial for triplet–triplet annihilator materials [63].

## Conflicts of Interest

The authors declare no conflicts of interest.

## Supporting information




**Supplementary File 1**: The authors have cited additional references within the Supporting Information [50, 54, 55, 64, 65, 66, 67, 68, 69, 70, 71, 72, 73, 74, 75, 76, 77, 78, 79, 80, 81, 82, 83, 84, 85, 86, 87, 88, 89, 90, 91, 92, 93, 94, 95, 96, 97, 98, 99, 100, 101, 102, 103, 104, 105, 106, 107, 108, 109, 110, 111, 112, 113, 114, 115, 116, 117, 118, 119, 120, 121, 122, 123, 124, 125, 126]. The supporting crystallographic data for this paper are provided free of charge by the joint Cambridge Crystallographic Data Centre and Fachinformationszentrum Karlsruhe [127].

## Data Availability

The data that support the findings of this study are available from the corresponding author upon reasonable request.

## References

[1] R. Jasti , J. Bhattacharjee , J. B. Neaton , and C. R. Bertozzi , “Synthesis, Characterization, and Theory of [9]‐, [12]‐, and [18]Cycloparaphenylene: Carbon Nanohoop Structures,” Journal of the American Chemical Society 130 (2008): 17646–17647, 10.1021/ja807126u. 19055403 PMC2709987

[2] E. Kayahara , Y. Cheng , and S. Yamago , “Short‐Step Synthesis of Large Cycloparaphenylenes,” Chemistry Letters 47 (2018): 1108–1111, 10.1246/cl.180486.

[3] T. Iwamoto , Y. Watanabe , Y. Sakamoto , T. Suzuki , and S. Yamago , “Selective and Random Syntheses of [n]Cycloparaphenylenes (n = 8–13) and Size Dependence of Their Electronic Properties,” Journal of the American Chemical Society 133 (2011): 8354–8361, 10.1021/ja2020668. 21542589

[4] S. Yamago , Y. Watanabe , and T. Iwamoto , “Synthesis of [8]Cycloparaphenylene From a Square‐Shaped Tetranuclear Platinum Complex,” Angewandte Chemie International Edition 49 (2010): 757–759, 10.1002/anie.200905659. 20014269

[5] T. Sisto , M. R. Golder , E. S. Hirst , and R. Jasti , “Selective Synthesis of Strained [7]Cycloparaphenylene: An Orange‐Emitting Fluorophore,” Journal of the American Chemical Society 133 (2011): 15800–15802, 10.1021/ja205606p. 21913694

[6] J. Xia and R. Jasti , “Synthesis, Characterization, and Crystal Structure of [6]Cycloparaphenylene,” Angewandte Chemie International Edition 51 (2012): 2474–2476, 10.1002/anie.201108167. 22287256

[7] E. Kayahara , V. K. Patel , and S. Yamago , “Synthesis and Characterization of [5]Cycloparaphenylene,” Journal of the American Chemical Society 136 (2014): 2284–2287, 10.1021/ja413214q. 24460371

[8] P. J. Evans , E. R. Darzi , and R. Jasti , “Efficient Room‐Temperature Synthesis of a Highly Strained Carbon Nanohoop Fragment of Buckminsterfullerene,” Nature Chemistry 6 (2014): 404–408, 10.1038/nchem.1888. 24755591

[9] H. Omachi , S. Matsuura , Y. Segawa , and K. Itami , “A Modular and Size‐Selective Synthesis of [n]Cycloparaphenylenes: A Step Toward the Selective Synthesis of [n, n] Single‐Walled Carbon Nanotubes,” Angewandte Chemie International Edition 49 (2010): 10202–10205, 10.1002/anie.201005734. 21105035

[10] E. S. Hirst and R. Jasti , “Bending Benzene: Syntheses of [n]Cycloparaphenylenes,” Journal of Organic Chemistry 77 (2012): 10473–10478, 10.1021/jo302186h. 23126565

[11] M. R. Golder and R. Jasti , “Syntheses of the Smallest Carbon Nanohoops and the Emergence of Unique Physical Phenomena,” Accounts of Chemical Research 48 (2015): 557–566, 10.1021/ar5004253. 25689579

[12] Y. Segawa , A. Yagi , and K. Itami , “Chemical Synthesis of Cycloparaphenylenes,” Physical Sciences Reviews 2 (2017): 1–24.

[13] Y. Luan and H. Cong , “Recent Synthetic Advances on π‐Extended Carbon Nanohoops,” Synlett 28 (2017): 1383–1388, 10.1055/s-0036-1588978.

[14] M. Hermann , D. Wassy , and B. Esser , “Conjugated Nanohoops Incorporating Donor, Acceptor, Hetero‐ or Polycyclic Aromatics,” Angewandte Chemie International Edition 60 (2021): 15743–15766, 10.1002/anie.202007024. 32902109 PMC9542246

[15] S. E. Lewis , “Cycloparaphenylenes and Related Nanohoops,” Chemical Society Reviews 44 (2015): 2221–2304, 10.1039/C4CS00366G. 25735813

[16] D. Wu , W. Cheng , X. Ban , and J. Xia , “Cycloparaphenylenes (CPPs): An Overview of Synthesis, Properties, and Potential Applications,” Asian Journal of Organic Chemistry 7 (2018): 2161–2181, 10.1002/ajoc.201800397.

[17] A. Yagi , Y. Segawa , and K. Itami , “Synthesis and Properties of [9]Cyclo‐1,4‐Naphthylene: A π‐Extended Carbon Nanoring,” Journal of the American Chemical Society 134 (2012): 2962–2965, 10.1021/ja300001g. 22296245

[18] H. Jia , Y. Gao , Q. Huang , S. Cui , and P. Du , “Facile Three‐Step Synthesis and Photophysical Properties of [8]‐, [9]‐, and [12]Cyclo‐1,4‐Naphthalene Nanorings via Platinum‐Mediated Reductive Elimination,” Chemical Communications 54 (2018): 988–991, 10.1039/C7CC07370D. 29322132

[19] K. Okada , A. Yagi , Y. Segawa , and K. Itami , “Synthesis and Properties of [8]‐, [10]‐, [12]‐, and [16]Cyclo‐1,4‐Naphthylenes,” Chemical Science 8 (2017): 661–667, 10.1039/C6SC04048A. 28451214 PMC5297897

[20] J. M. Batson and T. M. Swager , “Towards a Perylene‐Containing Nanohoop,” Synlett 24 (2013): 2545–2549.

[21] B. Farajidizaji , C. Huang , H. Thakellapalli , et al., “Synthesis and Characterization of Functionalized [12]Cycloparaphenylenes Containing Four Alternating Biphenyl and Naphthyl Units,” Journal of Organic Chemistry 82 (2017): 4458–4464, 10.1021/acs.joc.7b00397. 28357851

[22] C. Huang , Y. Huang , N. G. Akhmedov , B. V. Popp , J. L. Petersen , and K. K. Wang , “Functionalized Carbon Nanohoops: Synthesis and Structure of a [9]Cycloparaphenylene Bearing Three 5,8‐Dimethoxynaphth‐1,4‐Diyl Units,” Organic Letters 16 (2014): 2672–2675, 10.1021/ol500904x. 24785776

[23] S. Li , C. Huang , H. Thakellapalli , et al., “Syntheses and Structures of Functionalized [9]Cycloparaphenylenes as Carbon Nanohoops Bearing Carbomethoxy and N ‐Phenylphthalimido Groups,” Organic Letters 18 (2016): 2268–2271, 10.1021/acs.orglett.6b00904. 27101316

[24] H. Omachi , Y. Segawa , and K. Itami , “Synthesis and Racemization Process of Chiral Carbon Nanorings: A Step Toward the Chemical Synthesis of Chiral Carbon Nanotubes,” Organic Letters 13 (2011): 2480–2483, 10.1021/ol200730m. 21486080

[25] Z. Sun , P. Sarkar , T. Suenaga , S. Sato , and H. Isobe , “Belt‐Shaped Cyclonaphthylenes,” Angewandte Chemie International Edition 54 (2015): 12800–12804, 10.1002/anie.201506424. 26333160

[26] Z. Sun , T. Suenaga , P. Sarkar , S. Sato , M. Kotani , and H. Isobe , “Stereoisomerism, Crystal Structures, and Dynamics of Belt‐Shaped Cyclonaphthylenes,” Proceedings of the National Academy of Sciences 113 (2016): 8109–8114, 10.1073/pnas.1606530113. PMC4961134 27357686

[27] P. Li , B. M. Wong , L. N. Zakharov , and R. Jasti , “Investigating the Reactivity of 1,4‐Anthracene‐Incorporated Cycloparaphenylene,” Organic Letters 18 (2016): 1574–1577, 10.1021/acs.orglett.6b00430. 27002794

[28] Z. A. Huang , C. Chen , X. D. Yang , et al., “Synthesis of Oligoparaphenylene‐Derived Nanohoops Employing an Anthracene Photodimerization–Cycloreversion Strategy,” Journal of the American Chemical Society 138 (2016): 11144–11147, 10.1021/jacs.6b07673. 27539737

[29] J. Wang , G. Zhuang , M. Chen , et al., “Selective Synthesis of Conjugated Chiral Macrocycles: Sidewall Segments of (−)/(+)‐(12,4) Carbon Nanotubes With Strong Circularly Polarized Luminescence,” Angewandte Chemie International Edition 59 (2020): 1619–1626, 10.1002/anie.201909401. 31710148

[30] T. Nishiuchi , Y. Makihara , and T. Kubo , “Strain‐Induced Quinoidal Character in a Carbon Nanoring Embedding Anthracene Units,” Journal of the American Chemical Society 147 (2025): 37488–37496, 10.1021/jacs.5c11812. 41054815 PMC12532303

[31] P. Della Sala , A. Capobianco , T. Caruso , et al., “An Anthracene‐Incorporated [8]Cycloparaphenylene Derivative as an Emitter in Photon Upconversion,” Journal of Organic Chemistry 83 (2018): 220–227, 10.1021/acs.joc.7b02590. 29231727

[32] Z. Sun , N. Miyamoto , S. Sato , H. Tokuyama , and H. Isobe , “An Obtuse‐angled Corner Unit for Fluctuating Carbon Nanohoops,” Chem—Asian Journal 12 (2016): 271–275. 10.1002/asia.201601614 27897398

[33] Y. Xu , S. Gsänger , M. B. Minameyer , et al., “Highly Strained, Radially π‐Conjugated Porphyrinylene Nanohoops,” Journal of the American Chemical Society 141 (2019): 18500–18507, 10.1021/jacs.9b08584. 31710474

[34] S. Kammermeier , P. G. Jones , and R. Herges , “Ring‐Expanding Metathesis of Tetradehydro‐Anthracene—Synthesis and Structure of a Tubelike, Fully Conjugated Hydrocarbon,” Angewandte Chemie International Edition in English 35 (1996): 2669–2671, 10.1002/anie.199626691.

[35] A. Doussot , M.‐F. Bakaï , E. Fouquet , and P. Hermange , “Ex Situ Generation of^18^ O_2_ and^17^ O_2_ From Endoperoxides for *O‐Labeling and Mechanistic Studies of Oxidations by Dioxygen,” Organic Letters 25 (2023): 4661–4665, 10.1021/acs.orglett.3c01487. 37276381

[36] J. Rigaudy , P. Scribe , and C. Brelière , “Transformations Photochimiques d'endoperoxydes Dérivés D'hydrocarbures Aromatiques Polycycliques—I,” Tetrahedron 37 (1981): 2585–2593, 10.1016/S0040-4020(01)98961-6.

[37] R. L. Donkers and M. S. Workentin , “Elucidation of the Electron Transfer Reduction Mechanism of Anthracene Endoperoxides,” Journal of the American Chemical Society 126 (2004): 1688–1698, 10.1021/ja035828a. 14871099

[38] D. Reinhard , F. Rominger , and M. Mastalerz , “Synthesis of Triphenylene‐Based Triptycenes via Suzuki–Miyaura Cross‐Coupling and Subsequent Scholl Reaction,” Journal of Organic Chemistry 80 (2015): 9342–9348, 10.1021/acs.joc.5b01520. 26315496

[39] G. Wittig , “TRIPTYCENE,” Organic Syntheses 39 (1959): 75.

[40] F. Bertani , N. Riboni , F. Bianchi , et al., “Triptycene‐Roofed Quinoxaline Cavitands for the Supramolecular Detection of BTEX in Air,” Chemistry—A European Journal 22 (2016): 3312–3319, 10.1002/chem.201504229. 26762207

[41] K. C. Bass , “9,10‐DIHYDROANTHRACENE,” Organic Syntheses 42 (1962): 48.

[42] R. Jasti , J. Bhattacharjee , J. B. Neaton , and C. R. Bertozzi , “Synthesis, Characterization, and Theory of [9]‐, [12]‐, and [18]Cycloparaphenylene: Carbon Nanohoop Structures,” Journal of the American Chemical Society 130 (2008): 17646–17647, 10.1021/ja807126u. 19055403 PMC2709987

[43] Y. Segawa , S. Miyamoto , H. Omachi , et al., “Concise Synthesis and Crystal Structure of [12]Cycloparaphenylene,” Angewandte Chemie International Edition 50 (2011): 3244–3248, 10.1002/anie.201007232. 21370367

[44] S. Hitosugi , S. Sato , T. Matsuno , T. Koretsune , R. Arita , and H. Isobe , “Pentagon‐Embedded Cycloarylenes With Cylindrical Shapes,” Angewandte Chemie International Edition 56 (2017): 9106–9110, 10.1002/anie.201704676. 28608471

[45] E. Kayahara , R. Qu , M. Kojima , T. Iwamoto , T. Suzuki , and S. Yamago , “Ligand‐Controlled Synthesis of [3]‐ and [4]Cyclo‐9,9‐Dimethyl‐2,7‐Fluorenes Through Triangle‐ and Square‐Shaped Platinum Intermediates,” Chemistry—A European Journal 21 (2015): 18939–18943, 10.1002/chem.201504369. 26541506

[46] H.‐W. Jiang , T. Tanaka , H. Mori , K. H. Park , D. Kim , and A. Osuka , “Cyclic 2,12‐Porphyrinylene Nanorings as a Porphyrin Analogue of Cycloparaphenylenes,” Journal of the American Chemical Society 137 (2015): 2219–2222, 10.1021/ja513102m. 25633052

[47] H. W. Jiang , T. Tanaka , T. Kim , et al., “Synthesis of [n]Cyclo‐5,15‐Porphyrinylene‐4,4′‐Biphenylenes Displaying Size‐Dependent Excitation‐Energy Hopping,” Angewandte Chemie International Edition 54 (2015): 15197–15201, 10.1002/anie.201507822. 26510641

[48] N. Narita , Y. Kurita , K. Osakada , T. Ide , H. Kawai , and Y. Tsuchido , “A Dodecamethoxy[6]Cycloparaphenylene Consisting Entirely of Hydroquinone Ethers: Unveiling In‐Plane Aromaticity Through a Rotaxane Structure,” Nature Communications 14 (2023): 8091, 10.1038/s41467-023-43907-7. PMC10703805 38062009

[49] Y. Tsuchido , R. Abe , T. Ide , and K. Osakada , “A Macrocyclic Gold(I)–Biphenylene Complex: Triangular Molecular Structure With Twisted Au_2_ (Diphosphine) Corners and Reductive Elimination of [6]Cycloparaphenylene,” Angewandte Chemie International Edition 59 (2020): 22928–22932, 10.1002/anie.202005482. 32692468

[50] Y. Segawa , H. Omachi , and K. Itami , “Theoretical Studies on the Structures and Strain Energies of Cycloparaphenylenes,” Organic Letters 12 (2010): 2262–2265, 10.1021/ol1006168. 20402525

[51] C. E. Colwell , T. W. Price , T. Stauch , and R. Jasti , “Strain Visualization for Strained Macrocycles,” Chemical Science 11 (2020): 3923–3930, 10.1039/D0SC00629G. 34122862 PMC8152662

[52] H. Terrones , M. Terrones , E. Hernández , N. Grobert , J.‐C. Charlier , and P. M. Ajayan , “New Metallic Allotropes of Planar and Tubular Carbon,” Physical Review Letters 84 (2000): 1716–1719, 10.1103/PhysRevLett.84.1716. 11017608

[53] Y. Han , S. Wu , K. Y. S. Khoo , and C. Chi , “Synthesis of Fully π‐Conjugated Non‐Alternant Carbon Nanobelts,” Nature Synthesis 4 (2025): 947–955, 10.1038/s44160-025-00797-5.

[54] K. Iida , K. Endo , Y. Li , K. Okabe , and M. Yabe , Polymer Compound, Reticulated Polymer Compound Produced by Crosslinking the Polymer Compound, Composition for Organic Electroluminescent Element, Organic Electroluminescent Element, Organic EL Display, and Organic EL Lighting , EP2272894 B1 (Mitsubishi Chemical Corp, 2009).

[55] K. Liu , R. A. Lalancette , and F. Jäkle , “B–N Lewis Pair Functionalization of Anthracene: Structural Dynamics, Optoelectronic Properties, and O_2_ Sensitization,” Journal of the American Chemical Society 139 (2017): 18170–18173, 10.1021/jacs.7b11062. 29185739

[56] M. P. Schuldt , F. Rominger , and M. Mastalerz , “Synthesis of Enantiopure [3]Cyclorubicenes,” Angewandte Chemie International Edition 65 (2025): e20880. 41208806 10.1002/anie.202520880 PMC12759246

[57] J. H. May , J. M. Fehr , J. C. Lorenz , L. N. Zakharov , and R. Jasti , “A High‐Yielding Active Template Click Reaction (AT−CuAAC) for the Synthesis of Mechanically Interlocked Nanohoops,” Angewandte Chemie International Edition 63 (2024): e202401823, 10.1002/anie.202401823. 38386798

[58] R. Jasti , B. P. Branchaud , B. White , T. Lovell , and C. Colwell , Nanohoop Compounds for Use in Biotechnology and Methods of Making and Using the Same , US20190025315A1 (University of Oregon, 2018).

[59] I. B. Berlman , “6‐GRAPHS,” in Handbook of Fluorescence Spectra of Aromatic Molecules (1971), 107–415.

[60] W. Stawski , J. M. Van Raden , C. W. Patrick , P. N. Horton , S. J. Coles , and H. L. Anderson , “Strained Porphyrin Tape–Cycloparaphenylene Hybrid Nanorings,” Organic Letters 25 (2023): 378–383, 10.1021/acs.orglett.2c04089. 36626241 PMC9872170

[61] A. Ciesielski , D. K. Stepień , M. A. Dobrowolski , Ł. U. Dobrzycki , and M. K. Cyrański , “On the Aromatic Stabilization of Benzenoid Hydrocarbons,” Chemical Communications 48 (2012): 10129, 10.1039/c2cc33974a. 22962659

[62] J. I. Wu , C. S. Wannere , Y. Mo , P. Schleyer , and U. H. Bunz , “4n π Electrons but Stable: N, N ‐Dihydrodiazapentacenes,” Journal of Organic Chemistry 74 (2009): 4343–4349, 10.1021/jo900684c. 19438180

[63] F. Edhborg , H. Bildirir , P. Bharmoria , K. Moth‐Poulsen , and B. Albinsson , “Intramolecular Triplet–Triplet Annihilation Photon Upconversion in Diffusionally Restricted Anthracene Polymer,” Journal of Physical Chemistry B 125 (2021): 6255–6263, 10.1021/acs.jpcb.1c02856. 34081465 PMC8279549

[64] C. Adamo and V. Barone , “Toward Reliable Density Functional Methods Without Adjustable Parameters: The PBE0 Model,” Journal of Chemical Physics 110 (1999): 6158–6170, 10.1063/1.478522.

[65] J. Autschbach , T. Ziegler , S. J. A. van Gisbergen , and E. J. Baerends , “Chiroptical Properties From Time‐dependent Density Functional Theory. I. Circular Dichroism Spectra of Organic Molecules,” Journal of Chemical Physics 116 (2002): 6930–6940, 10.1063/1.1436466.

[66] K. L. Bak , A. E. Hansen , K. Ruud , T. Helgaker , J. Olsen , and P. Jørgensen , “ *Ab initio* calculation of electronic circular dichroism fortrans‐cyclooctene using London atomic orbitals,” Theoretica Chimica Acta 90 (1995): 441–458.

[67] K. L. Bak , P. Jørgensen , T. Helgaker , K. Ruud , and H. J. R. A. Jensen , “Gauge‐origin Independent Multiconfigurational Self‐Consistent‐Field Theory for Vibrational Circular Dichroism,” Journal of Chemical Physics 98 (1993): 8873–8887, 10.1063/1.464445.

[68] V. Barone and M. Cossi , “Quantum Calculation of Molecular Energies and Energy Gradients in Solution by a Conductor Solvent Model,” Journal of Physical Chemistry A 102 (1998): 1995–2001, 10.1021/jp9716997.

[69] V. Barone , M. Cossi , and J. Tomasi , “A New Definition of Cavities for the Computation of Solvation Free Energies by the Polarizable Continuum Model,” Journal of Chemical Physics 107 (1997): 3210–3221, 10.1063/1.474671.

[70] V. Barone , M. Cossi , and J. Tomasi , “Geometry Optimization of Molecular Structures in Solution by the Polarizable Continuum Model,” Journal of Computational Chemistry 19 (1998): 404–417, 10.1002/(SICI)1096-987X(199803)19:4<404::AID-JCC3>3.0.CO;2-W.

[71] R. Bauernschmitt and R. Ahlrichs , “Treatment of Electronic Excitations Within the Adiabatic Approximation of Time Dependent Density Functional Theory,” Chemical Physics Letters 256 (1996): 454–464, 10.1016/0009-2614(96)00440-X.

[72] A. D. Becke , “Density‐functional Thermochemistry. III. The Role of Exact Exchange,” Journal of Chemical Physics 98 (1993): 5648–5652, 10.1063/1.464913.

[73] J.‐P. Blaudeau , M. P. McGrath , L. A. Curtiss , and L. Radom , “Extension of Gaussian‐2 (G2) Theory to Molecules Containing Third‐row Atoms K and Ca,” Journal of Chemical Physics 107 (1997): 5016–5021, 10.1063/1.474865.

[74] R. Cammi , “Quantum Cluster Theory for the Polarizable Continuum Model. I. The CCSD Level With Analytical First and Second Derivatives,” Journal of Chemical Physics 131 (2009): 164104, 10.1063/1.3245400. 19894924

[75] R. Cammi , “Coupled‐Cluster Theories for the Polarizable Continuum Model. II. Analytical Gradients for Excited States of Molecular Solutes by the Equation of Motion Coupled‐Cluster Method,” International Journal of Quantum Chemistry 110 (2010): 3040–3052, 10.1002/qua.22884.

[76] R. Cammi , B. Mennucci , and J. Tomasi , “Second‐Order Møller−Plesset Analytical Derivatives for the Polarizable Continuum Model Using the Relaxed Density Approach,” Journal of Physical Chemistry A 103 (1999): 9100–9108, 10.1021/jp991564w.

[77] R. Cammi , B. Mennucci , and J. Tomasi , “Fast Evaluation of Geometries and Properties of Excited Molecules in Solution: a Tamm‐Dancoff Model With Application to 4‐Dimethylaminobenzonitrile,” Journal of Physical Chemistry A 104 (2000): 5631–5637, 10.1021/jp000156l.

[78] E. Cancès , B. Mennucci , and J. Tomasi , “A new integral equation formalism for the polarizable continuum model: Theoretical background and applications to isotropic and anisotropic dielectrics,” Journal of Chemical Physics 107 (1997): 3032–3041.

[79] M. Caricato , “Absorption and Emission Spectra of Solvated Molecules With the EOM–CCSD–PCM Method,” Journal of Chemical Theory and Computation 8 (2012): 4494–4502, 10.1021/ct3006997. 26605609

[80] M. E. Casida , C. Jamorski , K. C. Casida , and D. R. Salahub , “Molecular Excitation Energies to High‐lying Bound States From Time‐dependent Density‐functional Response Theory: Characterization and Correction of the Time‐dependent Local Density Approximation Ionization Threshold,” Journal of Chemical Physics 108 (1998): 4439–4449, 10.1063/1.475855.

[81] M. Cossi and V. Barone , “Solvent Effect on Vertical Electronic Transitions by the Polarizable Continuum Model,” Journal of Chemical Physics 112 (2000): 2427–2435, 10.1063/1.480808.

[82] M. Cossi and V. Barone , “Time‐dependent Density Functional Theory for Molecules in Liquid Solutions,” Journal of Chemical Physics 115 (2001): 4708–4717, 10.1063/1.1394921.

[83] M. Cossi , V. Barone , R. Cammi , and J. Tomasi , “Ab Initio Study of Solvated Molecules: A New Implementation of the Polarizable Continuum Model,” Chemical Physics Letters 255 (1996): 327–335, 10.1016/0009-2614(96)00349-1.

[84] M. Cossi , V. Barone , B. Mennucci , and J. Tomasi , “Ab Initio Study of Ionic Solutions by a Polarizable Continuum Dielectric Model,” Chemical Physics Letters 286 (1998): 253–260, 10.1016/S0009-2614(98)00106-7.

[85] M. Cossi , V. Barone , and M. A. Robb , “A Direct Procedure for the Evaluation of Solvent Effects in MC‐SCF Calculations,” Journal of Chemical Physics 111 (1999): 5295–5302, 10.1063/1.479788.

[86] M. Cossi , N. Rega , G. Scalmani , and V. Barone , “Polarizable Dielectric Model of Solvation With Inclusion of Charge Penetration Effects,” Journal of Chemical Physics 114 (2001): 5691–5701, 10.1063/1.1354187.

[87] M. Cossi , N. Rega , G. Scalmani , and V. Barone , “Energies, Structures, and Electronic Properties of Molecules in Solution With the C‐PCM Solvation Model,” Journal of Computational Chemistry 24 (2003): 669–681, 10.1002/jcc.10189. 12666158

[88] M. Cossi , G. Scalmani , N. Rega , and V. Barone , “New Developments in the Polarizable Continuum Model for Quantum Mechanical and Classical Calculations on Molecules in Solution,” Journal of Chemical Physics 117 (2002): 43–54, 10.1063/1.1480445.

[89] L. A. Curtiss , M. P. McGrath , J.‐P. Blaudeau , N. E. Davis , R. C. Binning , and L. Radom , “Extension of Gaussian‐2 Theory to Molecules Containing Third‐row Atoms Ga–Kr,” Journal of Chemical Physics 103 (1995): 6104–6113, 10.1063/1.470438.

[90] M. M. Francl , W. J. Pietro , W. J. Hehre , et al., “Self‐consistent Molecular Orbital Methods. XXIII. A Polarization‐type Basis Set for Second‐row Elements,” Journal of Chemical Physics 77 (1982): 3654–3665, 10.1063/1.444267.

[91] M. J. Frisch , G. W. Trucks , H. B. Schlegel , et al., Wallingford, CT, 2016.

[92] G. R. Fulmer , A. J. M. Miller , N. H. Sherden , et al., “NMR Chemical Shifts of Trace Impurities: Common Laboratory Solvents, Organics, and Gases in Deuterated Solvents Relevant to the Organometallic Chemist,” Organometallics 29 (2010): 2176–2179, 10.1021/om100106e.

[93] F. Furche and R. Ahlrichs , “Adiabatic Time‐dependent Density Functional Methods for Excited state Properties,” Journal of Chemical Physics 117 (2002): 7433–7447, 10.1063/1.1508368.

[94] M. N. Glukhovtsev , A. Pross , M. P. McGrath , and L. Radom , “Extension of Gaussian‐2 (G2) Theory to Bromine‐ and Iodine‐containing Molecules: Use of Effective Core Potentials,” Journal of Chemical Physics 103 (1995): 1878–1885, 10.1063/1.469712.

[95] S. Grimme , S. Ehrlich , and L. Goerigk , “Effect of the Damping Function in Dispersion Corrected Density Functional Theory,” Journal of Computational Chemistry 32 (2011): 1456–1465, 10.1002/jcc.21759. 21370243

[96] A. E. Hansen and K. L. Bak , “ *Ab‐initio* Calculations of Electronic Circular Dichroism,” Enantiomer (1999): 455–476.

[97] T. Helgaker and P. Jørgensen , “An Electronic Hamiltonian for Origin Independent Calculations of Magnetic Properties,” Journal of Chemical Physics 95 (1991): 2595–2601, 10.1063/1.460912.

[98] P. Hohenberg and W. Kohn , “Inhomogeneous Electron Gas,” Physical Review 136 (1964): B864–B871, 10.1103/PhysRev.136.B864.

[99] W. Koch , A Chemist's Guide to Density Functional Theory, 2nd ed. (Wiley‐VCH, 2001), 10.1002/3527600043.

[100] W. Kohn and L. J. Sham , “Self‐Consistent Equations Including Exchange and Correlation Effects,” Physical Review 140 (1965): A1133–A1138, 10.1103/PhysRev.140.A1133.

[101] L. Krause , R. Herbst‐Irmer , G. M. Sheldrick , and D. Stalke , “Comparison of Silver and Molybdenum Microfocus X‐ray Sources for Single‐crystal Structure Determination,” Journal of Applied Crystallography 48 (2015): 3–10, 10.1107/S1600576714022985. 26089746 PMC4453166

[102] R. Krishnan , J. S. Binkley , R. Seeger , and J. A. Pople , “Self‐consistent Molecular Orbital Methods. XX. A Basis Set for Correlated Wave Functions,” Journal of Chemical Physics 72 (1980): 650–654, 10.1063/1.438955.

[103] C. Lee , W. Yang , and R. G. Parr , “Development of the Colle‐Salvetti Correlation‐energy Formula Into a Functional of the Electron Density,” Physical Review B, Condensed Matter and Materials Physics 37 (1988): 785–789, 10.1103/PhysRevB.37.785. 9944570

[104] F. Lipparini , G. Scalmani , B. Mennucci , E. Cancès , M. Caricato , and M. J. Frisch , “A Variational Formulation of the Polarizable Continuum Model,” Journal of Chemical Physics 133 (2010): 014106, 10.1063/1.3454683. 20614958

[105] A. D. McLean and G. S. Chandler , “Contracted Gaussian Basis Sets for Molecular Calculations. I. Second Row Atoms, Z=11–18,” Journal of Chemical Physics 72 (1980): 5639–5648, 10.1063/1.438980.

[106] B. Mennucci , E. Cancès , and J. Tomasi , “Evaluation of Solvent Effects in Isotropic and Anisotropic Dielectrics and in Ionic Solutions With a Unified Integral Equation Method: Theoretical Bases, Computational Implementation, and Numerical Applications,” Journal of Physical Chemistry B 101 (1997): 10506–10517, 10.1021/jp971959k.

[107] B. Mennucci and J. Tomasi , “Continuum Solvation Models: A New Approach to the Problem of Solute's Charge Distribution and Cavity Boundaries,” Journal of Chemical Physics 106 (1997): 5151–5158, 10.1063/1.473558.

[108] S. Miertuš , E. Scrocco , and J. Tomasi , “Electrostatic Interaction of a Solute With a Continuum. A Direct Utilizaion of AB Initio Molecular Potentials for the Prevision of Solvent Effects,” Chemical Physics 55 (1981): 117–129, 10.1016/0301-0104(81)85090-2.

[109] S. Miertus̃ and J. Tomasi , “Approximate Evaluations of the Electrostatic Free Energy and Internal Energy Changes in Solution Processes,” Chemical Physics 65 (1982): 239–245, 10.1016/0301-0104(82)85072-6.

[110] N. M. O'Boyle , A. L. Tenderholt , and K. M. Langner , “cclib: A Library for Package‐Independent Computational Chemistry Algorithms,” Journal of Computational Chemistry 29 (2008): 839–845, 10.1002/jcc.20823. 17849392

[111] J. Olsen , K. L. Bak , K. Ruud , T. Helgaker , and P. Jørgensen , “Orbital Connections for Perturbation‐dependent Basis Sets,” Theoretica Chimica Acta 90 (1995): 421–439, 10.1007/BF01113545.

[112] R. G. Parr and W. Yang , Density‐functional Theory of Atoms and Molecules, 1st ed. (Oxford Univ. Press, 1994).

[113] J. L. Pascual‐Ahuir , E. Silla , and I. Tuñon , “GEPOL: An Improved Description of Molecular Surfaces. III. A New Algorithm for the Computation of a Solvent‐Excluding Surface,” Journal of Computational Chemistry 15 (1994): 1127–1138, 10.1002/jcc.540151009.

[114] G. Scalmani and M. J. Frisch , “Continuous Surface Charge Polarizable Continuum Models of Solvation. I. General Formalism,” Journal of Chemical Physics 132 (2010): 114110, 10.1063/1.3359469. 20331284

[115] G. Scalmani , M. J. Frisch , B. Mennucci , J. Tomasi , R. Cammi , and V. Barone , “Geometries and Properties of Excited States in the Gas Phase and in Solution: Theory and Application of a Time‐dependent Density Functional Theory Polarizable Continuum Model,” Journal of Chemical Physics 124 (2006): 94107, 10.1063/1.2173258. 16526845

[116] G. M. Sheldrick , “SHELXT—Integrated Space‐group and Crystal‐structure Determination,” Acta Crystallographica Section A, Foundations and Advances 71 (2015): 3–8, 10.1107/S2053273314026370. 25537383 PMC4283466

[117] G. M. Sheldrick , “Crystal Structure Refinement With SHELXL,” Acta Crystallographica Section C, Structural Chemistry 71 (2015): 3–8, 10.1107/S2053229614024218. 25567568 PMC4294323

[118] P. J. Stephens , F. J. Devlin , C. F. Chabalowski , and M. J. Frisch , “Ab Initio Calculation of Vibrational Absorption and Circular Dichroism Spectra Using Density Functional Force Fields,” Journal of Physical Chemistry 98 (1994): 11623–11627, 10.1021/j100096a001.

[119] J. J. Stewart , “Optimization of Parameters for Semiempirical Methods V: Modification of NDDO Approximations and Application to 70 Elements,” Journal of Molecular Modeling 13 (2007): 1173–1213, 10.1007/s00894-007-0233-4. 17828561 PMC2039871

[120] R. E. Stratmann , G. E. Scuseria , and M. J. Frisch , “An Efficient Implementation of Time‐dependent Density‐functional Theory for the Calculation of Excitation Energies of Large Molecules,” Journal of Chemical Physics 109 (1998): 8218–8224, 10.1063/1.477483.

[121] J. Tomasi , B. Mennucci , and E. Cancès , “The IEF Version of the PCM Solvation Method: An Overview of a New Method Addressed to Study Molecular Solutes at the QM Ab Initio Level,” Journal of Molecular Structure: THEOCHEM 464 (1999): 211–226, 10.1016/S0166-1280(98)00553-3.

[122] C. van Caillie and R. D. Amos , “Geometric derivatives of excitation energies using SCF and DFT,” Chemical Physics Letters 308 (1999): 249–255.

[123] S. H. Vosko , L. Wilk , and M. Nusair , “Accurate Spin‐dependent Electron Liquid Correlation Energies for Local Spin Density Calculations: A Critical Analysis,” Canadian Journal of Physics 58 (1980): 1200–1211, 10.1139/p80-159.

[124] M. J. Turner , J. J. McKinnon , D. Jayatilaka , and M. A. Spackman , “Visualisation and Characterisation of Voids in Crystalline Materials,” CrystEngComm 13 (2011): 1804–1813, 10.1039/C0CE00683A.

[125] P. R. Spackman , M. J. Turner , J. J. McKinnon , et al., “CrystalExplorer: A Program for Hirshfeld Surface Analysis, Visualization and Quantitative Analysis of Molecular Crystals,” Journal of Applied Crystallography 54 (2021): 1006–1011, 10.1107/S1600576721002910. 34188619 PMC8202033

[126] H. Takaba , H. Omachi , Y. Yamamoto , J. Bouffard , and K. Itami , “Selective Synthesis of [12]Cycloparaphenylene,” Angewandte Chemie (International ed in English) 48 (2009): 6112–6116, 10.1002/anie.200902617. 19588479

[127] Deposition Numbers CCDC‐2516805 (for **2**), 2516806 (for **4**), 2516807 (for **[6.6]CAPP‐OTES**), 2516808 (for **[8.8]CAPP‐OTES**), 2516809 (for **[10.10]CAPP‐OTES**), 2516810 (for **[12.12]CAPP‐OTES**), 2516811 (for **[14.14]CAPP‐OTES**), 2516812 (for **[16.16]CAPP‐OTES**), 2516813 (for **[6.6]CAPP‐OH**), 2521404 (for **[6.6]CAPP‐OTES‐O_2_ **), contain the supplementary crystallographic data for this paper. These data are provided free of charge by the joint Cambridge Crystallographic Data Centre and Fachinformationszentrum Karlsruhe Access Structures service.

