# Supplementary material for: Toward Larger Cyclo‐9,10‐Anthryleneparaphenylenes
Source: Chemistry. 2026 Mar 27;32(22):e70878. doi: 10.1002/chem.70878 (PMC13250362; doi:10.1002/chem.70878)
Supplement: Supplementary file 1 — The authors have cited additional references within the Supporting Information [50, 54, 55, 64, 65, 66, 67, 68, 69, 70, 71, 72, 73, 74, 75, 76, 77, 78, 79, 80, 81, 82, 83, 84, 85, 86, 87, 88, 89, 90, 91, 92, 93, 94, 95, 96, 97, 98, 99, 100, 101, 102, 103, 104, 105, 106, 107, 108, 109, 110, 111, 112, 113, 114, 115, 116, 117, 118, 119, 120, 121, 122, 123, 124, 125, 126]. The supporting crystallographic data for this paper are provided free of charge by the joint Cambridge Crystallographic Data Centre and Fachinformationszentrum Karlsruhe [127]. [file CHEM-32-e70878-s001.pdf]

Towards Larger Cyclo-9,10-anthryleneparaphenylenes

Moritz P. Schuldt, Frank Rominger, Sven M. Elbert, Michael Mastalerz*

Organisch-Chemisches Institut
Ruprecht-Karls-Universität Heidelberg
Im Neuenheimer Feld 272, 69120 Heidelberg, Germany
E-mail: michael.mastalerz@oci.uni-heidelberg.de

-Supporting Information-

Table of Contents

1 General Remarks........ S3

2 Experimental procedures........ S6

3 Spectra........ S16

3.1 ^{1}H NMR and ^{13}C NMR spectra........ S16

3.2 2D NMR spectra........ S27

3.3 IR spectra........ S47

3.4 Mass spectra........ S53

3.5 UV/Vis and fluorescence spectra........ S61

3.6 PXRD........ S68

4 Crystallographic data........ S70

5 Computational details........ S81

5.1 Strain Calculations........ S81

5.2 TDDFT:........ S82

5.3 XYZ-Coordinate........ S85

6 References........ S104

1 General Remarks

Materials: All used reagents and solvents were purchased from Fisher Scientific, Sigma Aldrich, BLDPharm, Deutero, Eurisotop, Honeywell, Grüssing, TCI or VWR Chemicals and used without further purification, if not mentioned otherwise. The reactions were performed under standard conditions (25°C, 1013 mbar). Complete removal of solvents was achieved by applying high vacuum ($1\cdot10^{-3}$ mbar). 9,10-bis(4-bromophenyl)-9,10-dihydroanthracene-9,10-diol (**2**) and 9,10-Bis(4,4,5,5-tetramethyl-1,3,2-dioxaborolan-2-yl)anthracene (**3**) were prepared according to literature procedures.[S1-2]

Thin layer and flash column chromatography: Analytical thin layer chromatography was performed with POLYGRAM® SIL G/UV254 gel plates sold by Macherey-Nagel. Detection was accomplished using UV-light (254 nm). Flash column chromatography was performed using Silica gel 60 (40–63 µm / 230–400 mesh ASTM) purchased from Macherey-Nagel.

Nuclear magnetic resonance (NMR): All reported NMR spectra were recorded on a Bruker Avance III 400 (400 MHz) or a Bruker Avance III 600 (600 MHz) spectrometer. Chemical shifts (δ) are given in parts per million (ppm) and coupling constants in Hertz (Hz). All spectra were calibrated relative to traces of less-deuterated solvent ($CDCl_3$: 7.26 ppm / 77.16 ppm, THF-d_8: 3.58 ppm / 67.21 ppm).[S3] The following abbreviations were used for ^1H NMR spectra to indicate the signal multiplicity: s (singlet), d (doublet), t (triplet), q (quartet), m (multiplet). All ^{13}C NMR spectra were measured with ^1H-decoupling.

Mass spectrometry (MS): MS experiments were performed on a Bruker timsTOFfleX spectrometer (HR-MALDI-MS, HR-APCI-MS) or a Bruker Autoflex Speed (MALDI-MS). For MALDI-MS experiments DCTB (trans-2-[3-(4-tert-Butylphenyl)-2-methylpropenylidene)malononitrile) was used as matrix. All measurements were performed by the mass spectrometry division of the University of Heidelberg under the supervision of Dr. Jürgen H. Gross.

Infrared spectroscopy (IR): IR spectra were recorded on a ZnSe ATR crystal using a Bruker Tensor 27 or Thermo Scientific Nicolet iS5 spectrometer with iD7 ATR module. The following abbreviations were used to indicate the absorption intensity: vw (very weak), w (weak), m (medium), s (strong), vs (very strong).

Elemental analysis: Elemental analysis was performed in the Microanalytical laboratory of the University of Heidelberg with an Elementar vario Micro Cube Element Analyzer.

UV/vis and fluorescence spectroscopy: UV/vis spectra were recorded with a Jasco V-730 spectrometer and fluorescence spectra with a Jasco FP-8300 spectrometer. Fluorescence

quantum yields were determined with a Jasco FP-8500 spectrometer with a JASCO ILF-835 (100 mm) integrating sphere by averaging three measurements of solutions with OD < 0.1.

Melting points: The non-corrected melting points were determined with a Büchi Melting Point B-565.

X-ray crystal structure analysis: X-ray crystal diffractiograms were recorded with a Bruker APEX-II Quazar diffractometer using Mo-K_{α} radiation (λ = 0.71073 Å) or a STOE Stadivari diffractometer using Cu-K_{α} radiation (λ = 1.54178 Å). Intensities were corrected for Lorentz and polarization effects, an empirical scaling and absorption correction was applied using SADABS[S4] or X-Area LANA 2.8.4 (STOE, 2024) based on the Laue symmetry of the reciprocal space (μ, Tmin, Tmax). The structures were solved with SHELXT-2014[S5] or SHELXT-2018/2 and refined against F2 with a full-matrix least-squares algorithm using the SHELXL-2018/3 or the SHELXL-2019/2 software. [S6] All depicted crystal structures (except **[10.10]CAPP-OTES**) are shown as ORTEP plot with 50% ellipsoids.

Gel permeation chromatography (GPC): Preparative gel permeation chromatography was performed in dichloromethane (5 mL/min) with four PSS SDV 100 Å columns and one PSS SDV 500 Å column on a Nexera LC-40 recycling GPC consisting of a LC-40D pump, a SIL-40C autosampler, a DGU-403 degassing unit, a CTO-40C column oven and a FRC-10A fraction collector. Substances were detected by a SPD-M40A photo diode array detector.

Computational Details: All quantum chemical calculations were performed by employing the Gaussian16 program package.[S7] The theoretical approach is based on Kohn-Sham density functional methodologies[S8-11] using the B3LYP[S12-15] functional and GD3BJ dispersion correction.[S16] As basis set the triple-ζ-basis (6-311G(d))[S17-19] was used. The geometries were pre-optimized using the semi-empirical PM6 method.[S20] Ground states and transition states were confirmed by using frequency calculations to exhibit no or exactly one imaginary frequency. Prediction of excited state properties were performed using time-depended DFT[S21-32] using the B3LYP,[S12-15] CAM-B3LYP[S33] or PBE1PBE[S34] functional with GD3BJ dispersion correction[S16] or the ωB97X-D[S35] functional with its included dispersion correction as well as PCM[S36-60] solvation and convoluted with GaussSum.[S61] Strain energies were calculated by homodesmotic reactions of the corresponding cycle with phenylanthracene.

PXRD: PXRD diffractograms were recorded using a Rigaku SmartLab diffractometer (9 kW) containing a HyPix-3000 detector. For measurements CuKα radiation (λ =1.54059 Å) in combination with a Debye–Scherrer geometry was used. Samples were filled in glass mark tubes (diameter: 0.6 mm) and attached to a capillary spin stage (60 rpm rotation). Background measurements of empty tubes have been conducted prior to sample measurements and were subtracted from the corresponding measurements.

BET surface areas: Samples for Gas sorption measurements were activated by suspending the samples in *n*-pentane (for **[6.6]CAPP-OH**) or MeOH (for **[6.6]CAPP-OTES**) and decanting off the solvent. This process was repeated three times and the remaining solvents were removed at 50°C and high vacuum ($<10^{-3}$ mbar). The measurements were performed on a Quantachrome Autosorb IQ 3 gas sorption analyzer using nitrogen gas at 77 K and the resulting BET surface areas were calculated using the devices ASiQwin Software (version 5.2). Theoretical values were obtained by removing weakly bound solvent molecules from the corresponding single crystal X-ray structure and calculating the void surfaces (isosurface: 0.0003 e•au^{-3}) using CrystalExplorer (Version 21.5).[S62-63]

2 Experimental procedures

Synthesis of 9,10-bis(4-bromophenyl)-9,10-bis((triethylsilyl)oxy)-9,10-dihydroanthracene (4):

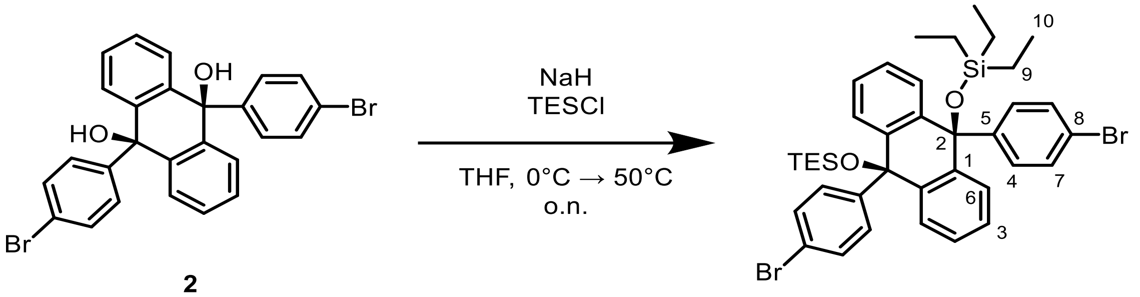

2 4

Sodium hydride (60% wt. in mineral oil, 766 mg, 19.2 mmol.) was suspended in tetrahydrofuran (20 mL) at 0°C. **2** (2.50 g, 4.79 mmol) was added and the solution was allowed to warm up to r.t. and stirred for 20 min and further 20 min at 50 °C. Triethylsilylchloride (4.80 mL, 28.7 mmol) was added and the reaction was stirred at 50 °C overnight. The mixture was cooled to r.t., water (10 mL) was added and the mixture was extracted with dichloromethane (3x50 mL). the organic phases were combined, dried over magnesium sulfate and the solvents were removed at the rotary evaporator. The resulting solid was sonicated with methanol (50 mL), filtered and dried *in vacuo* to obtain the silyl ether **4** as colorless solid (3.42 g, 95%). **M.p.:** 155 °C. **^{1}H NMR (600 MHz, $CDCl_3$):** δ = 7.59 (dd, J = 6.0, 3.4 Hz, 4H, H-6), 7.29 (dd, J = 6.0, 3.4 Hz, 4H, H-3), 7.20 (d, J = 8.7 Hz, 4H, H-7), 6.94 (d, J = 8.7 Hz, 4H, H-4), 0.87 (t, J = 8.0 Hz, 18H, H-10), 0.41 (q, J = 7.9 Hz, 12H, H-9) ppm. **^{13}C NMR (151 MHz, $CDCl_3$):** δ = 149.2 (C-5), 139.5 (C-1), 130.6 (C-7), 128.9 (C-4), 128.6 (C-6), 127.9 (C-3), 120.8 (C-8), 76.2 (C-2), 7.4 (C-10), 6.7 (C-9) ppm. **IR:** $\tilde{\nu}$ = 2952 (w), 2933 (w), 2906 (w), 2874 (w), 1481 (m), 1454 (w), 1444 (w), 1411 (w), 1393 (w), 1238 (m), 1179 (w), 1165 (w), 1139 (w), 1108 (w), 1071 (s), 1060 (s), 1005 (s), 971 (w), 940 (m), 922 (w), 904 (m), 845 (m), 833 (m), 813 (s), 756 (s), 726 (vs), 701 (m), 680 (m), 656 (m), 626 (w) cm^{-1}. **UV-Vis (dichloromethane):** λ_{max} (lg ε) = 268 (3.18) nm. **MS (HR-APCI):** *m/z* calculated for $[M\text{-}OTES]^+$: 617.0505, found 617.0506. **Elemental Analysis:** calculated for $C_{38}H_{46}Br_2O_2Si_2$: C 60.79%, H 6.18%, found: C 60.45%, H 6.44%.

[n.n]CAPP-OTES:

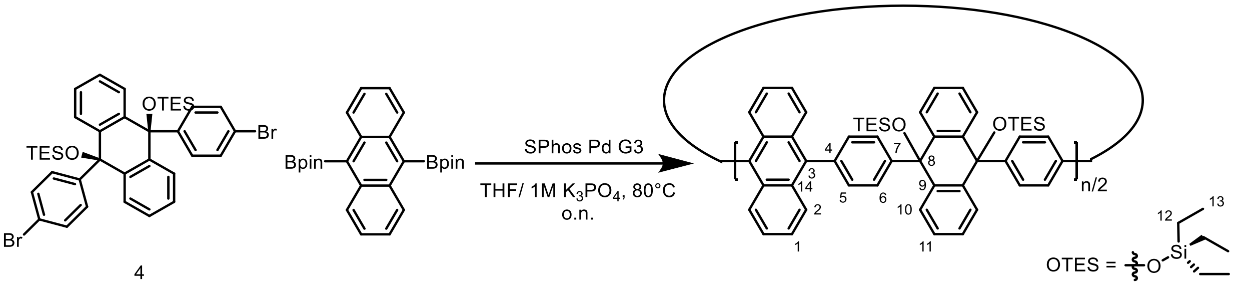

Silyl ether **4** (400 mg, 533 µmol), boronic ester **3** (229.2 mg, 533 µmol) and Sphos Pd G3 (41.6 mg, 53.3 µmol) were dissolved in tetrahydrofuran (32 mL) in a screw capped flask. The mixture was heated until it started to reflux, potassium phosphate solution (1 M, 16 mL) was added, the reaction vessel was closed and the mixture was stirred overnight at 80°C. The reaction mixture was cooled down to r.t., the phases were separated and the aqueous phase was extracted with dichloromethane (2 × 100 mL). The combined organic phases were dried over magnesium sulfate and filtered through a silica plug using dichloromethane. The solvents were removed *in vacuo* and the resulting solids were taken up in dichloromethane (2 mL). The different cycles were separated by gel-permeation-chromatography (dichloromethane, 5 mL/min, four SDV 100 Å columns and one SDV 500 Å column, see flow chart below) and dried *in vacuo* to obtain the **[n.n]CAPP-OTES** macrocycles as colorless solids:

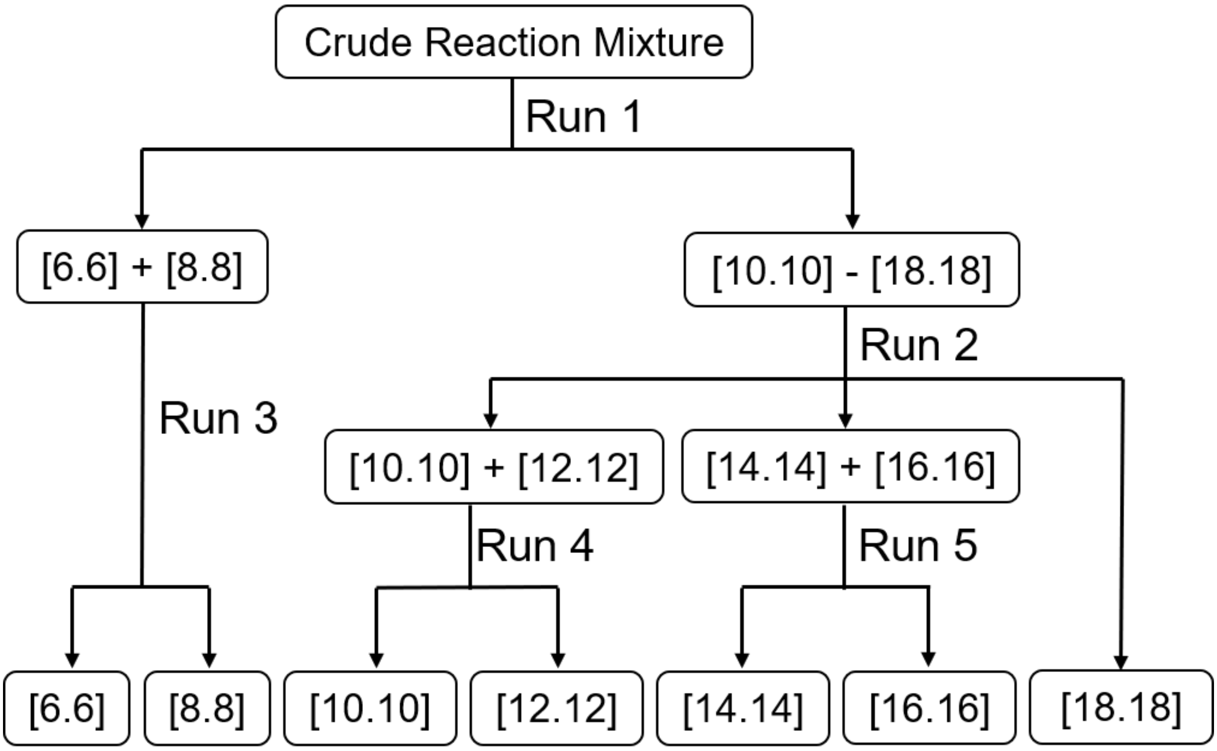

Figure S1: Flow chart of the GPC separation of **[6.6]CAPP-OTES** to **[18.18]CAPP-OTES**. See also Figure 3b) in the main article for GPC chromatograms of the various runs.

[6.6]CAPP-OTES (59.2 mg, 14%):

M.p.: >296 °C decomp. **^{1}H NMR (600 MHz, $CDCl_3$):** δ = 7.77 (dd, *J* = 6.0, 3.4 Hz, 4H, H-10), 7.57 (d, *J* = 8.5 Hz, 4H, H-6), 7.49 (dd, *J* = 6.8, 3.3 Hz, 4H, H-2), 7.40 (dd, *J* = 6.0, 3.4 Hz, 4H, H-11), 7.18 (d, *J* = 8.4 Hz, 4H, H-5), 7.11 (dd, *J* = 6.8, 3.3 Hz, 4H, H-1), 0.95 (t, *J* = 7.9 Hz, 36H, H-13), 0.50 (q, *J* = 8.0 Hz, 24H, H-12) ppm. **^{13}C NMR (151 MHz, $CDCl_3$):** δ = 150.5 (C-7), 140.0 (C-9), 137.1 (C-4), 136.8 (C-3), 130.7 (C-5), 129.8 (C-14), 129.6 (C-10), 127.0 (C-2), 126.7 (C-6), 124.9 (C-1), 76.4 (C-8), 7.5 (C-13), 6.7 (C-12) ppm. **IR (ATR):** $\tilde{\nu}$ = 2950 (w), 2934 (w), 2906 (w), 2873 (w), 1737 (w), 1657 (w), 1649 (w), 1631 (w), 1502 (w), 1478 (w), 1460 (w), 1444 (w), 1411 (w), 1391 (m), 1236 (m), 1183 (w), 1158 (w), 1138 (w), 1110 (w), 1081 (m), 1056 (m), 1006 (m), 972 (w), 942 (m), 923 (w), 905 (m), 890 (w), 846 (m), 816 (s), 794 (w), 768 (s), 758 (s), 723 (vs), 686 (m), 670 (s), 665 (m), 645 (m), 631 (m), 611 (m), 582 (w), 558 (w) cm^{-1}. **UV-Vis (dichloromethane):** λ_{max} (lg ε) = 261 (5.32), 340 (4.03), 358 (4.38), 376 (4.59), 396 (4.54) nm. **Fluorescence (dichloromethane, λ_{exc} = 253 nm):** λ_{max} = 419, 436 nm. ***Φ*** = 54±10%. **MS (HR-MALDI):** *m/z* calculated for $[M]^+$: 2299.0982, found 2299.1011. **Elemental Analysis:** calculated for $C_{156}H_{162}O_6Si_6$: C 81.41%, H 7.10%, found: C 81.08%, H 6.98%.

[8.8]CAPP-OTES (34.6 mg, 8.5%):

M.p.: >185 °C decomp. **^{1}H NMR (600 MHz, $CDCl_3$):** δ = 7.99 (dd, *J* = 6.0, 3.4 Hz, 4H, H-10), 7.55 (dd, *J* = 6.0, 3.4 Hz, 4H, H-11), 7.22 (dd, *J* = 6.8, 3.3 Hz, 4H, H-2), 7.15 (d, *J* = 8.4 Hz, 4H, H-6), 6.98 (d, *J* = 8.5 Hz, 4H, H-5), 6.43 (dd, *J* = 6.9, 3.3 Hz, 4H, H-1), 0.96 (t, *J* = 7.9 Hz, 18H, ,H-13), 0.56 (q, *J* = 7.9 Hz, 12H, H-12) ppm.**^{13}C NMR (151 MHz, $CDCl_3$):** δ = 147.2 (C-7), 140.8 (C-9), 137.4 (C-4), 136.3 (C-3), 130.5 (C-5), 129.4 (C-14), 128.2 (C-10), 127.7 (C-6), 127.6 (C-11), 126.6 (C-2), 124.4 (C-1), 77.5 (C-8), 7.5 (C-13), 6.9 (C-12) ppm. **IR (ATR):** $\tilde{\nu}$ = 2950 (m), 2933 (w), 2908 (w), 2873 (m), 1507 (w), 1457 (w), 1446 (w), 1412 (w), 1391 (w), 1237 (m), 1159 (w), 1139 (w), 1109 (w), 1053 (m), 1003 (s), 972 (w), 942 (m), 921 (w), 905 (m), 890 (m), 848 (m), 817 (m), 759 (s), 730 (vs), 682 (m), 668 (s), 666 (s), 647 (m), 632 (m), 611 (m), 581 (w), 560 (w) cm^{-1}. **UV-Vis (dichloromethane):** λ_{max} (lg ε) = 265 (5.43), 340 (4.16), 358 (4.49), 377 (4.67), 397 (4.60) nm. **Fluorescence (dichloromethane, λ_{exc} = 253 nm):** λ_{max} = 424, 439 nm. ***Φ*** = 33±8%.**MS (HR-MALDI):** *m/z* calculated for $[M]^+$: 3065.4644, found 3065.4670. **Elemental Analysis:** calculated for $C_{208}H_{216}O_8Si_8$: C 81.41%, H 7.10%, found: C 81.12%, H 6.94%.

[10.10]CAPP-OTES (14.2 mg, 3.5%):

M.p.: 170 °C decomp. **^{1}H NMR (600 MHz, $CDCl_3$):** *δ* = 7.86 (dd, *J* = 6.0, 3.4, 4H, H-10), 7.47 (dd, *J* = 6.0, 3.4, 4H, H-11), 7.36–7.32 (m, 8H, H-2+H-6), 7.09 (d, *J* = 8.2, 4H, H-5), 6.69 (dd, *J* = 6.8, 3.3, 4H, H-1), 0.96 (t, *J* = 8.0, 18H, H-13), 0.55 (q, *J* = 7.9, 12H, H-12) ppm. **^{13}C NMR (151 MHz, $CDCl_3$):** *δ* = 148.3 (C-3), 140.5 (C-9), 137.3 (C-4), 136.5 (C-7), 130.6 (C-5), 129.6 (C-14), 128.9 (C-10), 127.7 (C-11), 127.4 (C-2), 126.7 (C-6), 124.5 (C-1), 76.9 (C-8)*, 7.2 (C-13), 6.0 (C-12) ppm. **IR (ATR):** ṽ = 3063 (w), 2952 (w), 2909 (w), 2874 (m), 1558 (w), 1519 (w), 1506 (w), 1456 (w), 1446 (w), 1414 (w), 1393 (w), 1238 (m), 1197 (w), 1185 (w), 1162 (w), 1139 (w), 1111 (w), 1075 (m), 1058 (m), 1004 (m), 972 (w), 942 (m), 906 (m), 891 (m), 847 (m), 817 (m), 793 (w), 760 (s), 728 (vs), 681 (s), 669 (s), 667 (s), 647 (m), 632 (m), 611 (m), 584 (w) cm^{-1}. **UV-Vis (dichloromethane):** λ_{max} (lg *ε*) = 263 (5.50), 341 (4.24), 359 (4.57), 378 (4.77), 398 (4.71) nm. **Fluorescence (dichloromethane, λ_{exc} = 253 nm):** λ_{max} = 424, 439 nm. ***Φ*** = 30±9%. **MS (HR-MALDI):** *m/z* calculated for $[M]^+$: 3831.8306, found 3831.8354.

*This signals chemical shift was assigned by ^{1}H,^{13}C HMBC, as it coincides with the one from $CDCl_3$.

[12.12]CAPP-OTES (7.5 mg, 1.8%):

M.p.: 209 °C decomp. **^{1}H NMR (600 MHz, $CDCl_3$):** *δ* = 7.87 (dd, *J* = 6.0, 3.5, 4H, H-10), 7.42 (dd, *J* = 6.1, 3.4, 4H, H-11), 7.21 (d, *J* = 7.8, 4H, H-6), 7.19-7.16 (m, 4H, H-2), 6.90 (d, *J* = 7.9, 4H, H-5), 6.41 (br, 4H, H-1), 0.96 (t, *J* = 7.9, 18H, H-13), 0.54 (q, *J* = 7.9, 12H, H-12) ppm. **^{13}C NMR (151 MHz, $CDCl_3$):** *δ* = 148.2 (C-7), 140.4 (C-9), 137.2 (C-4), 136.3 (C-3), 130.6 (C-5), 129.4 (C-14), 128.8 (C-10), 127.7 (C-11), 127.3 (C-6), 126.5 (C-2), 124.5 (C-1),77.2 (C-8)*, 7.5 (C-13), 6.9 (C-12) ppm. **IR (ATR):** ṽ = 3063 (vw), 2951 (w), 2909 (w), 2873 (w), 1506 (vw), 1457 (w), 1446 (w), 1413 (w), 1391 (w), 1263 (vw), 1237 (w), 1195 (w), 1185 (w), 1165 (w), 1139 (w), 1077 (m), 1054 (m), 1003 (m), 969 (w), 942 (m), 921 (w), 905 (m), 890 (m), 847 (m), 817 (m), 792 (w), 759 (s), 730 (vs), 682 (m), 666 (s), 646 (m), 632 (m), 611 (m), 582 (w), 556 (w) cm^{-1}. **UV-Vis (dichloromethane):** λ_{max} (lg *ε*) = 264 (5.65), 343 (4.42), 360 (4.73), 379 (4.92), 400 (4.85) nm. **Fluorescence (dichloromethane, λ_{exc} = 253 nm):** λ_{max} = 424, 439 nm. ***Φ*** = 45±9%. **MS (HR-MALDI):** *m/z* calculated for $[M]^+$: 4598.1969, found 4598.2000.

*This signals chemical shift was assigned by ^{1}H,^{13}C HMBC, as it coincides with the $CDCl_3$.

[14.14]CAPP-OTES (6.0 mg, 1.5%):

M.p.: >209 °C decomp. **^{1}H NMR (600 MHz, $CDCl_3$):** δ = 7.80 (dd, *J* = 6.0, 3.4, 4H, H-10), 7.38 (dd, *J* = 6.0, 3.4, 4H,H-11), 7.31 (d, *J* = 8.0, 4H, H-6), 7.29 - 7.26 (d, *J* = 3.2, 4H, H-2), 6.99 (d, *J* = 8.2, 4H, H-5), 6.62 (dd, *J* = 6.8, 3.4, 4H, H-1), 0.93 (t, *J* = 7.9, 18H, H-13), 0.51 (q, *J* = 8.0, 12H, H-12) ppm.**^{13}C NMR (151 MHz, $CDCl_3$):** δ = 148.7 (C-7), 140.3 (C-9), 137.2 (C-4), 136.5 (C-3), 130.6 (C-5), 129.5 (C-14), 129.0 (C-10), 127.7 (C-11), 127.2 (C-6), 126.6 (C-2), 124.6 (C-1), 76.9 (C-8)*, 7.5 (C-13), 6.8 (C-12) ppm. **IR (ATR):** ṽ =2950 (w), 2909 (w), 2873 (w), 1503 (w), 1457 (w), 1445 (w), 1412 (w), 1391 (w), 1238 (m), 1185 (w), 1165 (w), 1139 (w), 1077 (m), 1057 (m), 1004 (m), 941 (m), 906 (m), 890 (w), 847 (m), 817 (m), 759 (s), 730 (vs), 684 (m), 668 (s), 666 (s), 646 (m), 632 (m), 611 (s), 585 (m), 558 (m) cm^{-1}. **UV-Vis (dichloromethane):** λ_{max} (lg ε) = 263 (5.79), 342 (4.55), 359 (4.87), 378 (5.06), 399 (5.00) nm. **Fluorescence (dichloromethane, λ_{exc} = 253 nm):** λ_{max} = 424, 439 nm. ***Φ*** = 38±9%. **MS (HR-MALDI):** *m/z* calculated for $[M]^+$: 5364.5631, found 5364.5703.

*This signals chemical shift was assigned by ^{1}H,^{13}C HMBC, as it coincides with the one from $CDCl_3$.

[16.16]CAPP-OTES (5.0 mg, 1.2%):

M.p.: >230 °C decomp. **^{1}H NMR (600 MHz, $CDCl_3$):** δ = 7.86 (dd, *J* = 6.0, 3.4, 4H, H-10), 7.41 (dd, *J* = 6.0, 3.4, 4H, H-11), 7.26-7.23 (m, 8H, H-2+H-6), 6.97 (d, *J* = 8.1, 4H, H-5), 6.55 (dd, *J* = 7.0, 3.4, 4H, H-1), 0.93 (t, *J* = 7.9, 18H, H-13), 0.52 (q, *J* = 7.9, 12H, H-12) ppm. **^{13}C NMR (151 MHz, $CDCl_3$):** δ = 148.3 (C-7), 140.5 (C-9), 137.3 (C-4), 136.4 (C-3), 130.6 (C-5), 129.5 (C-14), 128.7 (C-10), 127.7 (C-11), 127.3 (C-6), 126.6 (C-2), 124.5 (C-1), 77.1 (C-8)*, 7.5 (C-13), 6.9 (C-12) ppm. **IR (ATR):** ṽ = 2951 (m), 2934 (w), 2909 (w), 2874 (w), 1740 (w), 1459 (w), 1445 (w), 1391 (w), 1238 (m), 1184 (w), 1166 (w), 1139 (m), 1111 (w), 1083 (m), 1060 (m), 1006 (m), 942 (m), 922 (w), 906 (m), 891 (m), 848 (m), 818 (m), 793 (w), 759 (s), 734 (vs), 685 (m), 670 (m), 668 (m), 666 (m), 647 (w), 633 (m), 611 (m), 585 (w), 561 (w) cm^{-1}. **UV-Vis (dichloromethane):** λ_{max} (lg ε) = 263 (5.75), 342 (4.50), 360 (4.82), 378 (5.01), 399 (4.94) nm. **Fluorescence (dichloromethane, λ_{exc} = 253 nm):** λ_{max} = 424, 441 nm. ***Φ*** = 27±8%. **MS (HR-MALDI):** *m/z* calculated for $[M]^+$: 6131.9371, found 6131.9402.

*This signals chemical shift was assigned by ^{1}H,^{13}C HMBC, as it coincides with the one from $CDCl_3$.

[18.18]CAPP-OTES (4.3 mg, 1.1%):

M.p.: >288 °C decomp. **^{1}H NMR (600 MHz, $CDCl_3$):** δ = 7.83 (dd, J = 6.0, 3.4 Hz, 4H, H-10), 7.39 (dd, J = 6.0, 3.4 Hz, 4H, H-11), 7.32-7.27 (m, 8H, H-2+H-6), 7.01 (d, J = 8.0 Hz, 4H, H-5), 6.63 (dd, J = 6.8, 3.3 Hz, 4H, H-1), 0.92 (t, J = 7.9 Hz, 18H, H-13), 0.50 (q, J = 7.9 Hz, 12H, H-12) ppm. **^{13}C NMR (151 MHz, $CDCl_3$):** δ = 148.4 (C-7), 140.2 (C-9), 137.1 (C-4), 136.3 (C-3), 130.4 (C-5), 129.3 (C-14), 128.7 (C-2), 127.5 (C-1), 127.0 (C-6), 126.4 (C-10), 124.4 (C-11), 76.6 (C-8)*, 7.3 (C-13), 6.6 (C-12) ppm. **IR (ATR):** $\tilde{\nu}$ = 2950 (w), 2934 (w), 2909 (w), 2874 (w), 1456 (w), 1446 (w), 1413 (w), 1393 (w), 1237 (w), 1139 (w), 1054 (m), 1004 (m), 973 (w), 942 (m), 906 (m), 891 (w), 847 (m), 817 (m), 759 (s), 731 (vs), 720 (vs), 684 (s), 669 (s), 667 (s), 647 (m), 632 (m), 611 (m), 585 (w), 558 (w) cm^{-1}. **UV-Vis (dichloromethane):** λ_{max} (lg ε) = 263 (5.90), 342 (4.63), 360 (4.96), 378 (5.15), 399 (5.09) nm. **Fluorescence (dichloromethane, λ_{exc} = 253 nm):** λ_{max} = 424, 439 nm. **Φ** = 29±7%. **MS (HR-MALDI):** *m/z* calculated for $[M]^+$: 6899.3023, found 6899.3091.

*This signals chemical shift was assigned by ^{1}H,^{13}C HMBC, as it coincides with the one from $CDCl_3$.

[6.6]CAPP-OH via deprotection of [6.6]CAPP-OTES:

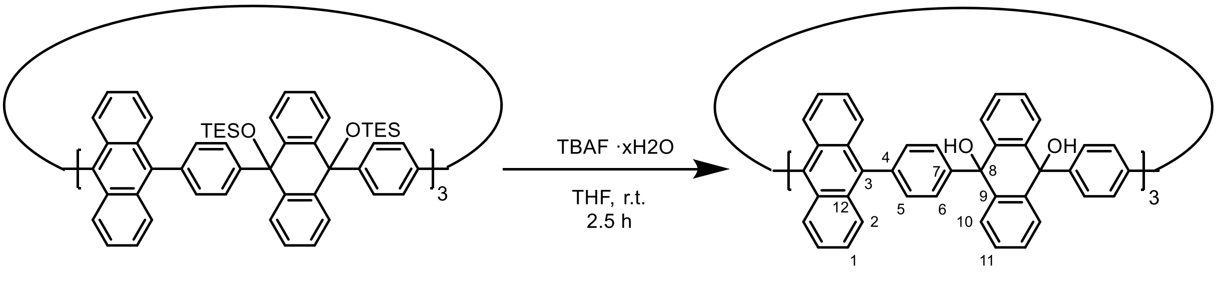

[6.6]CAPP-OTES (53.0 mg, 23.0 µmol) was dissolved in tetrahydrofuran (3 mL) and $[CH_3(CH_2)_3]_4NF\cdot xH_2O$ (67.7 mg, 214 µmol) was added. The resulting mixture was stirred at r.t. for 2.5 h. Water (15 mL) was added, the resulting precipitate was removed by filtration, washed with water (30 mL), methanol (20 mL) and dichloromethane (20 mL) and dried *in vacuo* to obtain the deprotected macrocycle **[6.6]CAPP-OH** as colorless solid (32.6 mg, 20.2 µmol) in 88% yield.

M.p.: >291 °C decomp. **^{1}H NMR (600 MHz, THF-d_8):** δ = 7.93 (dd, J = 6.0, 3.4 Hz, 4H, H-10), 7.65 (d, J = 8.3 Hz, 4H, H-6), 7.48 (dd, J = 6.8, 3.3 Hz, 4H, H-2), 7.38 (dd, J = 6.0, 3.4 Hz, 4H, H-11), 7.18 (d, J = 8.3 Hz, 4H, H-5), 7.04 (dd, J = 6.8, 3.3 Hz, 4H, H-1), 5.71 (s, 2H, OH) ppm. **^{13}C NMR (151 MHz, THF-d_8):** δ = 149.0 (C-7), 141.7 (C-9), 137.4 (C-4), 136.9 (C-3), 130.5 (C-5), 130.0 (C-12), 128.2 (C-10), 127.6 (C-11), 127.4 (C-6), 126.8 (C-2), 124.9 (C-1), 73.2 (C-8) ppm. **IR:** $\tilde{\nu}$ = 3545 (w), 3062 (w), 3029 (vw), 1508 (w), 1483 (w), 1475 (w), 1446 (w), 1393 (m),

1326 (w), 1261 (w), 1237 (w), 1188 (w), 1158 (w), 1135 (w), 1108 (w), 1018 (m), 1009 (m), 944 (m), 920 (w), 884 (m), 819 (s), 794 (w), 761 (vs), 743 (m), 719 (m), 671 (m), 645 (m), 631 (m), 612 (m), 584 (w), 552 (w) cm^{-1}. **UV-Vis (dichloromethane):** λ_{max} (lg ε) = 260 (5.31), 340 (3.98), 357 (4.36), 375 (4.57), 396 (4.52) nm. **Fluorescence (dichloromethane, λ_{exc} = 254 nm):** λ_{max} = 420, 437 nm. ***Φ*** = 45±10%. **MS (HR-MALDI):** *m/z* calculated for $[M]^+$: 1614.5793, found 1614.5824. **Elemental Analysis:** calculated for $C_{120}H_{78}O_6$·2.5THF: C 86.95%, H 5.47%, found: C 87.21%, H 5.20%.

[8.8]CAPP-OH via deprotection of [8.8]CAPP-OTES:

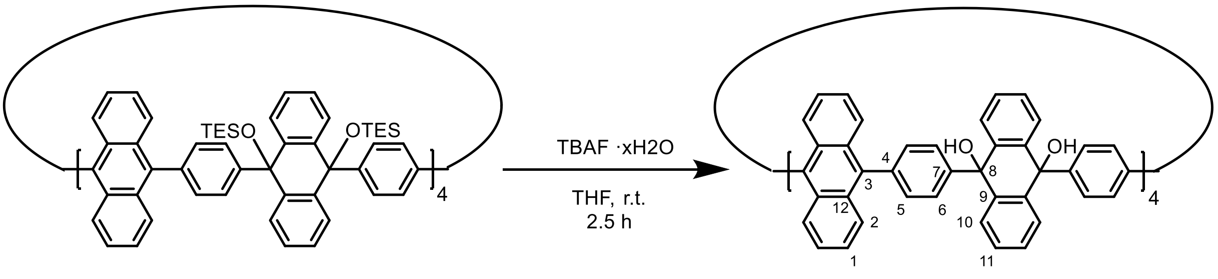

[8.8]CAPP-OTES (57.4 mg, 18.7 µmol) was dissolved in tetrahydrofuran (3 mL) and $[CH_3(CH_2)_3]_4NF\cdot xH_2O$ (73.2 mg, 232 µmol) was added. The resulting mixture was stirred at r.t. for 2.5 h. Water (15 mL) was added, the resulting precipitate was removed by filtration, washed with water (30 mL), methanol (20 mL) and dichloromethane (20 mL) and dried *in vacuo* to give the deprotected macrocycle **[8.8]CAPP-OH** as colorless solid (27.8 mg, 12.9 µmol) in 69% yield.

M.p.: >211 °C decomp. **^{1}H NMR (600 MHz, THF-d$_8$):** δ = 8.20 (dd, *J* = 5.9, 3.4 Hz, 4H, H-10), 7.54 (dd, *J* = 5.9, 3.4 Hz, 4H, H-11), 7.18 (d, *J* = 8.3 Hz, 4H, H-6), 7.12 (dd, *J* = 6.8, 3.3 Hz, 4H, H-2), 6.93 (d, *J* = 8.4 Hz, 4H, H-5), 6.26 (dd, *J* = 6.8, 3.3 Hz, 4H, H-1), 5.71 (s, 2H, OH) ppm. **^{13}C NMR (161 MHz, THF-d$_8$):*** δ = 146.8, 142.5, 137.2, 136.4, 129.9, 129.3, 128.1, 127.2, 126.6, 124.0, 73.9. **IR:** ṽ = 3514 (w), 3377 (w), 3063 (w), 3027 (vw), 1603 (w), 1507 (w), 1474 (w), 1446 (w), 1392 (m), 1318 (w), 1159 (m), 1132 (m), 1018 (m), 1009 (m), 943 (m), 919 (w), 884 (m), 820 (s), 759 (vs), 733 (s), 714 (m), 695 (m), 667 (s), 645 (m), 631 (s), 611 (s), 582 (m), 551 (m) cm^{-1}. **UV-Vis (dichloromethane):** λ_{max} (lg ε) = 265 (5.50), 340 (4.23), 358 (4.54), 376 (4.73), 397 (4.65) nm. **Fluorescence (dichloromethane, λ_{exc} = 254 nm):** λ_{max} = 421, 438 nm. ***Φ*** = 56±7%. **MS (HR-MALDI):** *m/z* calculated for $[M]^+$: 2152.7726, found 2152.7757. **Elemental Analysis:** calculated for $C_{160}H_{104}O_8\cdot 5\ H_2O$: C 85.61%, H 5.12%, found: C 85.87%, H 5.19%.

*Due to the low solubility of **[8.8]CAPP-OH** no ^{13}C NMR signals above 3σ could be detected (even at 176 MHz and 40960 scans). Therefore, the center of the ^{1}H,^{13}C HMBC signals are reported.

[6.6]CAPP-OH and [8.8]CAPP-OH via direct Suzuki Coupling:

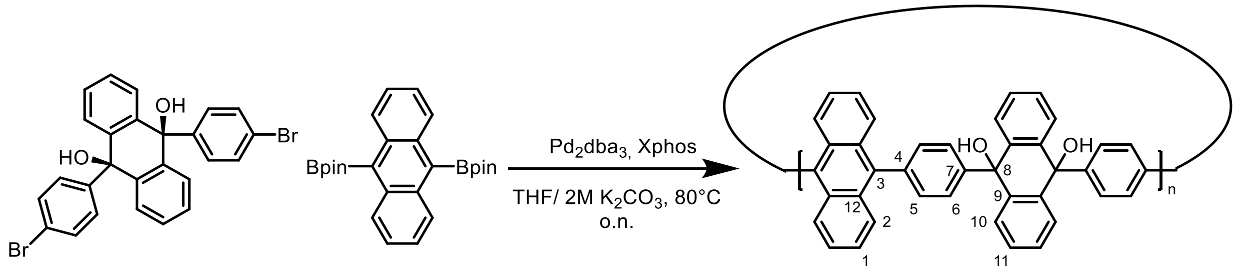

2 (100 mg, 191 µmol), **3** (82.4 mg, 191 µmol) and XPhos (27.4 mg, 57.5 µmol) were dissolved in THF (15 mL) and 2M K_2CO_3 solution (7.5 mL) was added. Pd_2dba_3 (17.5 mg, 19.1 µmol) was added and the mixture was stirred for 2.5 d at 80 °C. The reaction was left to cool to r.t., the organic phase was separated, the aqueous phase extracted with THF (2 × 15 mL). The organic phases were combined and dried over magnesium sulfate. Solvents were removed *in vacuo* and the mixture was suspended in dichloromethane and added to a silica plug. First unknown impurities were eluted with dichloromethane, then the macrocycles were eluted with tetrahydrofuran and solid residues remained on the column. The solvent of the tetrahydrofuran fraction was removed *in vacuo.* The resulting solids were redissolved in THF, syringe filtrated and the solvent was removed *in vacuo*. The resulting solids were taken up in 50% THF/*n*-heptane and purified by HPLC (SiO_2, 50% THF/*n*-heptane) to obtain **[6.6]CAPP-OH** (5.3 mg, 5%) and **[8.8]CAPP-OH** (2.3 mg, 2%).

[6.6]CAPP-OTES-O_2:

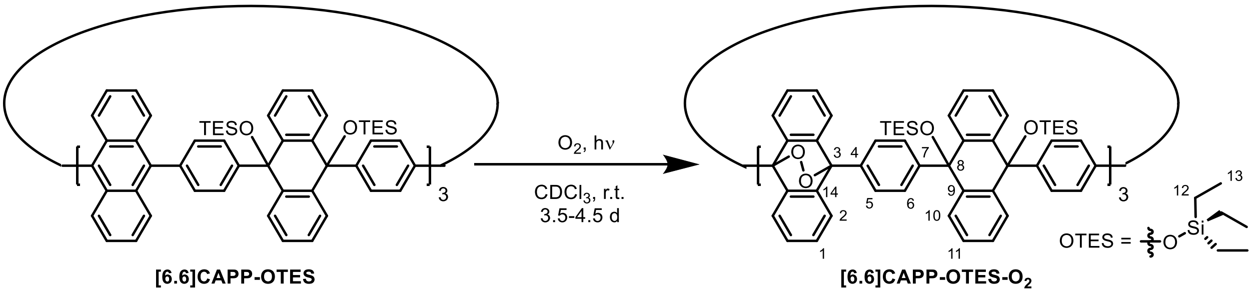

[6.6]-CAP-OTES (15.0 mg, 6.5 µmol) was dissolved in $CDCl_3$ (2 mL) and placed under an oxygen atmosphere. The mixture was exposed to sunlight and stirred for 3.5-4.5 d (The reaction process was monitored by ^{1}H NMR). Solvents were removed in vacuo to obtain the peroxide **[6.6]CAPP-OTES-O_2** compound as colorless solid (15.2 mg, 6.3 µmol) in 97% yield.

M.p.: >182 °C decomp. **^{1}H NMR (600 MHz, $CDCl_3$):** δ = 7.71 (dd, *J* = 6.1, 3.4, 4H, H-10), 7.56 (d, *J* = 8.7, 4H, H-6), 7.44 (d, *J* = 8.7, 4H, H-5), 7.36 (dd, *J* = 6.1, 3.4, 4H, H-11), 6.96 (d, *J* = 1.5, 8H, H-1+H-2), 0.94 (t, *J* = 8.0, 18H, H-13), 0.48 (q, *J* = 8.0, 12H, H-12) ppm. **^{13}C NMR**

(151 MHz, $CDCl_3$): δ = 151.3 (C-4), 140.1 (C-14), 139.7 (C-9), 131.2 (C-7), 129.4 (C-10), 127.9 (C-11), 127.5 (C-1/2), 126.8 (C-5), 126.7 (C-6), 123.3 (C-1/2), 83.9 (C-3), 76.3 (C-8), 7.5 (C-13), 6.7 (C-12) ppm. **IR:** $\tilde{\nu}$ = 2953 (w), 2934 (w), 2908 (w), 2874 (m), 1505 (w), 1456 (m), 1446 (m), 1408 (w), 1260 (w), 1239 (m), 1177 (w), 1159 (w), 1140 (w), 1082 (s), 1059 (s), 1007 (s), 987 (m), 974 (w), 943 (w), 927 (w), 906 (m), 888 (w), 848 (m), 818 (s), 758 (s), 725 (vs), 680 (s), 668 (m), 667 (m), 640 (vs), 617 (w), 579 (m), 557 (m) cm^{-1}. **UV-Vis (dichloromethane):** λ_{max} (lg ε) = 263 (3.97) nm. **MS (HR-MALDI):** *m/z* calculated for $[M]^+$: 2395.0676, found 2395.0672.

[6.6]CAPP:

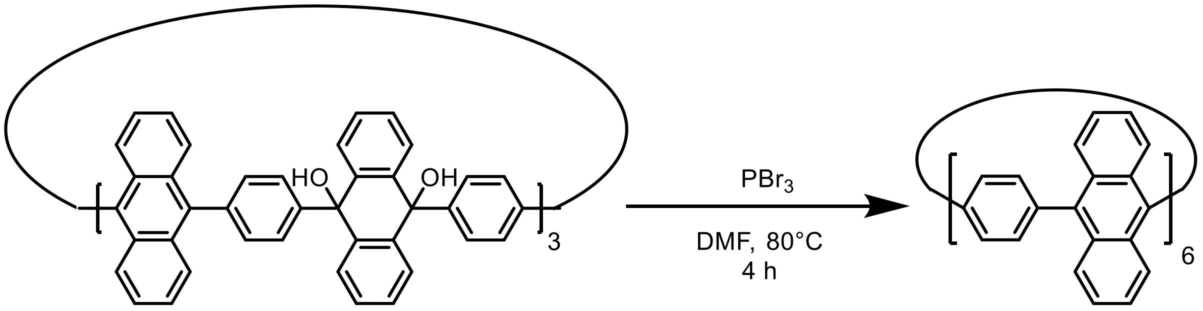

[6.6]CAPP-OH **[6.6]CAPP**

[6.6]CAPP-OH (10 mg, 6.2 µmol) was suspended in dry DMF (3 mL) under an argon atmosphere. Phosphorous tribromide (0.06 mL, 668 µmol) was added and the mixture was heated to 80°C for 4 h. After cooling to r.t. a sample was taken for MALDI-TOF MS. The solution was carefully removed and the remaining precipitate washed with dry DMF (2 × 1 mL). The solids were suspended in DMF (1 mL) and the suspension (ca. 5-10 µL) was added into a fluorescence cuvette with DCM (2 mL) under an argon atmosphere.

UV-Vis (dichloromethane): λ_{max} = 406, 385, 365 nm. **Fluorescence (dichloromethane, λ_{exc} = 350 nm):** λ_{max} = 427 nm. **MS (HR-MALDI):** *m/z* calculated for $[M]^+$: 1512.5629, found 1512.5635.

[8.8]CAPP:

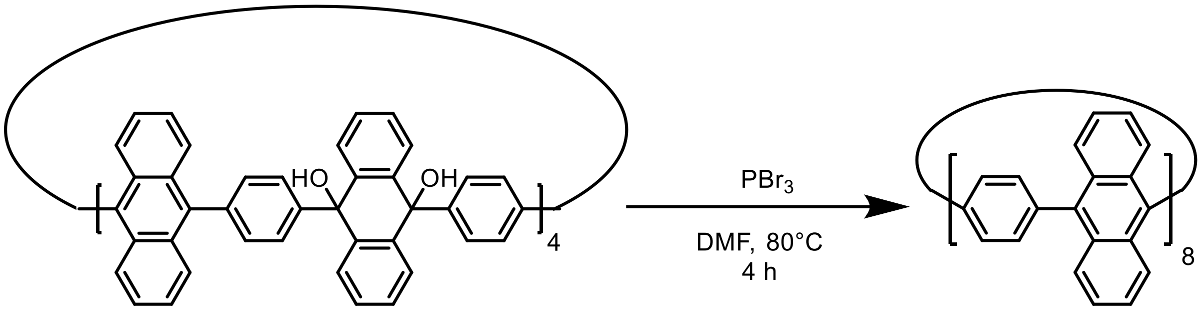

[8.8]CAPP-OH **[8.8]CAPP**

[8.8]CAPP-OH (2 mg, 0.9 µmol) was suspended in dry DMF (1 mL) under an argon atmosphere. Phosphorous tribromide (0.01 mL, 134 µmol) was added and the mixture was heated to 80°C for 4 h. After cooling to r.t. a sample was taken for MALDI-TOF MS (as this showed only traces of $[M]^+$, no UV/Vis was measured).

MS (HR-MALDI): *m/z* calculated for $[M]^+$: 2016.7507, found 2016.7537, *m/z* calculated for $[M+O]^+$: 2032.7456, found 2032.7487.

3 Spectra

3.1 ^{1}H NMR and ^{13}C NMR spectra

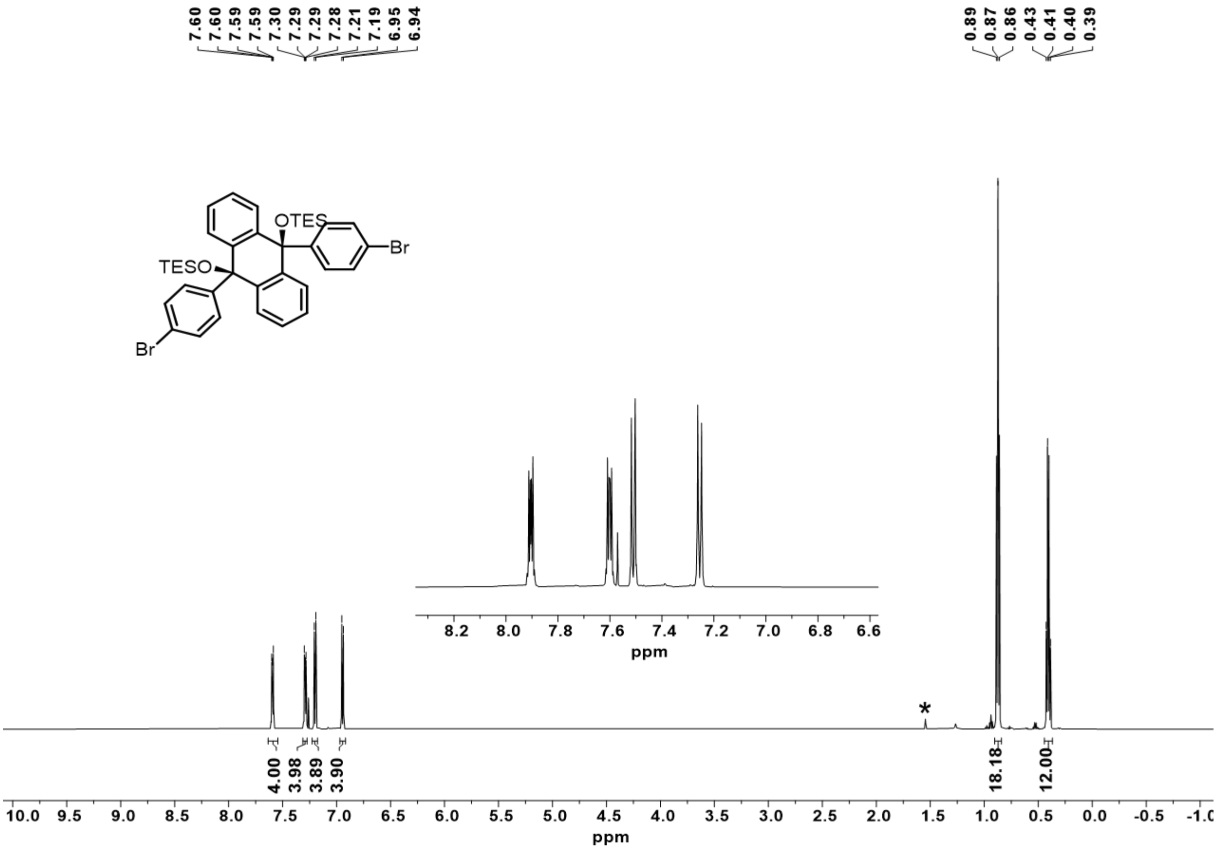

Figure S2: ^{1}H NMR spectrum of **4** ($CDCl_3$, 600 MHz, 300K). *water

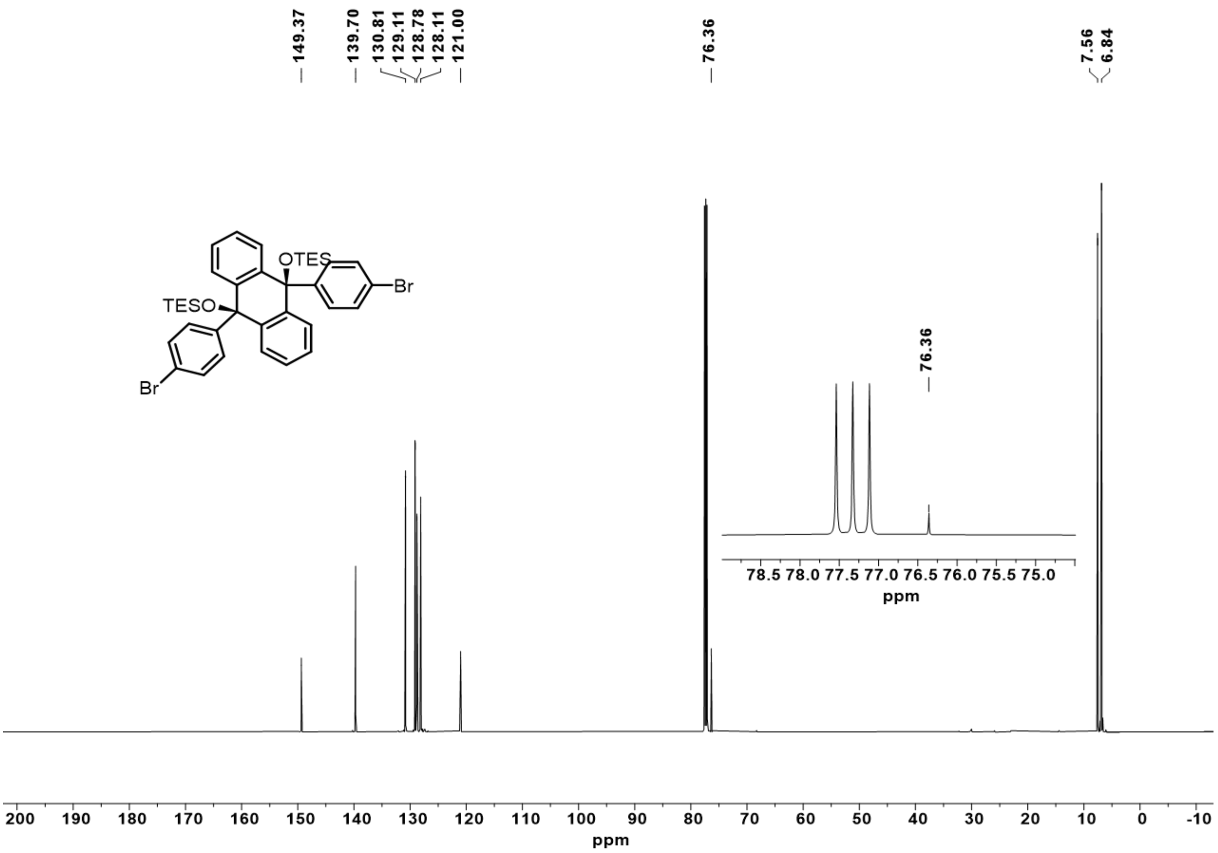

Figure S3: ^{13}C NMR spectrum of **4** ($CDCl_3$, 151 MHz, 300K).

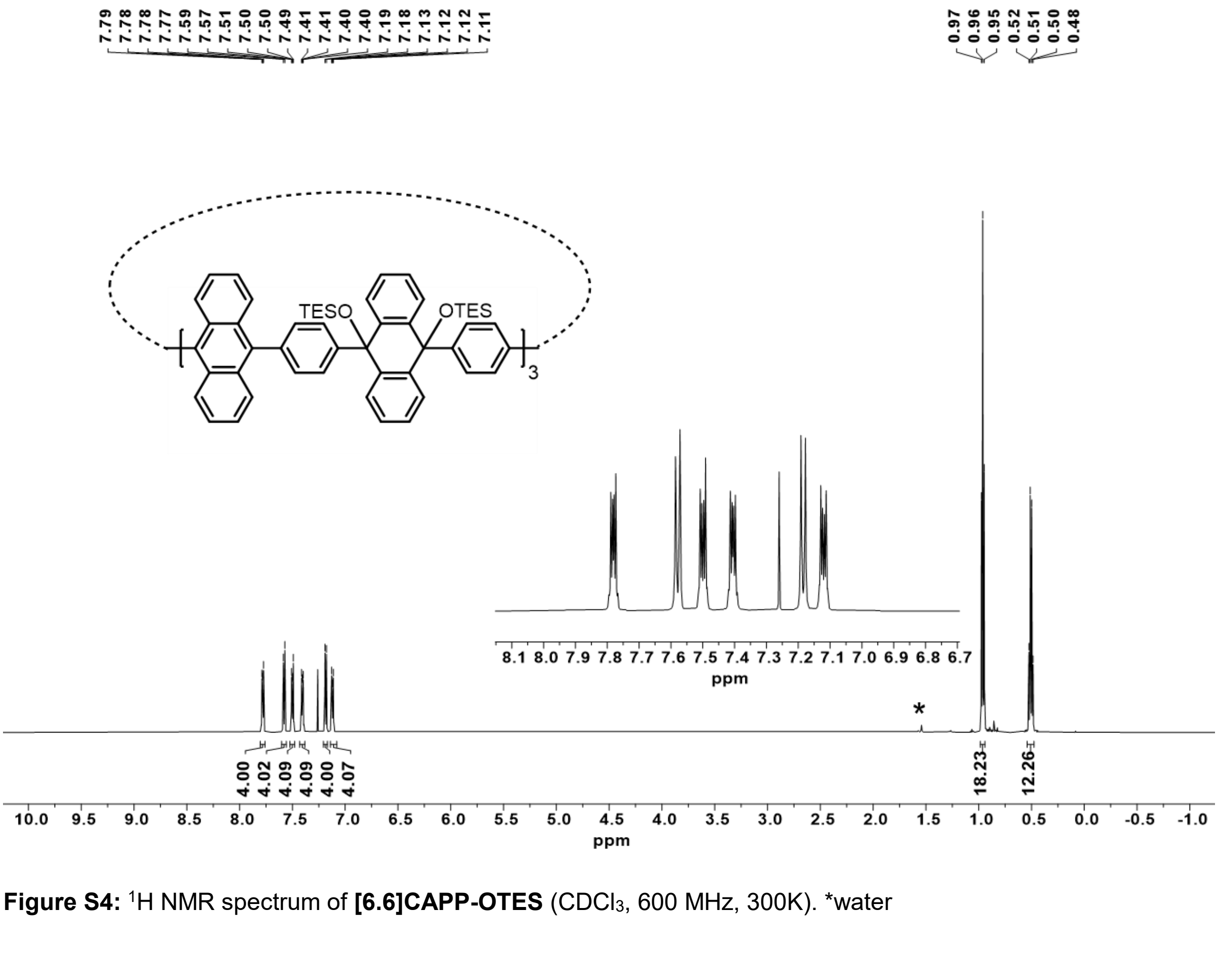

Figure S4: ^{1}H NMR spectrum of **[6.6]CAPP-OTES** ($CDCl_3$, 600 MHz, 300K). *water

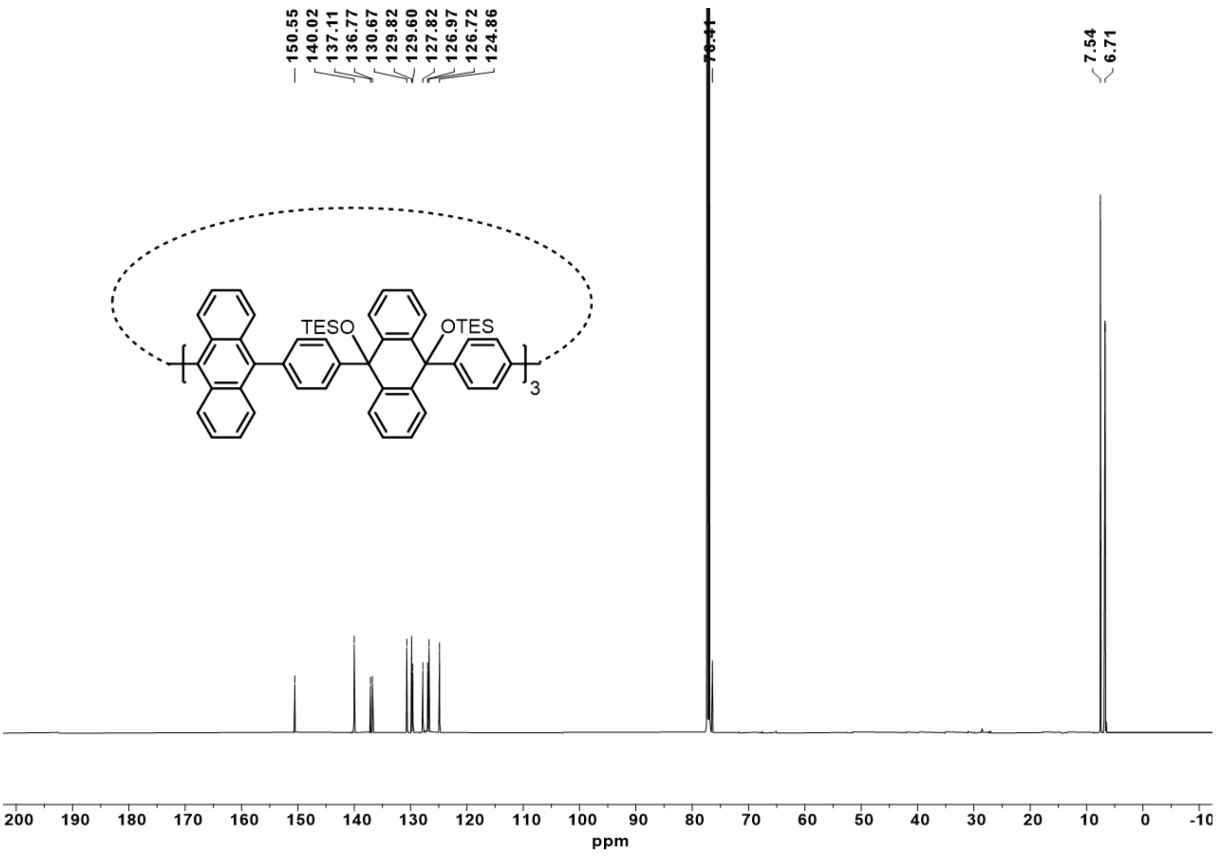

Figure S5: ^{13}C NMR spectrum of **[6.6]CAPP-OTES** ($CDCl_3$, 151 MHz, 300K).

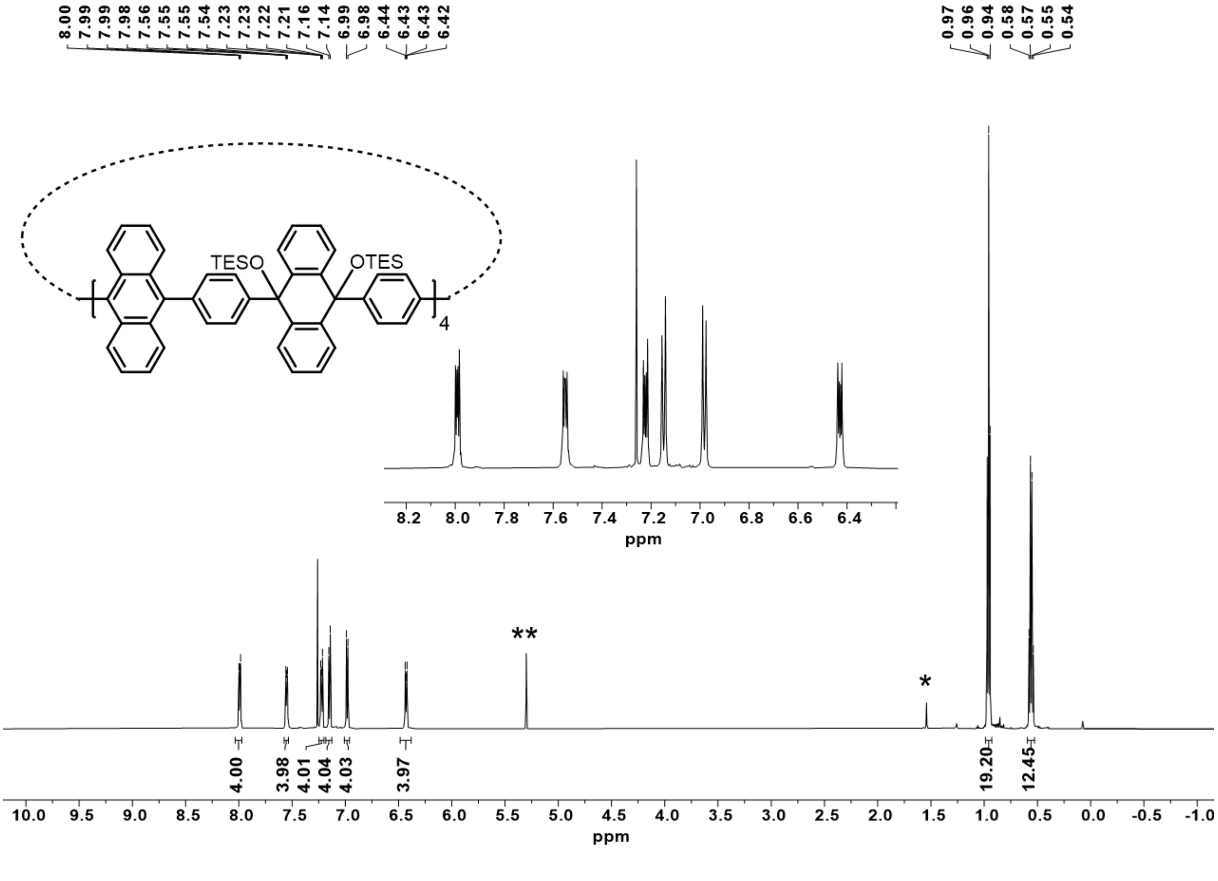

Figure S6: ^{1}H NMR spectrum of **[8.8]CAPP-OTES** ($CDCl_3$, 600 MHz, 300K). *water, ** dichloromethane

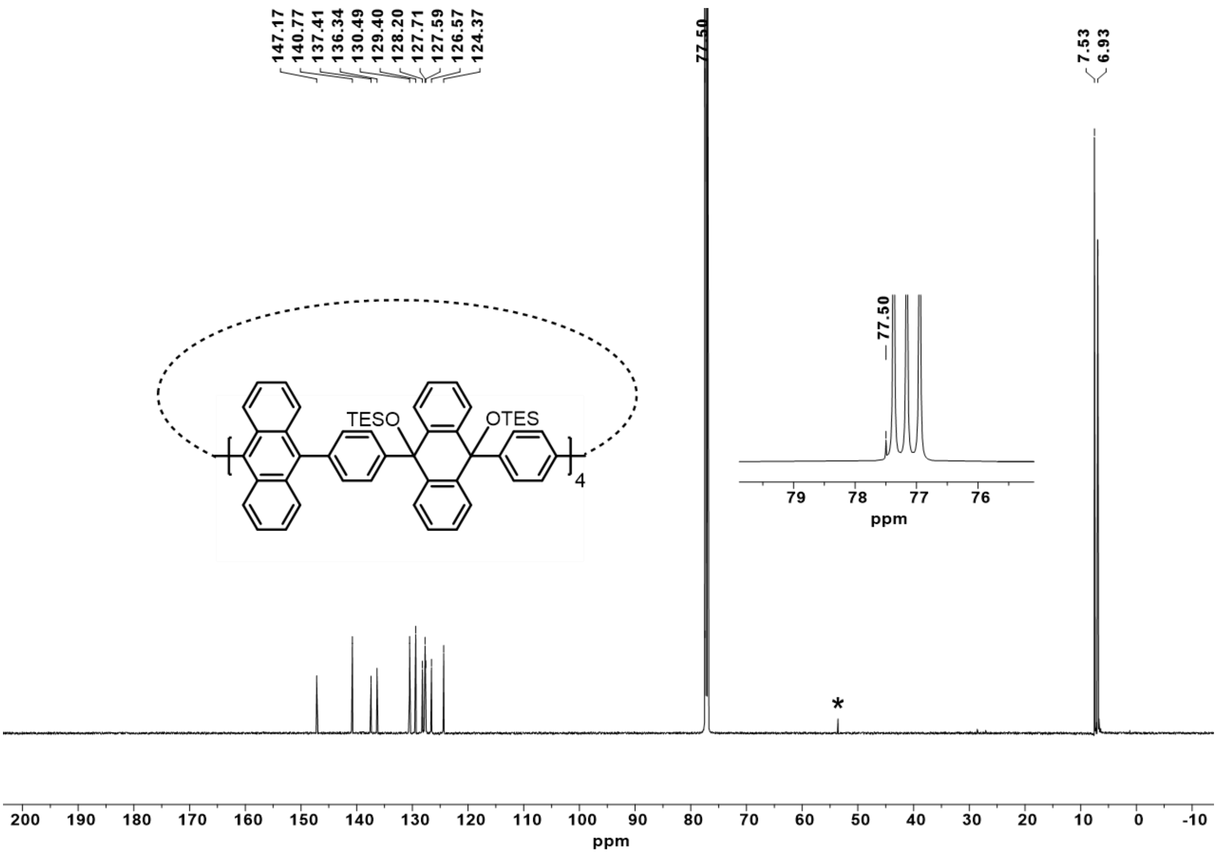

Figure S7: ^{13}C NMR spectrum of **[8.8]CAPP-OTES** ($CDCl_3$, 151 MHz, 300K). *dichloromethane

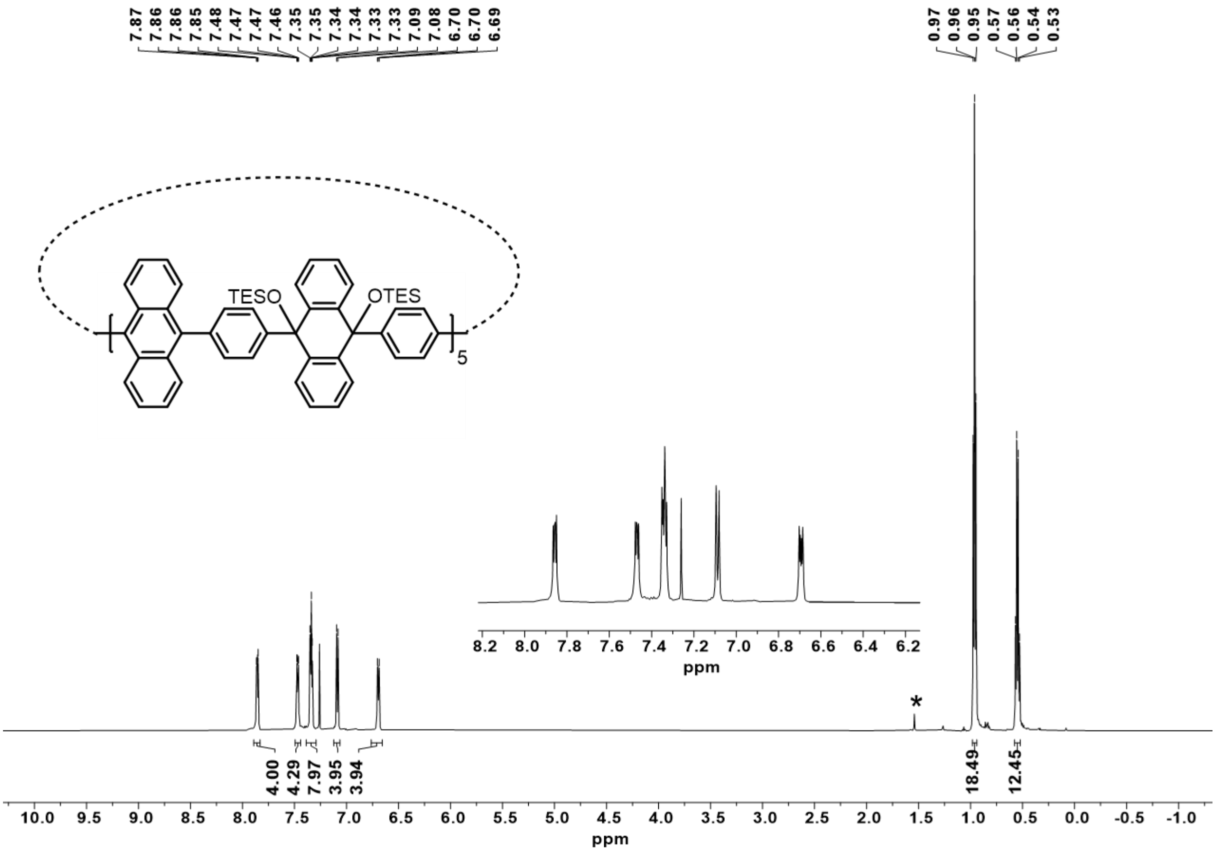

Figure S8: ^{1}H NMR spectrum of **[10.10]CAPP-OTES** ($CDCl_3$, 600 MHz, 300K). *water

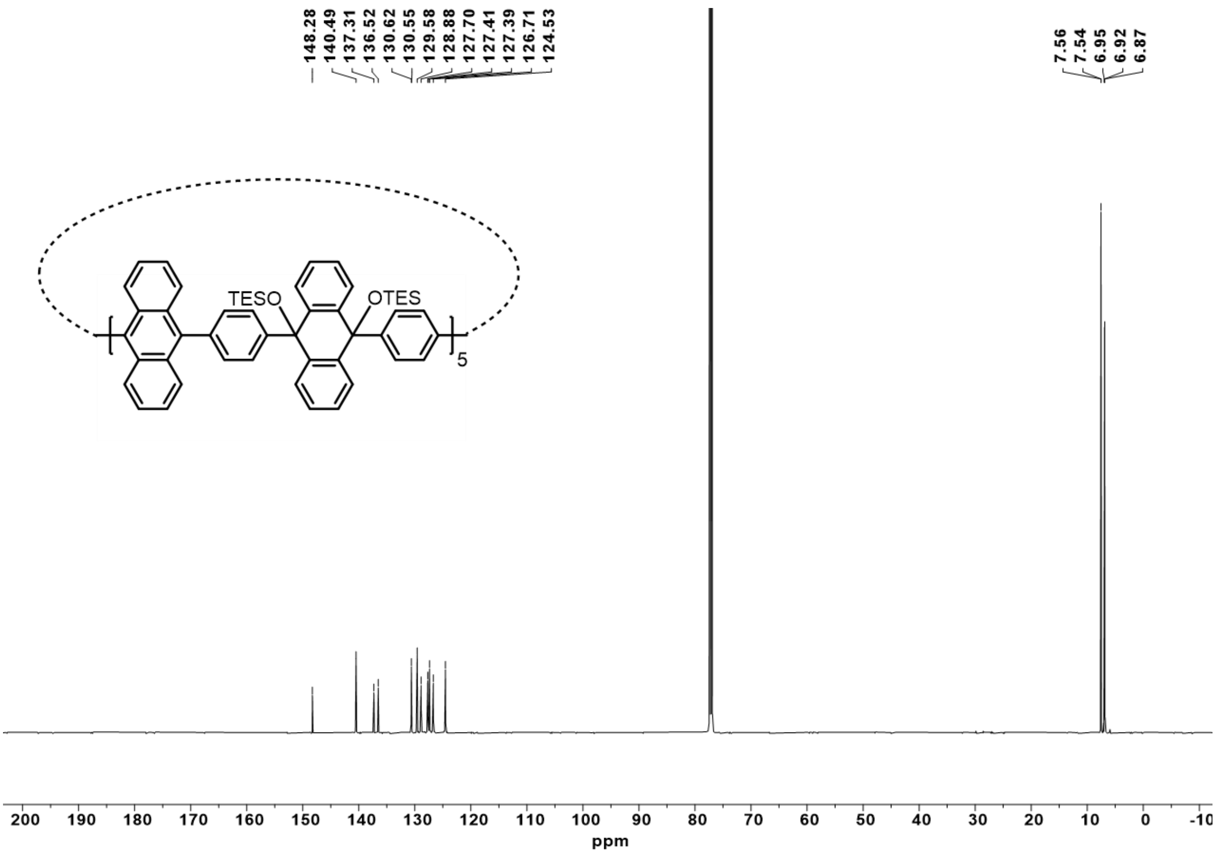

Figure S9: ^{13}C NMR spectrum of **[10.10]CAPP-OTES** ($CDCl_3$, 151 MHz, 300K).

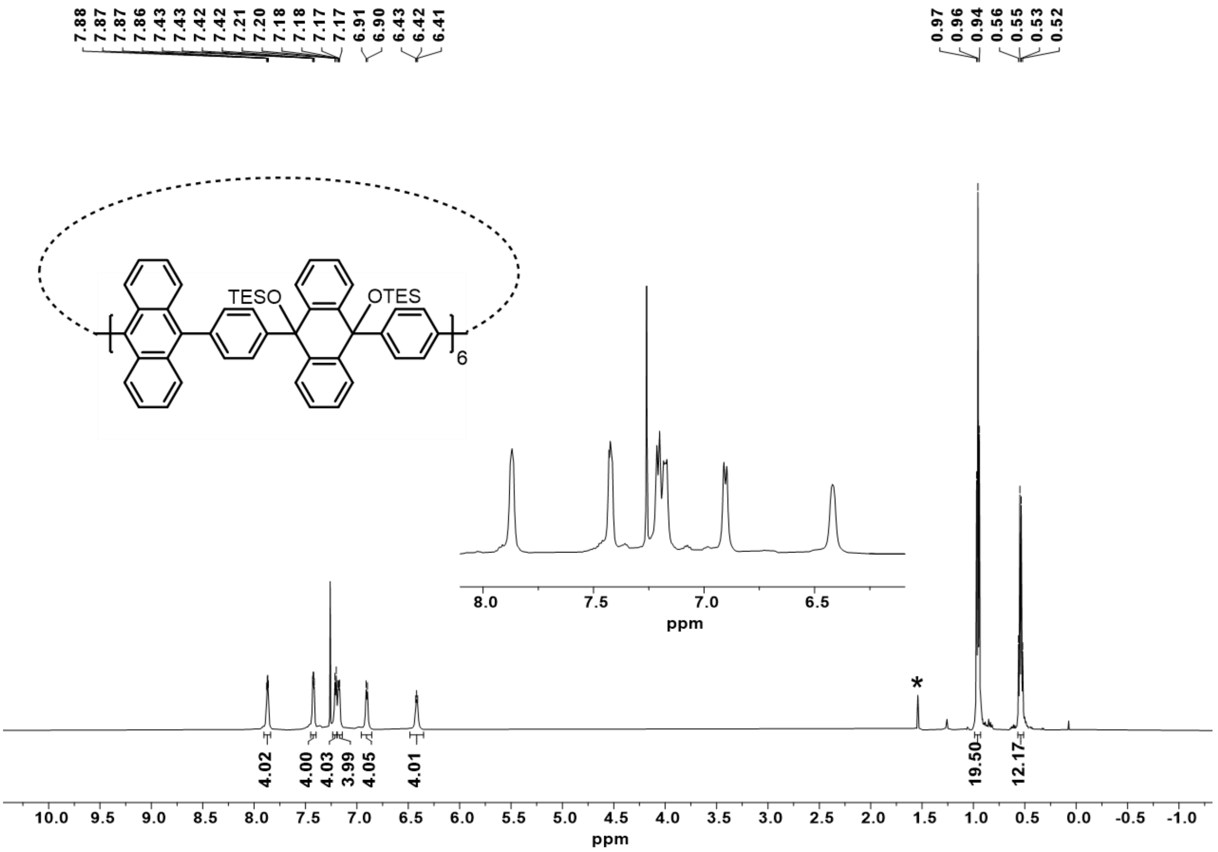

Figure S10: ^{1}H NMR spectrum of **[12.12]CAPP-OTES** ($CDCl_3$, 600 MHz, 300K). *water

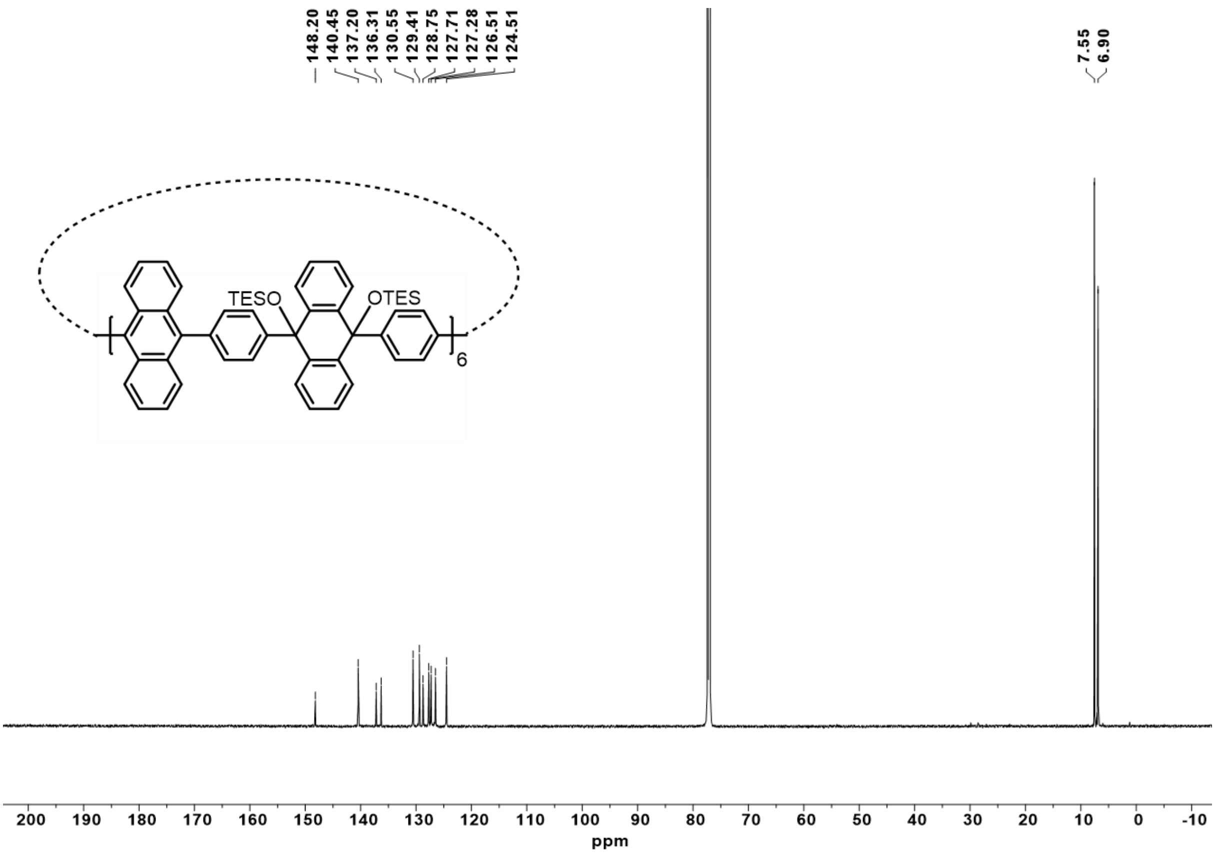

Figure S11: ^{13}C NMR spectrum of **[12.12]CAPP-OTES** ($CDCl_3$, 151 MHz, 300K).

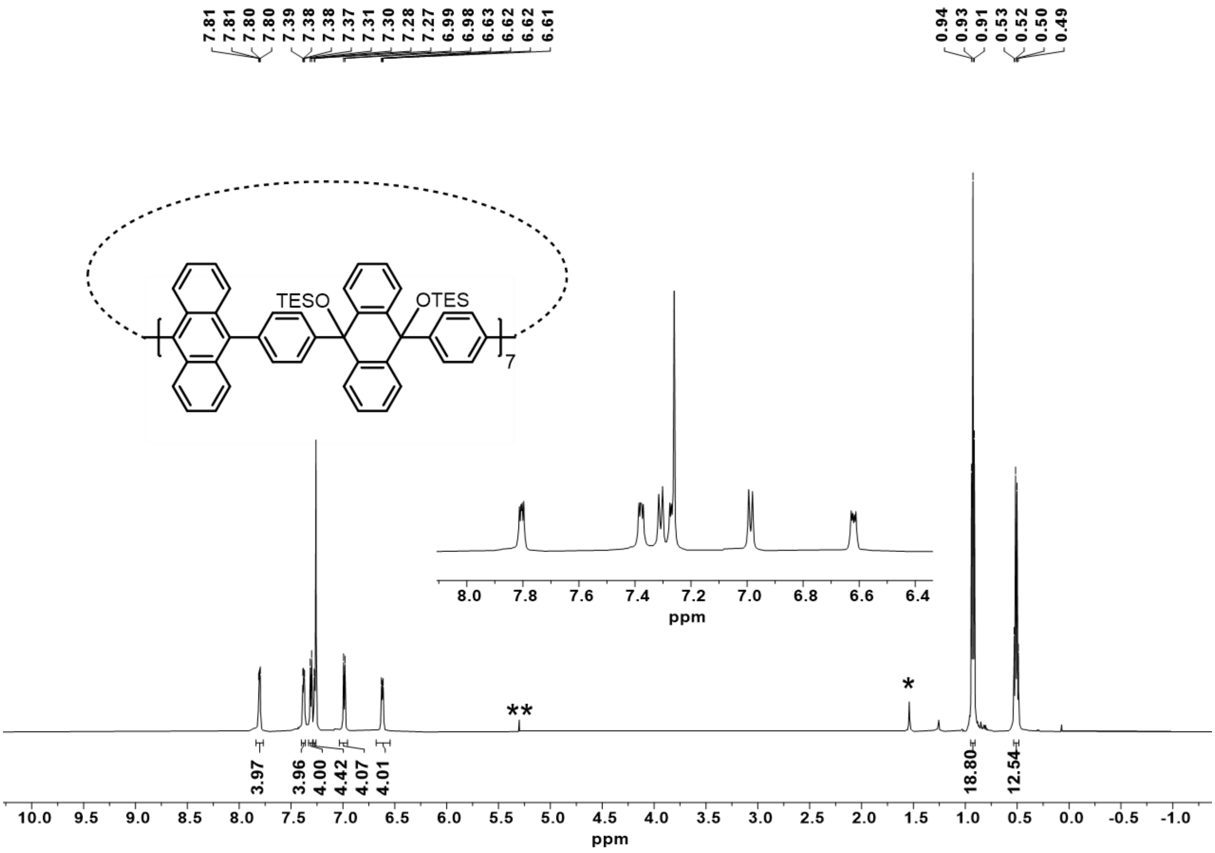

Figure S12: ^{1}H NMR spectrum of **[14.14]CAPP-OTES** ($CDCl_3$, 600 MHz, 300K). *water **DCM

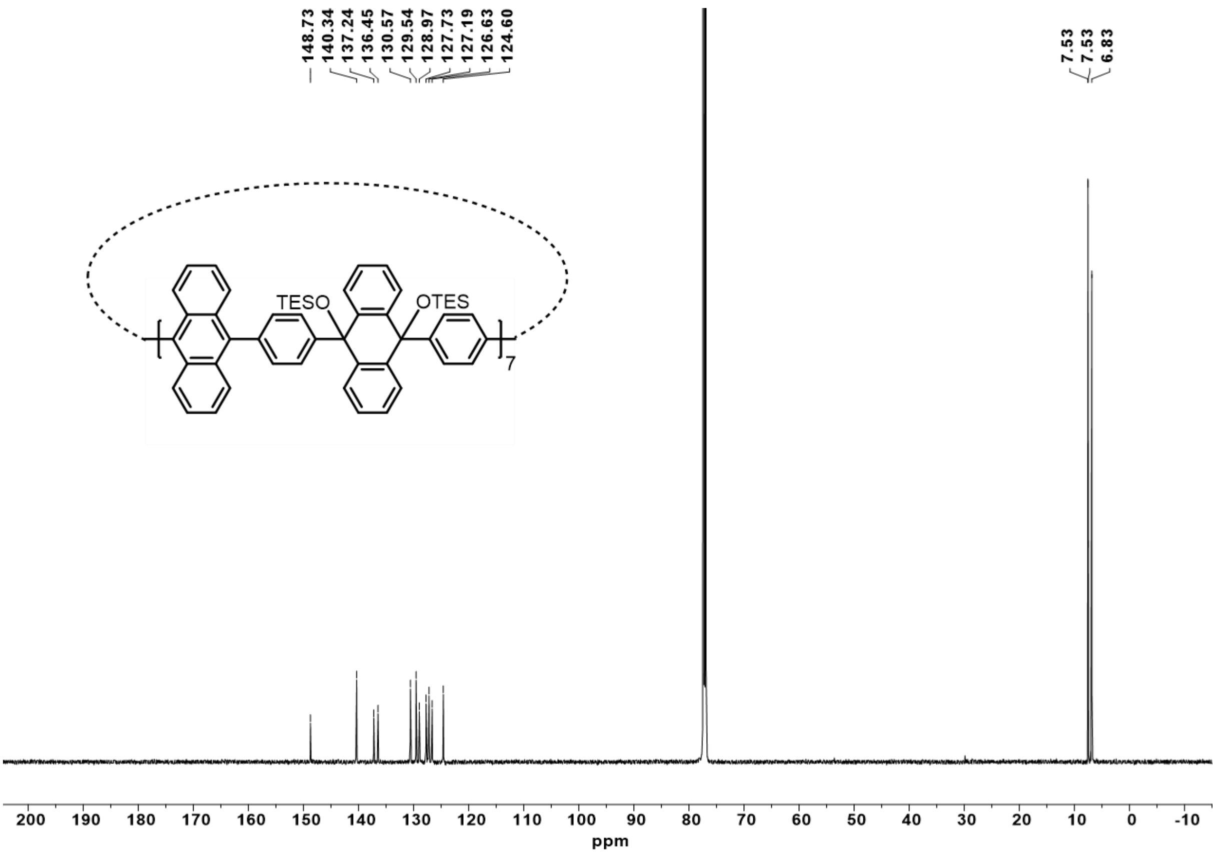

Figure S13: ^{13}C NMR spectrum of **[14.14]CAPP-OTES** ($CDCl_3$, 151 MHz, 300K).

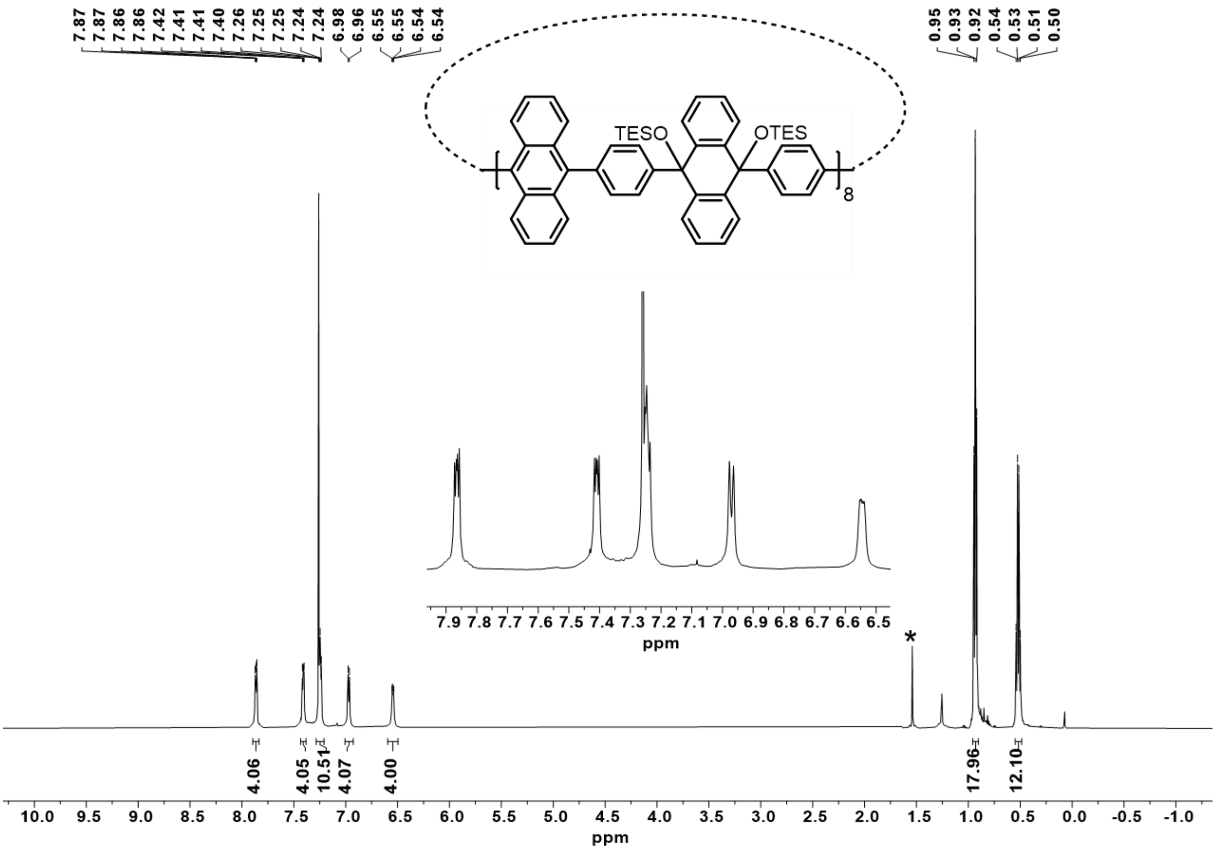

Figure S14: ^{1}H NMR spectrum of **[16.16]CAPP-OTES** ($CDCl_3$, 600 MHz, 300K). *water

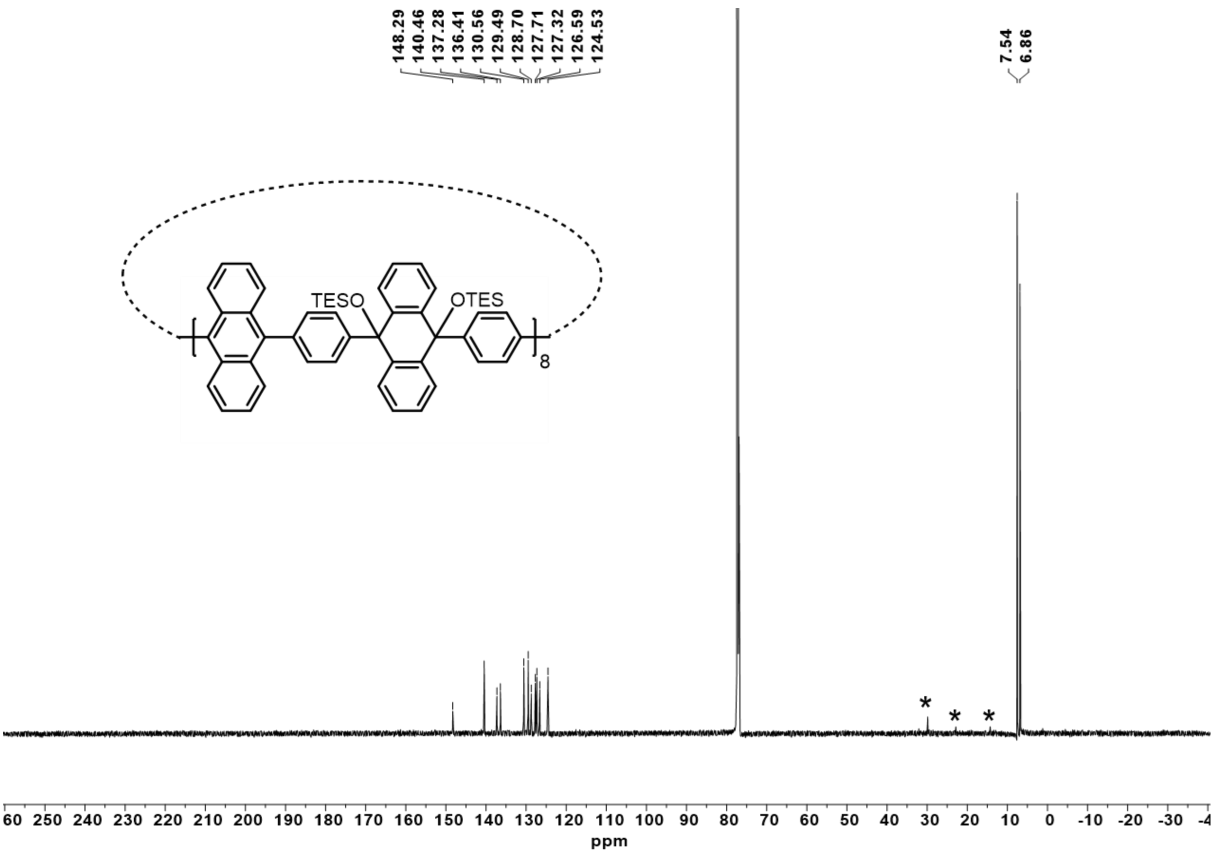

Figure S15: ^{13}C NMR spectrum of **[16.16]CAPP-OTES** ($CDCl_3$, 151 MHz, 300K).

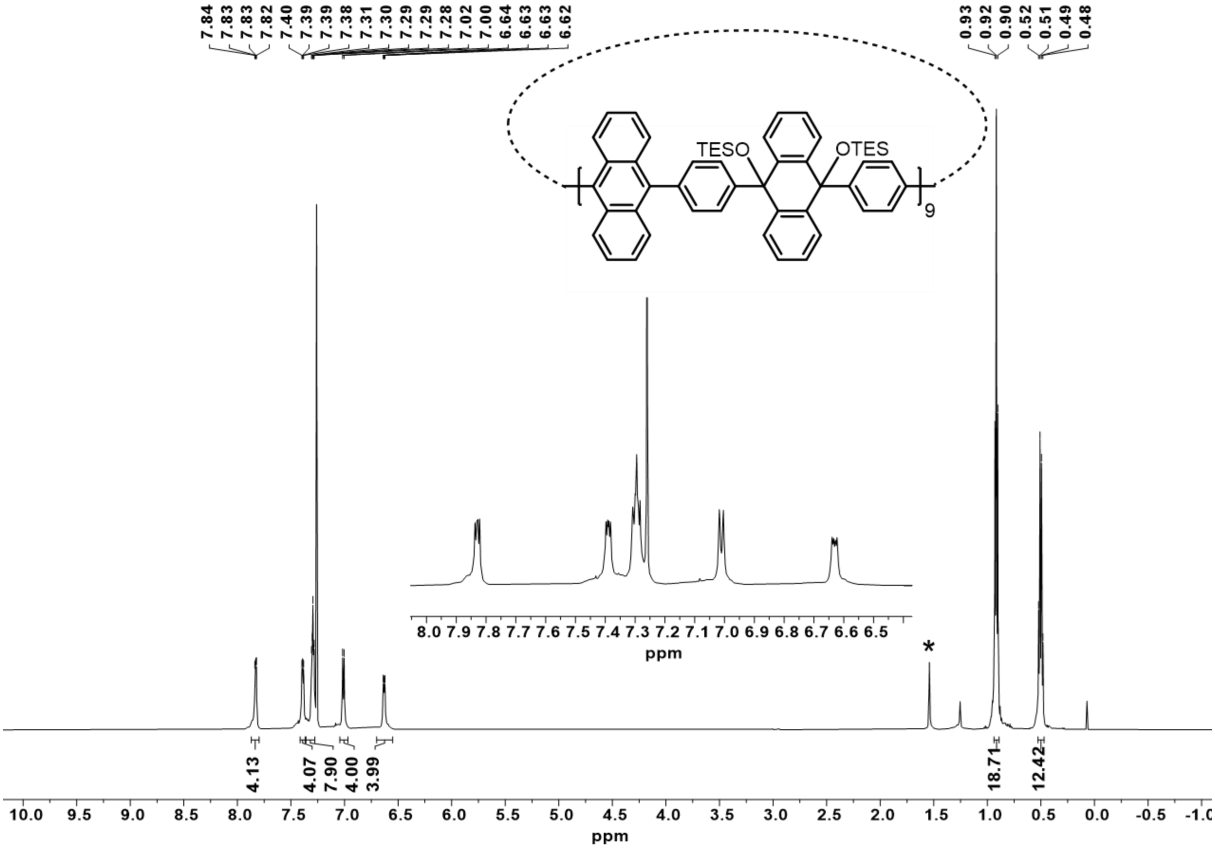

Figure S16: ^{1}H NMR spectrum of **[18.18]CAPP-OTES** ($CDCl_3$, 600 MHz, 300K). *water

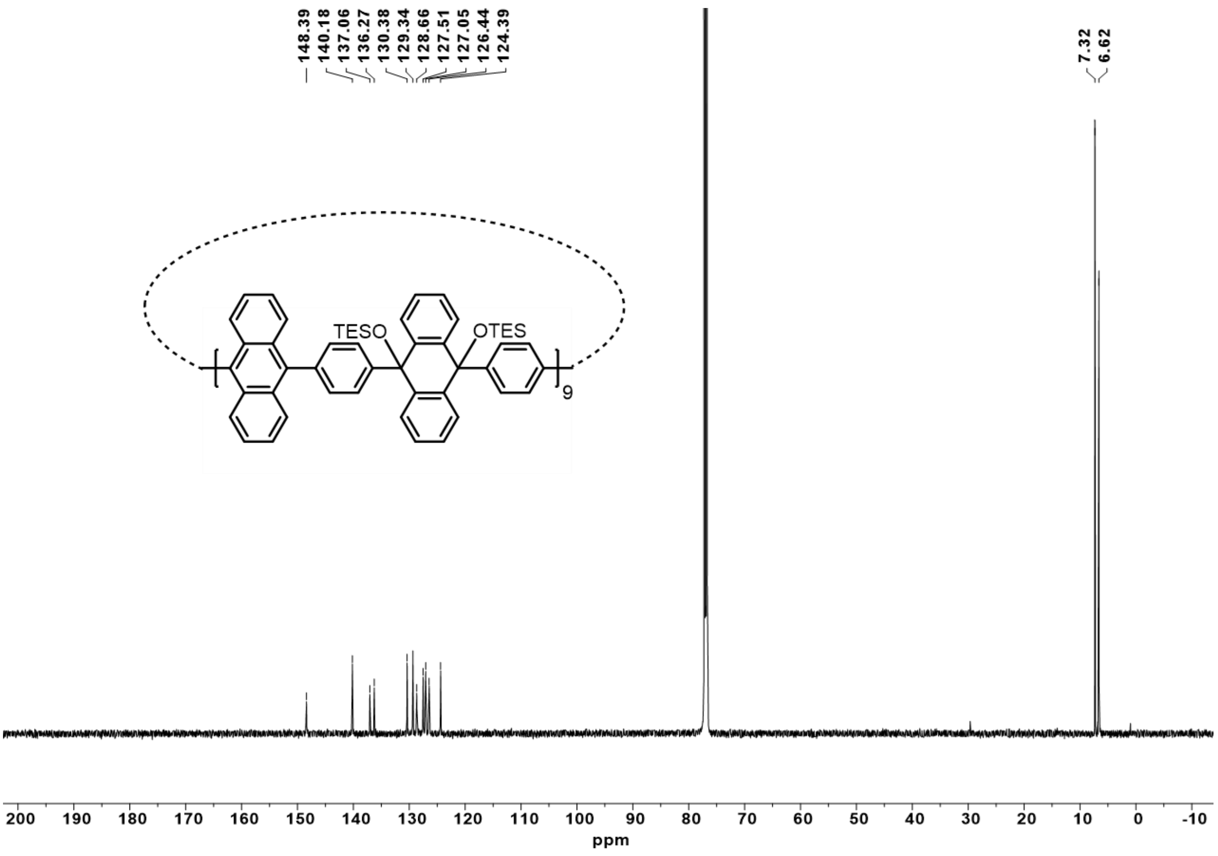

Figure S17: ^{13}C NMR spectrum of **[18.18]CAPP-OTES** ($CDCl_3$, 151 MHz, 300K).

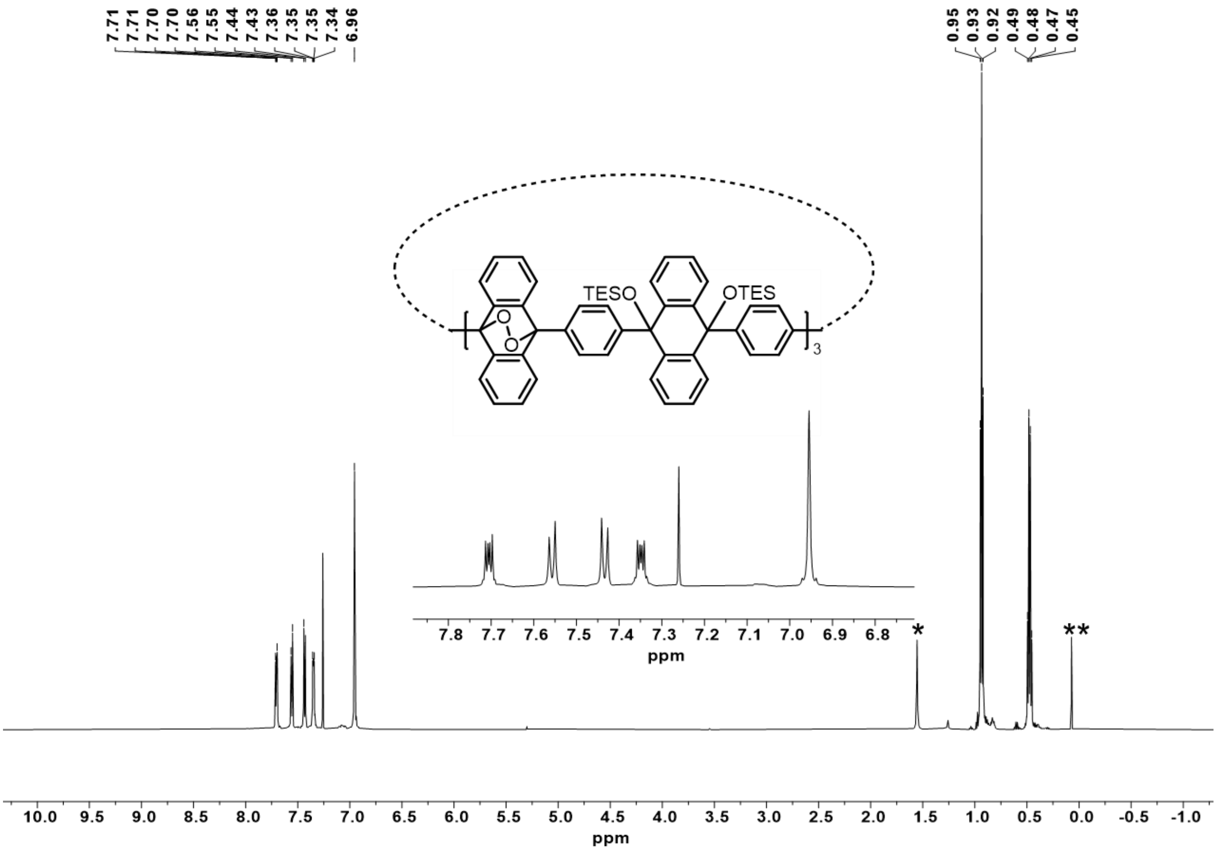

Figure S18: ^{1}H NMR spectrum of **[6.6]CAPP-OTES-O$_2$** ($CDCl_3$, 600 MHz, 300K). * water **silicon grease

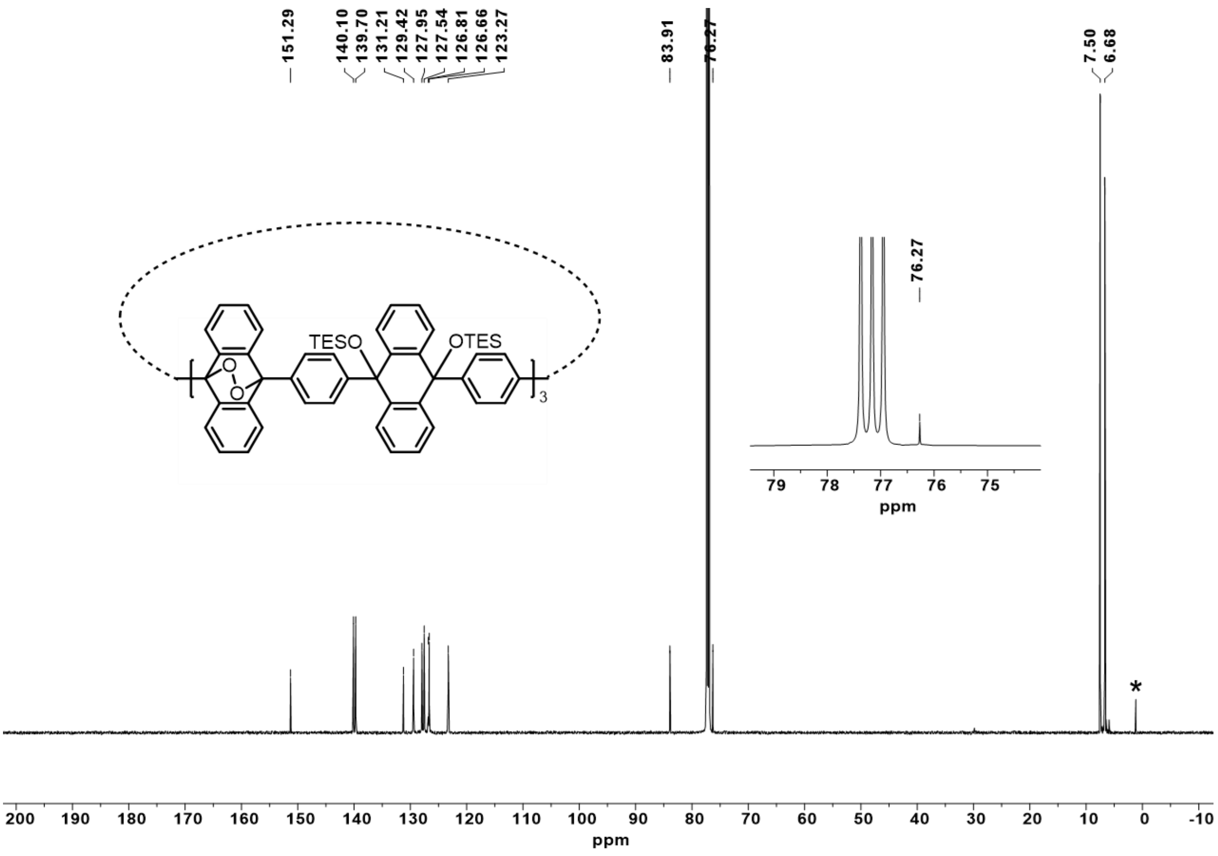

Figure S19: ^{13}C NMR spectrum of **[6.6]CAPP-OTES-O$_2$** ($CDCl_3$, 151 MHz, 300K). *silicon grease

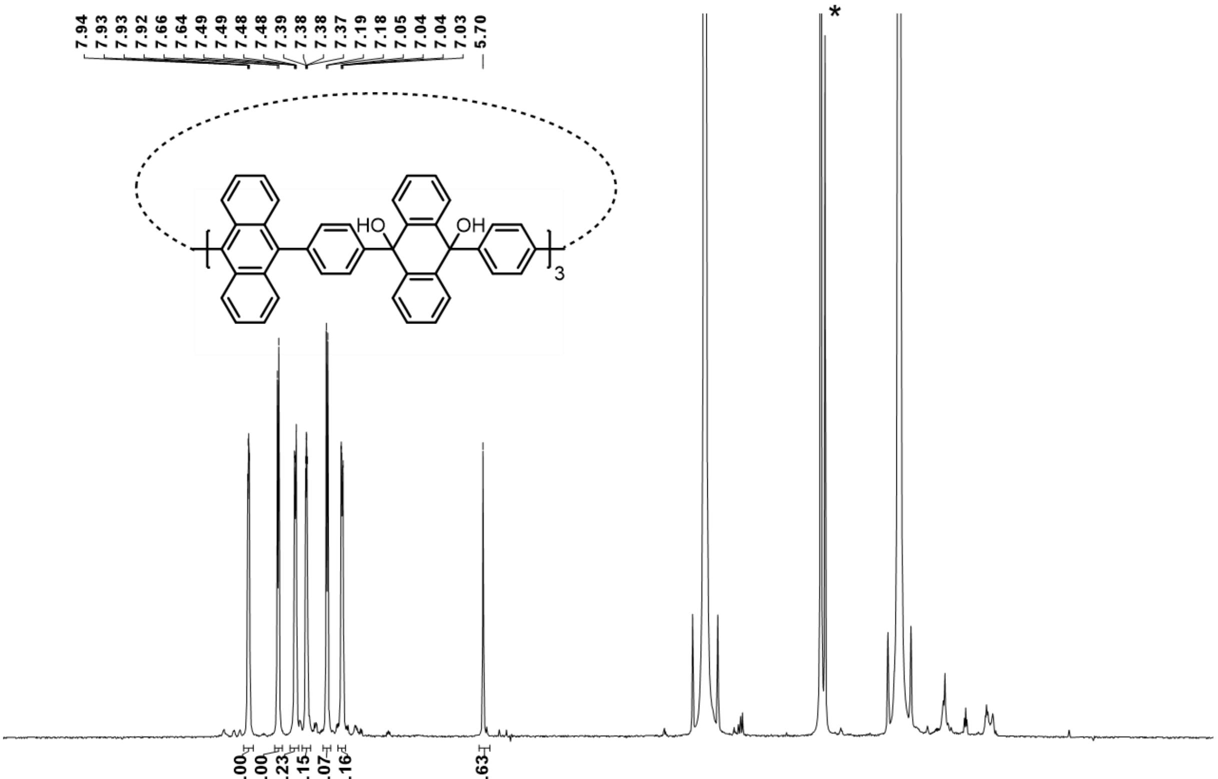

Figure S20: ^{1}H NMR spectrum of **[6.6]CAPP-OH** (THF-d_8, 600 MHz, 300K). *water

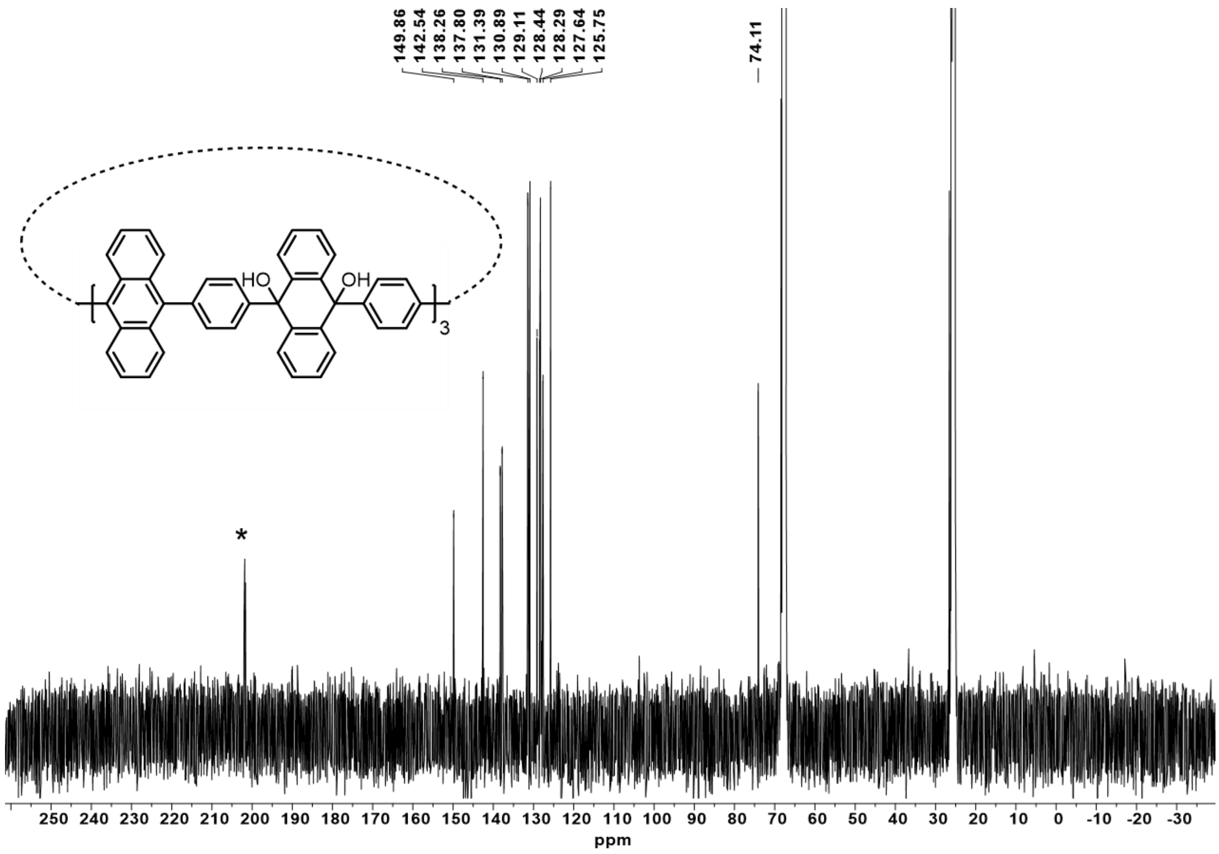

Figure S21: ^{13}C NMR spectrum of **[6.6]CAPP-OH** (THF-d_8, 151 MHz, 300K). *impurities from THF-d8

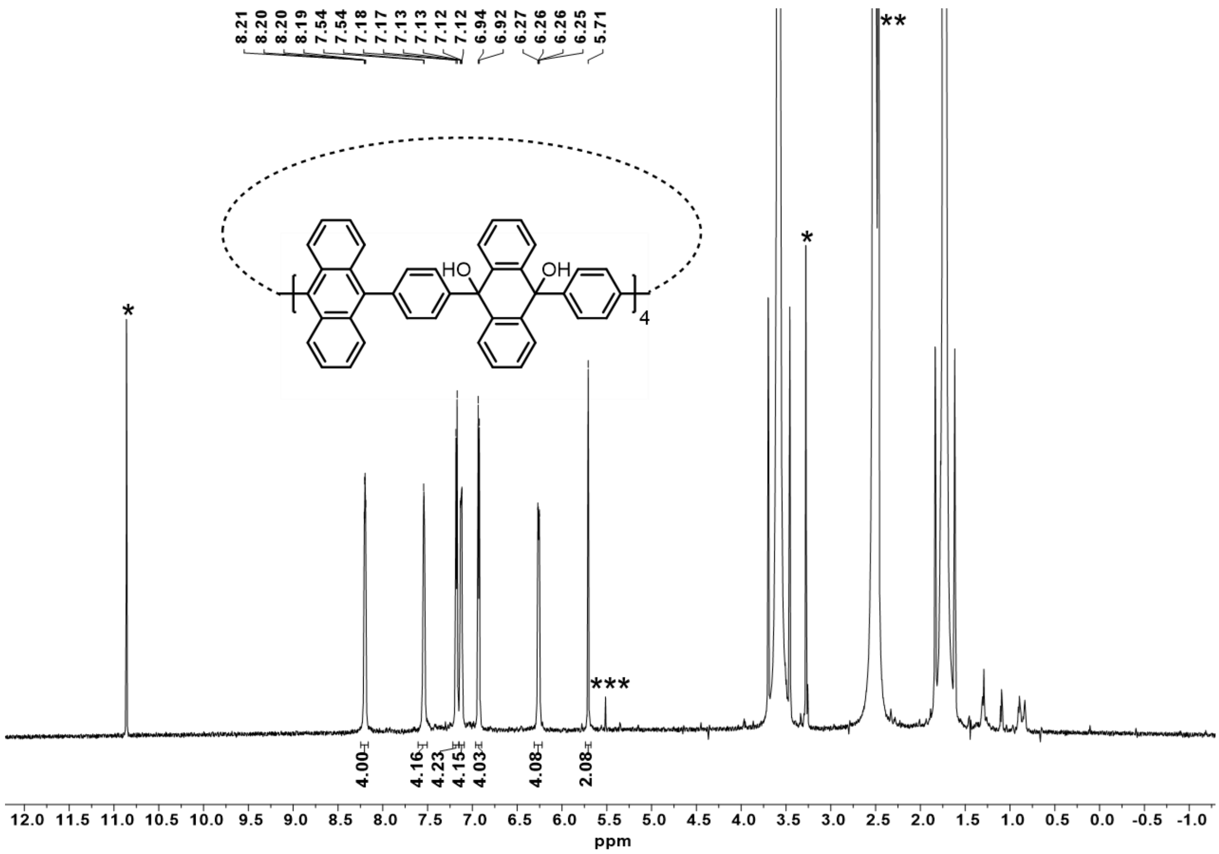

Figure S22: ^{1}H NMR spectrum of **[8.8]CAPP-OH** (THF-d_8, 600 MHz, 300K). *impurities from THF-d8 **water ***DCM

3.2 2D NMR spectra

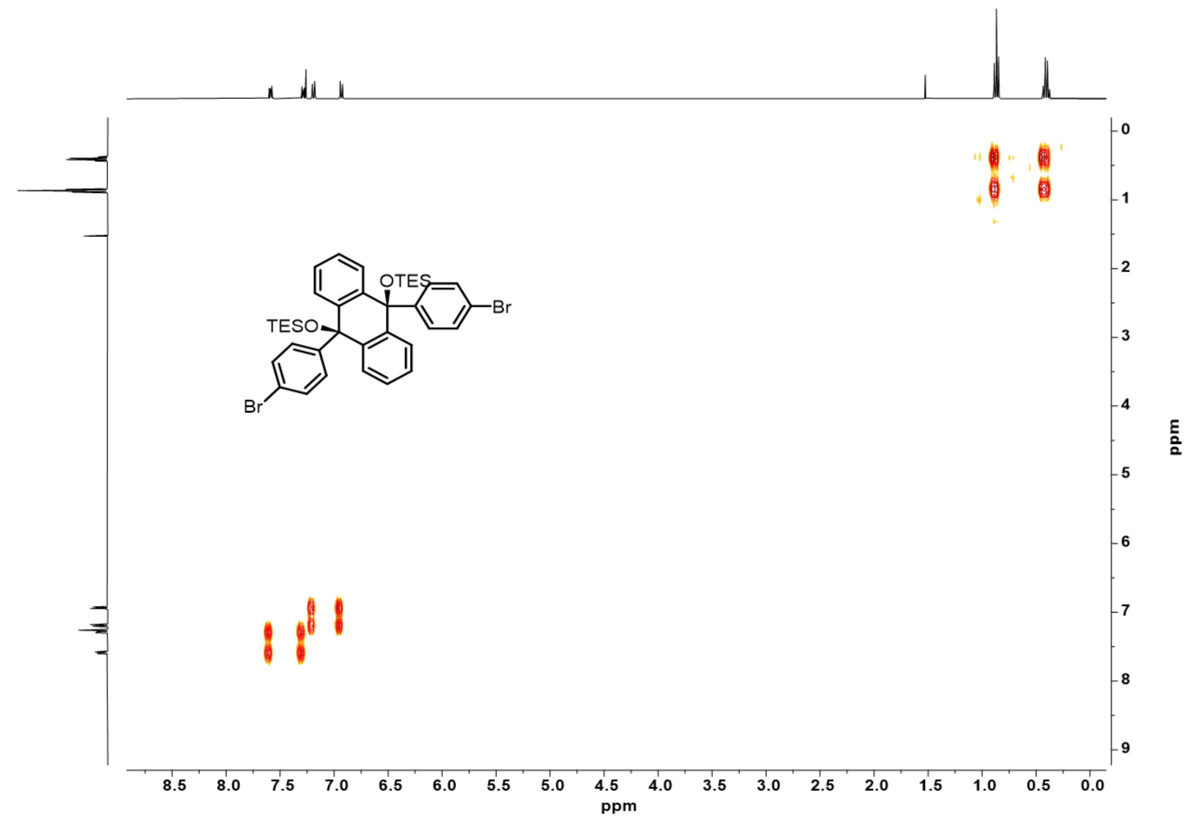

Figure S23: $^{1}H,^{1}H$ COSY spectrum of **4** ($CDCl_3$, 400 MHz, 300 K).

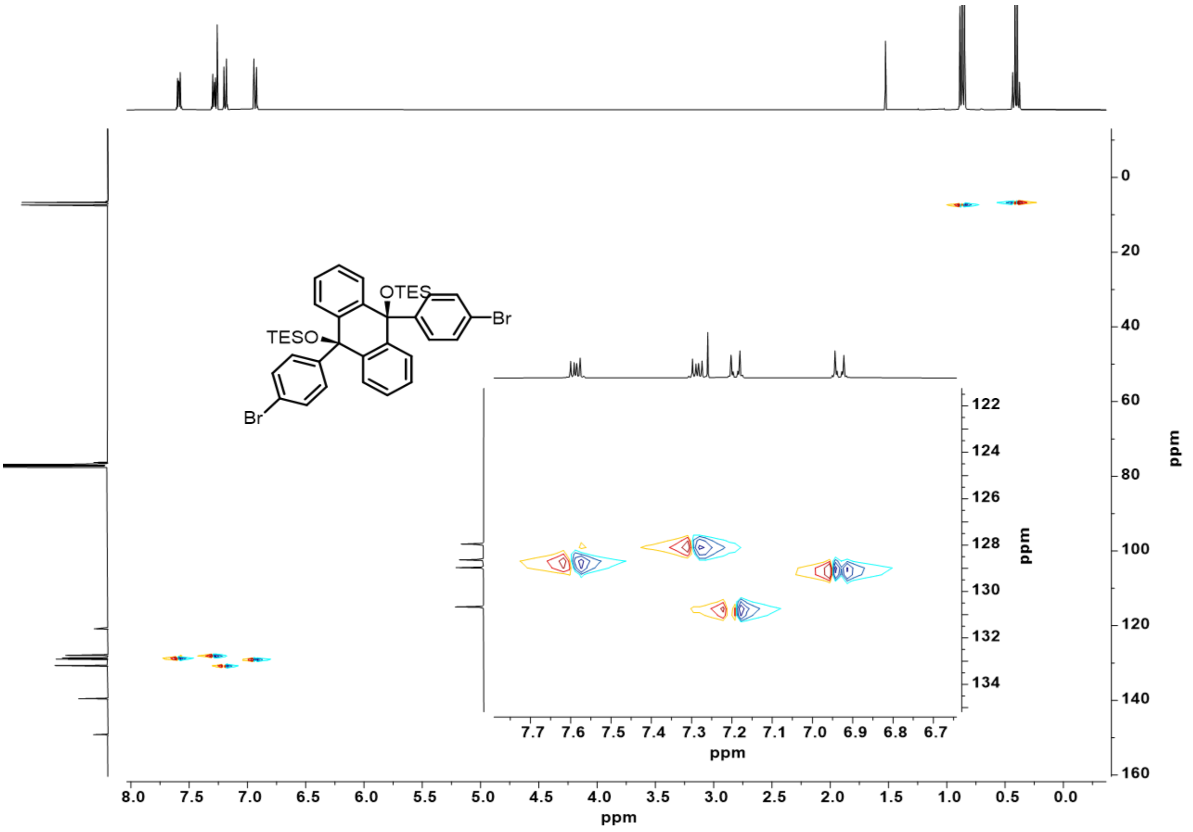

Figure S24: $^{1}H,^{13}C$ HSQC spectrum of **4** ($CDCl_3$, 400 MHz, 101 MHz 300 K).

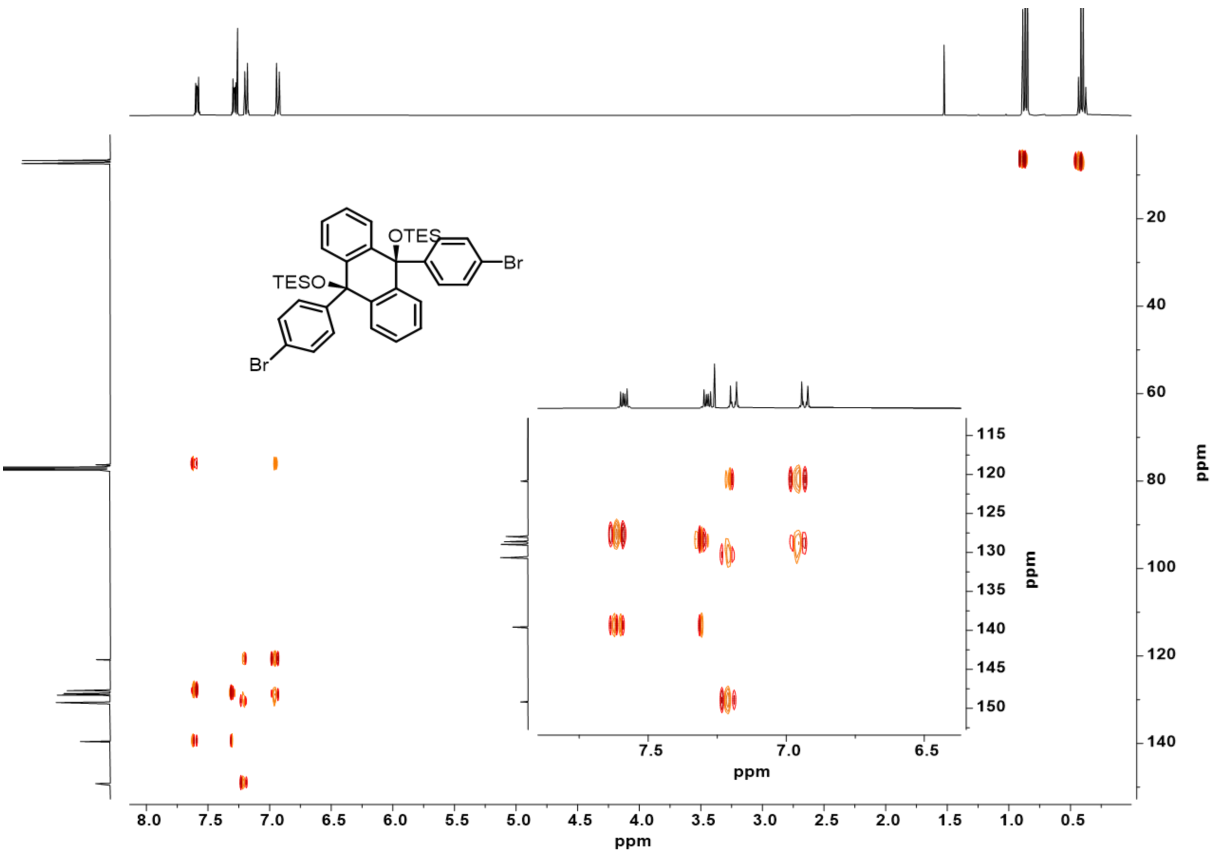

Figure S25: $^{1}H,^{13}C$ HMBC spectrum of **4** ($CDCl_3$, 400 MHz, 101 MHz 300 K).

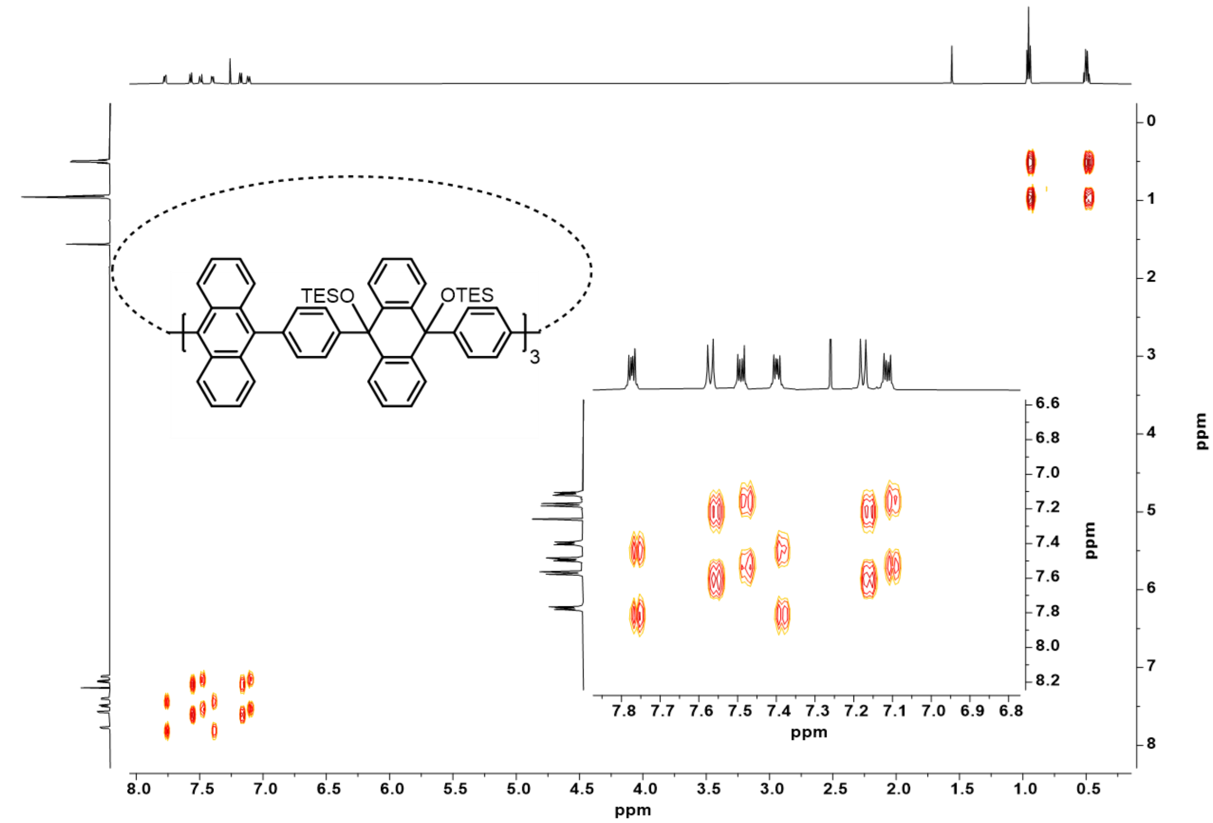

Figure S26: $^{1}H,^{1}H$ COSY spectrum of **[6.6]CAPP-OTES** ($CDCl_3$, 600 MHz, 300 K).

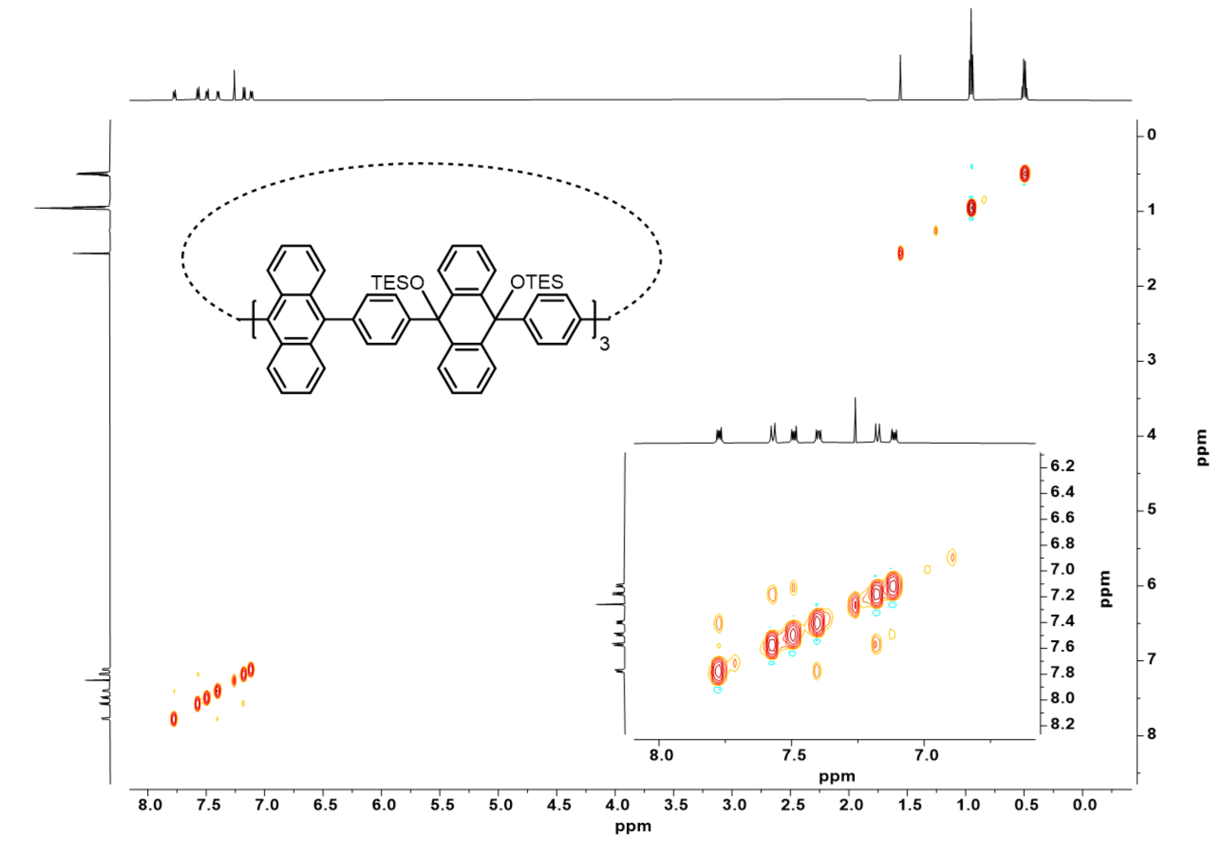

Figure S27: $^{1}H,^{1}H$ NOESY spectrum of **[6.6]CAPP-OTES** ($CDCl_3$, 600 MHz, 300 K).

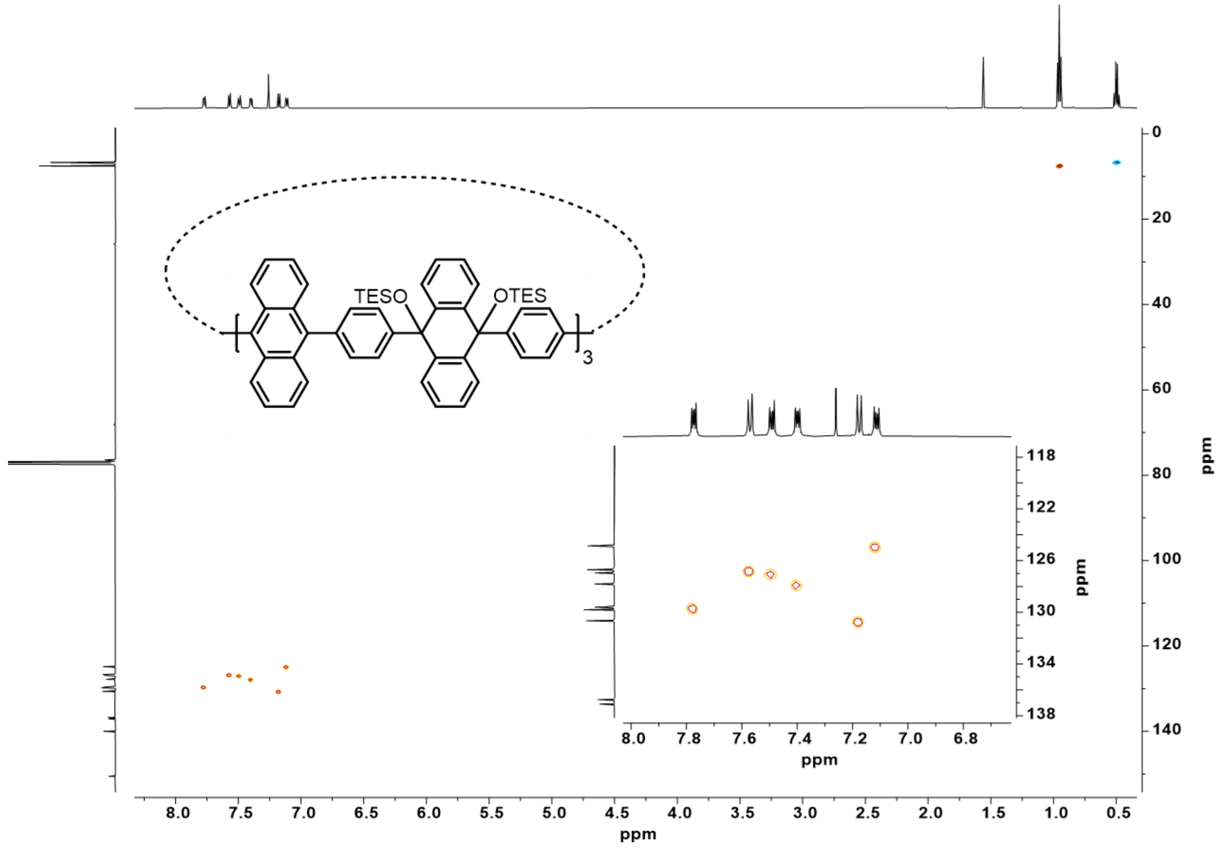

Figure S28: $^{1}H,^{13}C$ HSQC spectrum of **[6.6]CAPP-OTES** ($CDCl_3$, 600 MHz, 151 MHz 300 K).

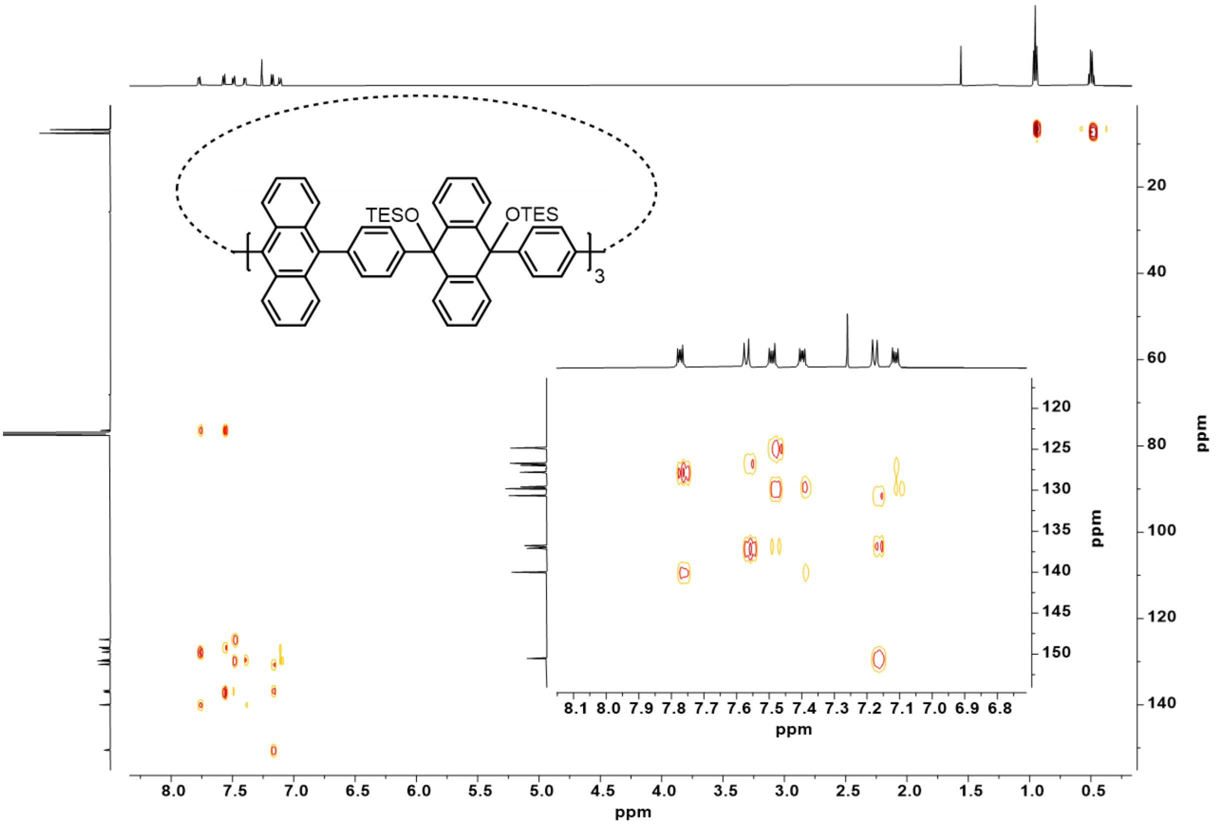

Figure S29: $^{1}H,^{13}C$ HMBC spectrum of **[6.6]CAPP-OTES** ($CDCl_3$, 600 MHz, 151 MHz 300 K).

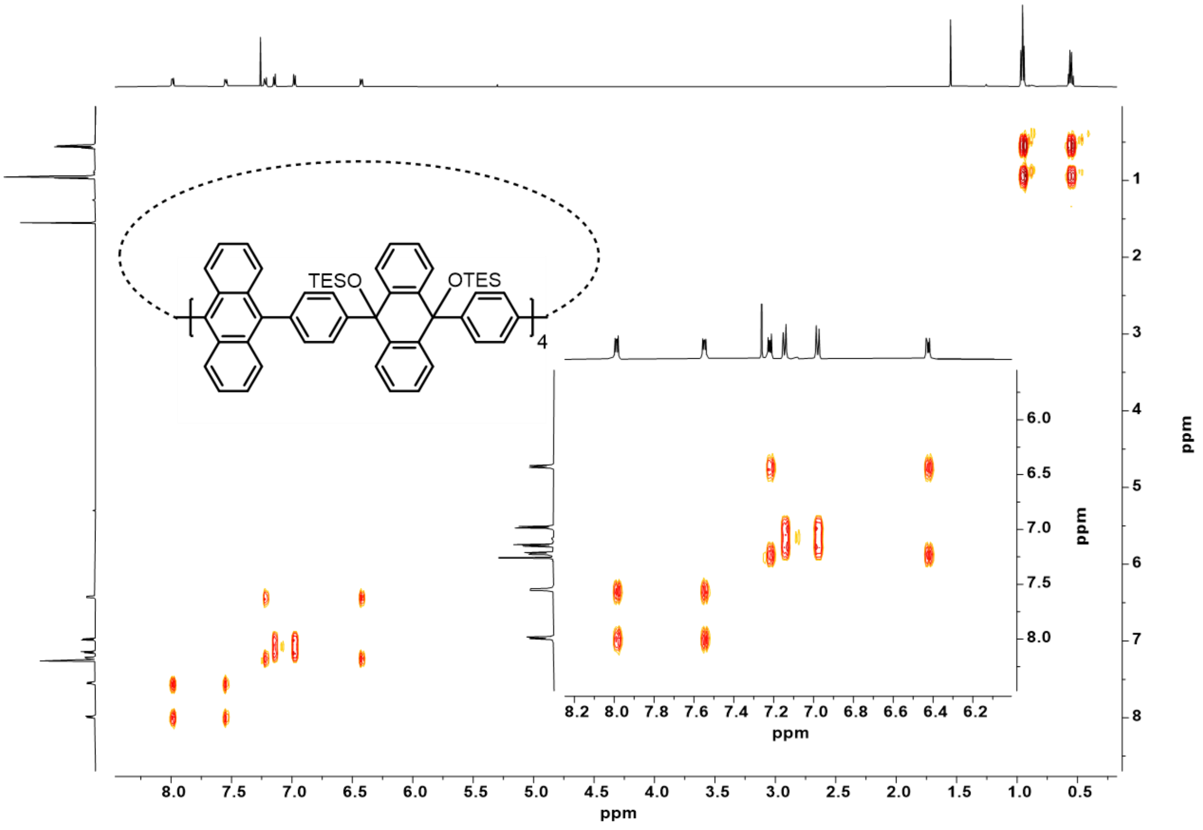

Figure S30: $^{1}H,^{1}H$ COSY spectrum of **[8.8]CAPP-OTES** ($CDCl_3$, 600 MHz, 300 K).

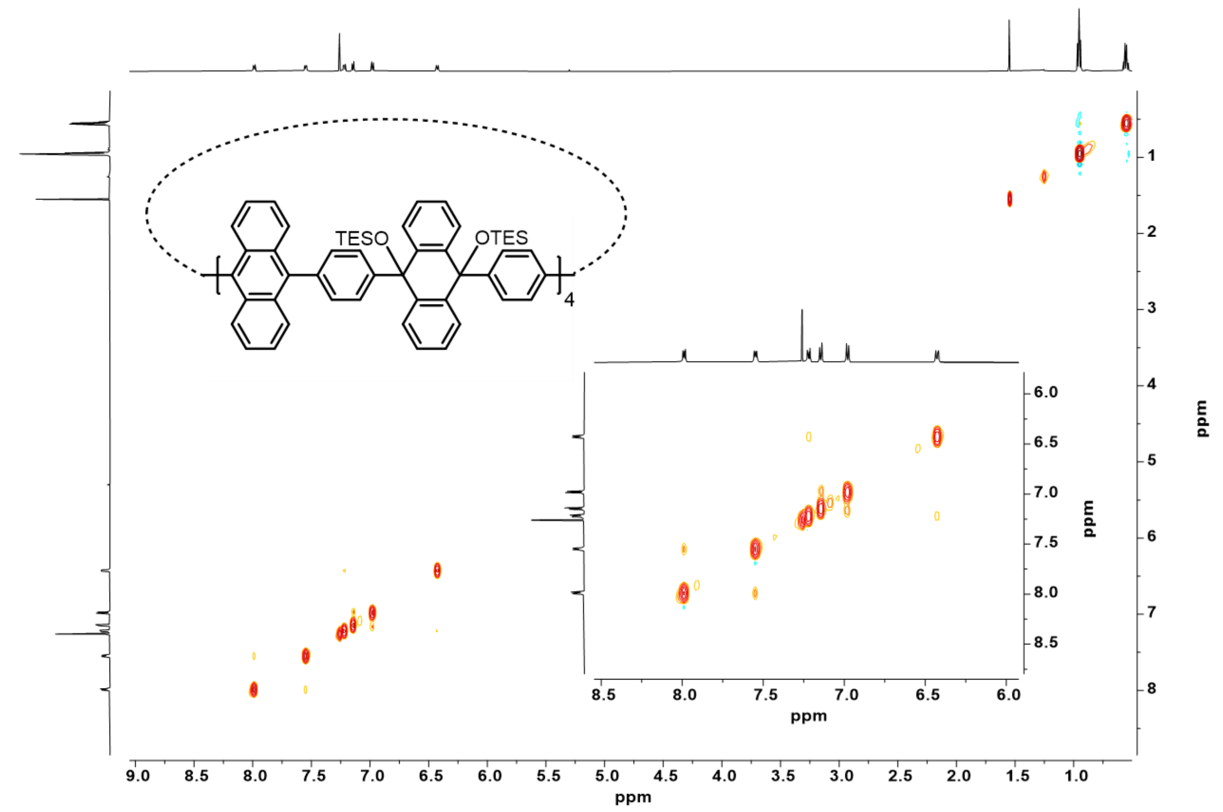

Figure S31: $^{1}H,^{1}H$ NOESY spectrum of **[8.8]CAPP-OTES** ($CDCl_3$, 600 MHz, 300 K).

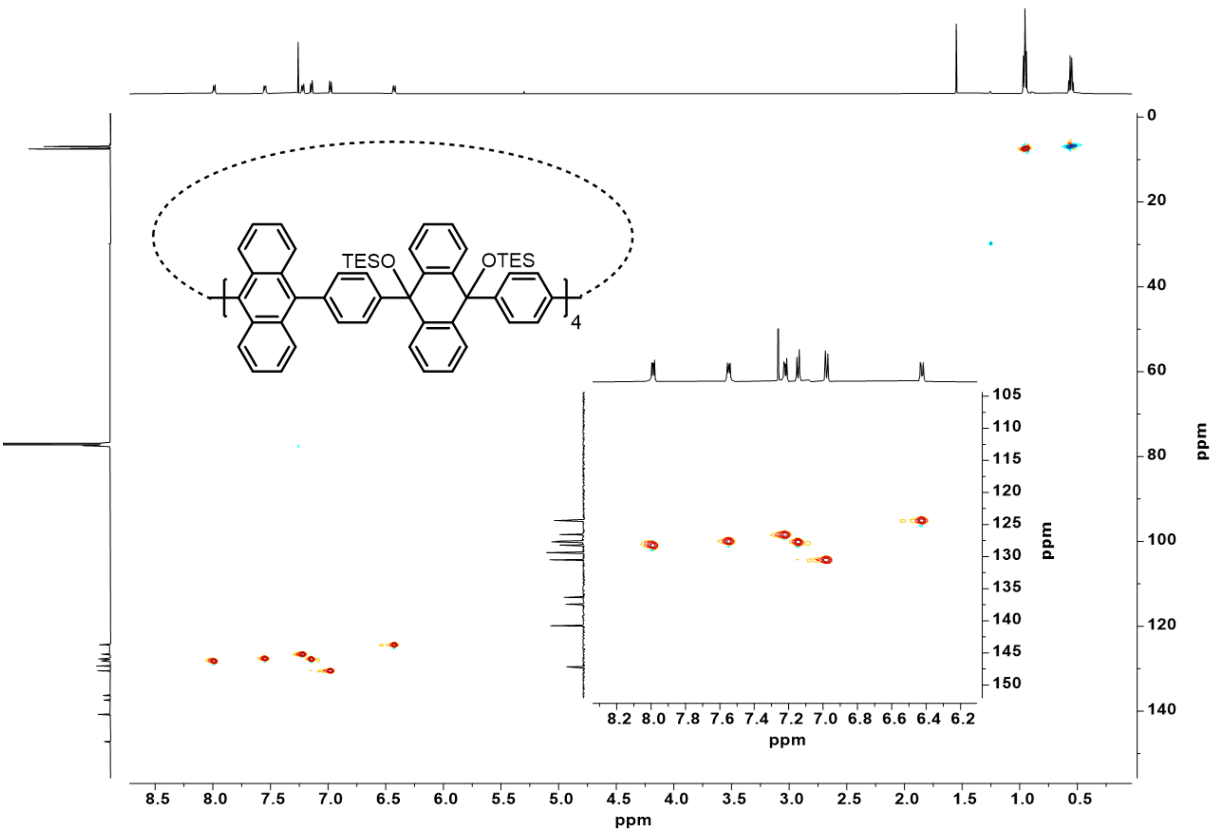

Figure S32: $^{1}H,^{13}C$ HSQC spectrum of **[8.8]CAPP-OTES** ($CDCl_3$, 600 MHz, 151 MHz 300 K).

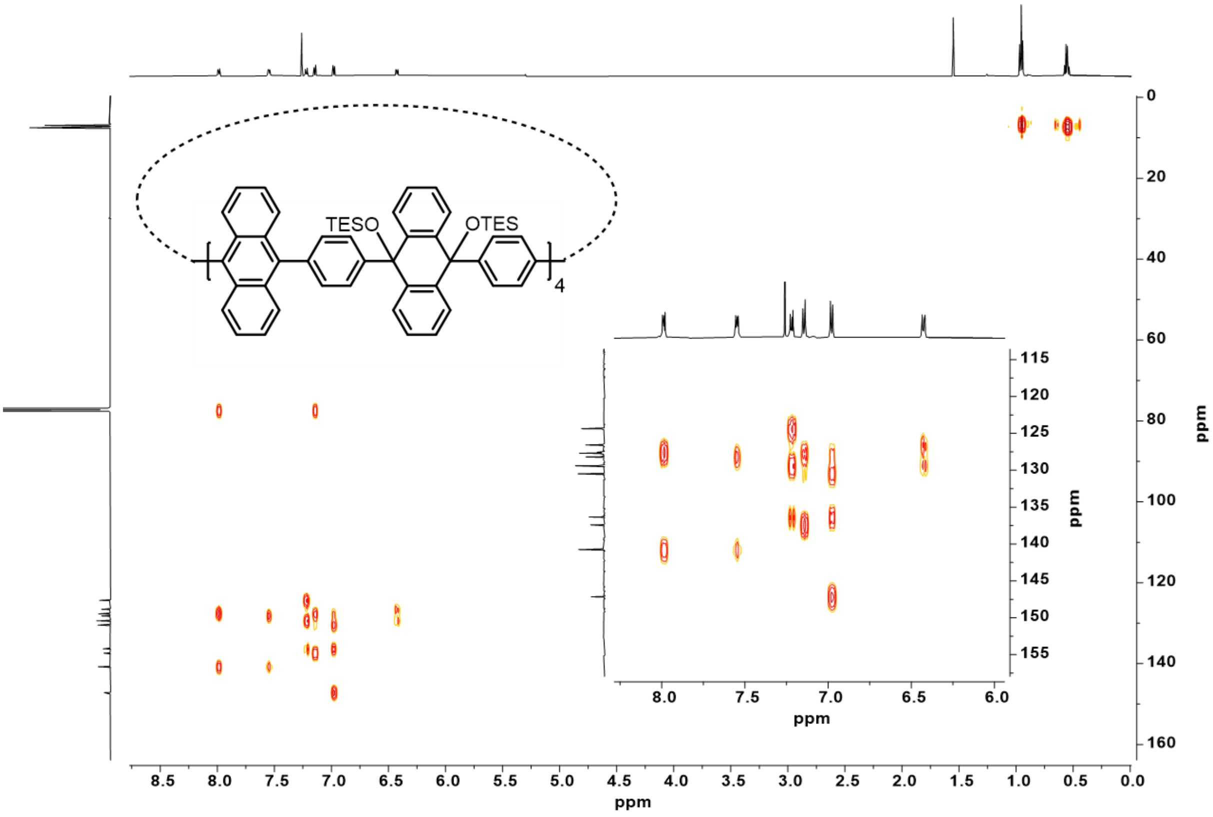

Figure S33: $^{1}H,^{13}C$ HMBC spectrum of **[8.8]CAPP-OTES** ($CDCl_3$, 600 MHz, 151 MHz 300 K).

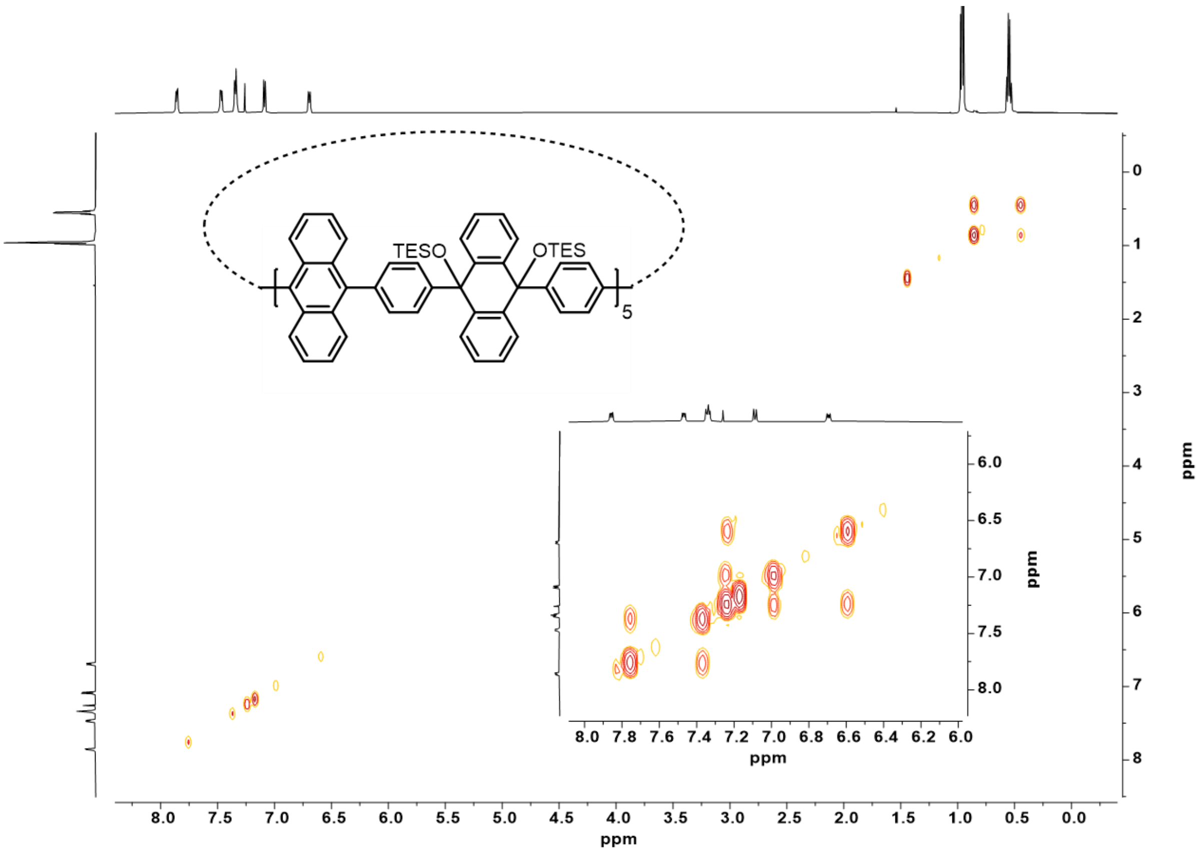

Figure S34: $^{1}H,^{1}H$ COSY spectrum of **[10.10]CAPP-OTES** ($CDCl_3$, 700 MHz, 300 K).

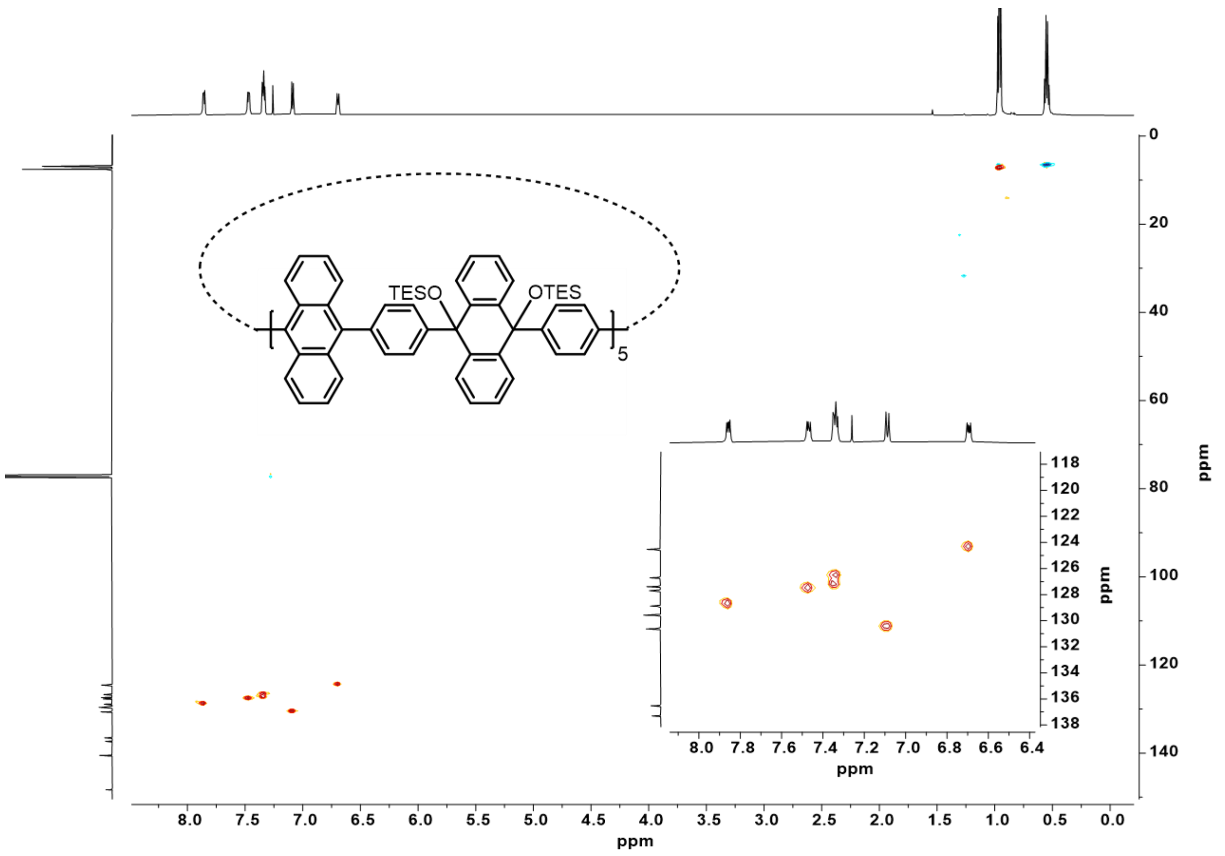

Figure S35: $^{1}H,^{13}C$ HSQC spectrum of **[10.10]CAPP-OTES** ($CDCl_3$, 700 MHz, 176 MHz 300 K).

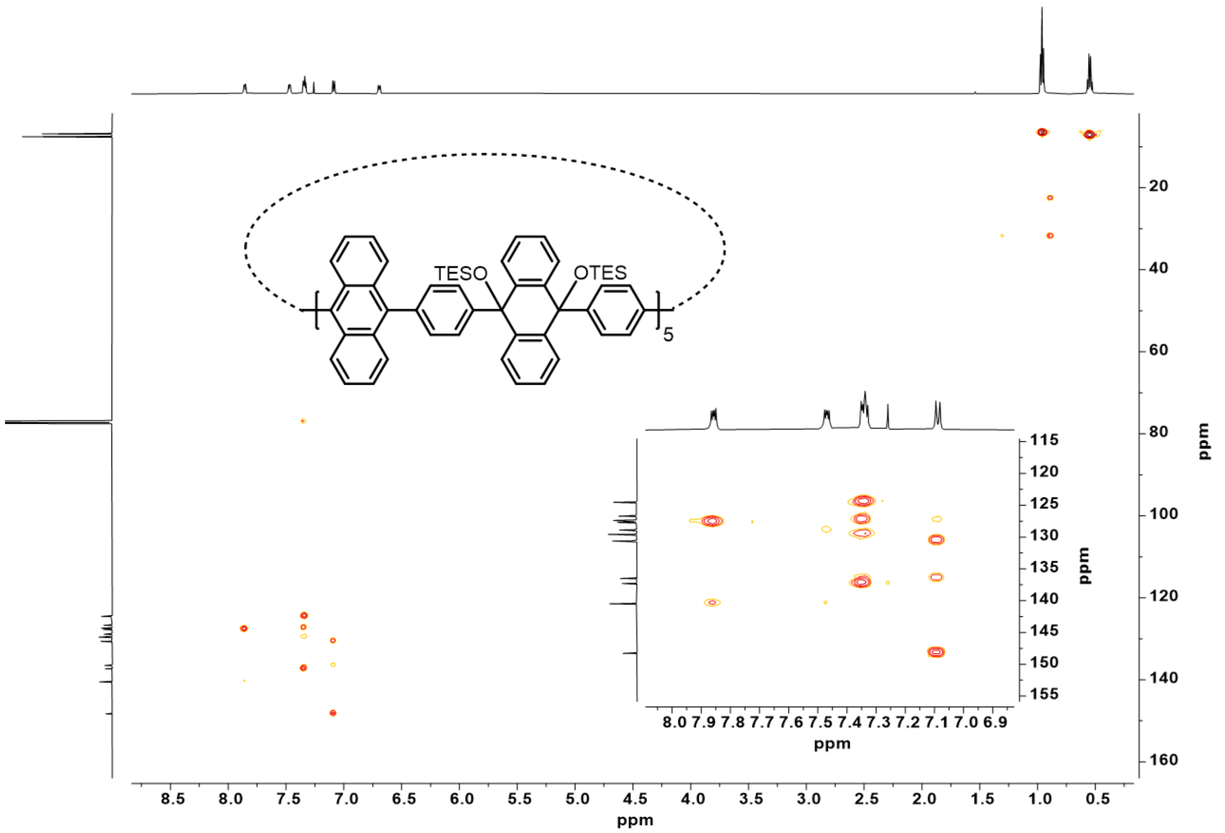

Figure S36: $^{1}H,^{13}C$ HMBC spectrum of **[10.10]CAPP-OTES** ($CDCl_3$, 700 MHz, 176 MHz 300 K).

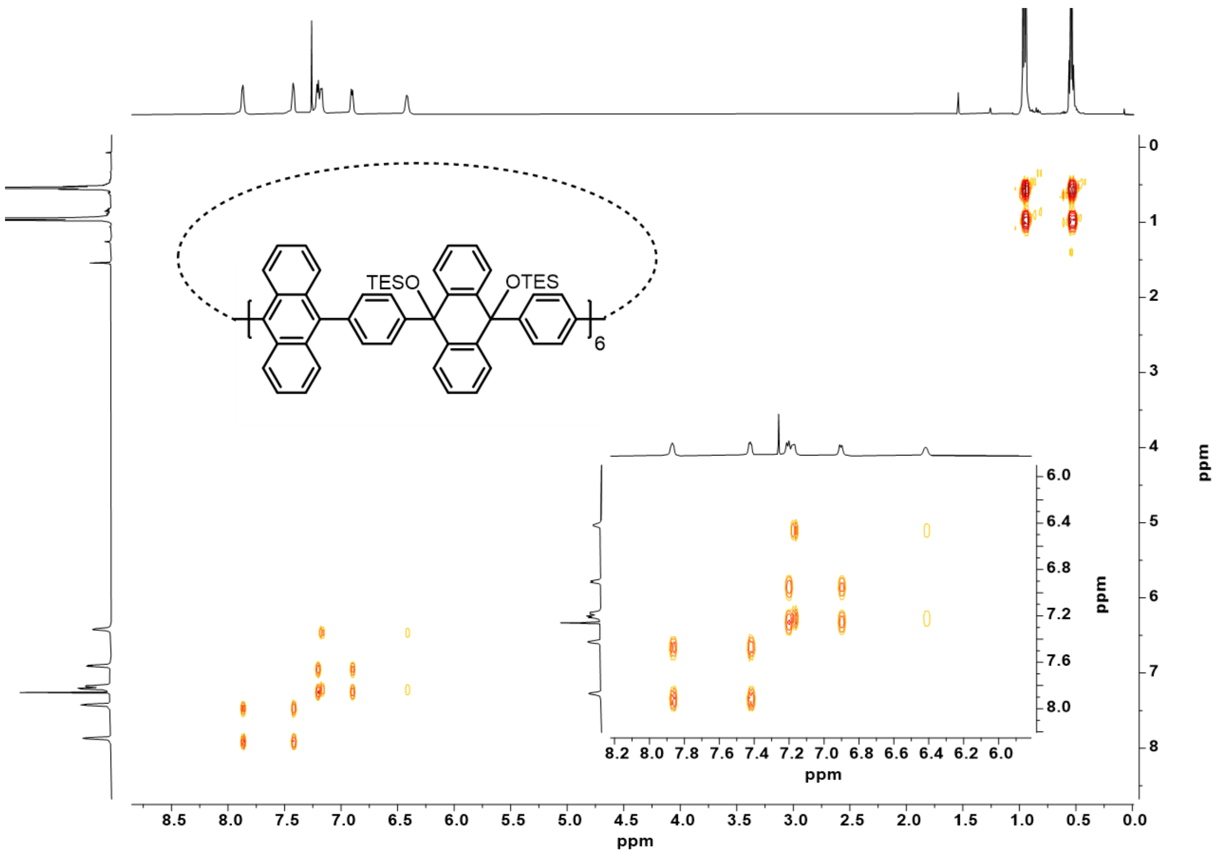

Figure S37: ${}^{1}H,{}^{1}H$ COSY spectrum of **[12.12]CAPP-OTES** ($CDCl_3$, 600 MHz, 300 K).

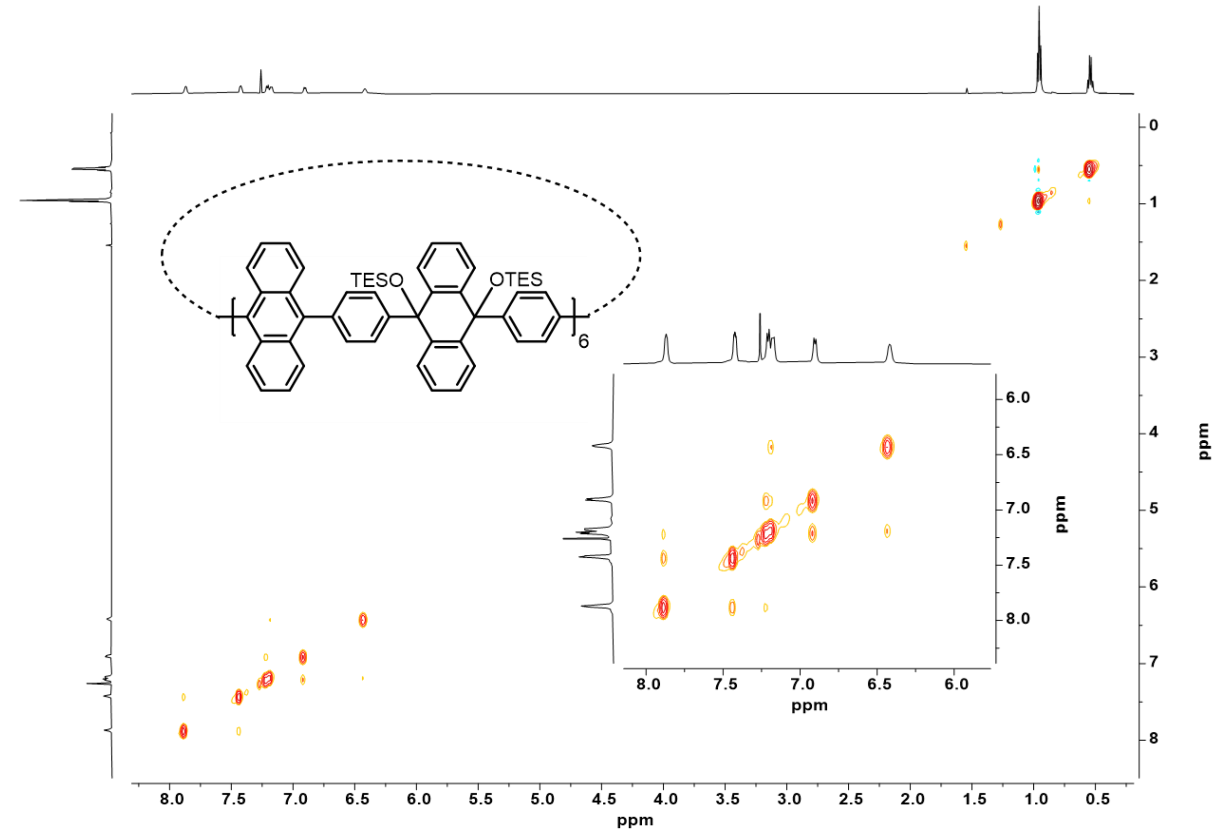

Figure S38: ${}^{1}H,{}^{1}H$ NOESY spectrum of **[12.12]CAPP-OTES** ($CDCl_3$, 600 MHz, 300 K).

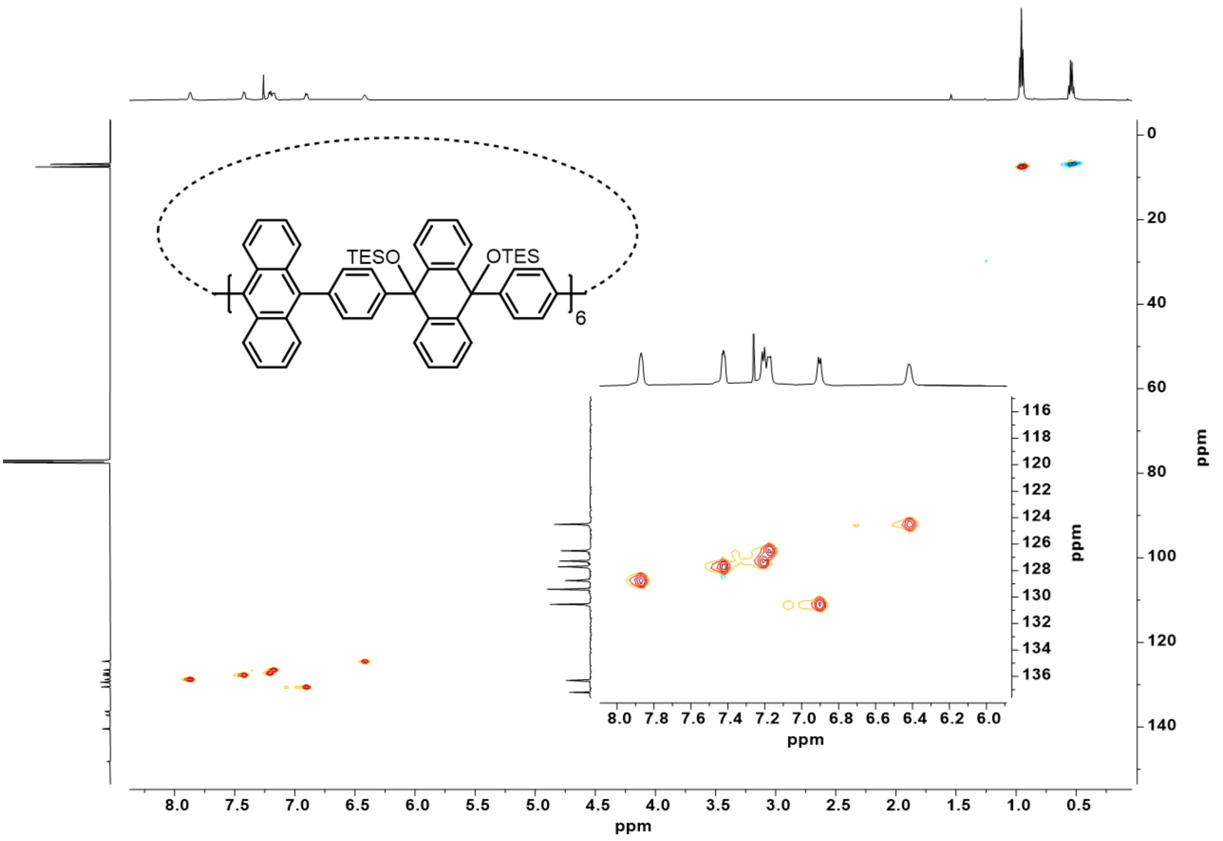

Figure S39: $^{1}H,^{13}C$ HSQC spectrum of **[12.12]CAPP-OTES** ($CDCl_3$, 600 MHz, 151 MHz 300 K).

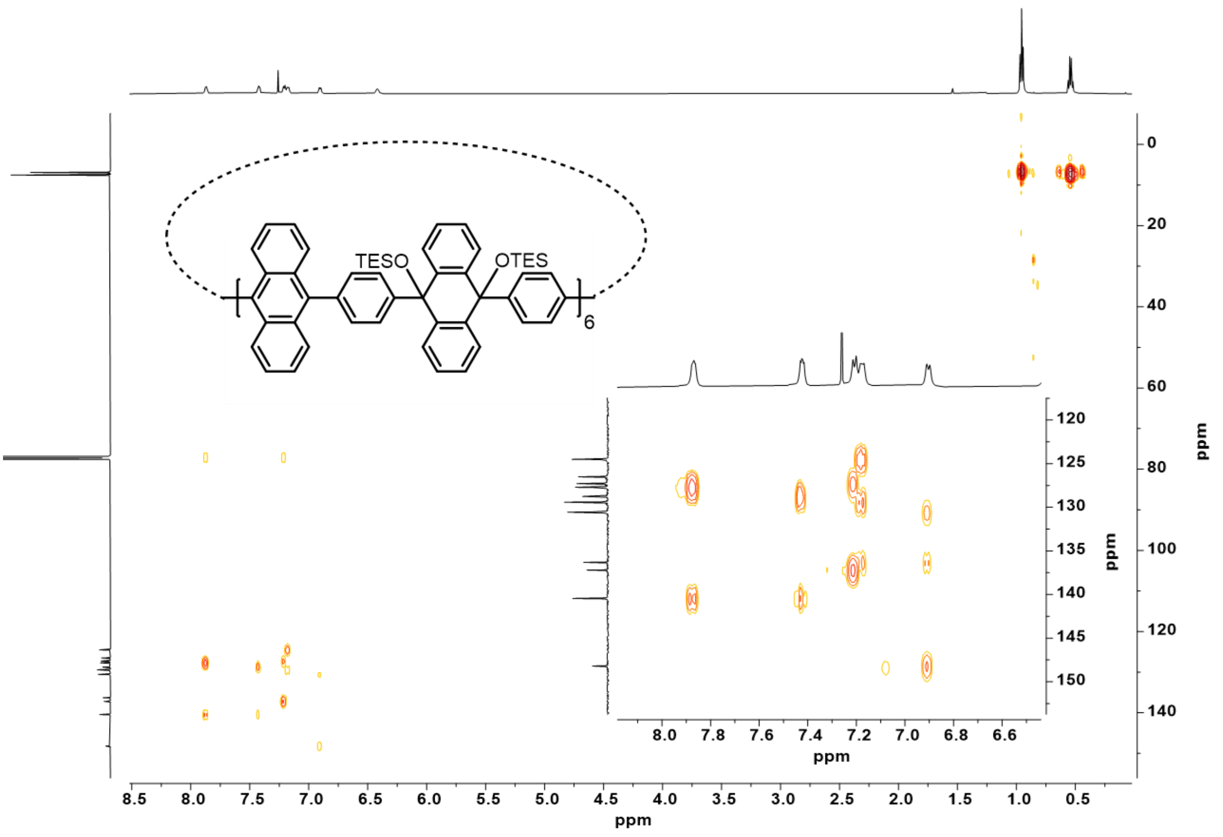

Figure S40: $^{1}H,^{13}C$ HMBC spectrum of **[12.12]CAPP-OTES** ($CDCl_3$, 600 MHz, 151 MHz 300 K).

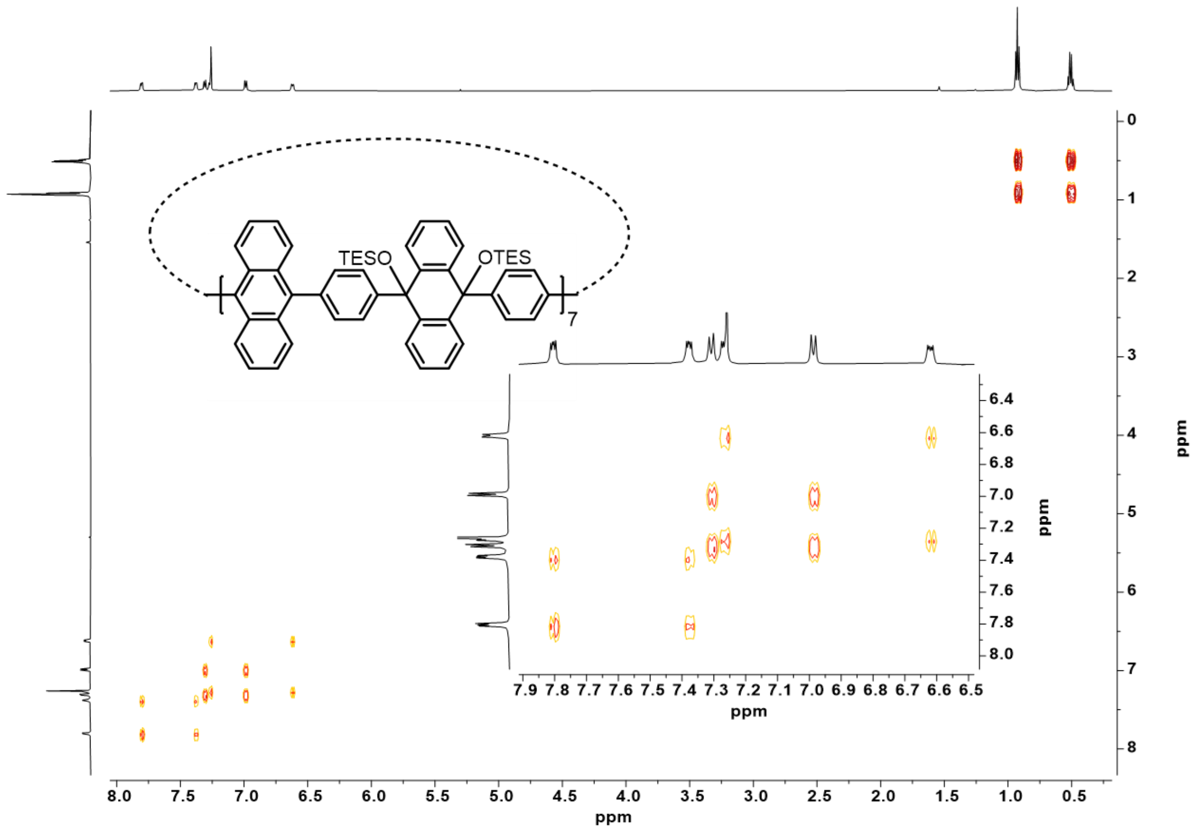

Figure S41: $^{1}H,^{1}H$ COSY spectrum of **[14.14]CAPP-OTES** ($CDCl_3$, 600 MHz, 300 K).

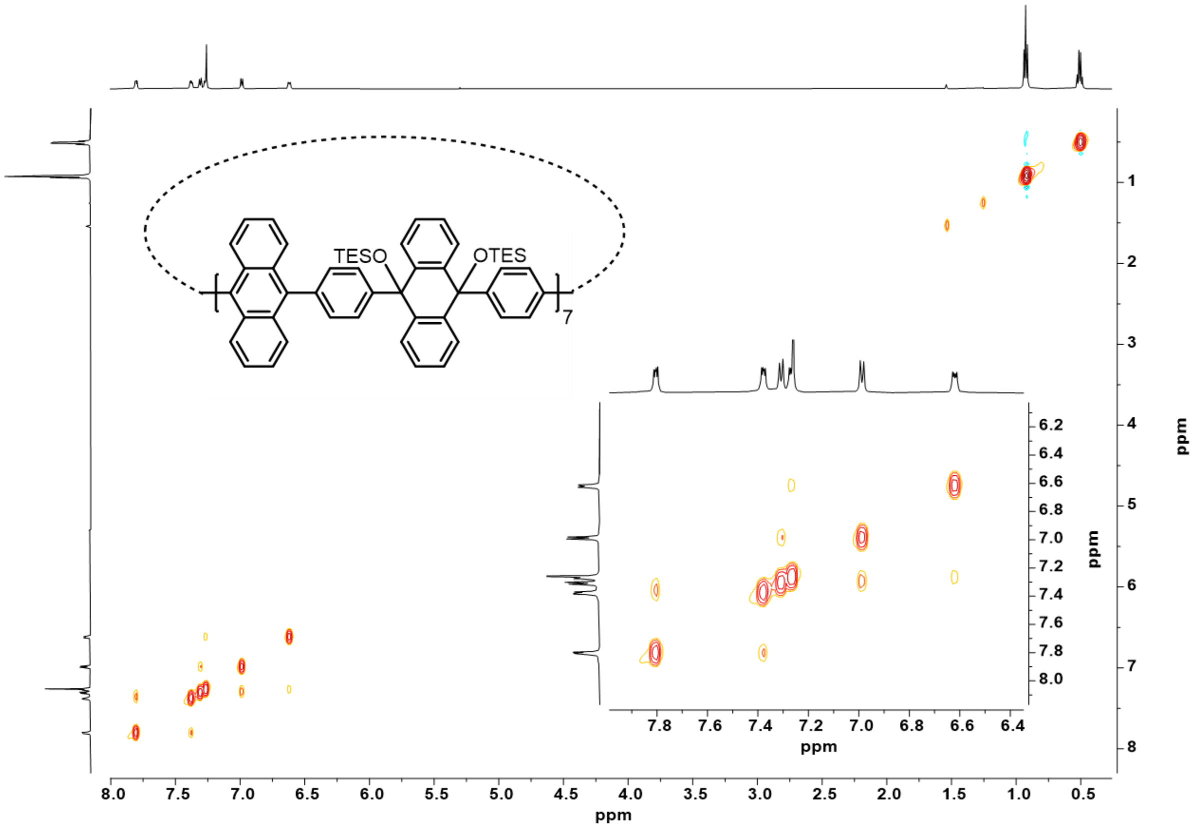

Figure S42: $^{1}H,^{1}H$ NOESY spectrum of **[14.14]CAPP-OTES** ($CDCl_3$, 600 MHz, 300 K).

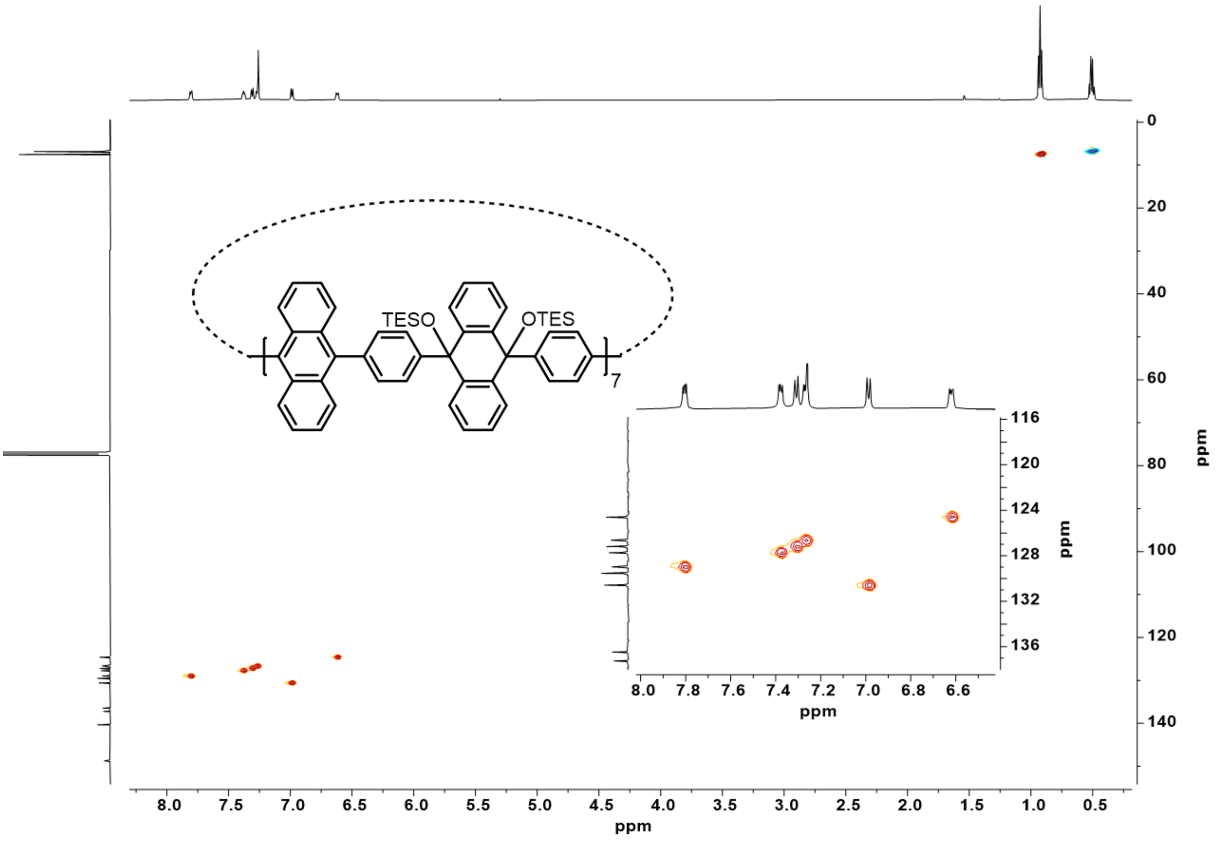

Figure S43: $^{1}H,^{13}C$ HSQC spectrum of **[14.14]CAPP-OTES** ($CDCl_3$, 600 MHz, 151 MHz 300 K).

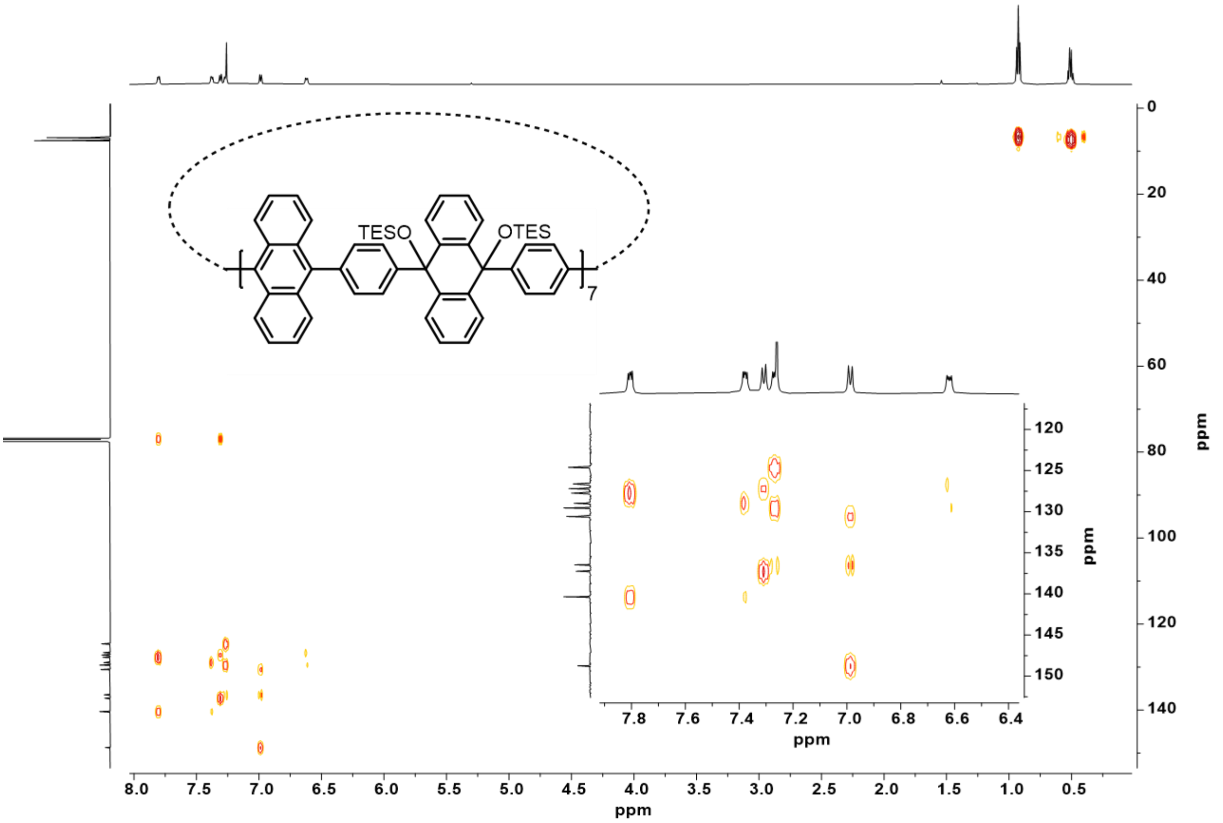

Figure S44: $^{1}H,^{13}C$ HMBC spectrum of **[14.14]CAPP-OTES** ($CDCl_3$, 600 MHz, 151 MHz 300 K).

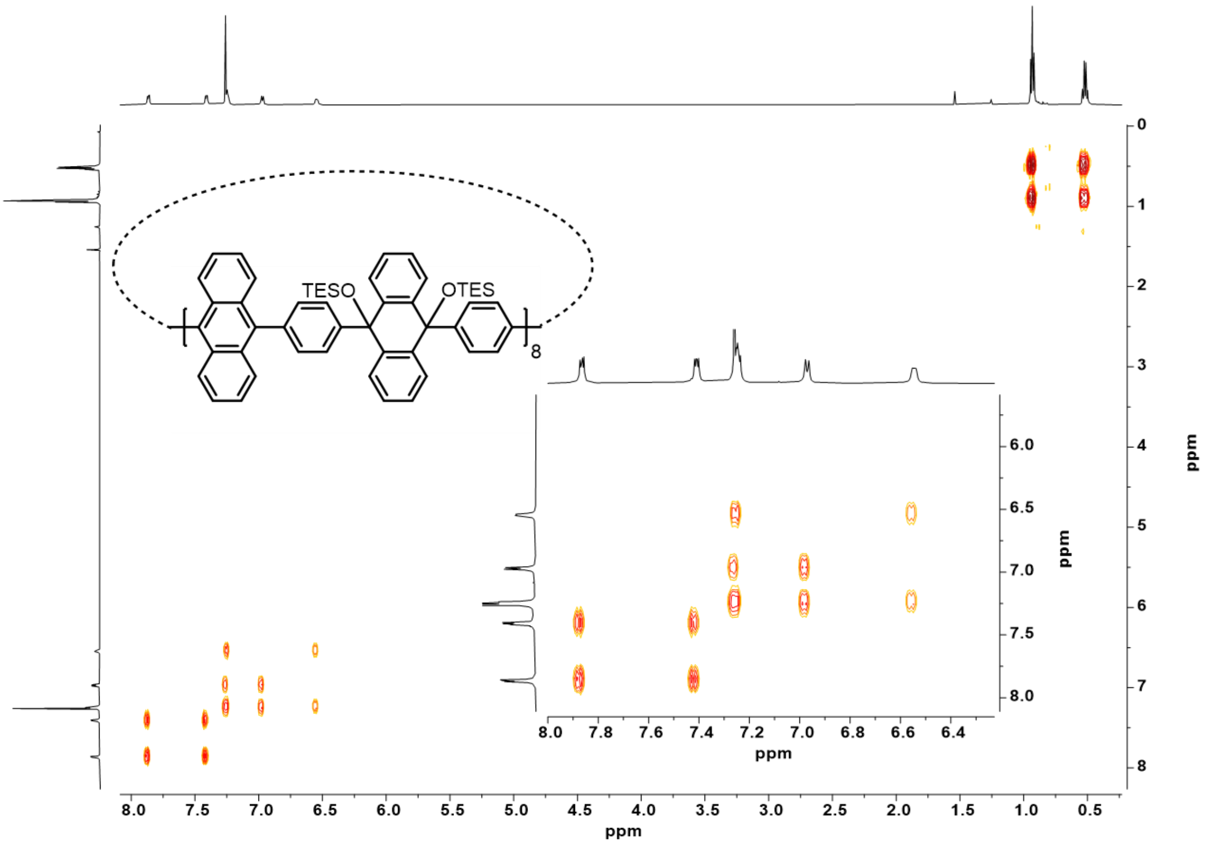

Figure S45: $^{1}H,^{1}H$ COSY spectrum of **[16.16]CAPP-OTES** ($CDCl_3$, 600 MHz, 300 K).

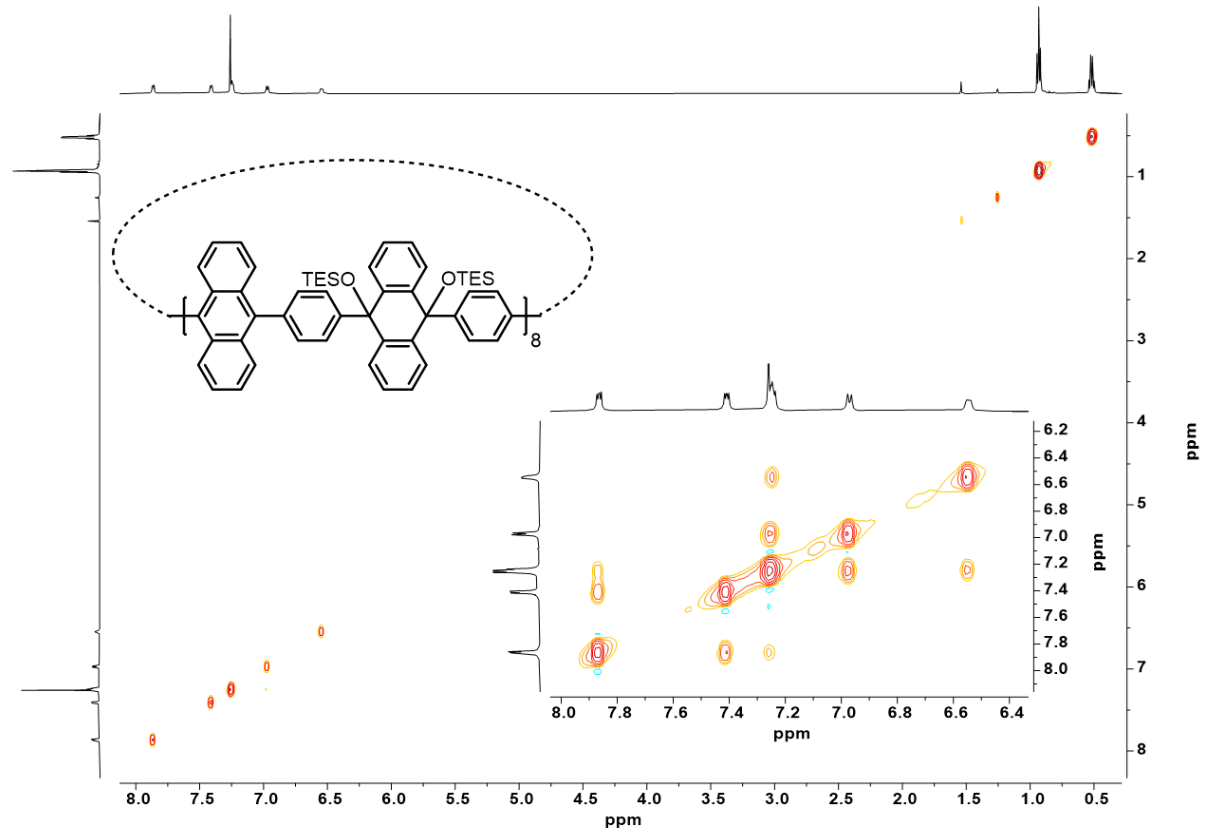

Figure S46: $^{1}H,^{1}H$ NOESY spectrum of **[16.16]CAPP-OTES** ($CDCl_3$, 600 MHz, 300 K).

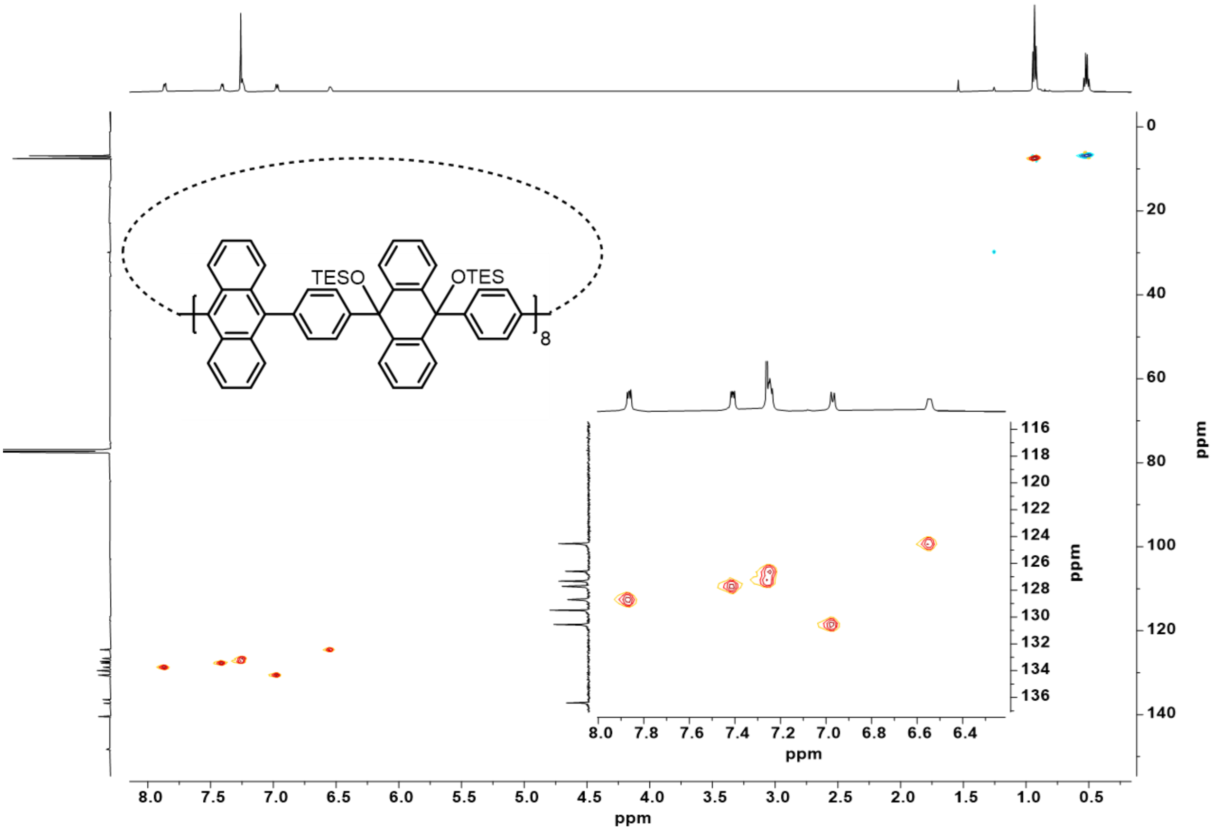

Figure S47: $^{1}H,^{13}C$ HSQC spectrum of **[16.16]CAPP-OTES** ($CDCl_3$, 600 MHz, 151 MHz 300 K).

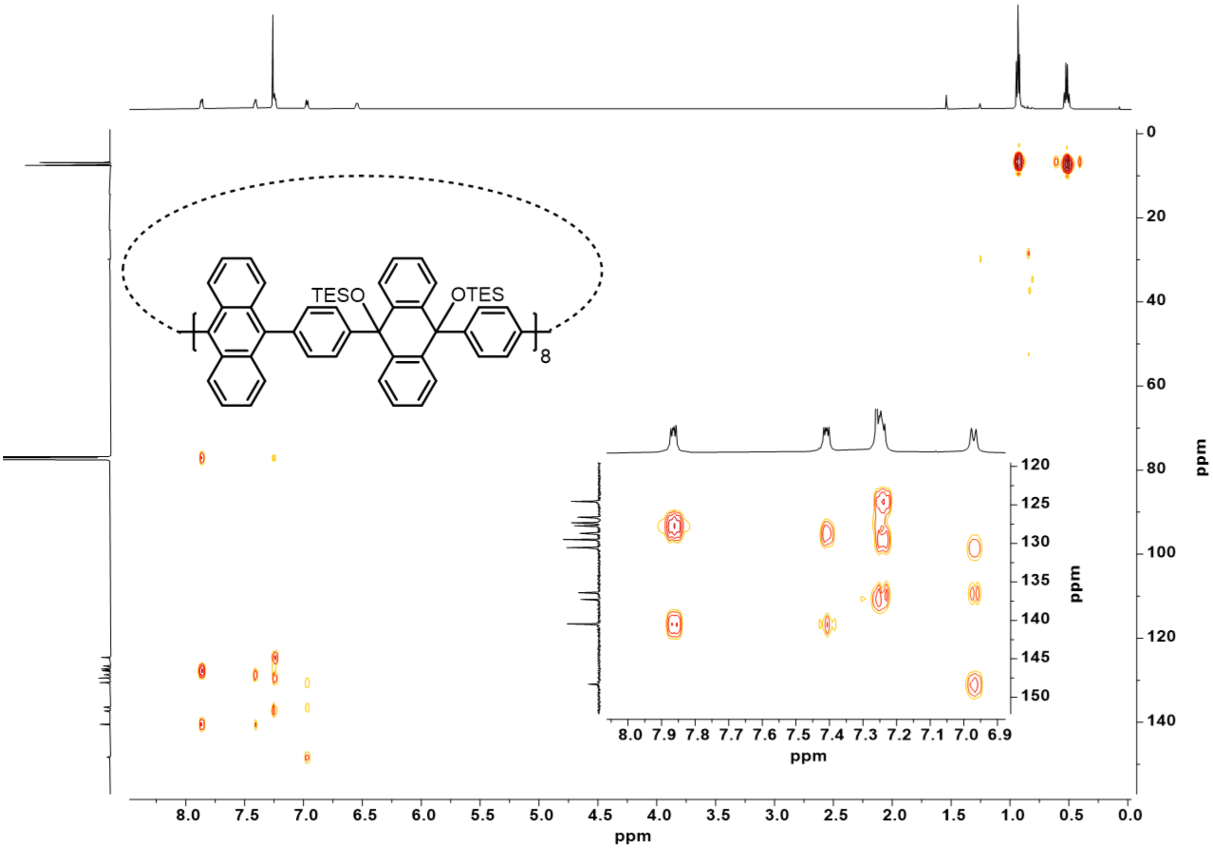

Figure S48: $^{1}H,^{13}C$ HMBC spectrum of **[16.16]CAPP-OTES** ($CDCl_3$, 600 MHz, 151 MHz 300 K).

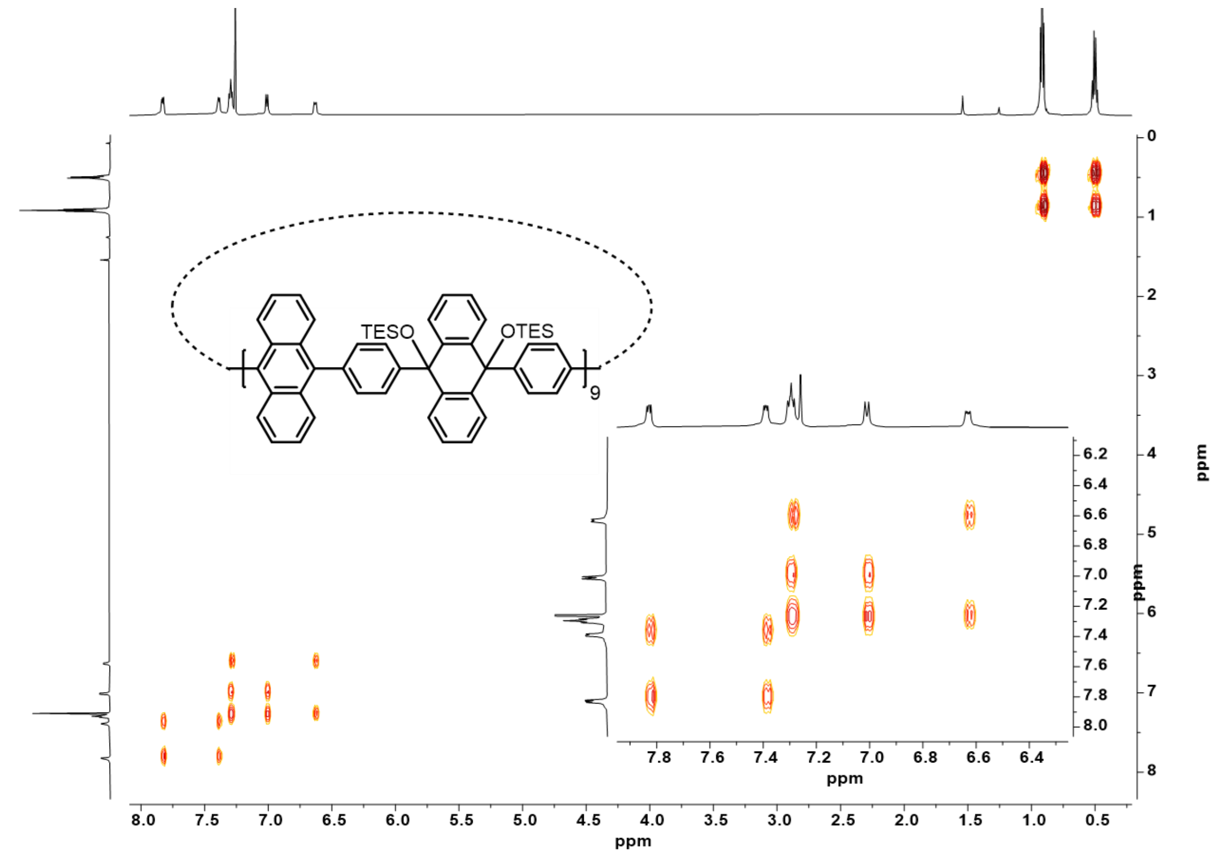

Figure S49: $^{1}H,^{1}H$ COSY spectrum of **[18.18]CAPP-OTES** ($CDCl_3$, 600 MHz, 300 K).

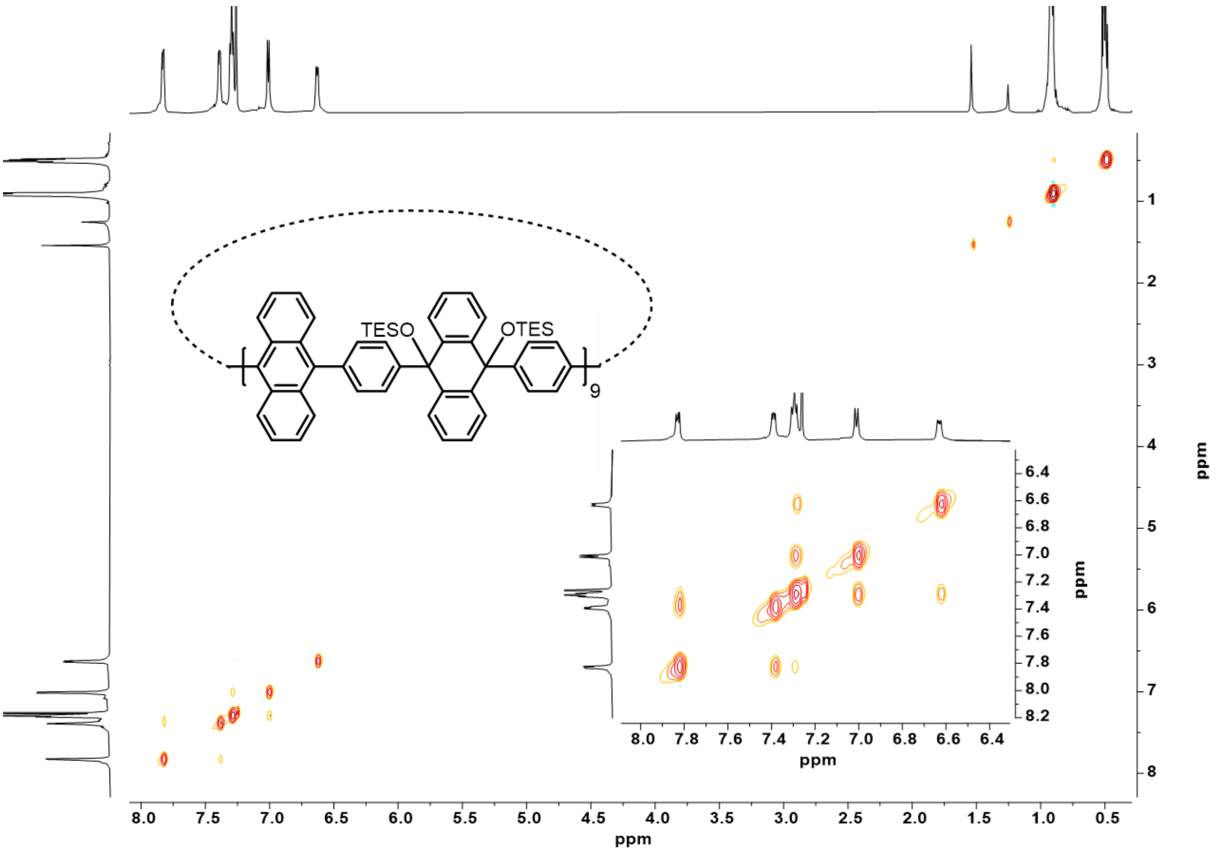

Figure S50: $^{1}H,^{1}H$ NOESY spectrum of **[18.18]CAPP-OTES** ($CDCl_3$, 600 MHz, 300 K).

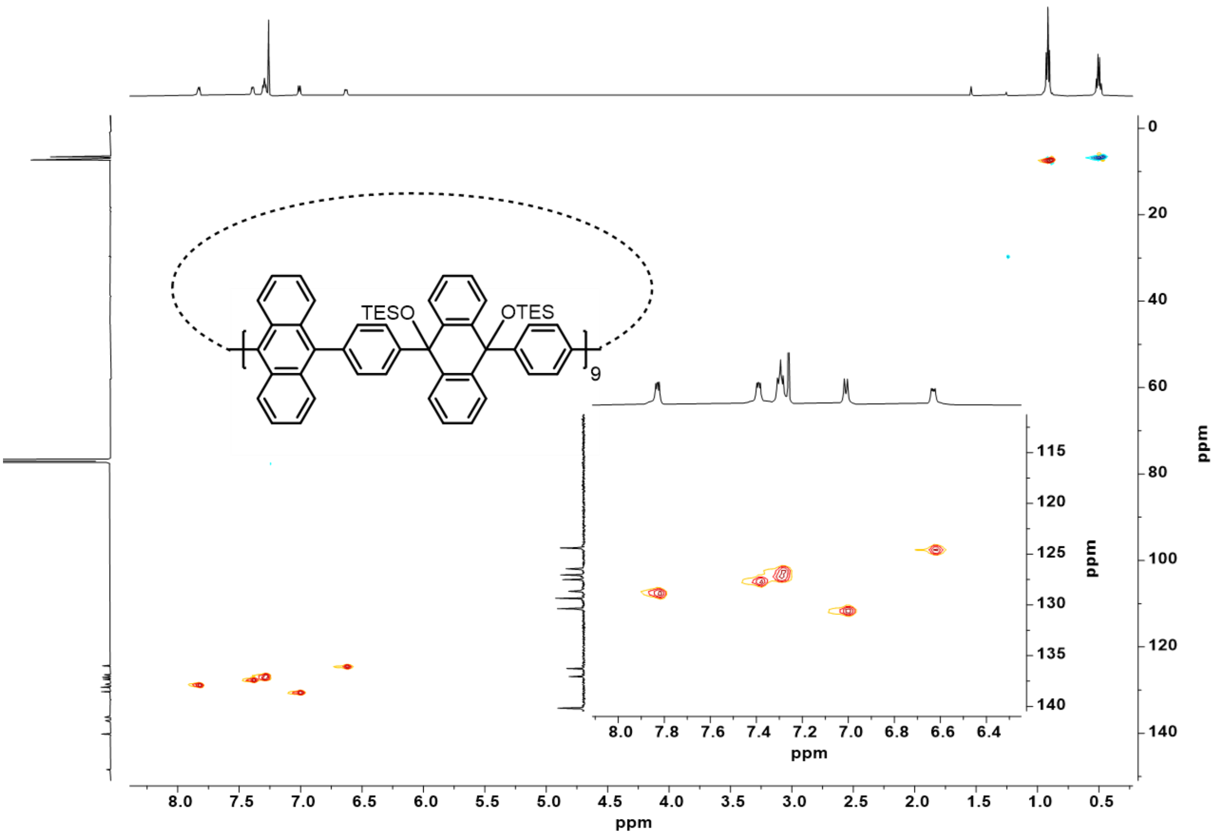

Figure S51: $^{1}H,^{13}C$ HSQC spectrum of **[18.18]CAPP-OTES** ($CDCl_3$, 600 MHz, 151 MHz 300 K).

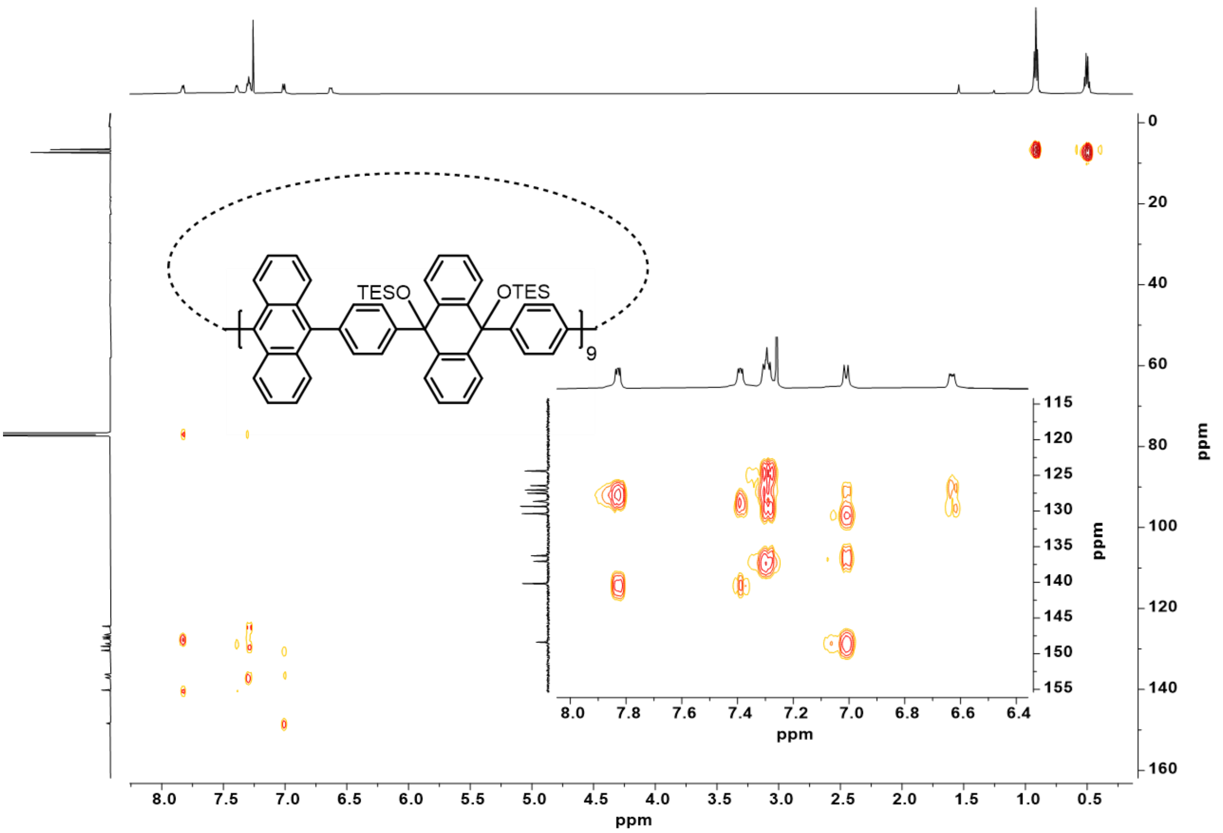

Figure S52: $^{1}H,^{13}C$ HMBC spectrum of **[18.18]CAPP-OTES** ($CDCl_3$, 600 MHz, 151 MHz 300 K).

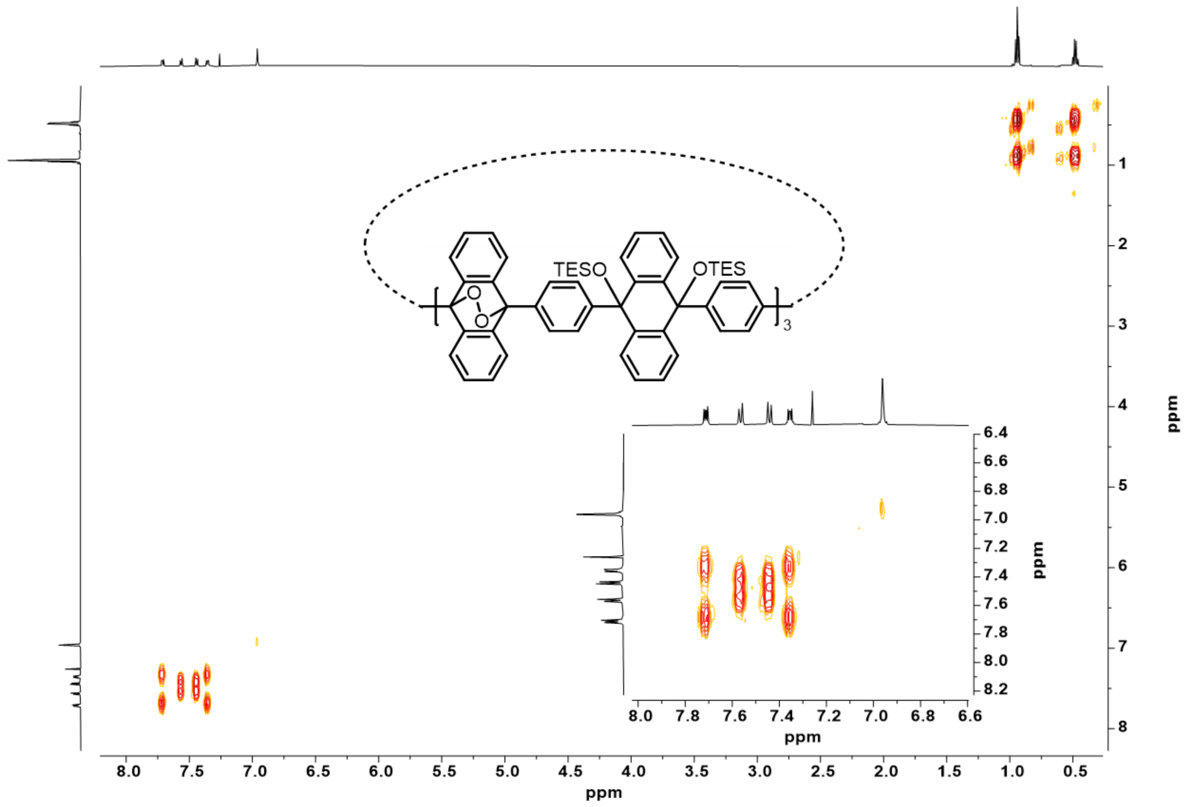

Figure S53: $^{1}H,^{1}H$ COSY spectrum of **[6.6]CAPP-OTES-O$_2$** ($CDCl_3$, 600 MHz, 300 K).

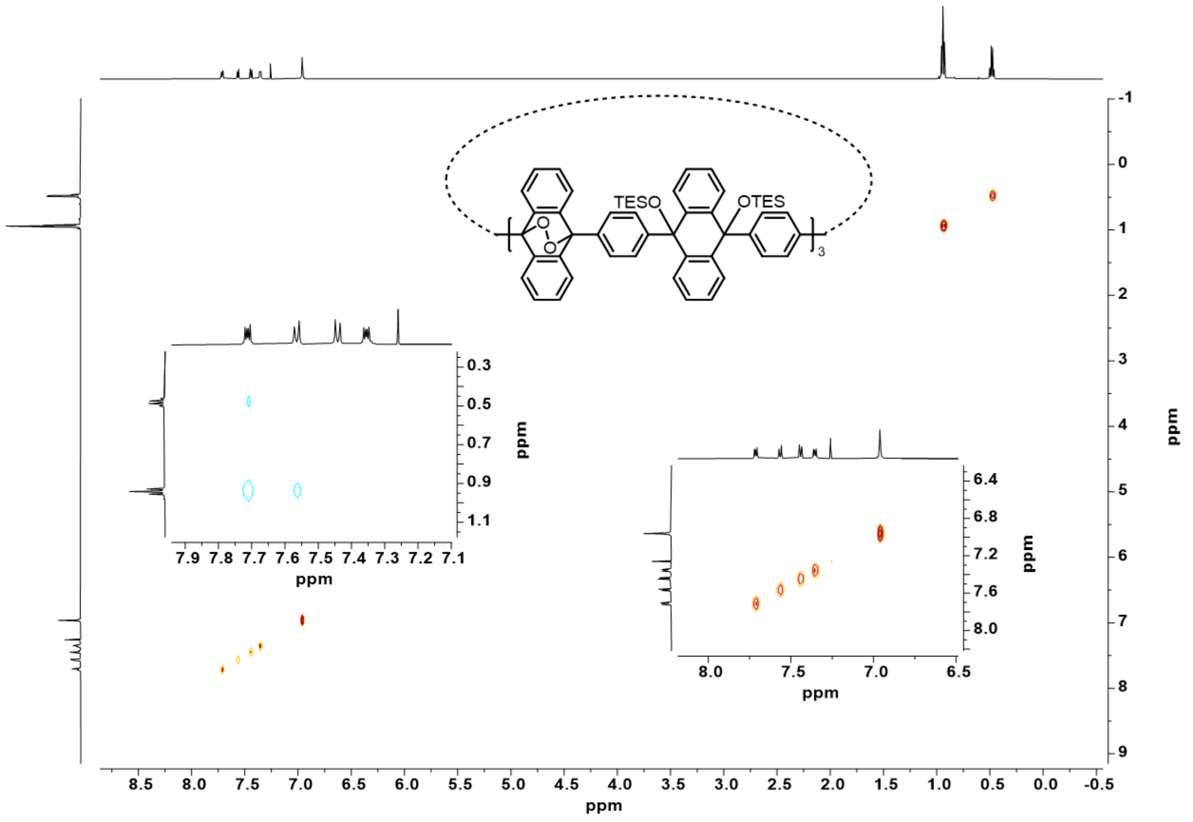

Figure S54: $^{1}H,^{1}H$ NOESY spectrum of **[6.6]CAPP-OTES-O$_2$** ($CDCl_3$, 600 MHz, 300 K).

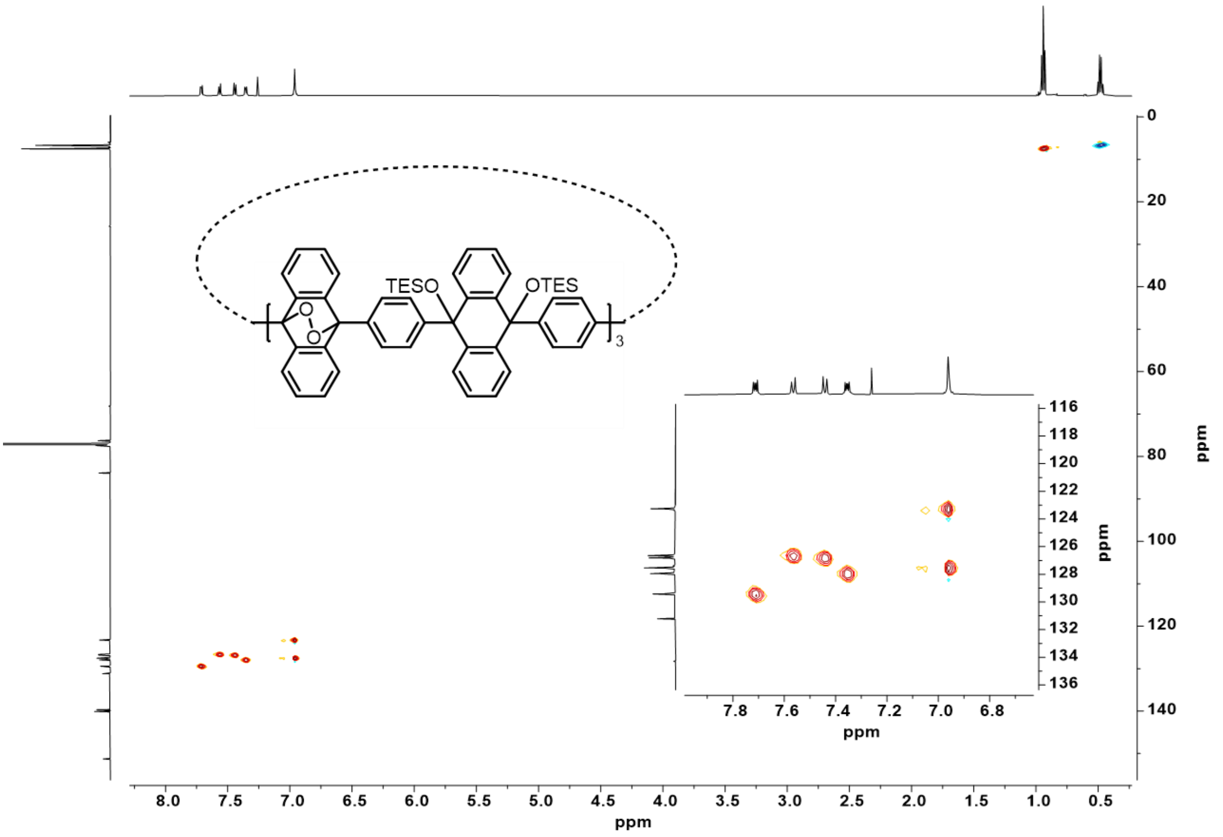

Figure S55: ^{1}H,^{13}C HSQC spectrum of **[6.6]CAPP-OTES-O$_2$** (CDCl$_3$, 600 MHz, 151 MHz 300 K).

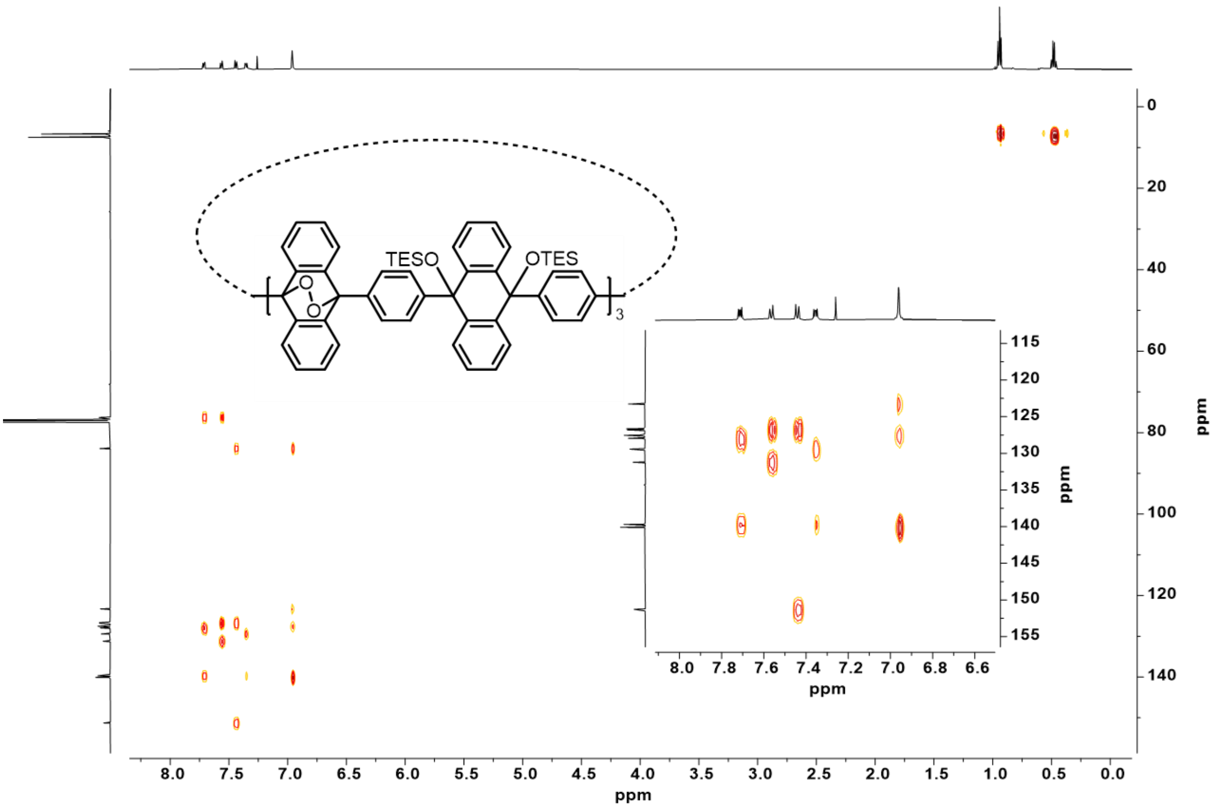

Figure S56: ^{1}H,^{13}C HMBC spectrum of **[6.6]CAPP-OTES-O$_2$** (CDCl$_3$, 600 MHz, 151 MHz 300 K).

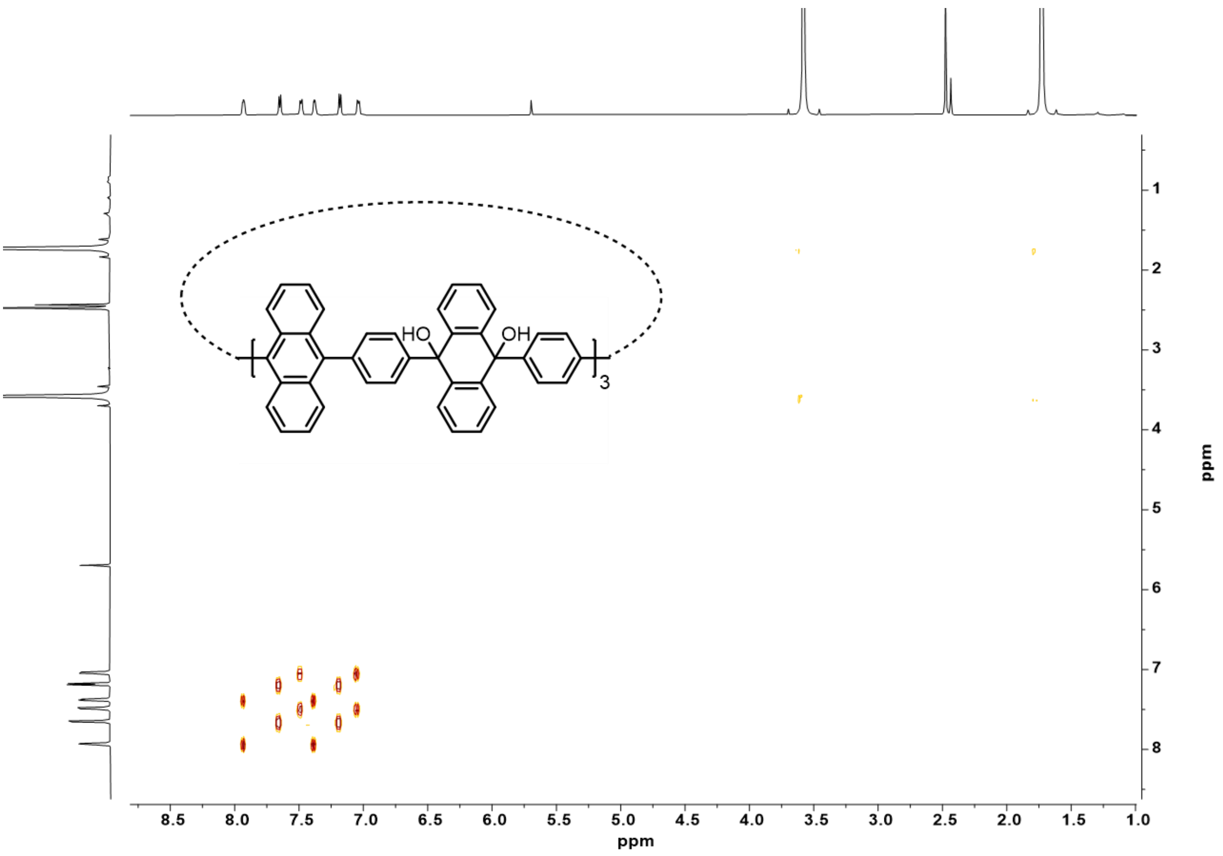

Figure S57: ^{1}H,^{1}H COSY spectrum of **[6.6]CAPP-OH** (THF-d$_8$, 600 MHz, 300 K).

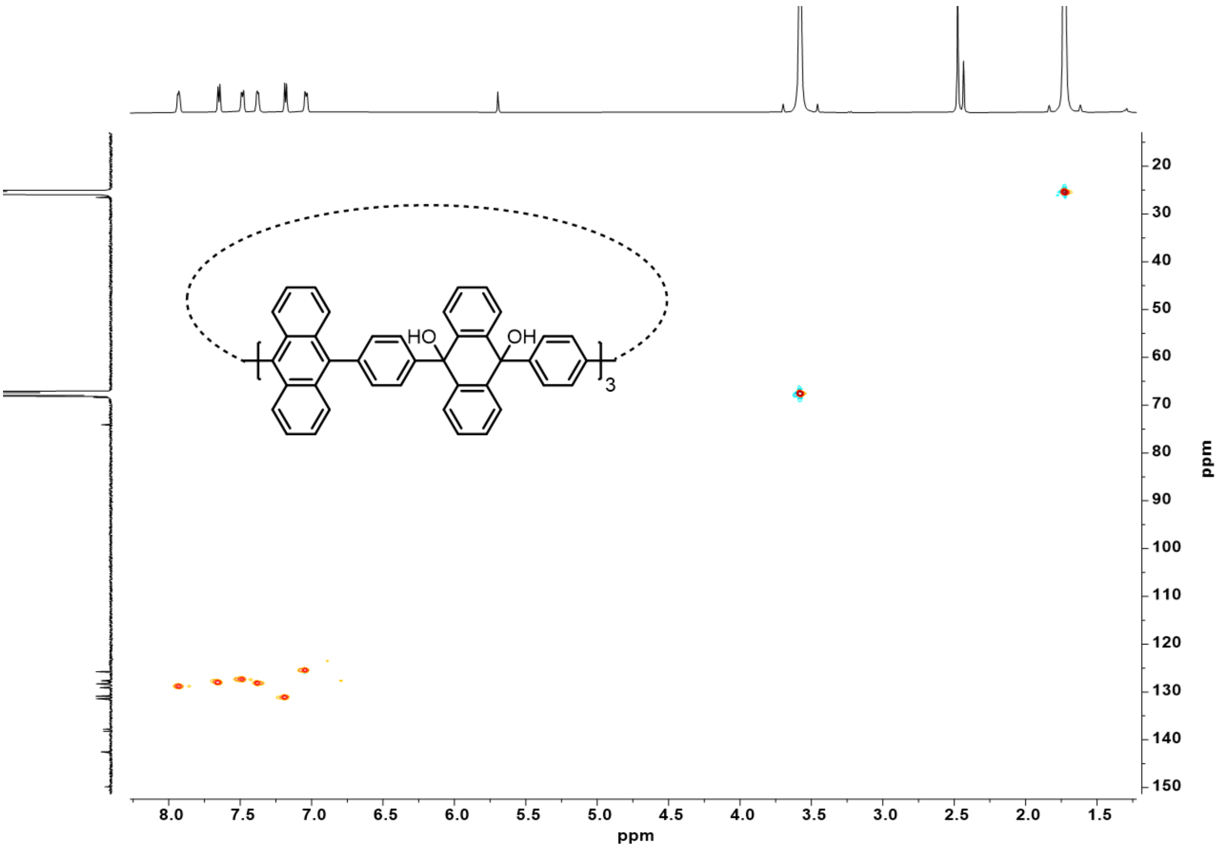

Figure S58: ^{1}H,^{13}C HSQC spectrum of **[6.6]CAPP-OH** (THF-d$_8$, 600 MHz, 151 MHz 300 K).

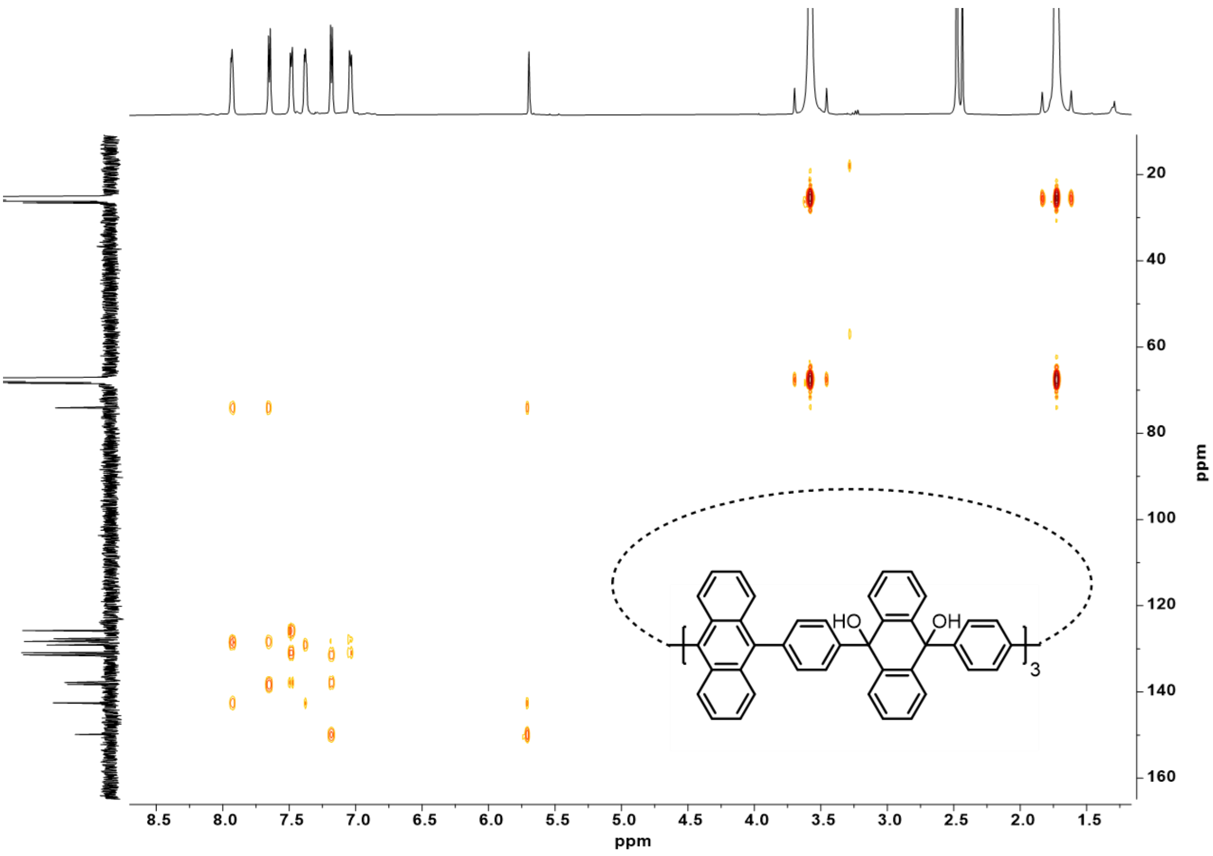

Figure S59: $^{1}H,^{13}C$ HMBC spectrum of **[6.6]CAPP-OH** (THF-d_8, 600 MHz, 151 MHz 300 K).

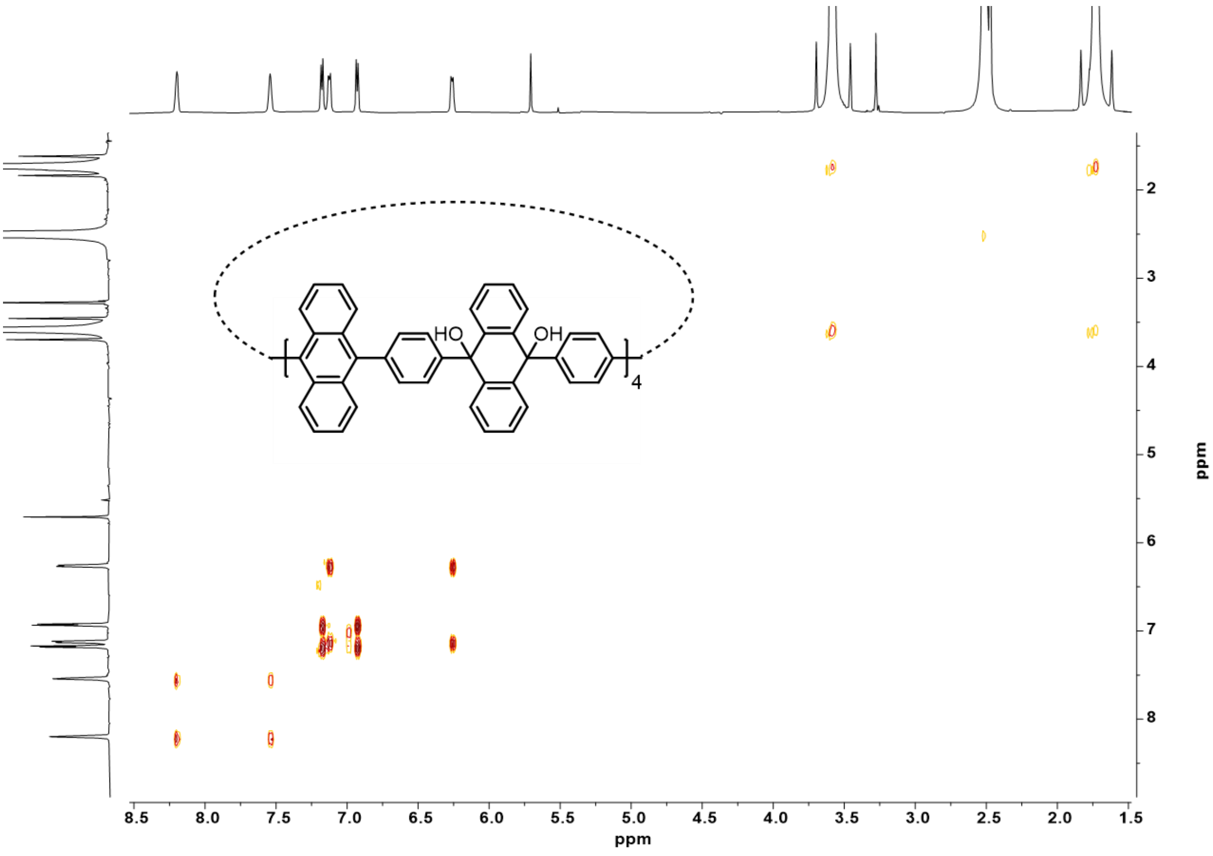

Figure S60: $^{1}H,^{1}H$ COSY spectrum of **[8.8]CAPP-OH** (THF-d_8, 600 MHz, 300 K).

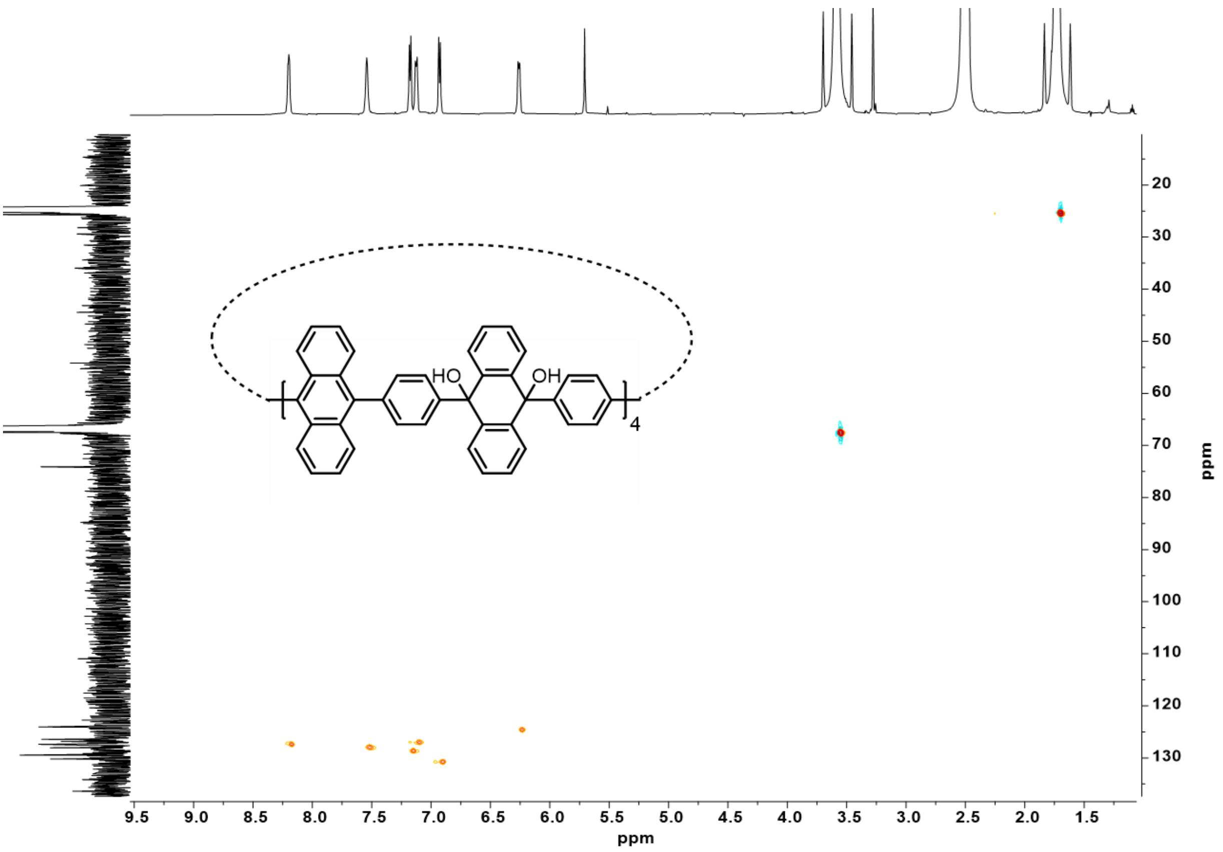

Figure S61: ^{1}H,^{13}C HSQC spectrum of **[8.8]CAPP-OH** (THF-d_8, 600 MHz, 151 MHz 300 K).

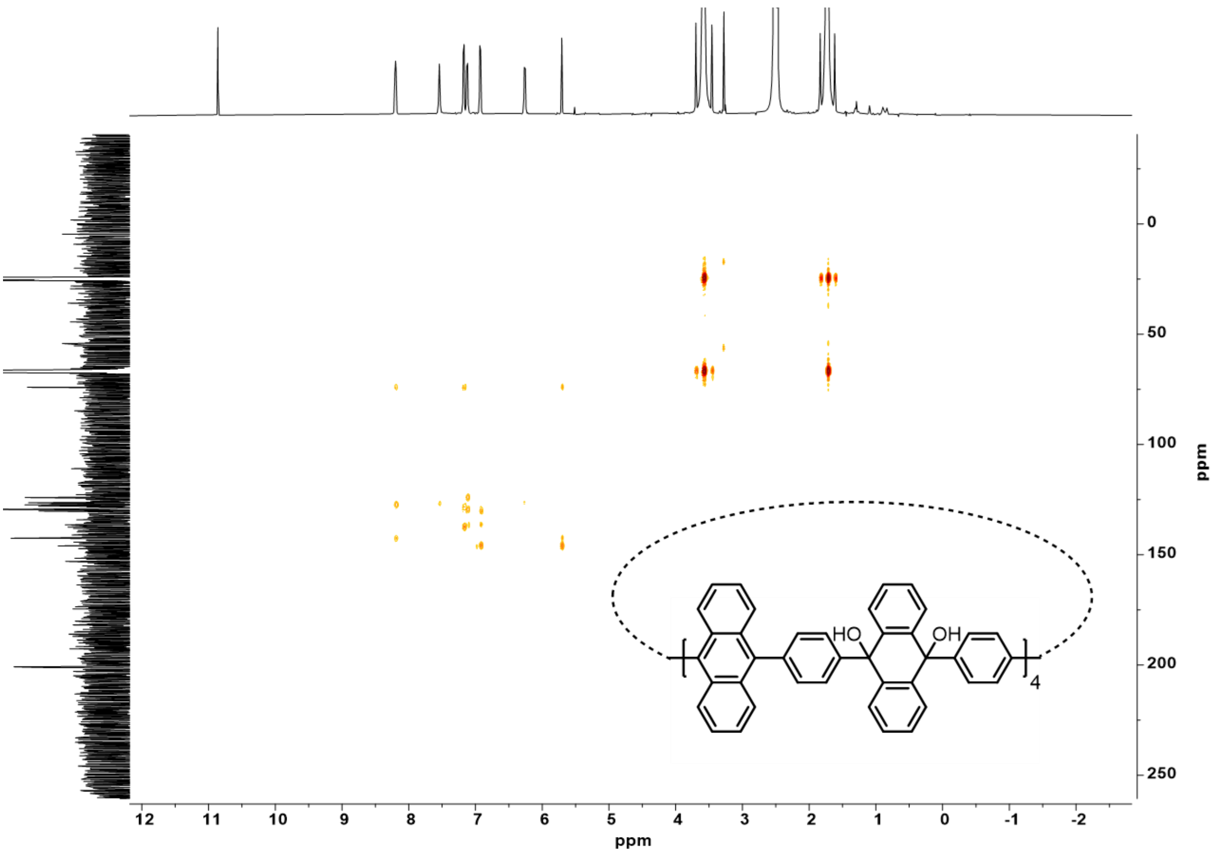

Figure S62: ^{1}H,^{13}C HMBC spectrum of **[8.8]CAPP-OH** (THF-d_8, 600 MHz, 151 MHz 300 K).

3.3 IR spectra

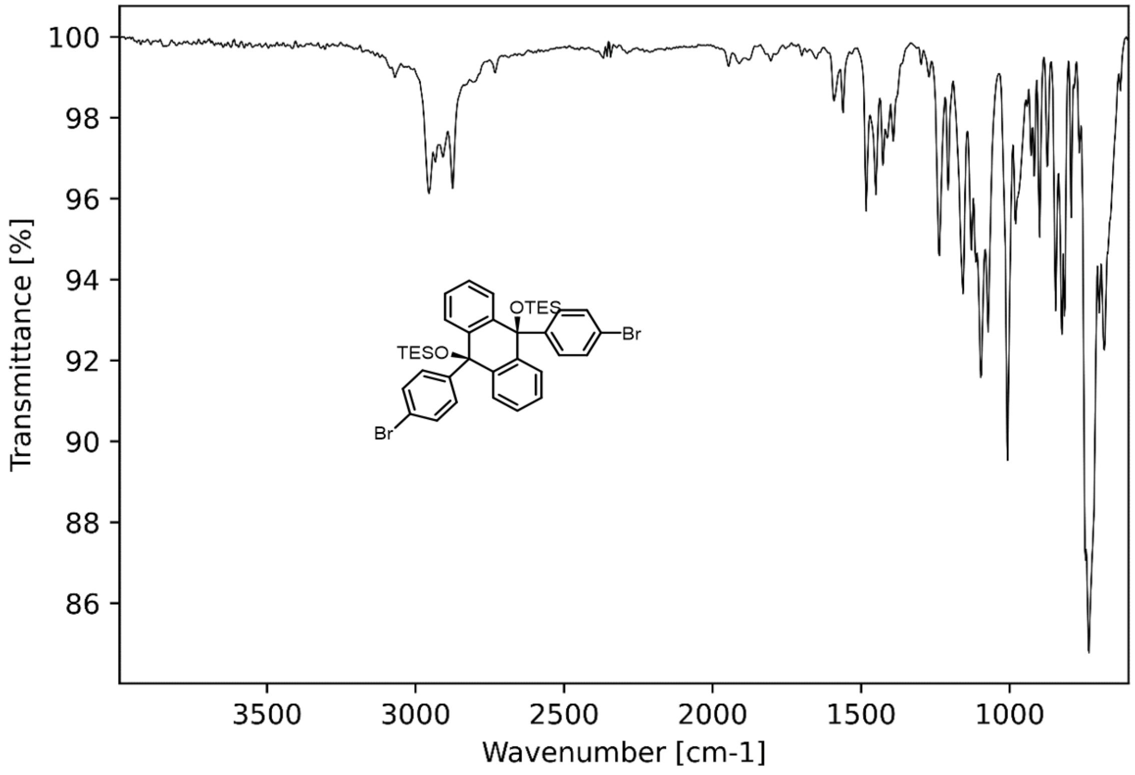

Figure S63: IR spectrum of **4** (ATR).

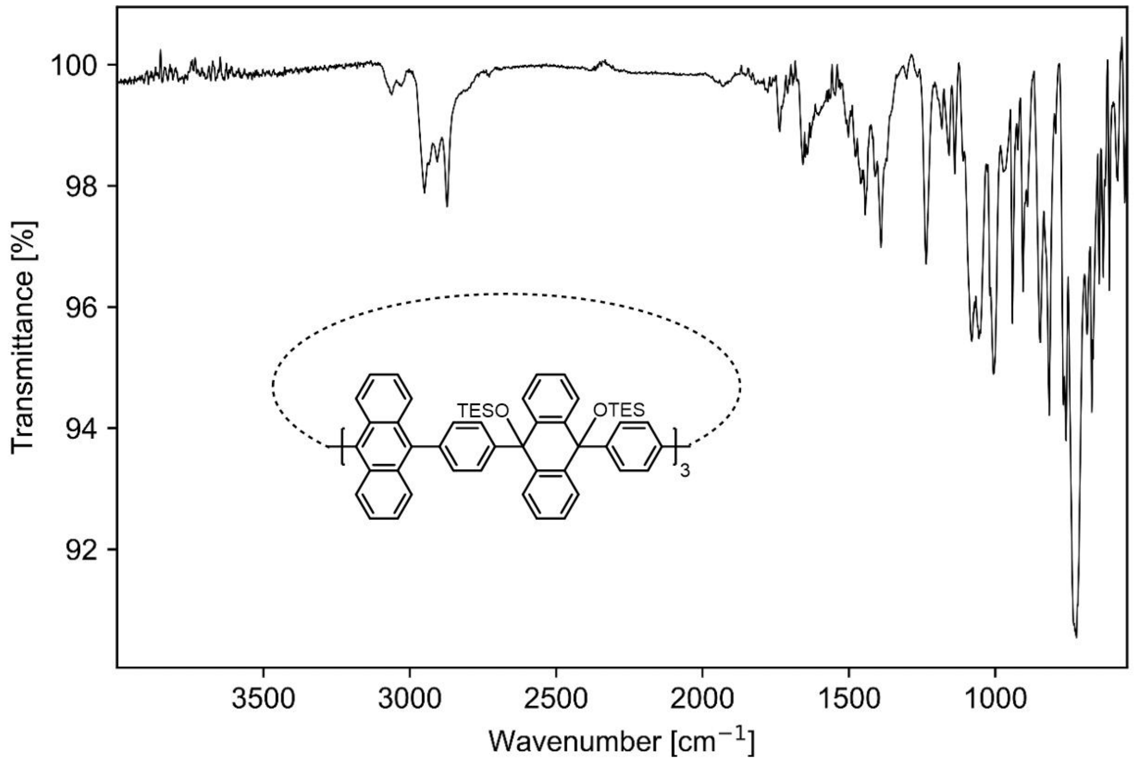

Figure S64: IR spectrum of **[6.6]CAPP-OTES** (ATR).

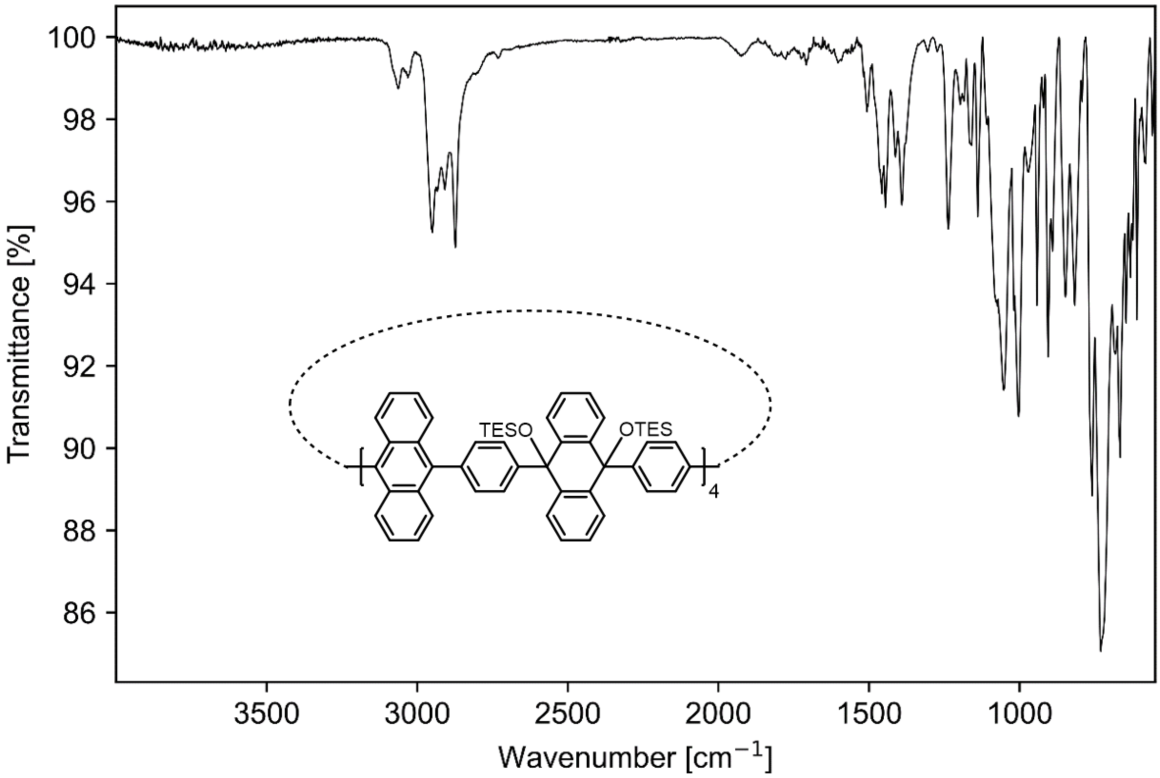

Figure S65: IR spectrum of **[8.8]CAPP-OTES** (ATR).

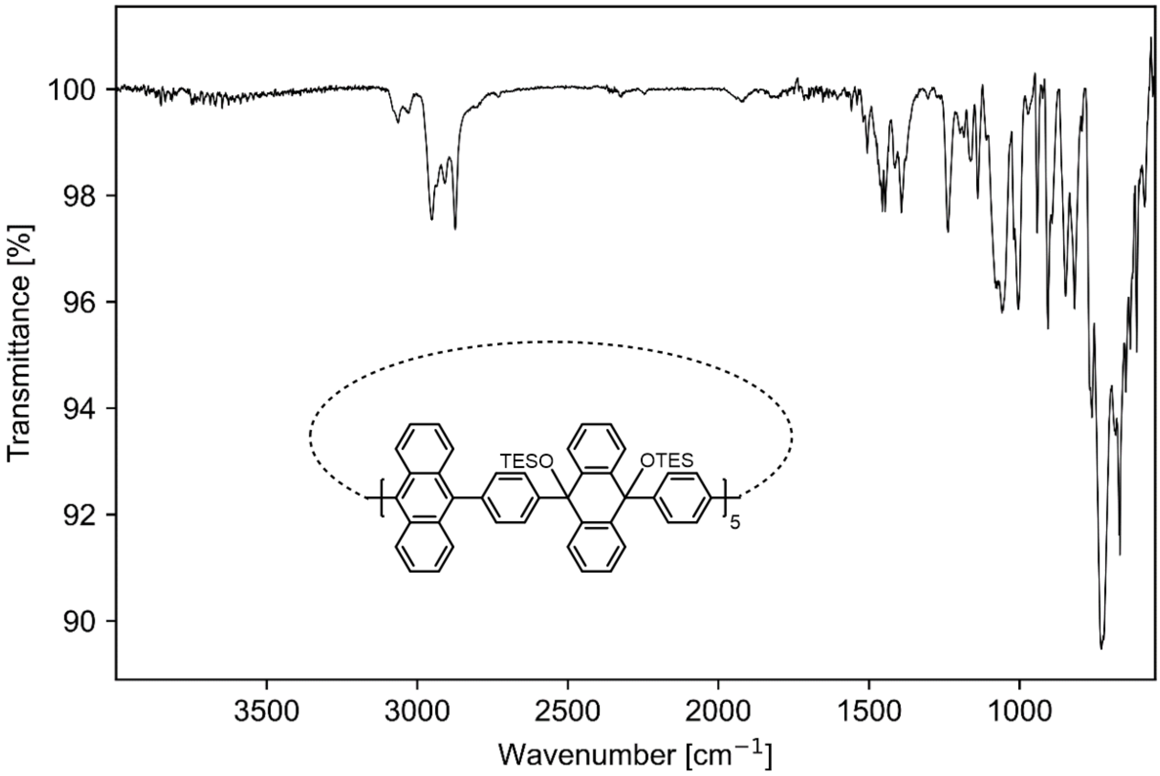

Figure S66: IR spectrum of **[10.10]CAPP-OTES** (ATR).

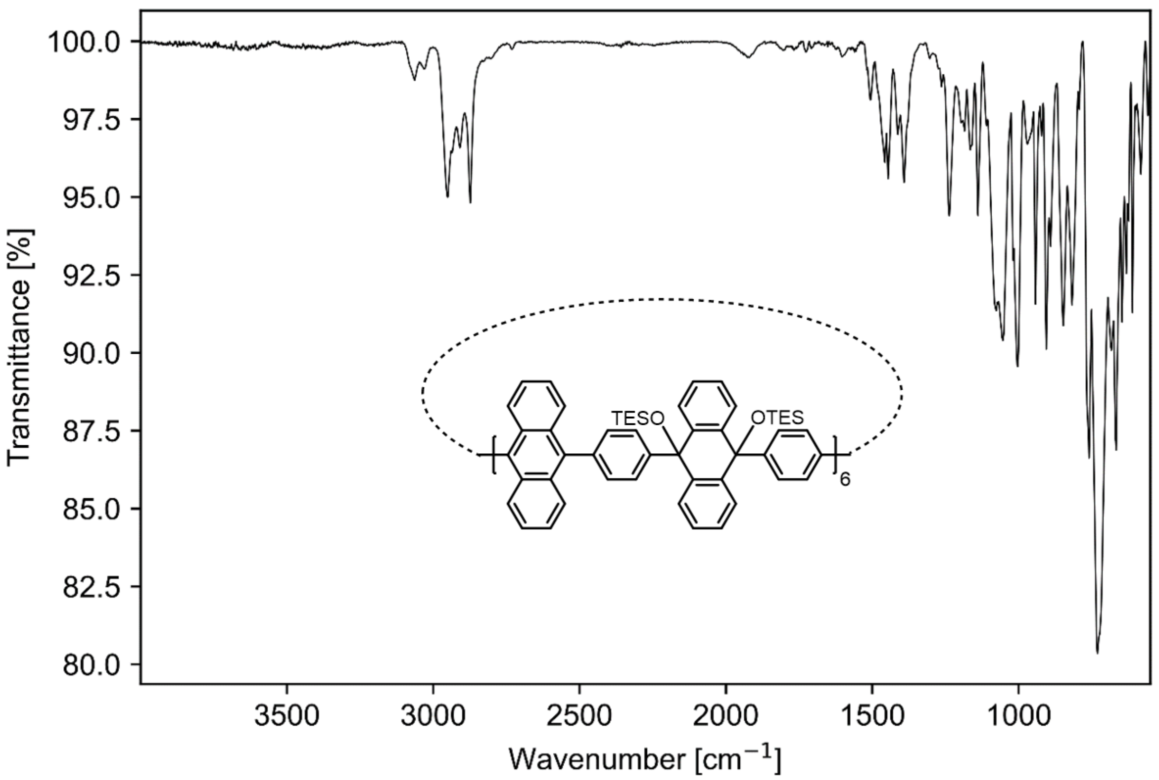

Figure S67: IR spectrum of **[12.12]CAPP-OTES** (ATR).

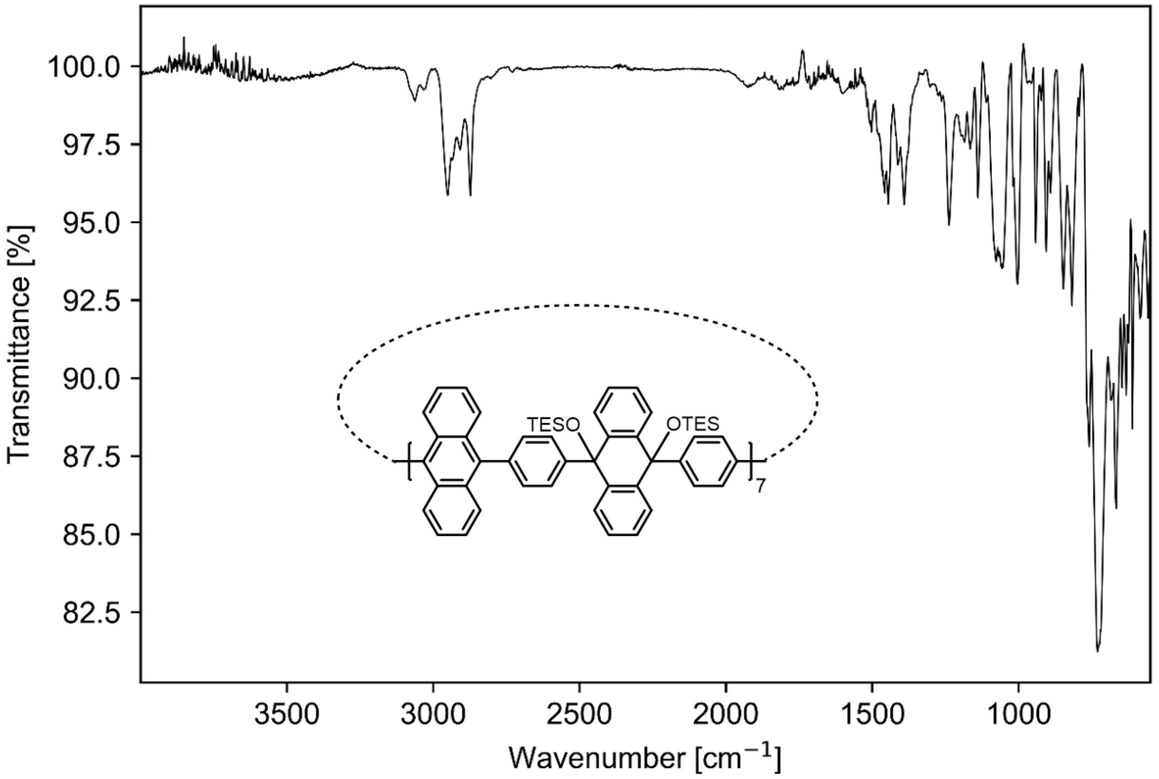

Figure S68: IR spectrum of **[14.14]CAPP-OTES** (ATR).

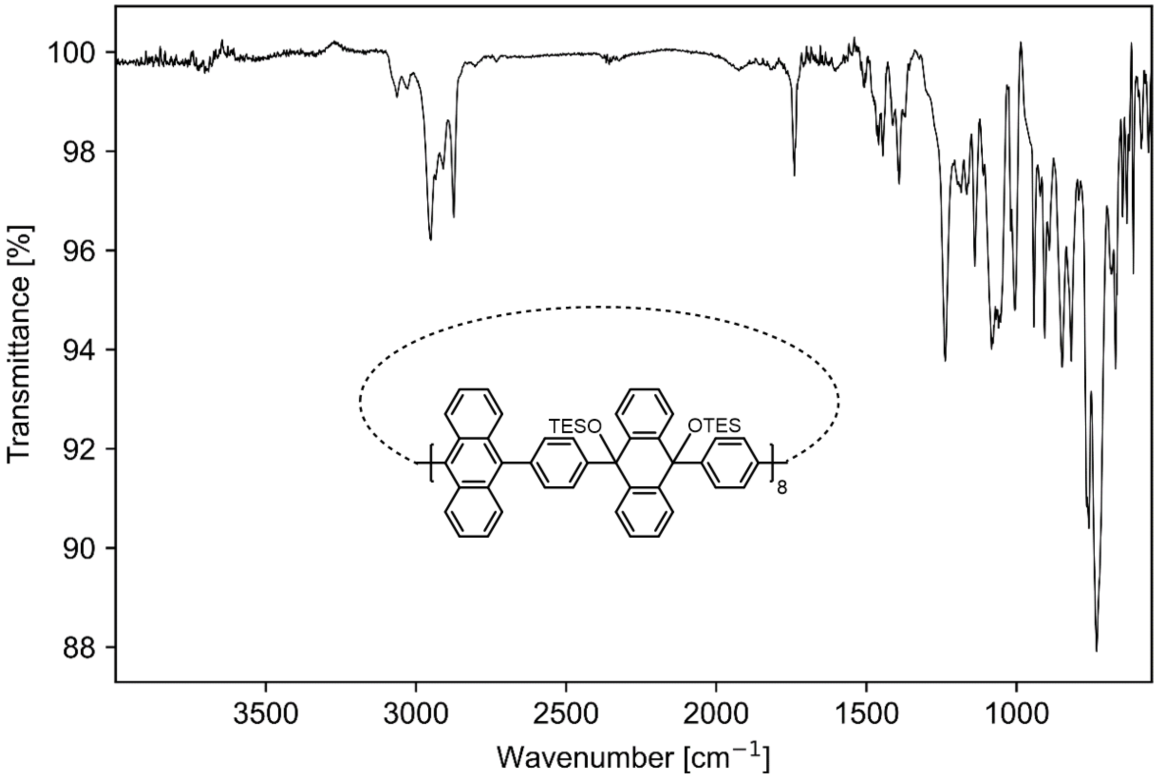

Figure S69: IR spectrum of **[16.16]CAPP-OTES** (ATR).

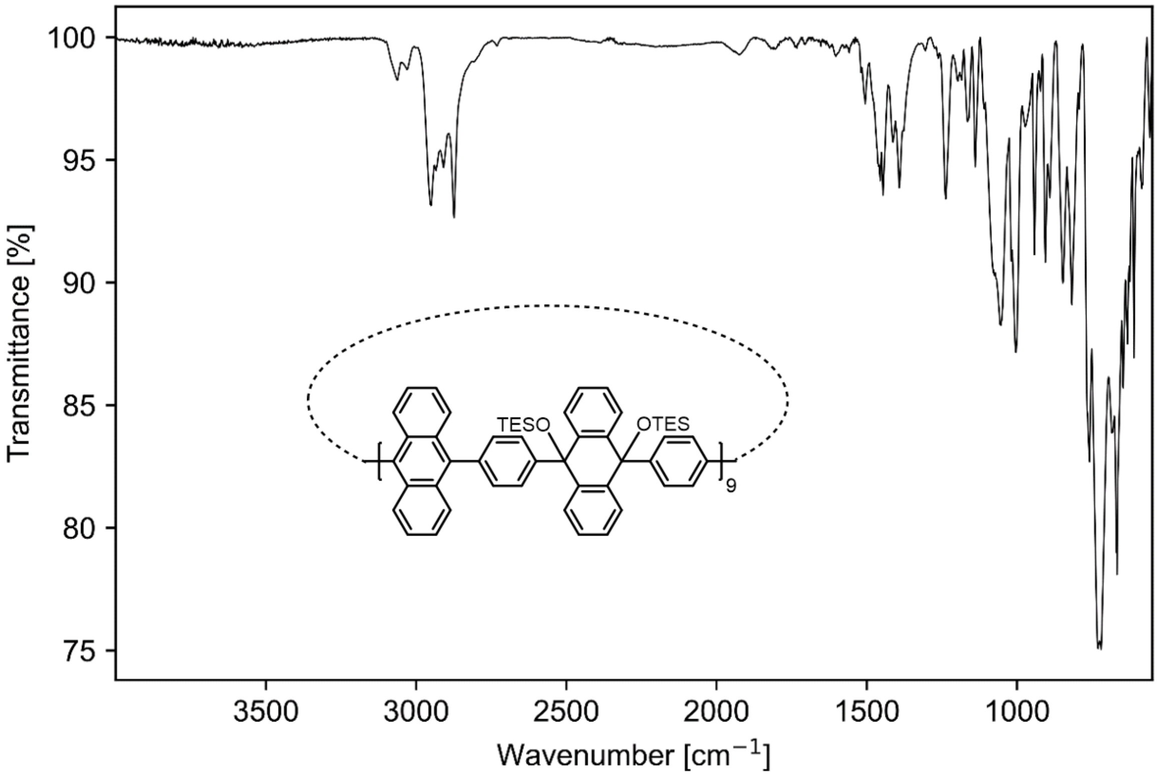

Figure S70: IR spectrum of **[16.16]CAPP-OTES** (ATR).

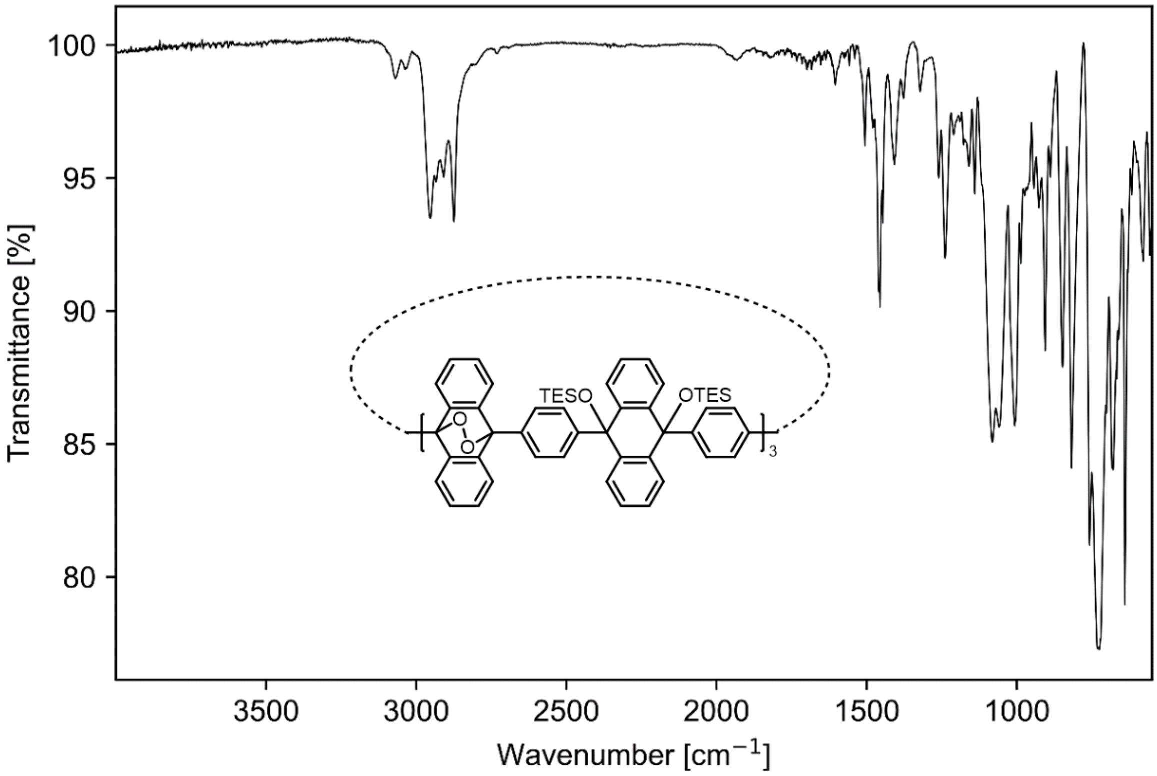

Figure S71: IR spectrum of **[6.6]CAPP-OTES-O_2** (ATR).

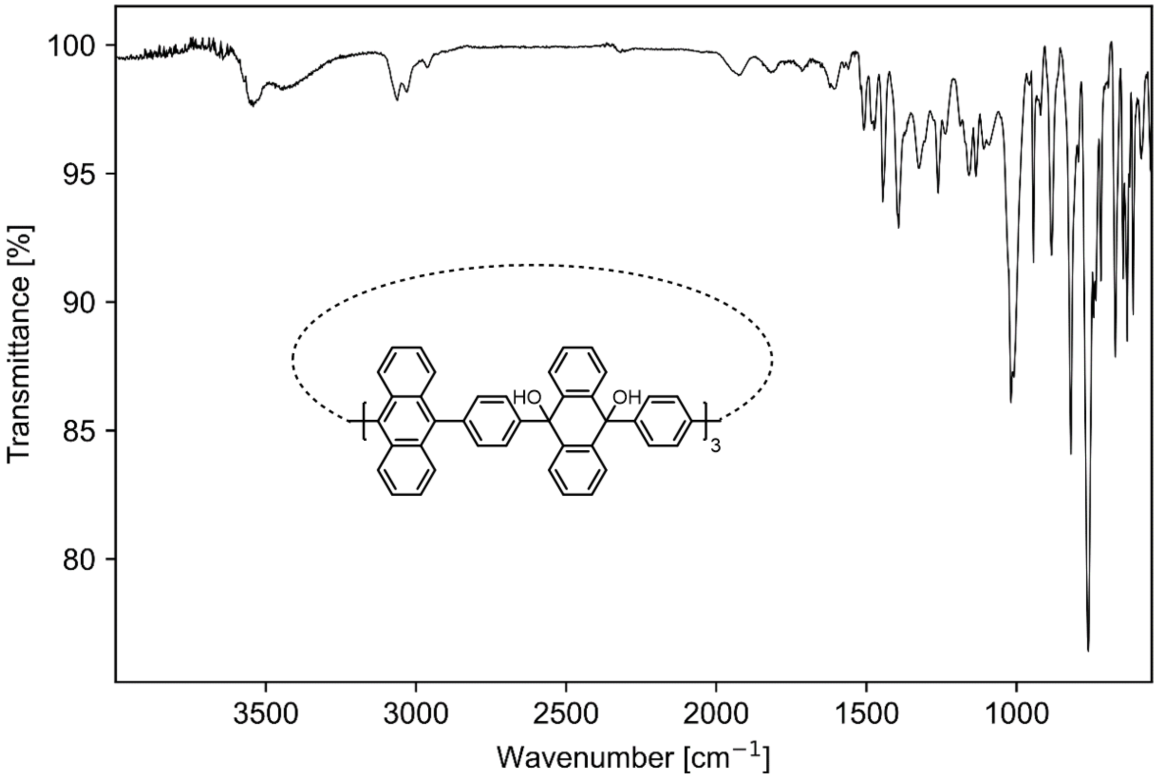

Figure S72: IR spectrum of **[6.6]CAPP-OH** (ATR).

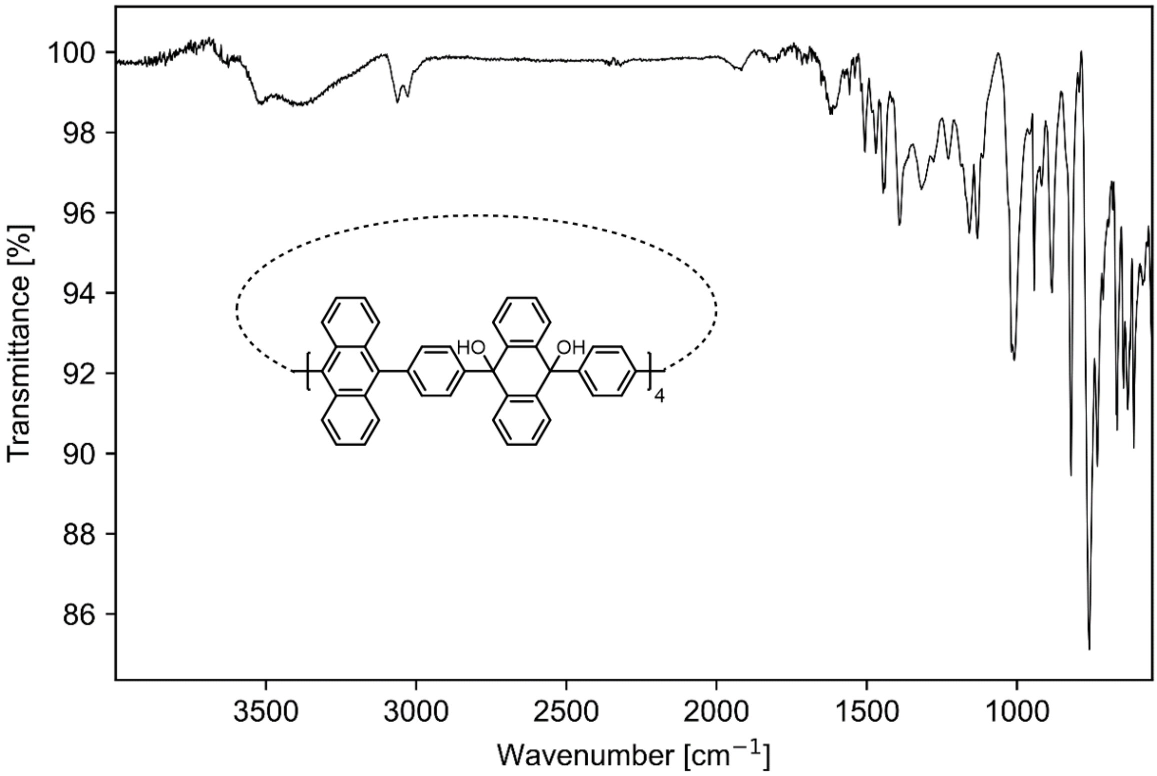

Figure S73: IR spectrum of **[8.8]CAPP-OH** (ATR).

3.4 Mass spectra

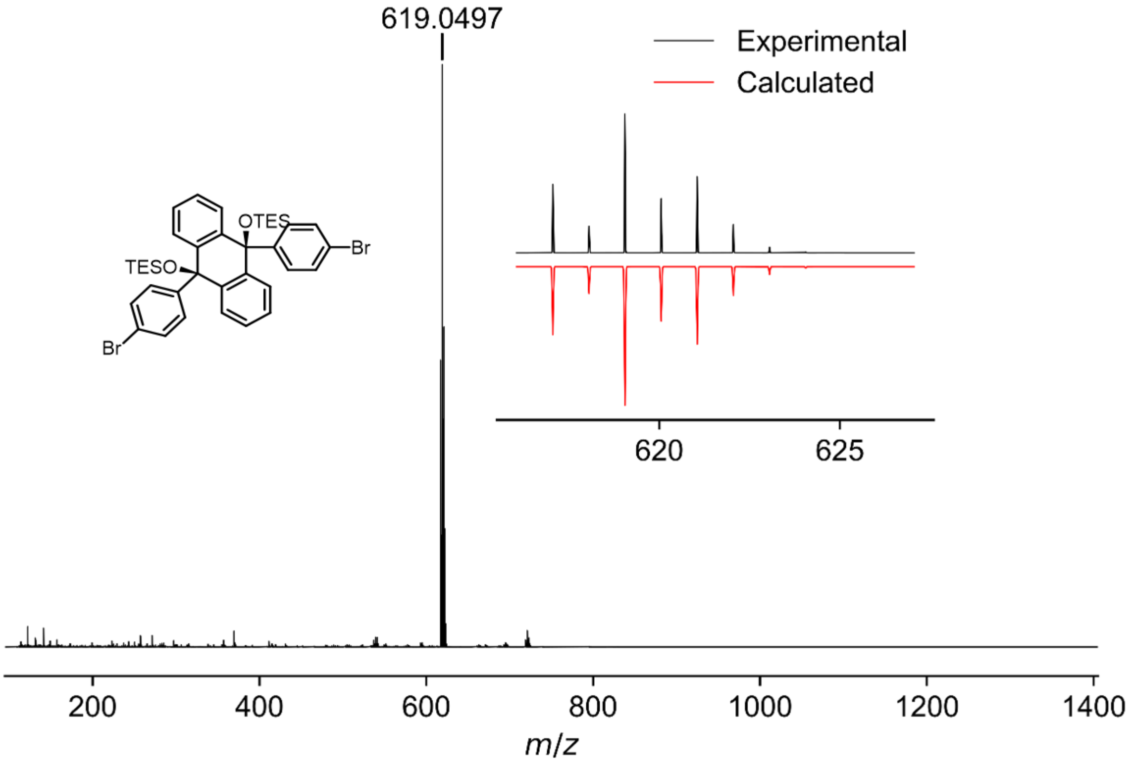

Figure S74: HR APCI mass spectrum of **4**.

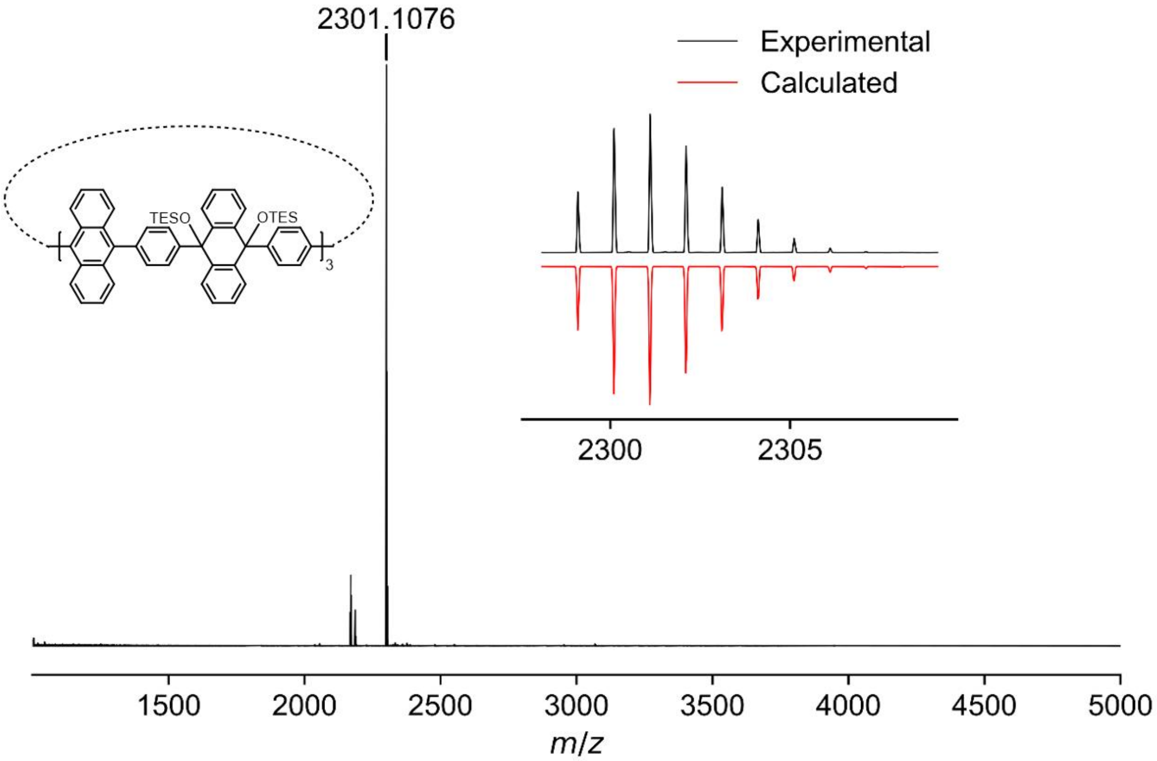

Figure S75: HR MALDI mass spectrum of **[6.6]CAPP-OTES** (DCTB, pos. mode).

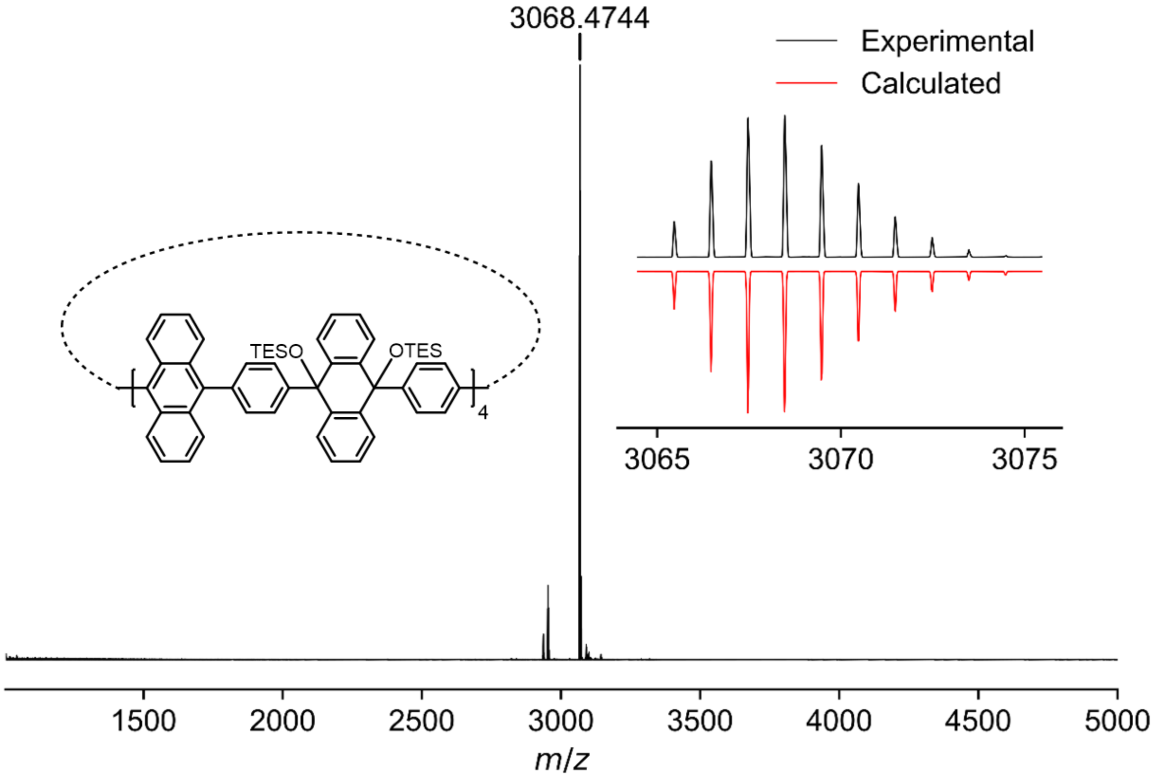

Figure S76: HR MALDI mass spectrum of **[8.8]CAPP-OTES** (DCTB, pos. mode).

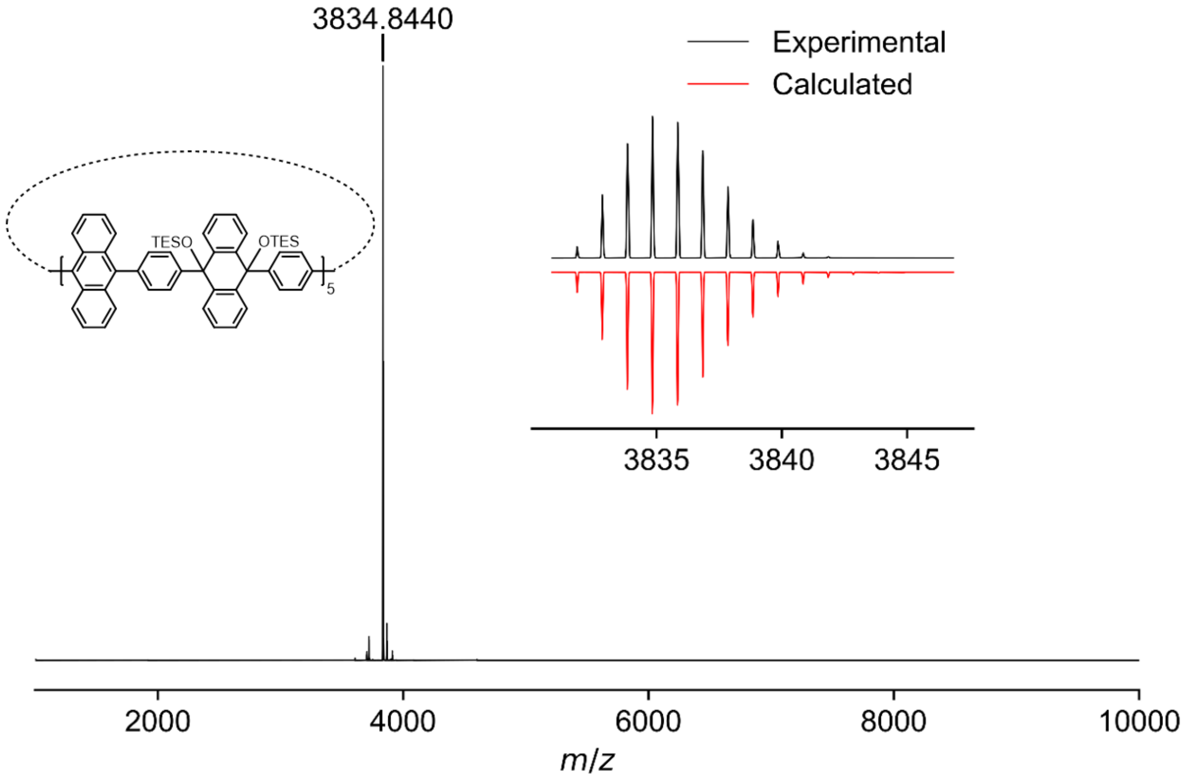

Figure S77: HR MALDI mass spectrum of **[10.10]CAPP-OTES** (DCTB, pos. mode).

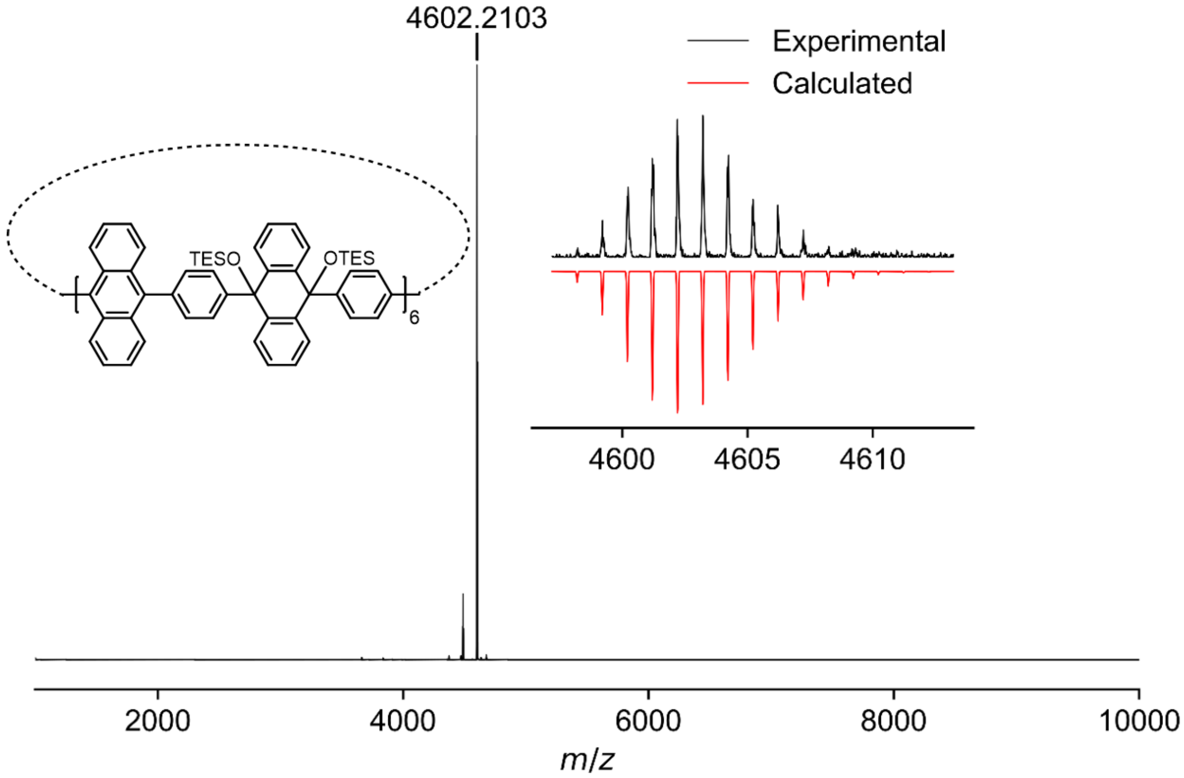

Figure S78: HR MALDI mass spectrum of **[12.12]CAPP-OTES** (DCTB, pos. mode).

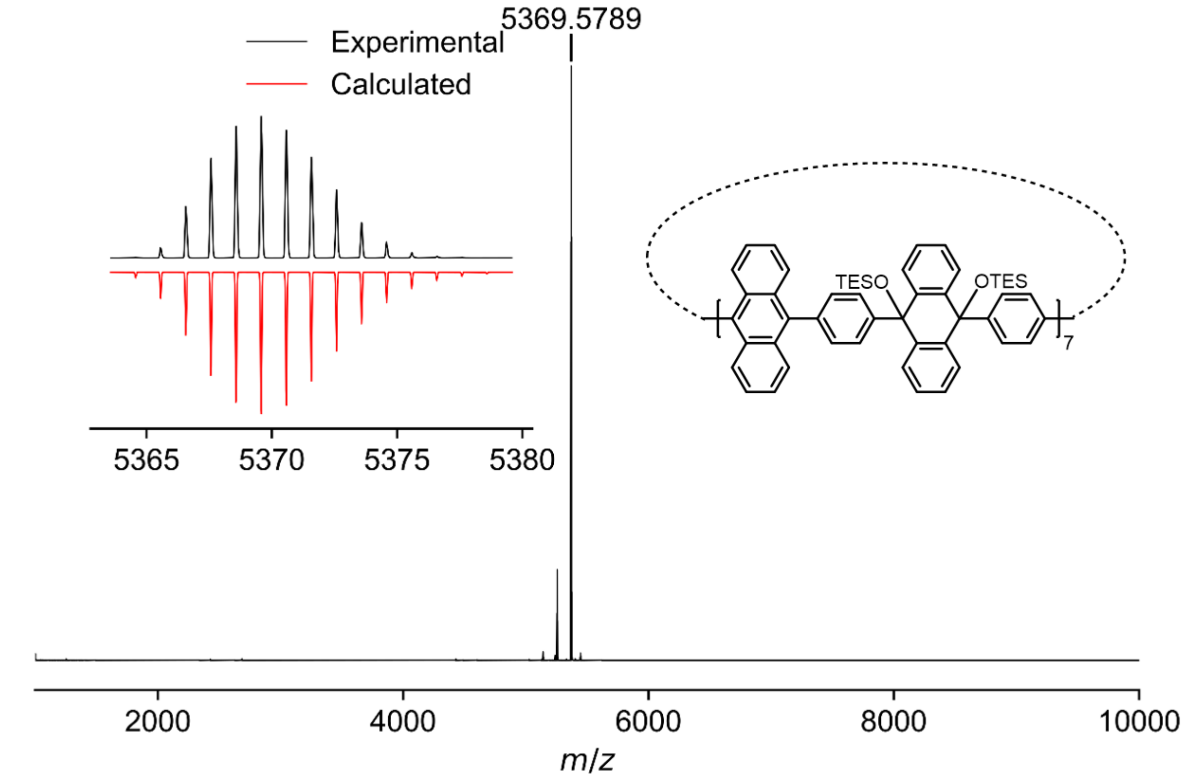

Figure S79: HR MALDI mass spectrum of **[14.14]CAPP-OTES** (DCTB, pos. mode).

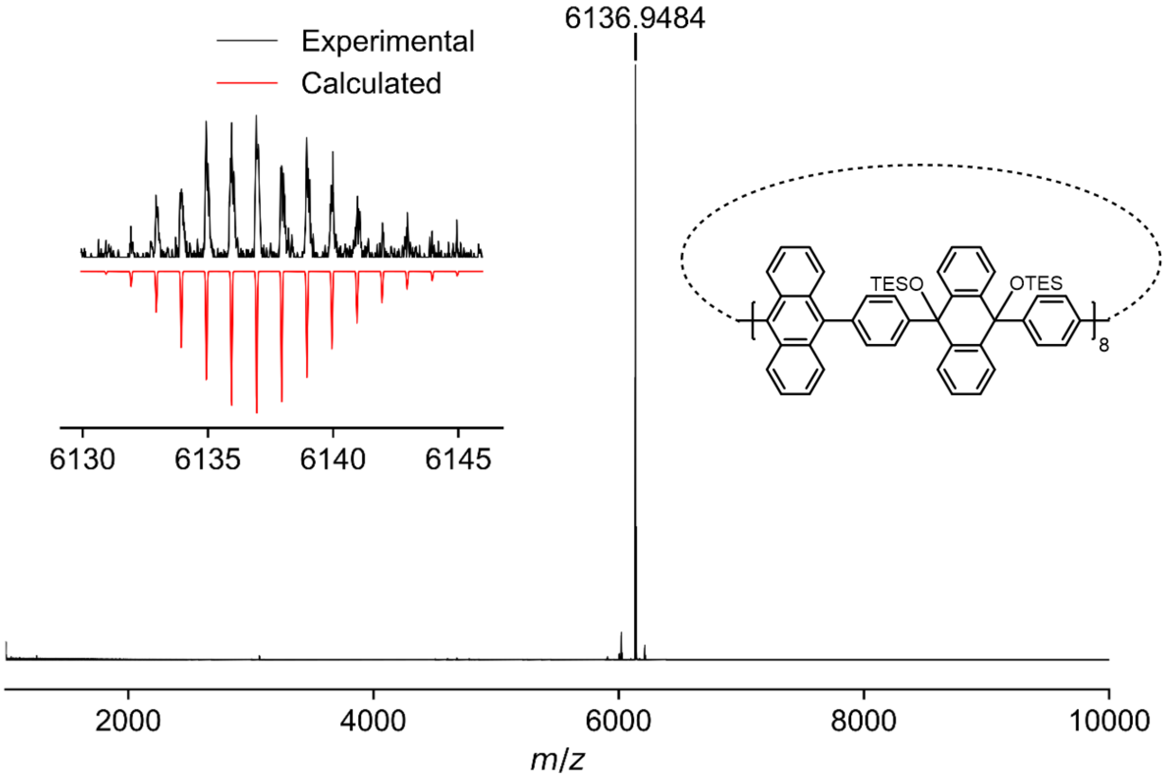

Figure S80: HR MALDI mass spectrum of **[16.16]CAPP-OTES** (DCTB, pos. mode).

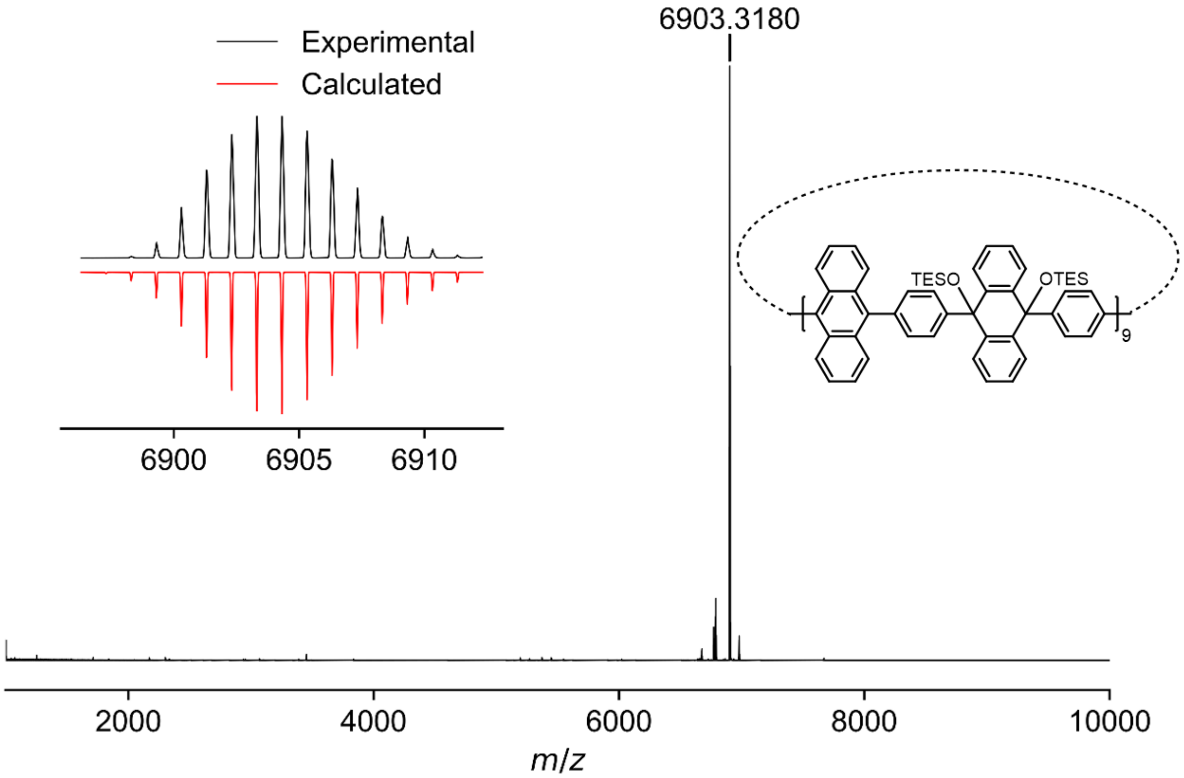

Figure S81: HR MALDI mass spectrum of **[18.18]CAPP-OTES** (DCTB, pos. mode).

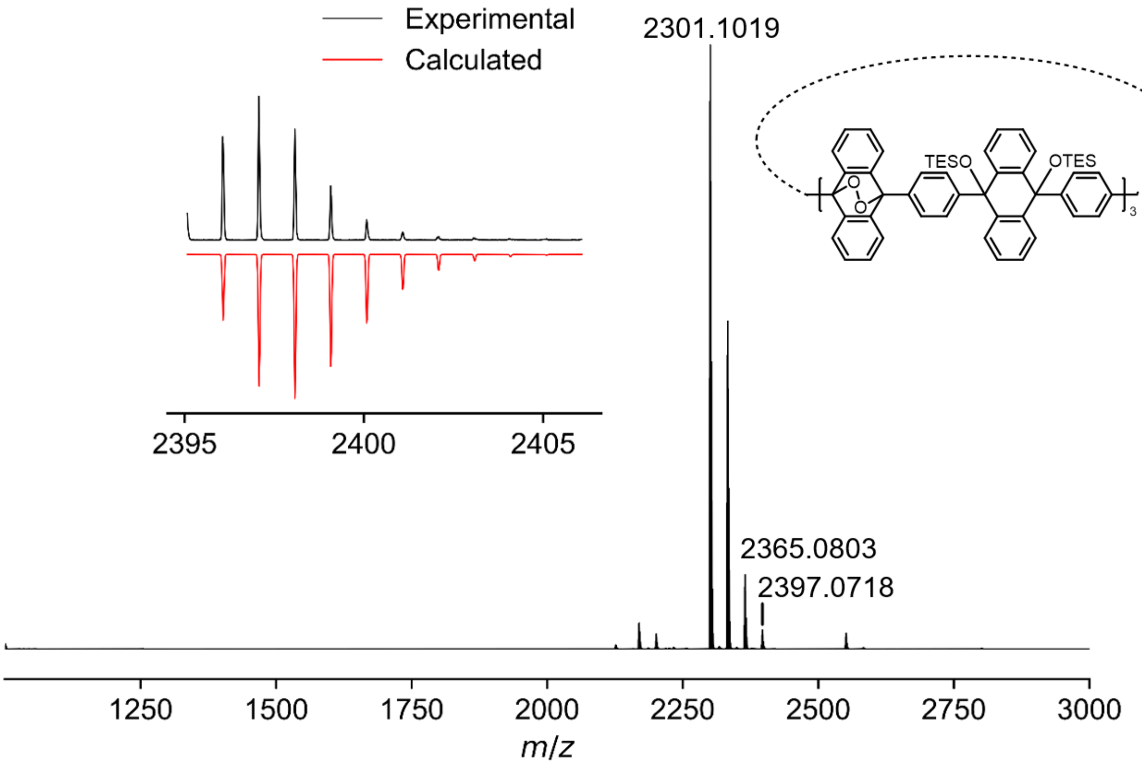

Figure S82: HR MALDI mass spectrum of **[6.6]CAPP-O₂** (DCTB, pos. mode).

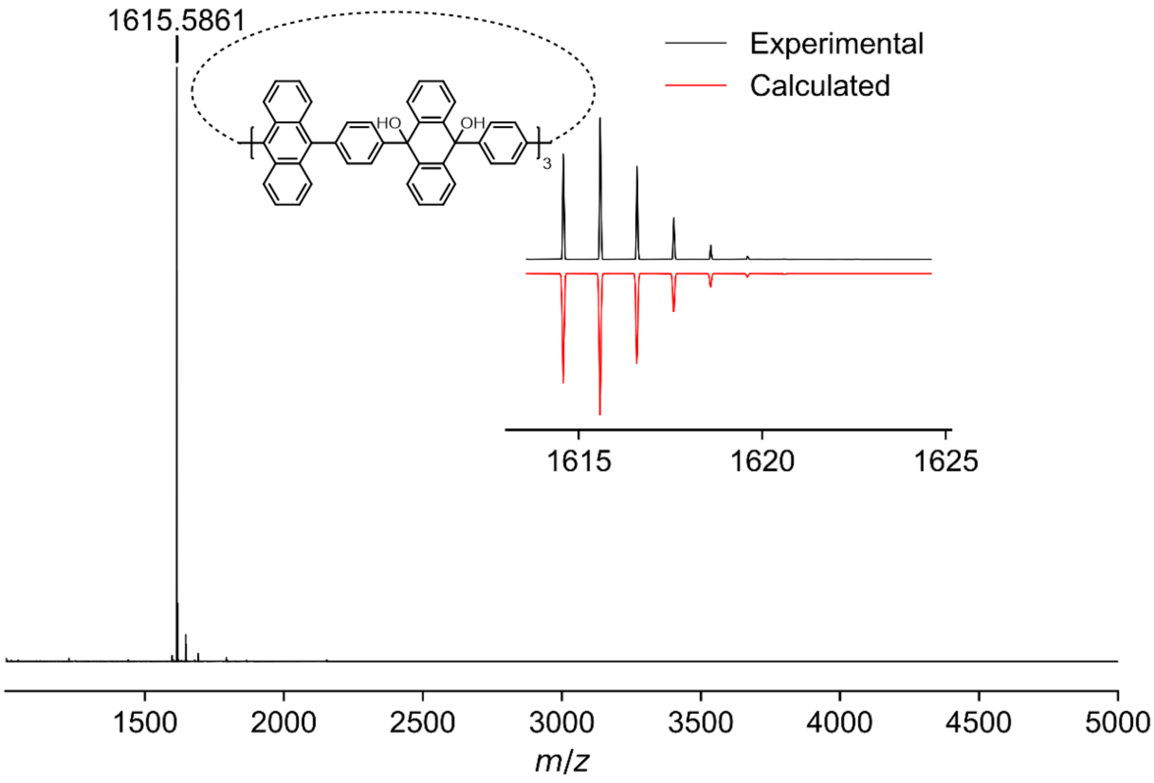

Figure S83: HR MALDI mass spectrum of **[6.6]CAPP-OH** (DCTB, pos. mode).

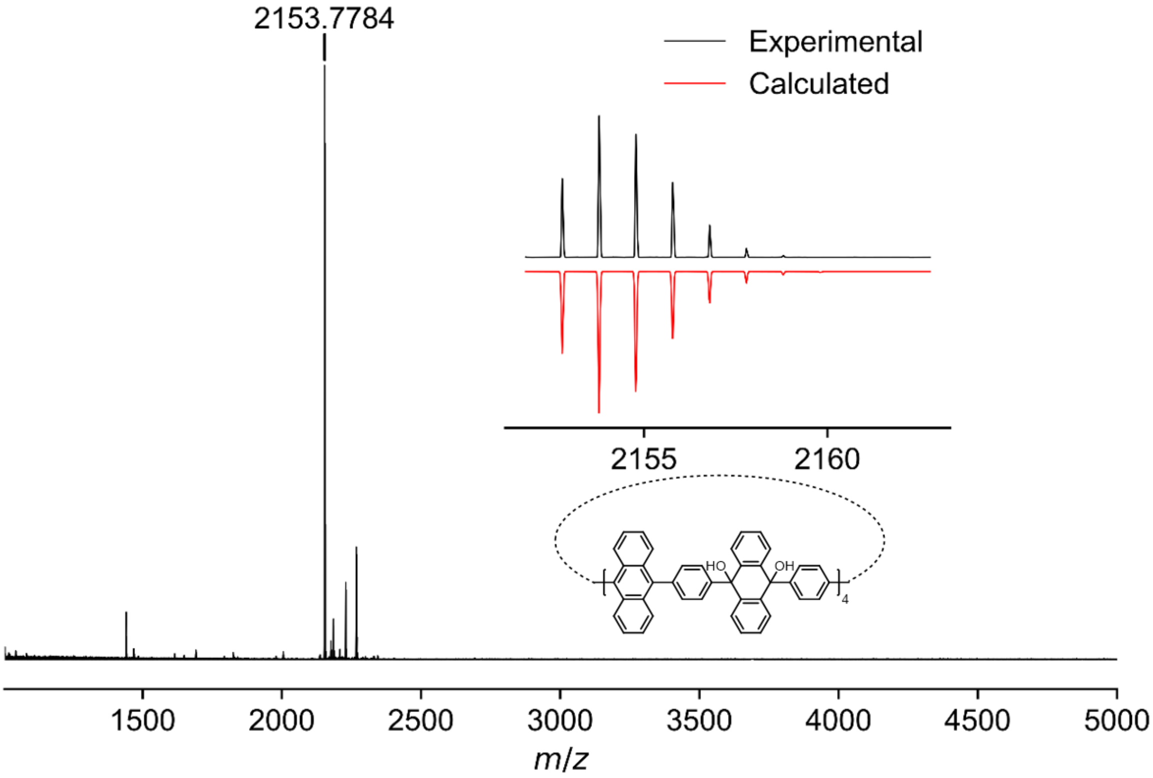

Figure S84: HR MALDI mass spectrum of **[8.8]CAPP-OH** (DCTB, pos. mode).

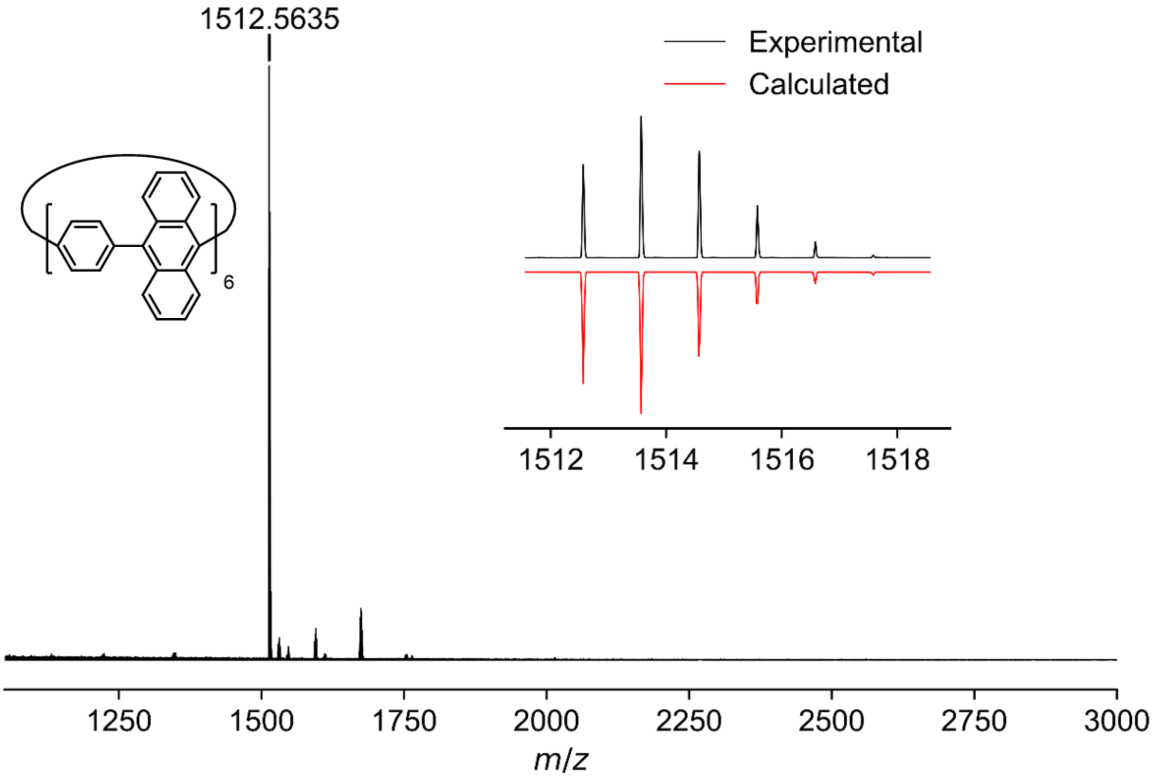

Figure S85: HR MALDI mass spectrum of crude **[6.6]CAPP** (DCTB, pos. mode).

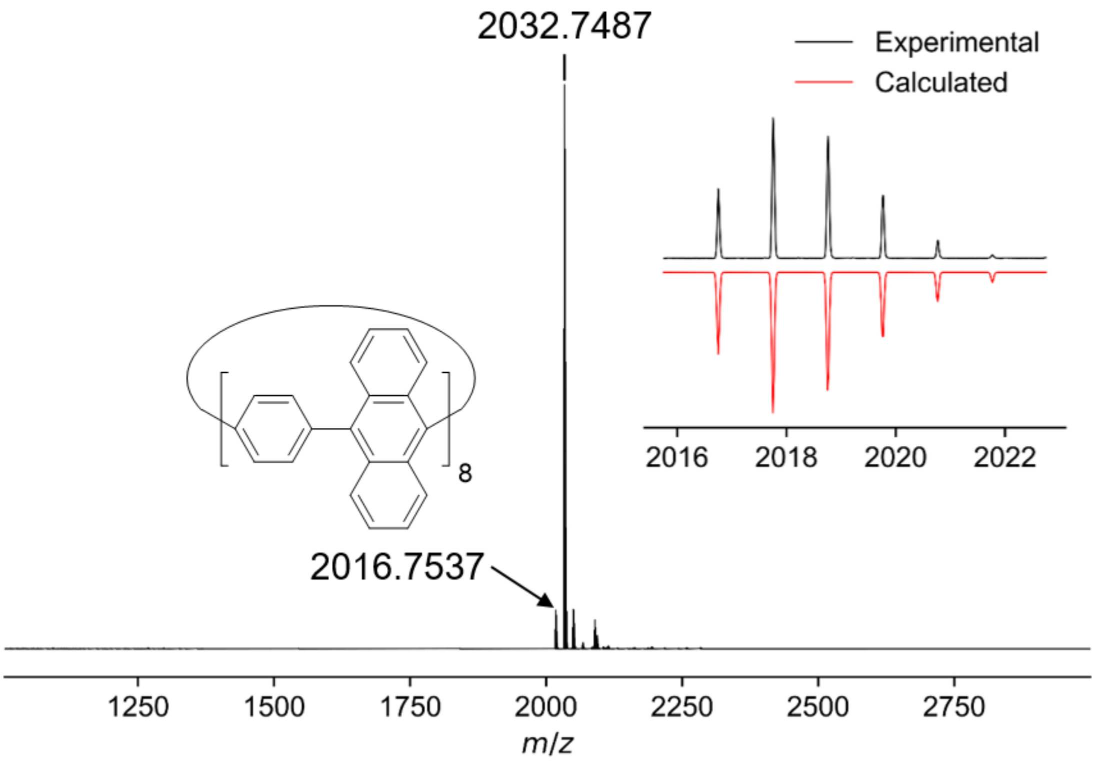

Figure S86: HR MALDI mass spectrum of crude **[8.8]CAPP** (DCTB, pos. mode).

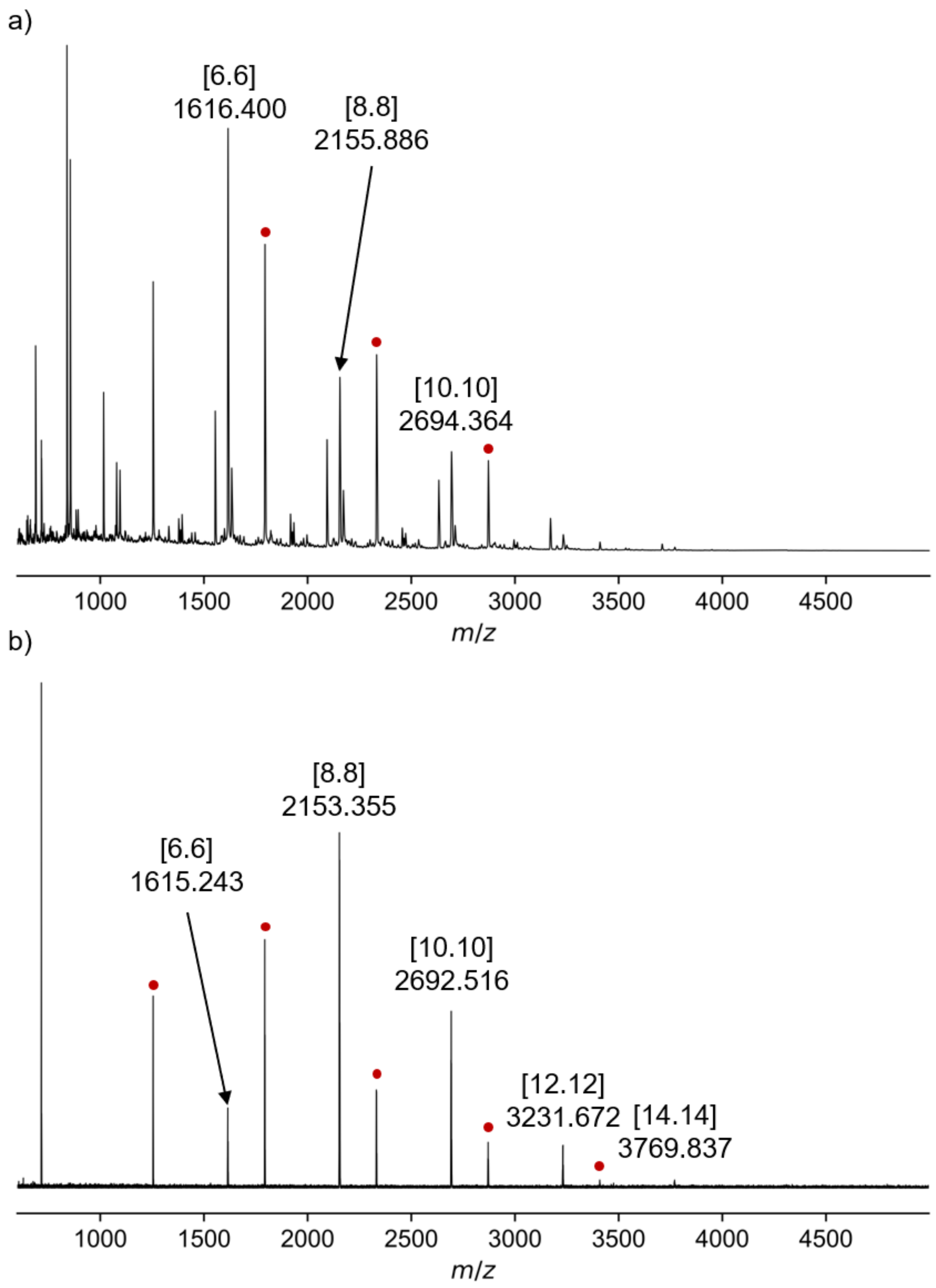

Figure S87: MALDI mass spectra of crude reaction mixtures of the direct route towards **[n.n]CAPP-OH** (DCTB, pos. mode) using a) Pd_2dba_3 and XPhos with 2M K_2CO_3 or b) SPhos Pd G3 with 1M K_3PO_4. Deborylated, linear oligomers with n diol units and n+1 anthracene units are marked with red dots.

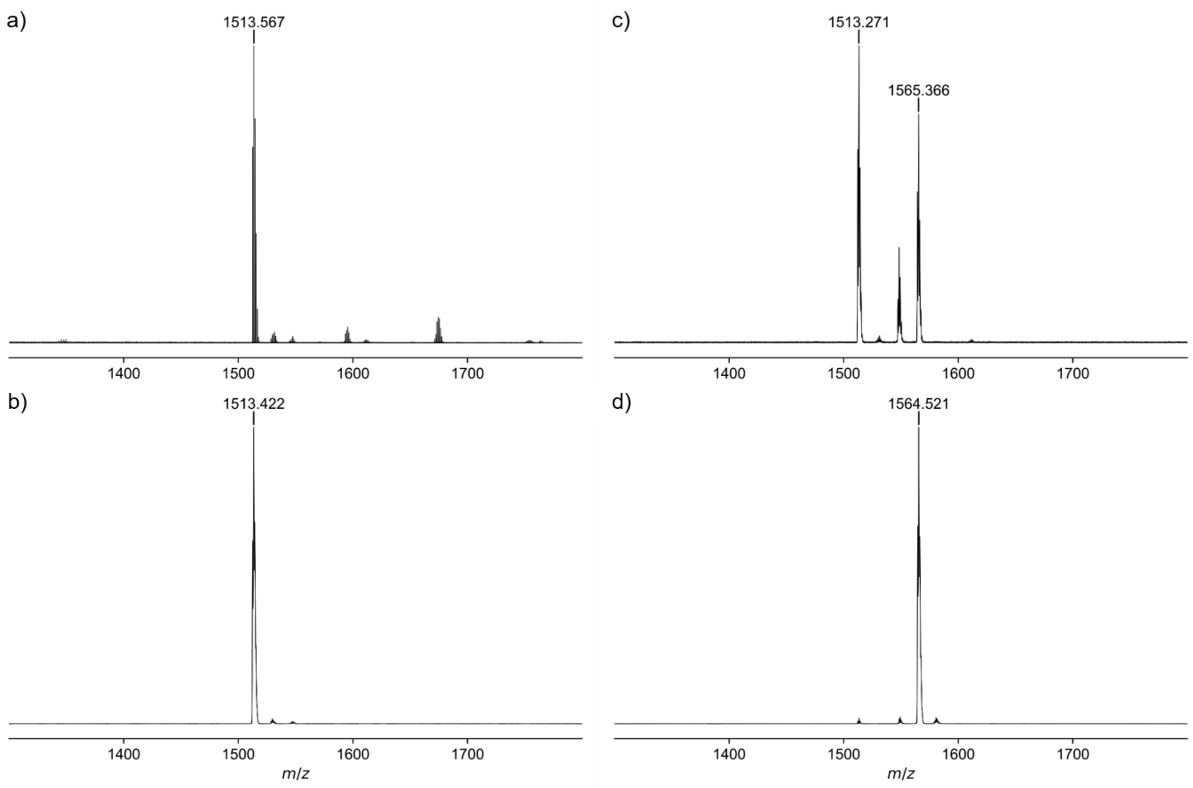

Figure S88: MALDI mass spectra of crude **[6.6]CAPP** (DCTB, pos. mode) a) directly after the reaction b) 19 h in the reaction mixture under argon c) filtration through a silica plug d) 2.5 h after the filtration.

3.5 UV/Vis and fluorescence spectra

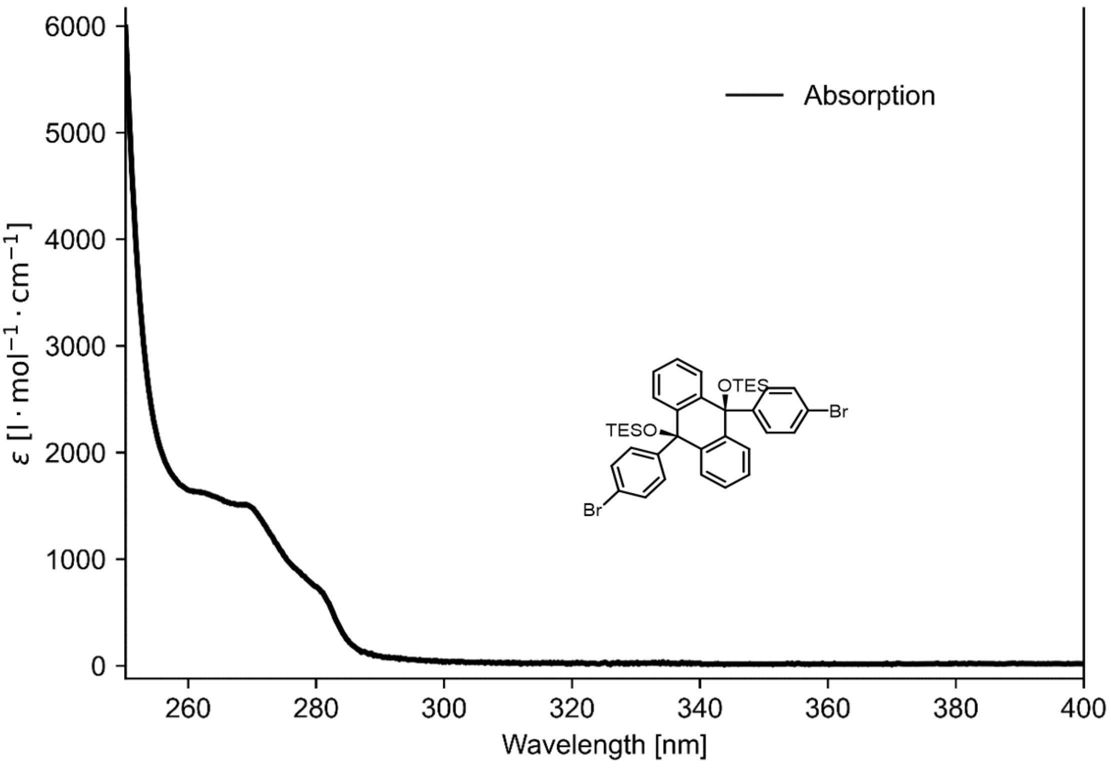

Figure S89: UV/Vis spectrum of **4** in dichloromethane.

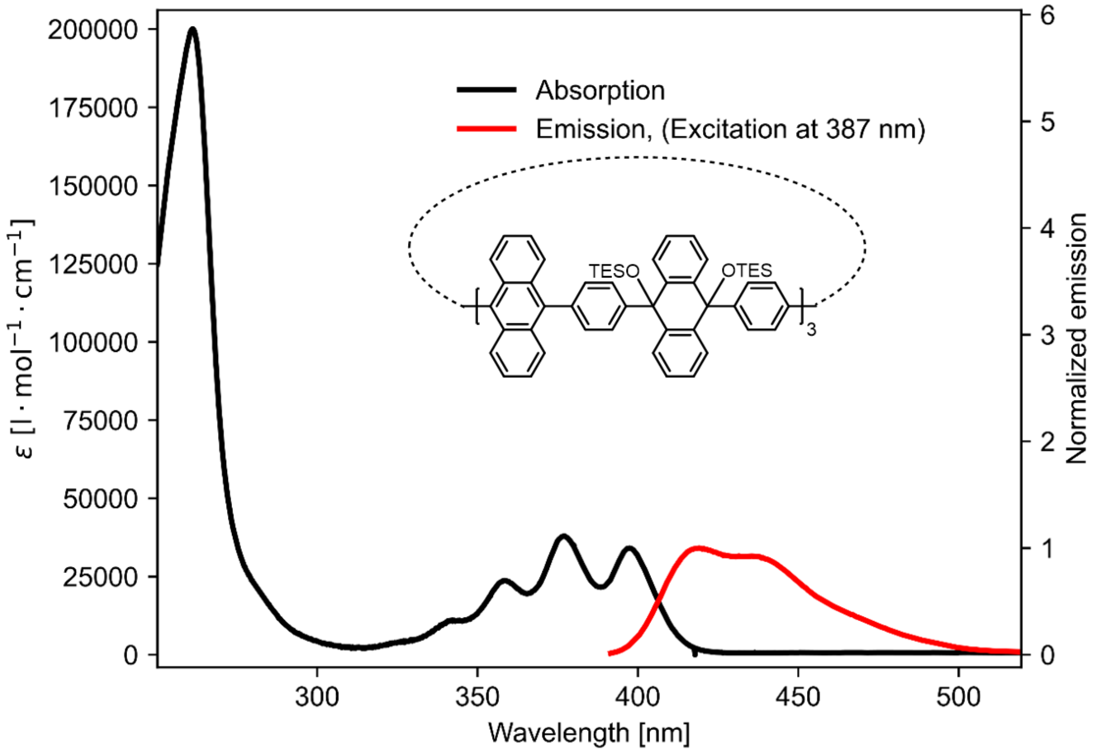

Figure S90: UV/Vis and fluorescence spectra of **[6.6]CAPP-OTES** in dichloromethane.

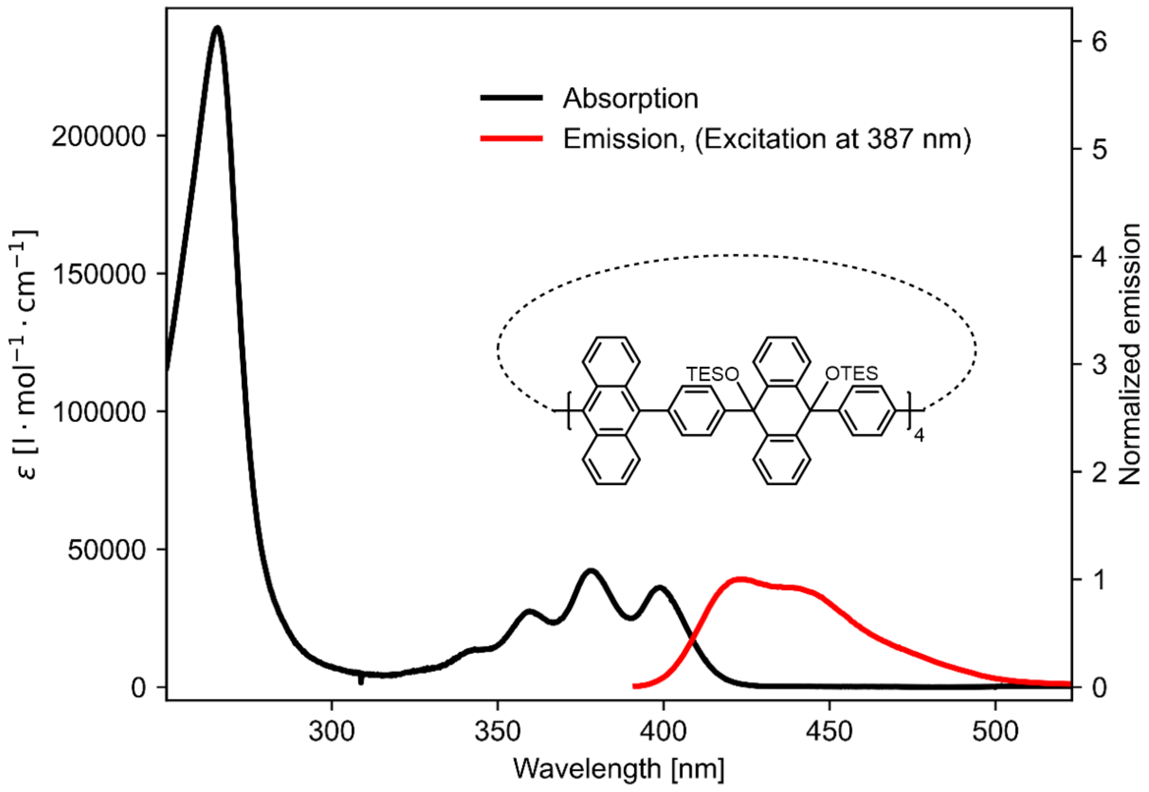

Figure S91: UV/Vis and fluorescence spectra of **[8.8]CAPP-OTES** in dichloromethane.

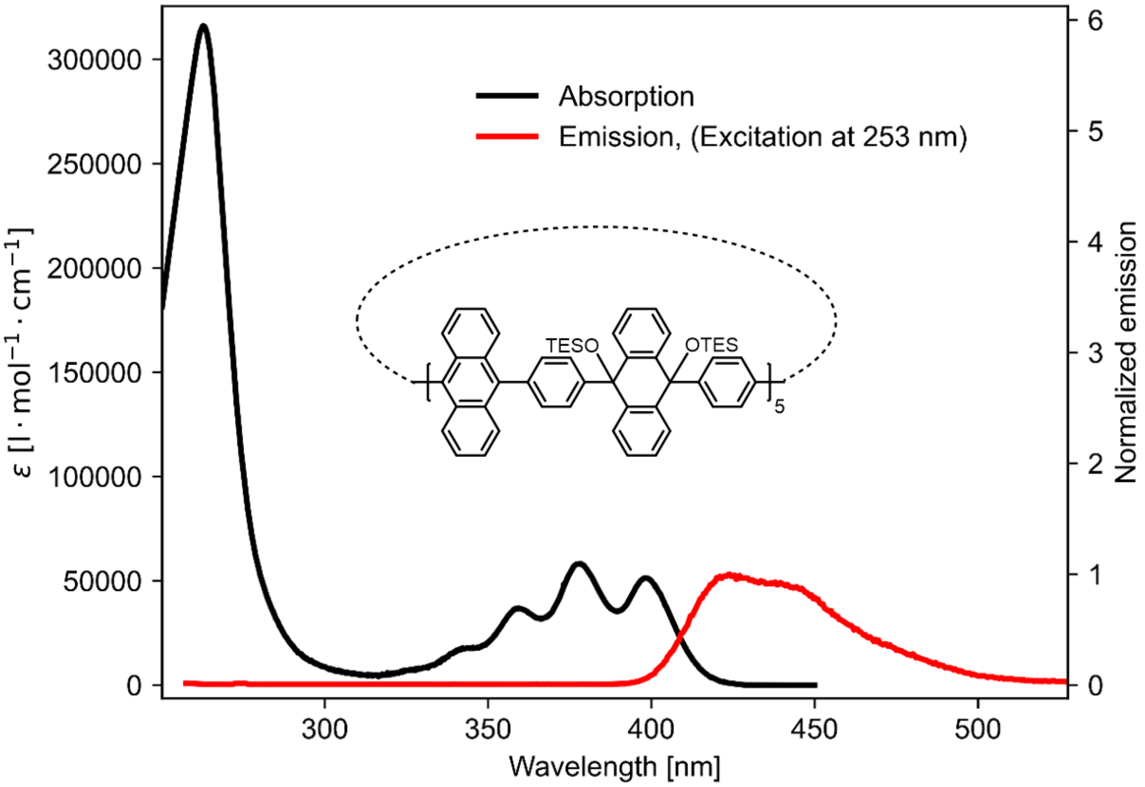

Figure S92: UV/Vis and fluorescence spectra of **[10.10]CAPP-OTES** in dichloromethane.

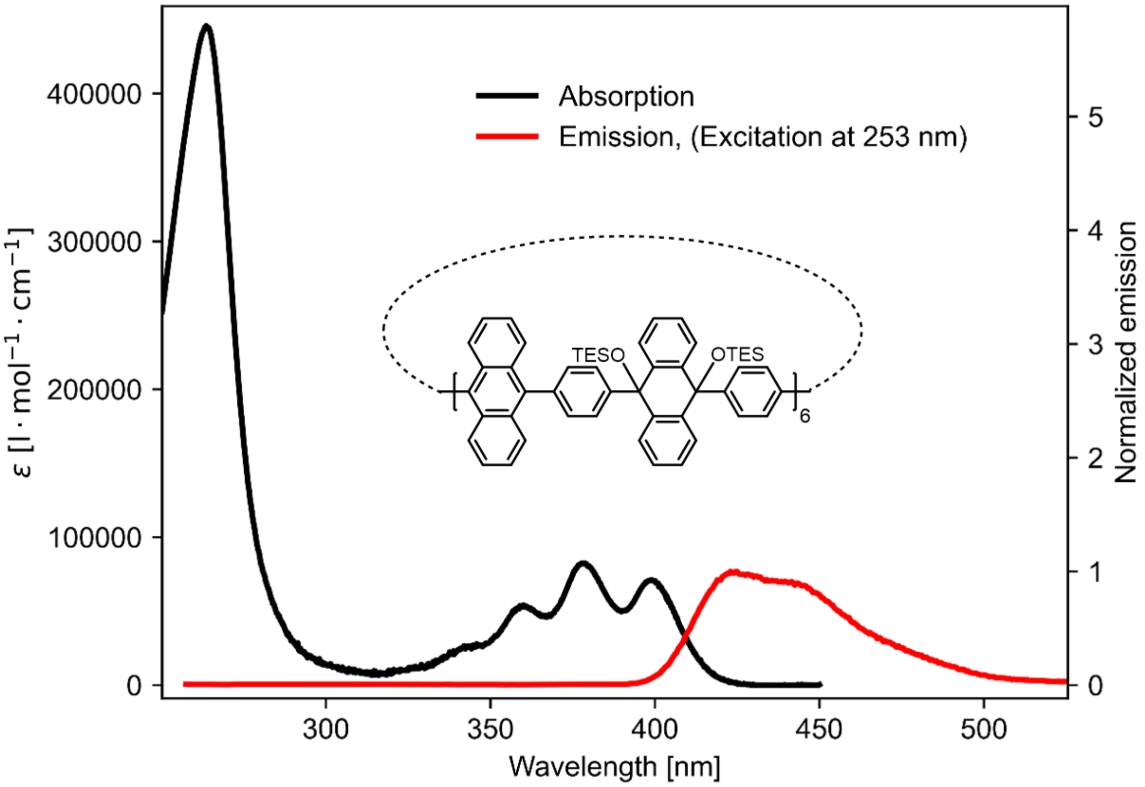

Figure S93: UV/Vis and fluorescence spectra of **[12.12]CAPP-OTES** in dichloromethane.

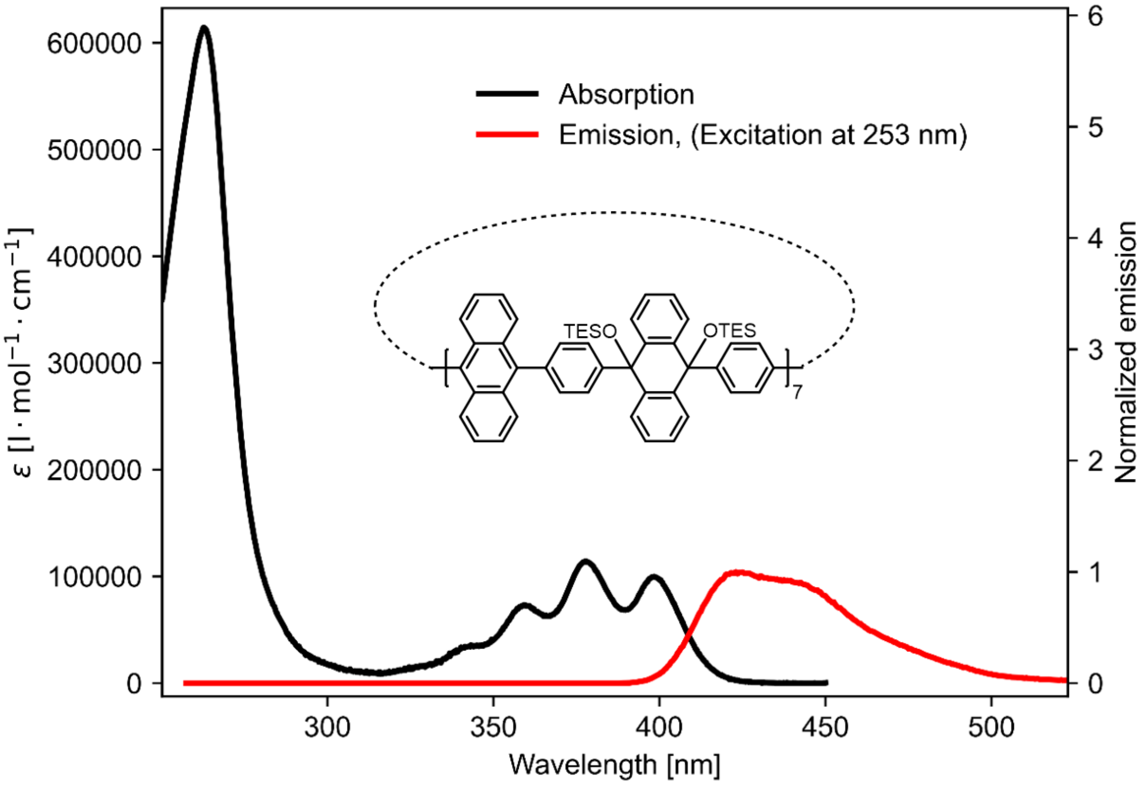

Figure S94: UV/Vis and fluorescence spectra of **[14.14]CAPP-OTES** in dichloromethane.

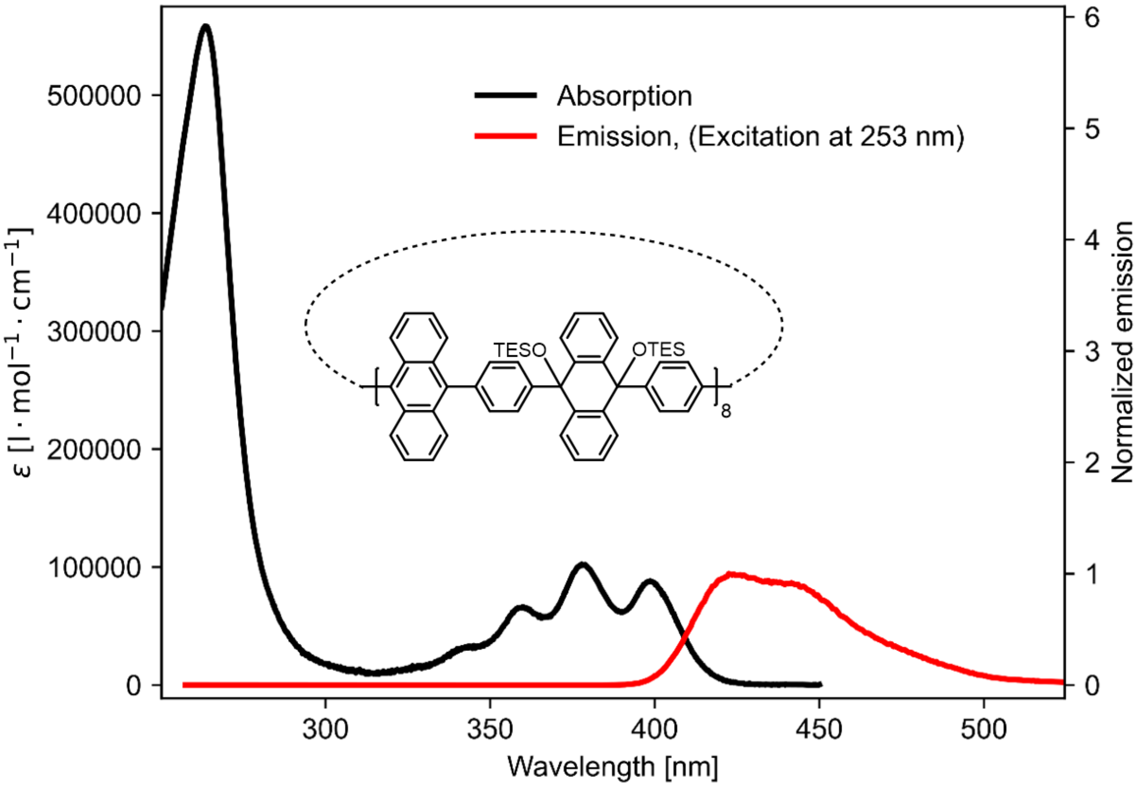

Figure S95: UV/Vis and fluorescence spectra of **[16.16]CAPP-OTES** in dichloromethane.

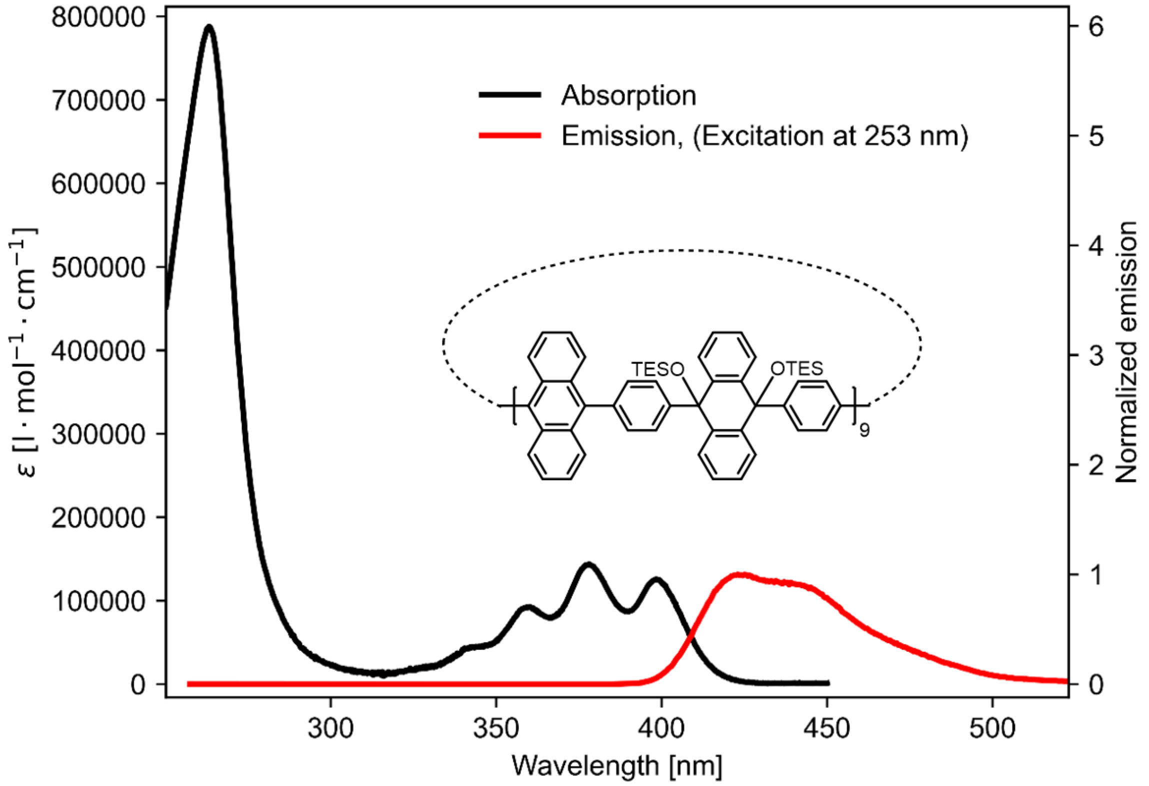

Figure S96: UV/Vis and fluorescence spectra of **[18.18]CAPP-OTES** in dichloromethane.

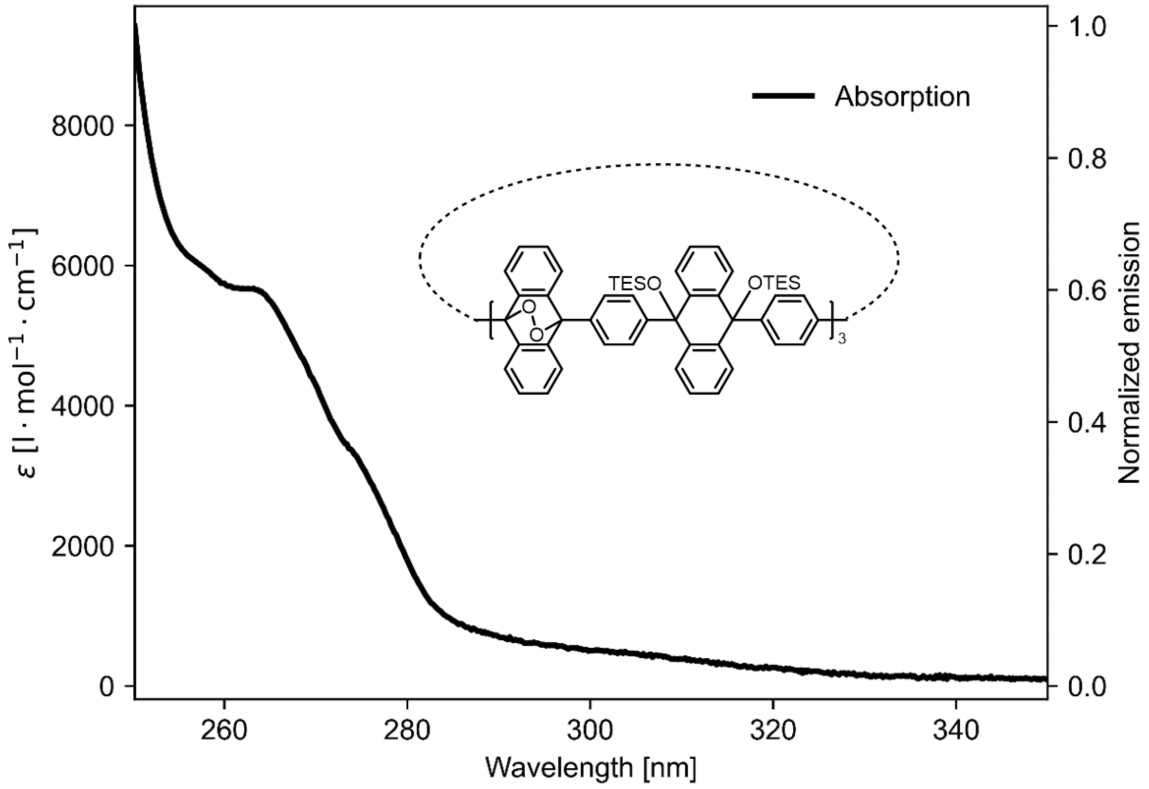

Figure S97: UV/Vis spectrum of **[6.6]CAPP-OTES-O_2** in dichloromethane.

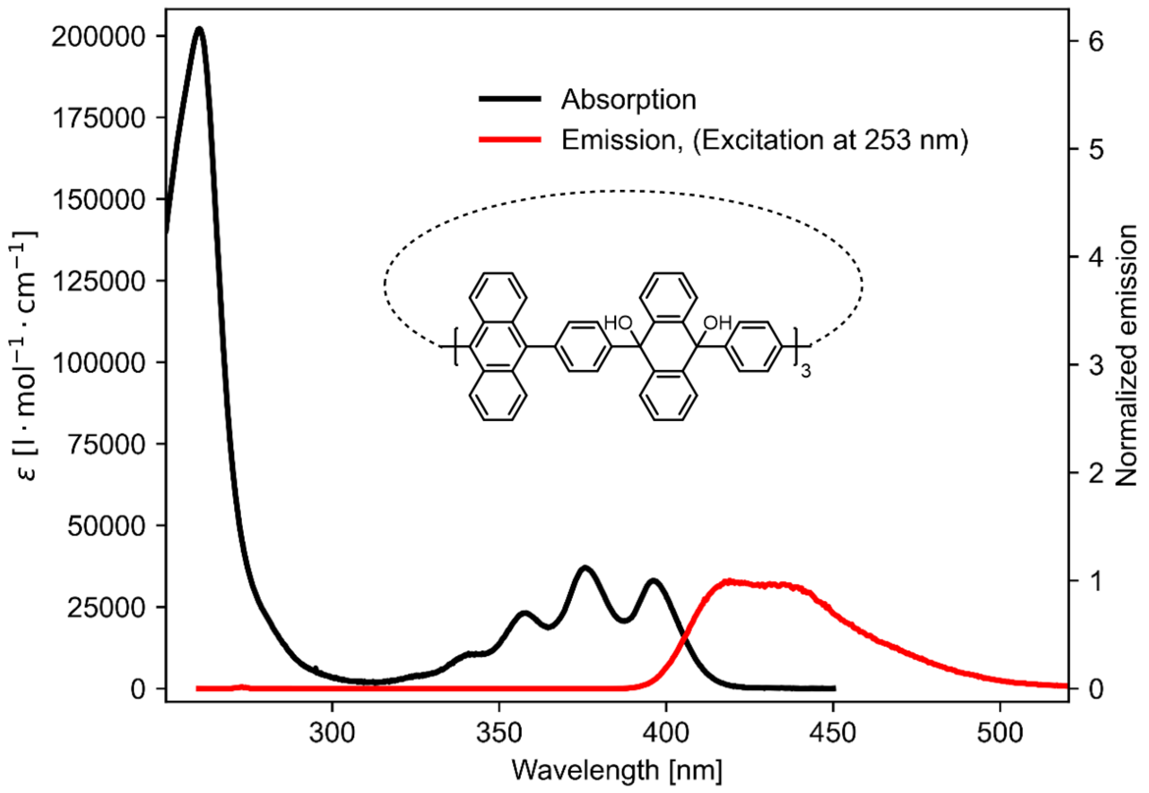

Figure S98: UV/Vis and fluorescence spectra of **[6.6]CAPP-OH** in tetrahydrofuran.

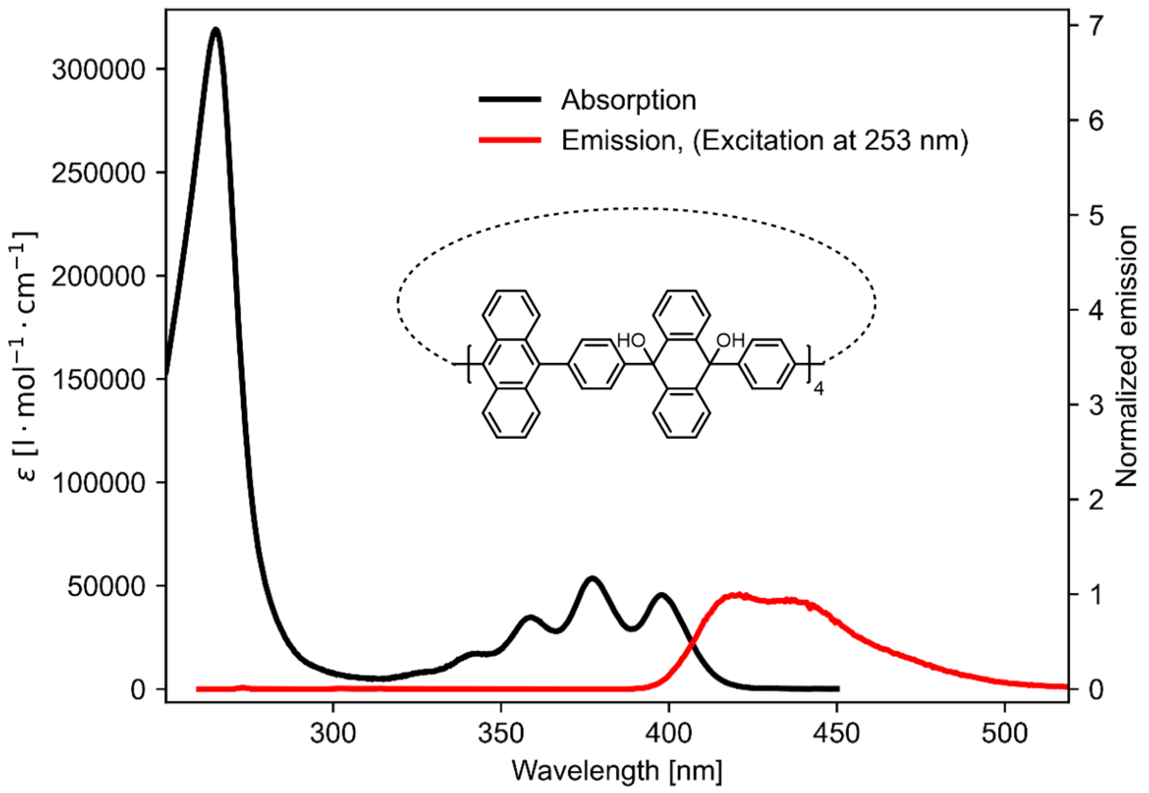

Figure S99: UV/Vis and fluorescence spectra of **[8.8]CAPP-OH** in tetrahydrofuran.

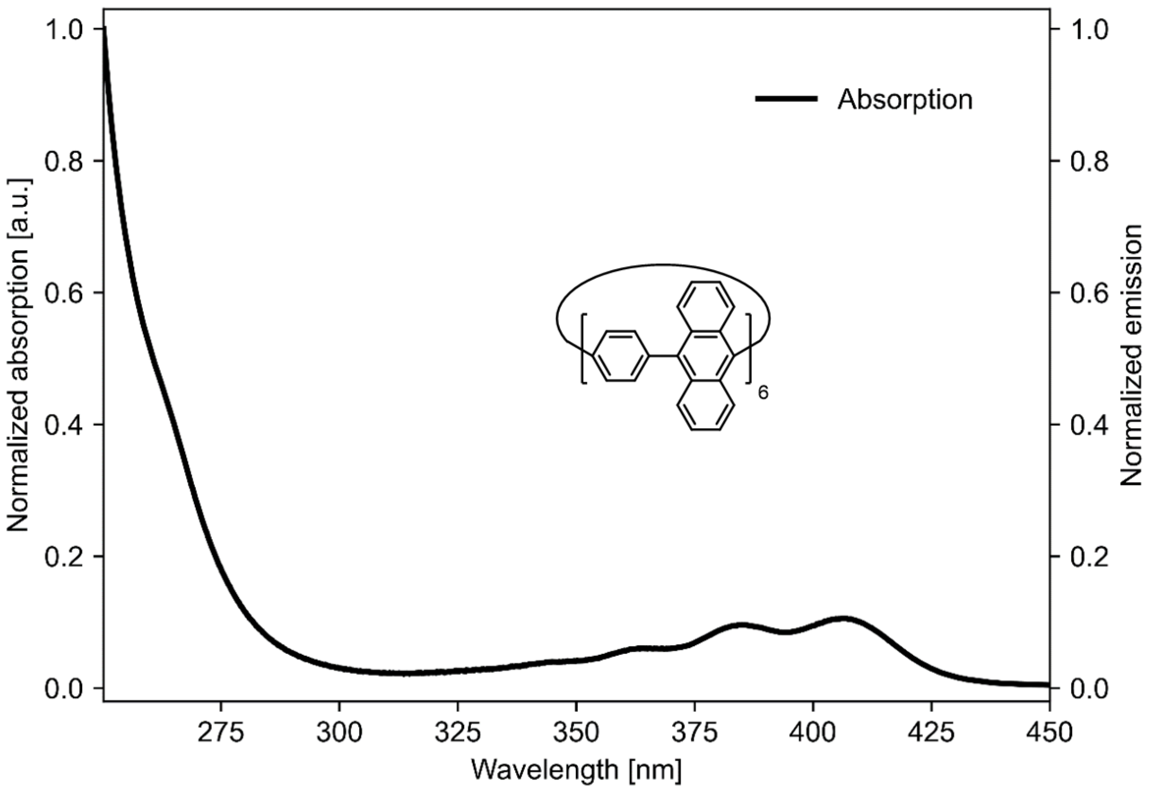

Figure S100: UV/Vis spectrum of **[6.6]CAPP** in dichloromethane (with traces of DMF).

3.6 PXRD

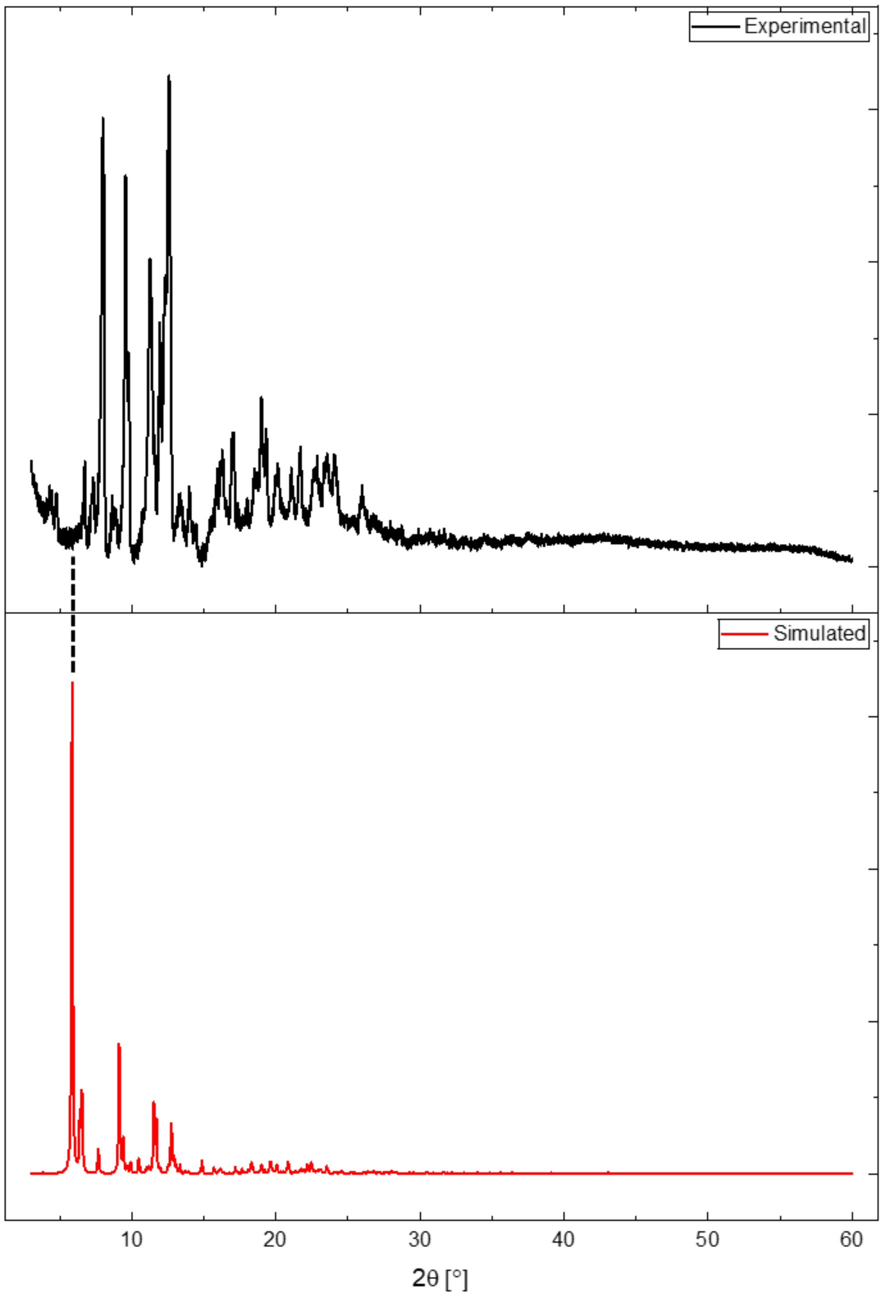

Figure S101: Baseline corrected PXRD pattern of **[6.6]CAPP-OTES** after the gas sorption measurements and calculated pattern from its SCXRD data (after manually removing *o*DCB molecules). The loss of the signal at $2\theta = 6°$, indicates a phase transition or reorganization of the crystalline lattice to an unknown crystalline phase during activation.

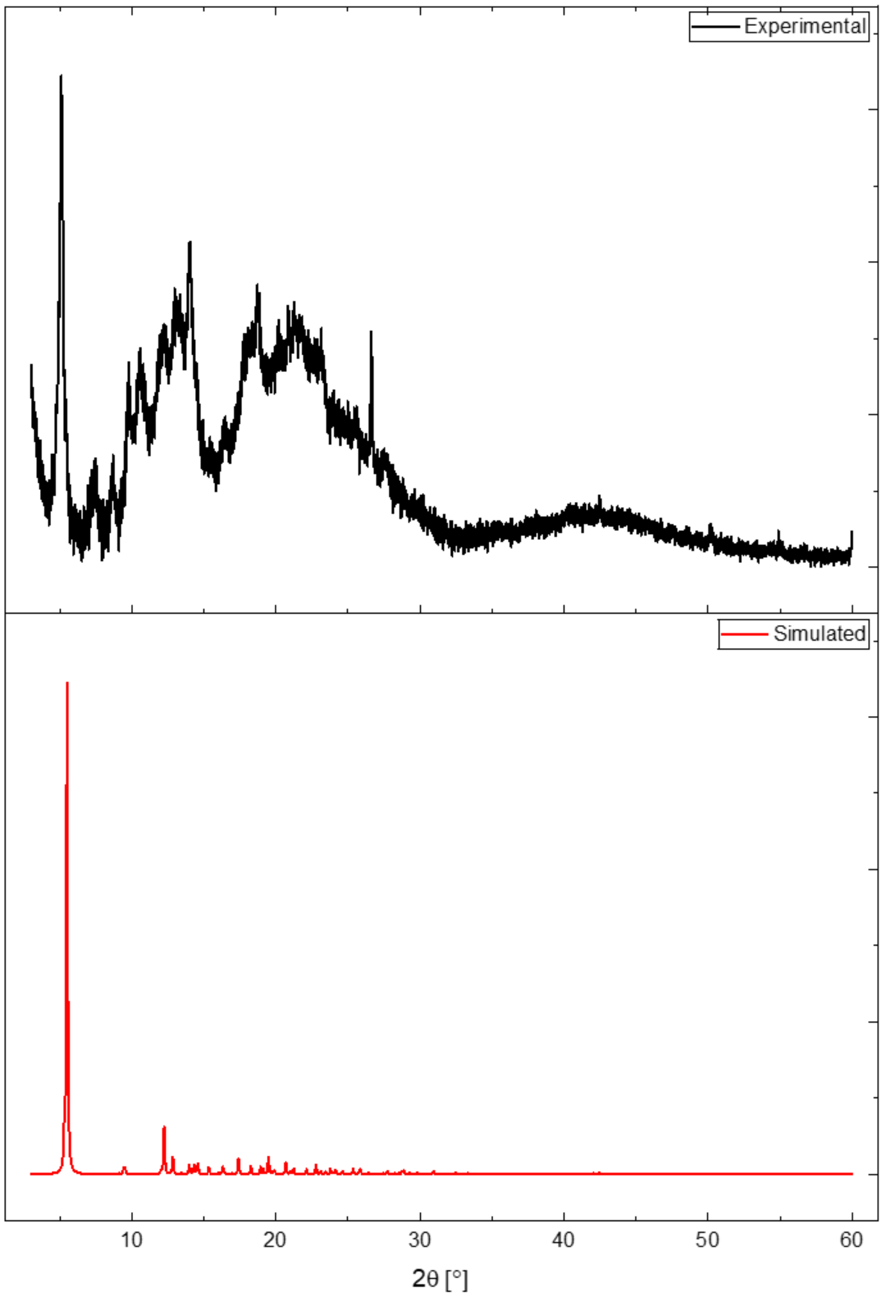

Figure S102: Baseline corrected PXRD pattern of **[6.6]CAPP-OH** after the gas sorption measurements and calculated pattern from its SCXRD data. The low signal to noise ratio, indicates a barely crystalline phase.

4 Crystallographic data

Crystallographic data for compound 2:

Crystals suitable for X-ray diffraction were obtained by slowly evaporating a solution of **2** in chloroform.

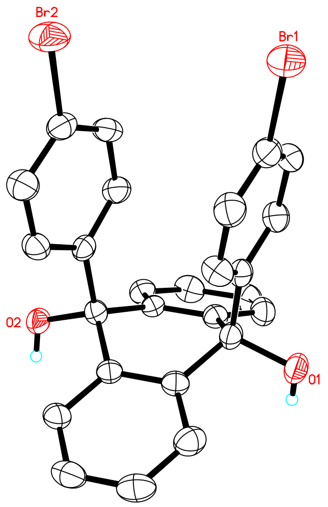

CCDC	2516805	
Empirical formula	$C_{27}H_{19}Br_2Cl_3O_2$	
Formula weight	641.59	
Temperature	200(2) K	
Wavelength	1.54178 Å	
Crystal system	orthorhombic	
Space group	*P*bcn	
Z	8	
Unit cell dimensions	*a* = 16.4300(5) Å	α = 90 deg.
	b = 13.6886(5) Å	β = 90 deg.
	c = 22.2456(6) Å	γ = 90 deg.
Volume	5003.1(3) $Å^3$	
Density (calculated)	1.70 g/cm^3	
Absorption coefficient	7.25 mm^{-1}	
Crystal shape	hexagonal plate	
Crystal size	0.086 x 0.050 x 0.041 mm^3	
Crystal colour	colourless	
θ range for data collection	4.0 to 68.3 deg.	
Index ranges	$-18 \leq h \leq 18$, $-16 \leq k \leq 15$, $-25 \leq l \leq 10$	
Reflections collected	17435	
Independent reflections	4364 (*R*(int) = 0.0350)	
Observed reflections	3176 (I > 2σ(I))	
Absorption correction	Semi-empirical from equivalents	
Max. and min. transmission	0.84 and 0.65	
Refinement method	Full-matrix least-squares on F^2	
Data/restraints/parameters	4364 / 464 / 335	
Goodness-of-fit on F^2	0.98	
Final *R* indices (I>2σ(I))	*R*1 = 0.032, *wR*2 = 0.071	
Largest diff. peak and hole	0.42 and -0.44 $eÅ^{-3}$	

Crystallographic data for compound 4:

Crystals suitable for X-ray diffraction were obtained by layering a solution of **4** in $CDCl_3$ with methanol.

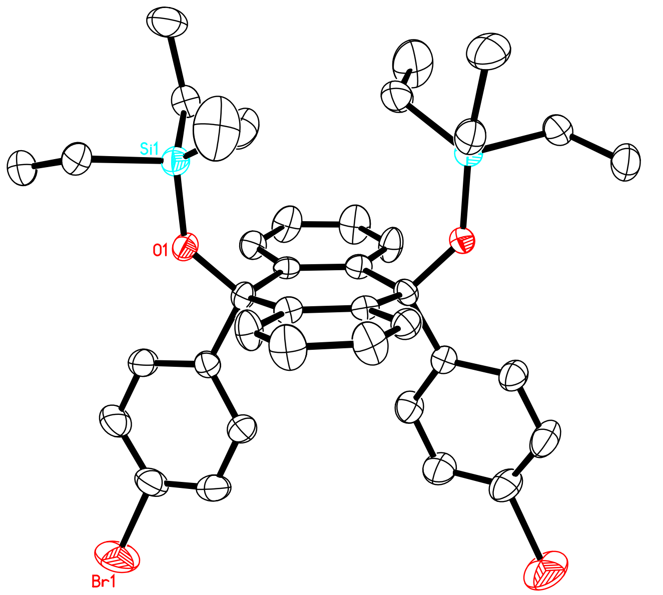

CCDC	2516806	
Empirical formula	$C_{38}H_{46}Br_2O_2Si_2$	
Formula weight	750.75	
Temperature	200(2) K	
Wavelength	0.71073 Å	
Crystal system	monoclinic	
Space group	*C*2/c	
Z	4	
Unit cell dimensions	a = 11.2561(11) Å	α = 90 deg.
	b = 15.1858(14) Å	β = 101.912(3) deg.
	c = 21.553(2) Å	γ = 90 deg.
Volume	3604.8(6) $Å^3$	
Density (calculated)	1.38 g/cm^3	
Absorption coefficient	2.35 mm^{-1}	
Crystal shape	brick	
Crystal size	0.049 x 0.045 x 0.042 mm^3	
Crystal colour	colourless	
θ range for data collection	1.9 to 25.0 deg.	
Index ranges	$-13 \le h \le 13$, $-18 \le k \le 17$, $-17 \le l \le 25$	
Reflections collected	16684	
Independent reflections	3195 (R(int) = 0.1052)	
Observed reflections	1773 (I > 2σ(I))	
Absorption correction	Semi-empirical from equivalents	
Max. and min. transmission	0.93 and 0.85	
Refinement method	Full-matrix least-squares on F^2	
Data/restraints/parameters	3195 / 203 / 217	
Goodness-of-fit on F^2	1.01	
Final R indices (I>2σ(I))	$R1$ = 0.052, $wR2$ = 0.083	
Largest diff. peak and hole	0.46 and -0.60 e$Å^{-3}$	

Crystallographic data for compound [6.6]CAPP-OTES:

Crystals suitable for X-ray diffraction were obtained by layering a solution of **[6.6]CAPP-OTES** in *o*DCB with methanol.

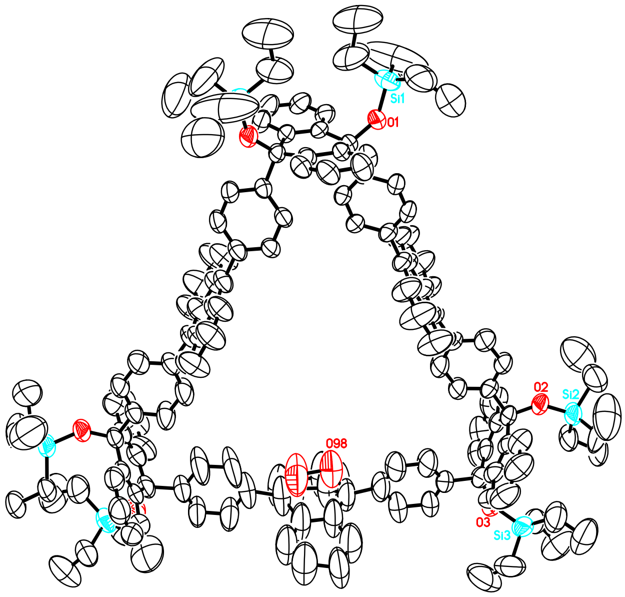

Identification code	2516807	
Empirical formula	$C_{168}H_{170}Cl_4O_{7.09}Si_6$	
Formula weight	2612.89	
Temperature	260(2) K	
Wavelength	1.54178 Å	
Crystal system	monoclinic	
Space group	*C*2/c	
Z	4	
Unit cell dimensions	*a* = 35.6543(7) Å	α = 90 deg.
	b = 30.2075(6) Å	β =91.978(2) deg.
	c = 16.9936(4) Å	γ = 90 deg.
Volume	18291.7(7) Å^3	
Density (calculated)	0.95 g/cm^3	
Absorption coefficient	1.32 mm^{-1}	
Crystal shape	brick	
Crystal size	0.133 x 0.072 x 0.068 mm^3	
Crystal colour	colourless blue fl	
θ range for data collection	3.2 to 53.4 deg.	
Index ranges	$-36 \leq h \leq 37$, $-28 \leq k \leq 31$, $-16 \leq l \leq 17$	
Reflections collected	53906	
Independent reflections	10765 (*R*(int) = 0.0610)	
Observed reflections	6582 (I > 2σ(I))	
Absorption correction	Semi-empirical from equivalents	
Max. and min. transmission	0.96 and 0.85	
Refinement method	Full-matrix least-squares on F^2	
Data/restraints/parameters	10765 / 1350 / 890	
Goodness-of-fit on F^2	1.79	
Final *R* indices (I>2σ(I))	*R*1 = 0.103, *wR*2 = 0.274	
Largest diff. peak and hole	0.58 and -0.34 eÅ^{-3}	

In the crystal structure of **[6.6]CAPP-OTES**, each macrocycle can be populated either by **[6.6]CAPP-OTES** or its one-fold peroxidized congener (see below). This was modelled with a population of 41% **[6.6]CAPP-OTES** and 59% one-fold peroxide.

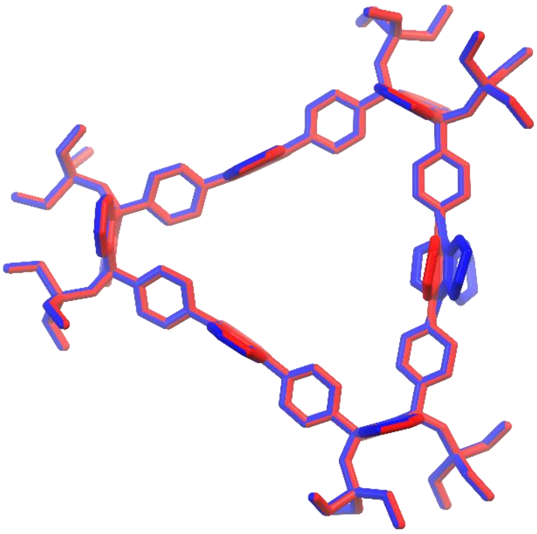

.

Figure S103: Overlay of **[6.6]CAPP-OTES** (red) and its one-fold peroxide (blue).

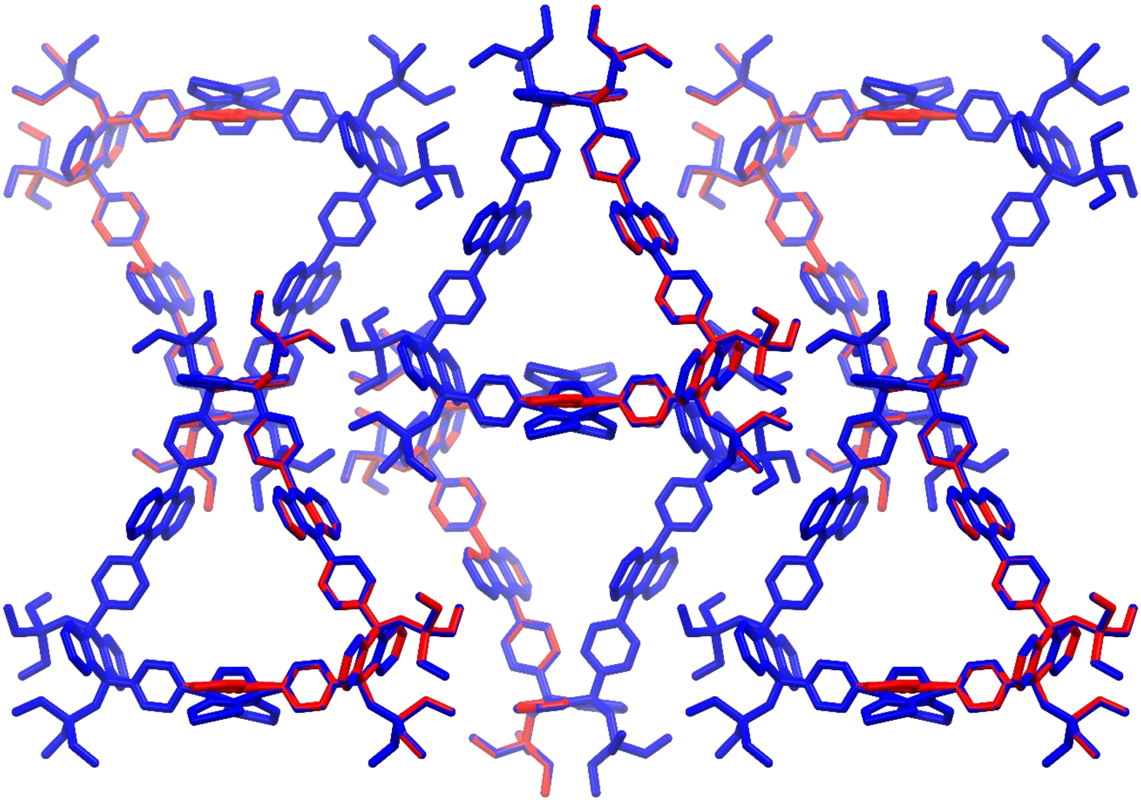

Figure S104: Overlay of the packing of **[6.6]CAPP-OTES** (red) and its one-fold peroxide (blue).

Crystallographic data for compound [8.8]CAPP-OTES:

Crystals suitable for X-ray diffraction were obtained by layering a solution of **[8.8]CAPP-OTES** in in *o*DCB with methanol.

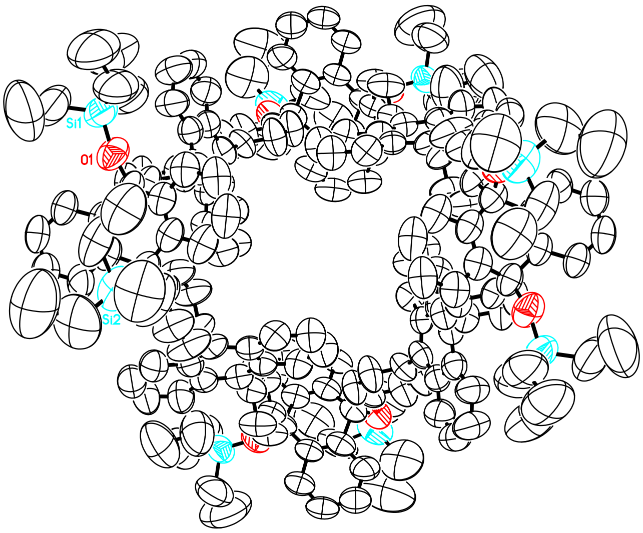

CCDC	2516808	
Empirical formula	$C_{208}H_{216}O_8Si_8$	
Formula weight	3068.52	
Temperature	200(2) K	
Wavelength	1.54178 Å	
Crystal system	tetragonal	
Space group	*I*&^-&B4	
Z	2	
Unit cell dimensions	*a* = 25.9475(8) Å	α = 90 deg.
	b = 25.9475(8) Å	β = 90 deg.
	c = 14.5259(8) Å	γ = 90 deg.
Volume	9779.9(8) $Å^3$	
Density (calculated)	1.04 g/cm^3	
Absorption coefficient	0.92 mm^{-1}	
Crystal shape	irregular	
Crystal size	0.140 x 0.075 x 0.060 mm^3	
Crystal colour	colourless	
θ range for data collection	3.5 to 55.1 deg.	
Index ranges	$-27 \le h \le 23$, $-21 \le k \le 27$, $-15 \le l \le 14$	
Reflections collected	10629	
Independent reflections	5430 (*R*(int) = 0.0253)	
Observed reflections	3951 (I > 2σ(I))	
Absorption correction	Semi-empirical from equivalents	
Max. and min. transmission	0.96 and 0.79	
Refinement method	Full-matrix least-squares on F^2	
Data/restraints/parameters	5430 / 1203 / 510	
Goodness-of-fit on F^2	1.05	
Final *R* indices (I>2σ(I))	*R*1 = 0.105, *wR*2 = 0.273	
Absolute structure parameter	0.38(2)	
Largest diff. peak and hole	0.27 and -0.25 e$Å^{-3}$	

Crystallographic data for compound [10.10]CAPP-OTES:

Crystals suitable for X-ray diffraction were obtained by layering a solution of **[10.10]CAPP-OTES** in in *o*DCB with methanol, which co-crystallized with its one-fold peroxidized congener. This was modelled with a population of 52% **[10.10]CAPP-OTES** and 48% one-fold peroxide, which could not be separated. Thus, 20% ellipsoids are shown.

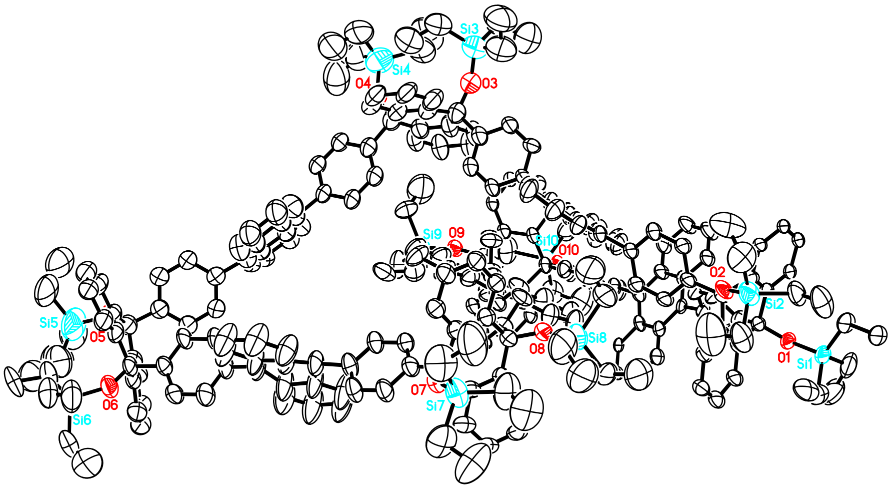

CCDC	2516809	
Empirical formula	$C_{260}H_{270}O_{10.93}Si_{10}$	
Formula weight	3850.48	
Temperature	200(2) K	
Wavelength	1.54178 Å	
Crystal system	triclinic	
Space group	$P\bar{1}$	
Z	2	
Unit cell dimensions	a = 14.7927(8) Å	α =93.244(6) deg.
	b = 26.872(2) Å	β =95.513(5) deg.
	c = 34.437(2) Å	γ =99.397(5) deg.
Volume	13406.4(15) $Å^3$	
Density (calculated)	0.95 g/cm^3	
Absorption coefficient	0.84 mm^{-1}	
Crystal shape	pole	
Crystal size	0.520 x 0.040 x 0.034 mm^3	
Crystal colour	colourless	
θ range for data collection	2.6 to 36.7 deg.	
Index ranges	$-11\leq h\leq 8$, $-20\leq k\leq 20$, $-26\leq l\leq 26$	
Reflections collected	39904	
Independent reflections	12781 (R(int) = 0.0864)	
Observed reflections	6869 ($I > 2\sigma(I)$)	
Absorption correction	Semi-empirical from equivalents	
Max. and min. transmission	0.97 and 0.91	
Refinement method	Full-matrix least-squares on F^2	
Data/restraints/parameters	12781 / 11730 / 2170	
Goodness-of-fit on F^2	2.92	
Final R indices ($I>2\sigma(I)$)	$R1$ = 0.196, $wR2$ = 0.463	
Largest diff. peak and hole	0.61 and -0.37 $eÅ^{-3}$	

Crystallographic data for compound [12.12]CAPP-OTES:

Crystals suitable for X-ray diffraction were obtained by layering a solution of **[12.12]CAPP-OTES** in in *o*DCB with methanol.

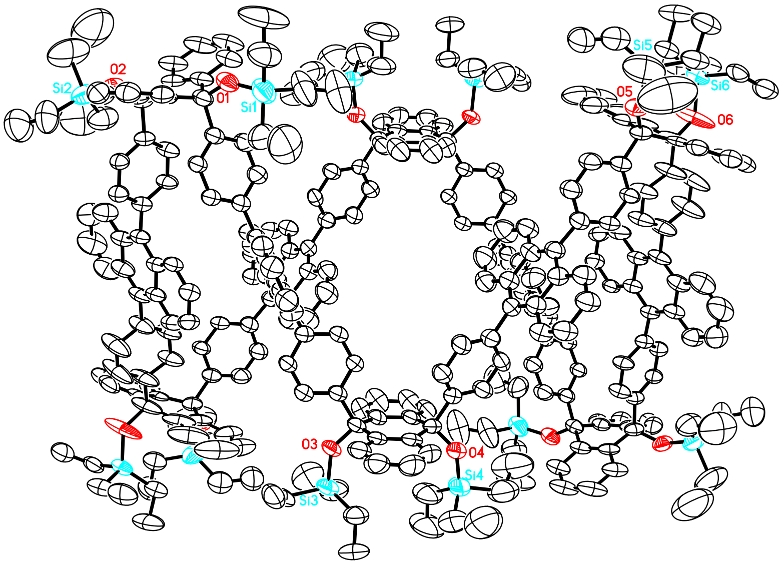

Identification code	2516810	
Empirical formula	$C_{396}H_{380}Cl_{28}O_{12}Si_{12}$	
Formula weight	6660.66	
Temperature	200(2) K	
Wavelength	1.54178 Å	
Crystal system	triclinic	
Space group	$P\bar{1}$	
Z	1	
Unit cell dimensions	a = 21.7268(7) Å	α =105.693(3) deg.
	b = 23.1185(8) Å	β =104.424(3) deg.
	c = 24.7378(8) Å	γ =114.212(3) deg.
Volume	9950.7(7) $Å^3$	
Density (calculated)	1.11 g/cm^3	
Absorption coefficient	2.51 mm^{-1}	
Crystal shape	prism	
Crystal size	0.182 x 0.075 x 0.037 mm^3	
Crystal colour	colourless	
θ range for data collection	4.9 to 51.9 deg.	
Index ranges	$-22 \le h \le 12$, $-23 \le k \le 23$, $-24 \le l \le 25$	
Reflections collected	62055	
Independent reflections	21764 (R(int) = 0.0632)	
Observed reflections	11628 ($I > 2\sigma(I)$)	
Absorption correction	Semi-empirical from equivalents	
Max. and min. transmission	0.97 and 0.79	
Refinement method	Full-matrix least-squares on F^2	
Data/restraints/parameters	21764 / 5919 / 1997	
Goodness-of-fit on F^2	2.00	
Final R indices ($I>2\sigma(I)$)	$R1$ = 0.137, $wR2$ = 0.338	
Largest diff. peak and hole	1.01 and -0.61 $eÅ^{-3}$	

Crystallographic data for compound [14.14]CAPP-OTES:

Crystals suitable for X-ray diffraction were obtained by layering a solution of **[14.14]CAPP-OTES** in in *o*DCB with methanol.

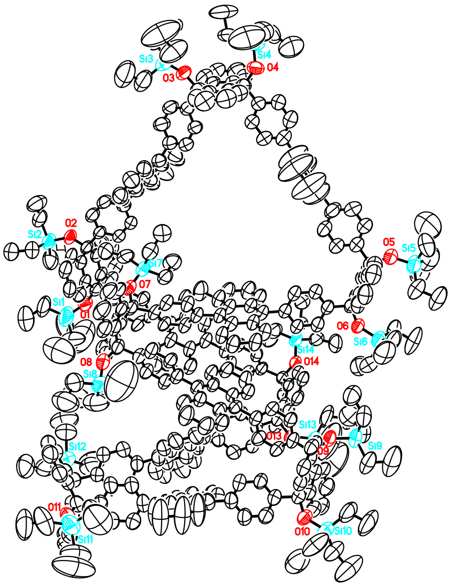

CCDC	2516811	
Empirical formula	$C_{370}H_{382}Cl_2O_{14}Si_{14}$	
Formula weight	5516.89	
Temperature	200(2) K	
Wavelength	1.54178 Å	
Crystal system	triclinic	
Space group	$P\bar{1}$	
Z	2	
Unit cell dimensions	*a* = 21.0454(3) Å	α =71.498(2) deg.
	b = 27.2519(5) Å	β =76.304(1) deg.
	c = 37.2947(7) Å	γ =76.772(1) deg.
Volume	19431.1(6) $Å^3$	
Density (calculated)	0.94 g/cm^3	
Absorption coefficient	0.94 mm^{-1}	
Crystal shape	brick	
Crystal size	0.190 x 0.150 x 0.056 mm^3	
Crystal colour	colourless	
θ range for data collection	4.7 to 53.4 deg.	
Index ranges	$-12 \leq h \leq 21$, $-25 \leq k \leq 28$, $-38 \leq l \leq 38$	
Reflections collected	146635	
Independent reflections	45625 (*R*(int) = 0.0588)	
Observed reflections	29081 ($I > 2\sigma(I)$)	
Absorption correction	Semi-empirical from equivalents	
Max. and min. transmission	0.96 and 0.74	
Refinement method	Full-matrix least-squares on F^2	
Data/restraints/parameters	45625 / 21509 / 3589	
Goodness-of-fit on F^2	2.23	
Final *R* indices ($I>2\sigma(I)$)	*R*1 = 0.127, *wR*2 = 0.338	
Largest diff. peak and hole	1.07 and -0.70 $eÅ^{-3}$	

Crystallographic data for compound [16.16]CAPP-OTES:

Crystals suitable for X-ray diffraction were obtained by layering a solution of **[16.16]CAPP-OTES** in in *o*DCB with methanol.

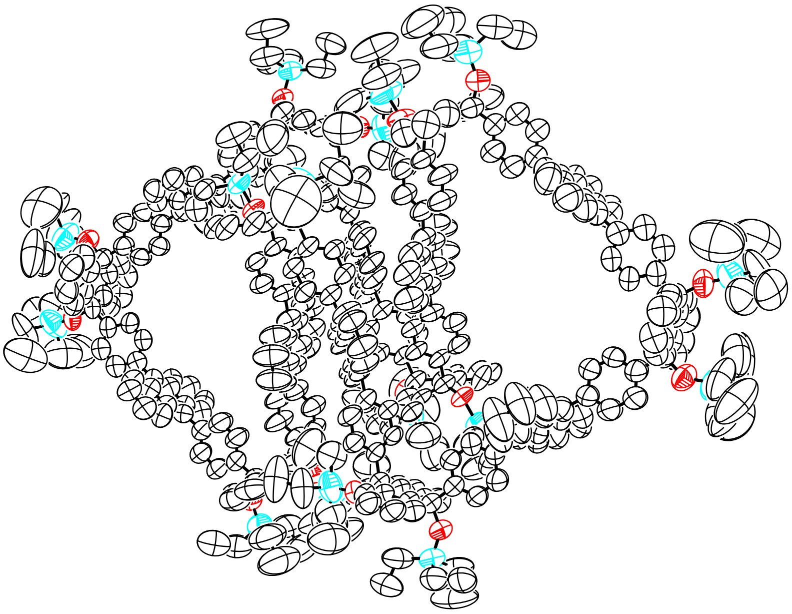

CCDC	2516812	
Empirical formula	$C_{416}H_{432}O_{16}Si_{16}$	
Formula weight	6137.03	
Temperature	240(2) K	
Wavelength	1.54178 Å	
Crystal system	triclinic	
Space group	$P\bar{1}$	
Z	1	
Unit cell dimensions	*a* = 18.3019(8) Å	α =109.922(3) deg.
	b = 24.5214(10) Å	β =99.663(3) deg.
	c = 30.5167(12) Å	γ =97.945(3) deg.
Volume	12407.9(9) $Å^3$	
Density (calculated)	0.82 g/cm^3	
Absorption coefficient	0.73 mm^{-1}	
Crystal shape	lanceolate	
Crystal size	0.195 x 0.102 x 0.035 mm^3	
Crystal colour	colourless	
θ range for data collection	4.4 to 41.6 deg.	
Index ranges	$-15 \le h \le 15$, $-21 \le k \le 21$, $-26 \le l \le 21$	
Reflections collected	55198	
Independent reflections	16477 (*R*(int) = 0.0638)	
Observed reflections	9261 ($I > 2\sigma(I)$)	
Absorption correction	Semi-empirical from equivalents	
Max. and min. transmission	0.98 and 0.72	
Refinement method	Full-matrix least-squares on F^2	
Data/restraints/parameters	16477 / 7928 / 2017	
Goodness-of-fit on F^2	2.93	
Final *R* indices ($I>2\sigma(I)$)	*R*1 = 0.194, *wR*2 = 0.477	
Largest diff. peak and hole	0.98 and -0.48 e$Å^{-3}$	

Crystallographic data for compound [6.6]CAPP-OTES-O_2:

Crystals suitable for X-ray diffraction were obtained by layering a solution of **[6.6]CAPP-OTES-O_2**in in chloroform with acetonitrile.

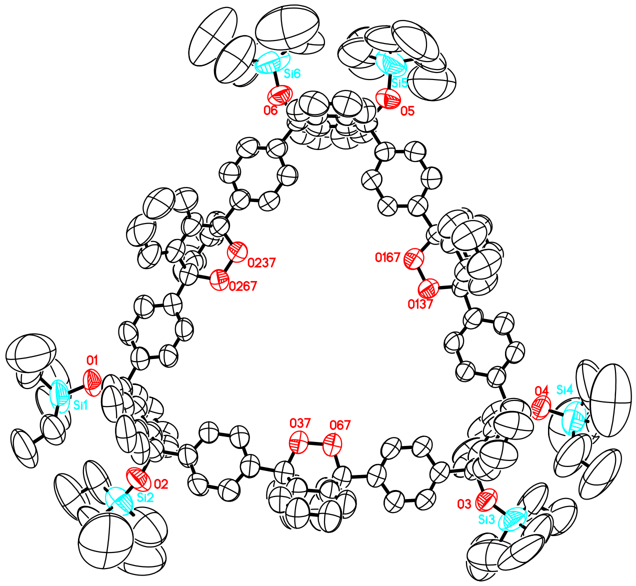

Identification code	2521404	
Empirical formula	$C_{156}H_{162}O_{12}Si_6$	
Formula weight	2397.39	
Temperature	240(2) K	
Wavelength	1.54178 Å	
Crystal system	monoclinic	
Space group	*C*2/c	
Z	8	
Unit cell dimensions	a = 42.5657(9) Å	α = 90 deg.
	b = 23.8246(5) Å	β =113.332(2) deg.
	c = 34.4279(9) Å	γ = 90 deg.
Volume	32058.6(13) Å^3	
Density (calculated)	0.99 g/cm^3	
Absorption coefficient	0.89 mm^{-1}	
Crystal shape	prism	
Crystal size	0.167 x 0.102 x 0.056 mm^3	
Crystal colour	colourless	
θ range for data collection	4.5 to 50.4 deg.	
Index ranges	$-39 \le h \le 42$, $-21 \le k \le 23$, $-34 \le l \le 34$	
Reflections collected	62513	
Independent reflections	16710 (R(int) = 0.0347)	
Observed reflections	10677 ($I > 2\sigma(I)$)	
Absorption correction	Semi-empirical from equivalents	
Max. and min. transmission	0.96 and 0.83	
Refinement method	Full-matrix least-squares on F^2	
Data/restraints/parameters	16710 / 5274 / 1567	
Goodness-of-fit on F^2	2.12	
Final R indices ($I > 2\sigma(I)$)	$R1$ = 0.113, $wR2$ = 0.307	
Largest diff. peak and hole	0.54 and -0.61 eÅ^{-3}	

Crystallographic data for compound [6.6]CAPP-OH:

Crystals suitable for X-ray diffraction were obtained by layering a solution of **[6.6]CAPP-OH** in in THF with *n*-pentane.

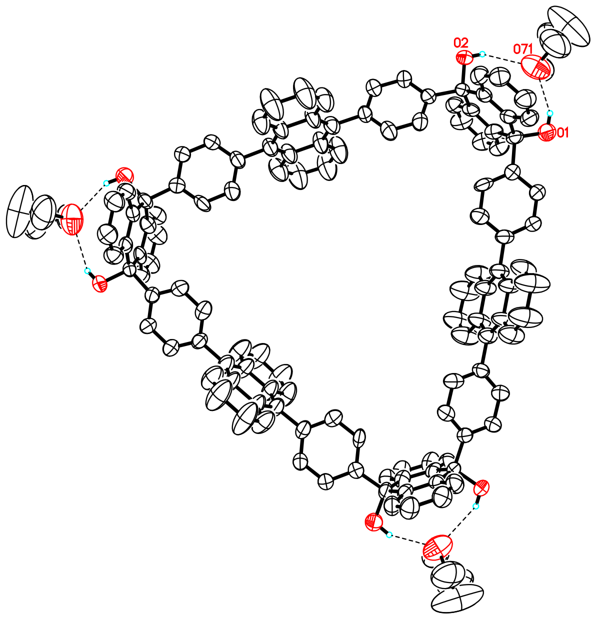

CCDC	2516813	
Empirical formula	$C_{264}H_{204}O_{18}$	
Formula weight	3664.26	
Temperature	200(2) K	
Wavelength	1.54178 Å	
Crystal system	trigonal	
Space group	$R\overline{3}$	
Z	3	
Unit cell dimensions	*a* = 32.1136(10) Å	α = 90 deg.
	b = 32.1136(10) Å	β = 90 deg.
	c = 20.6652(7) Å	γ = 120 deg.
Volume	18456.4(13) $Å^3$	
Density (calculated)	0.99 g/cm^3	
Absorption coefficient	0.48 mm^{-1}	
Crystal shape	rhombic	
Crystal size	0.047 x 0.040 x 0.023 mm^3	
Crystal colour	colourless	
θ range for data collection	3.8 to 48.5 deg.	
Index ranges	$-31 \leq h \leq 29$, $-26 \leq k \leq 31$, $-20 \leq l \leq 11$	
Reflections collected	15133	
Independent reflections	3884 (*R*(int) = 0.1199)	
Observed reflections	1689 ($I > 2\sigma(I)$)	
Absorption correction	Semi-empirical from equivalents	
Max. and min. transmission	0.99 and 0.88	
Refinement method	Full-matrix least-squares on F^2	
Data/restraints/parameters	3884 / 396 / 426	
Goodness-of-fit on F^2	0.93	
Final *R* indices ($I>2\sigma(I)$)	*R*1 = 0.076, *wR*2 = 0.171	
Largest diff. peak and hole	0.30 and -0.18 $eÅ^{-3}$	

5 Computational details

5.1 Strain Calculations

Strain energies of CPPs and their derivatives are commonly calculated by hypothetical homodesmotic reactions.[S64-65] In these reactions the macrocycle reacts with an endcap to from a linear oligomer matching the number of each hybridization and the number of carbon atoms with x C-H bonds between the starting materials and products. From this the strain energy can be calculated by:

$$E_{strain} = \sum E_{starting\ materials} - \sum E_{products} = E_{macrocycle} + E_{endcap} - E_{linear\ Oligomer}$$

<u>Homodesmotic reaction for **[6.6]CAPP-OH**:</u>

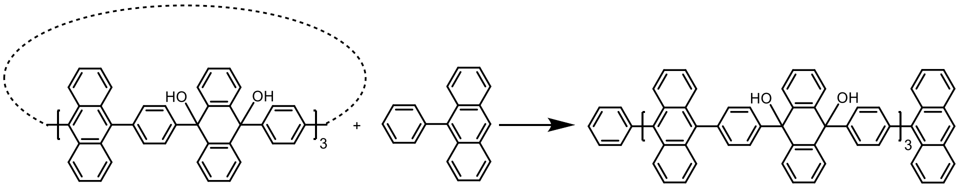

[6.6]CAPP-OH **S1** **S2**

Figure S105: Homodesmotic reaction for **[6.6]CAPP-OH**.

E_{strain} = $E_{[6.6]CAPP\text{-}OH}$+E_{S1}-E_{S2} = -5072.17290085 a.u.+(-770,73359732 a.u.)-(-5842.90857246 a.u.)=0,00207429 a.u.= 1.30 kcal/mol

<u>Homodesmotic reaction for **[6.6]CAPP**:</u>

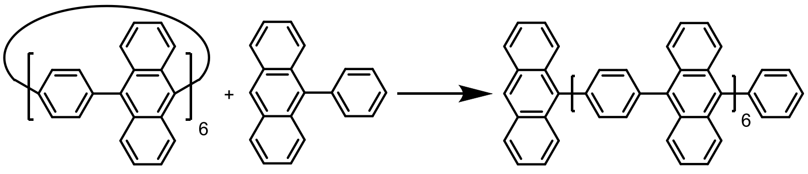

[6.6]CAPP **S1** **S3**

Figure S106: Homodesmotic reaction for **[6.6]CAPP**.

E_{strain} = $E_{[6.6]CAPP}$+E_{S1}-E_{S3} = -4617.09245886 a.u.+(-770.73359732 a.u.)-(-5387.92002481 a.u.) = 0.09396863 a.u.= 59.00 kcal/mol

5.2 TDDFT:

[6.6]CAPP-OH:

[6.6]CAPP-OH was used as benchmark molecule for TDDFT calculations of the absorption spectrum with different functionals (see below). As PBE1PBE matched the most red-shifted absorption of the experimental spectrum the closest, it was used for the calculation of the absorption of **[6.6]CAPP**.

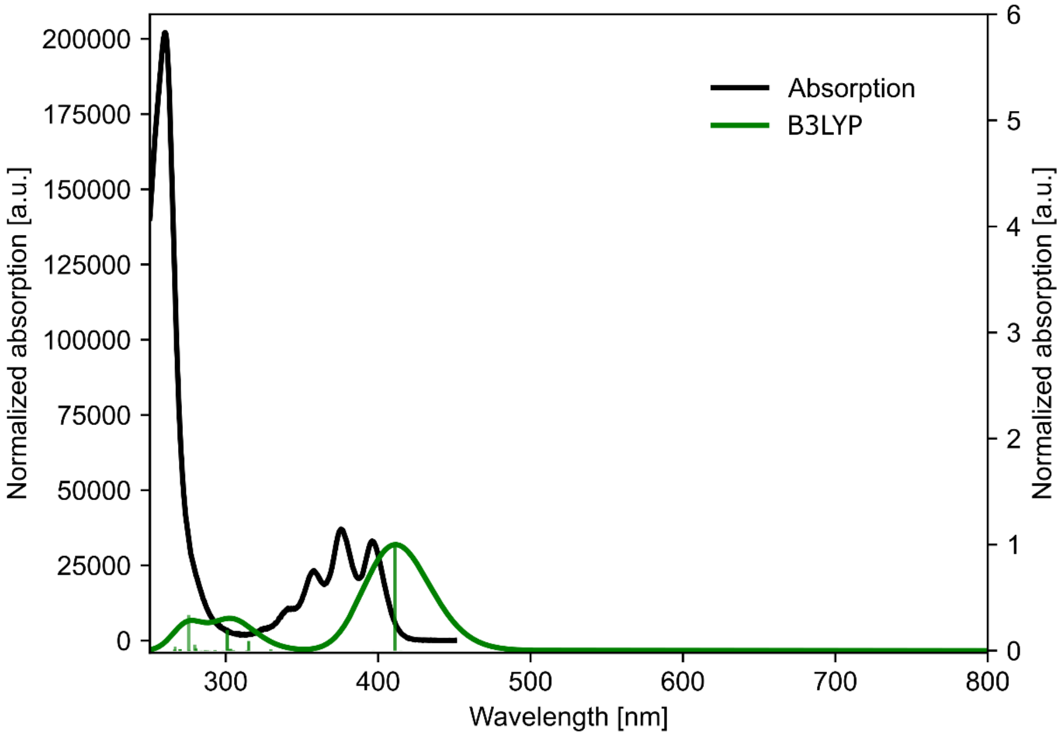

Figure S107: Experimental and TDDFT calculated (B3LYP-GD3BJ/6-311G(d) with THF solvation), with the corresponding osc. strengths as bar graph) UV/Vis spectra of **[6.6]CAPP-OH** in tetrahydrofuran.

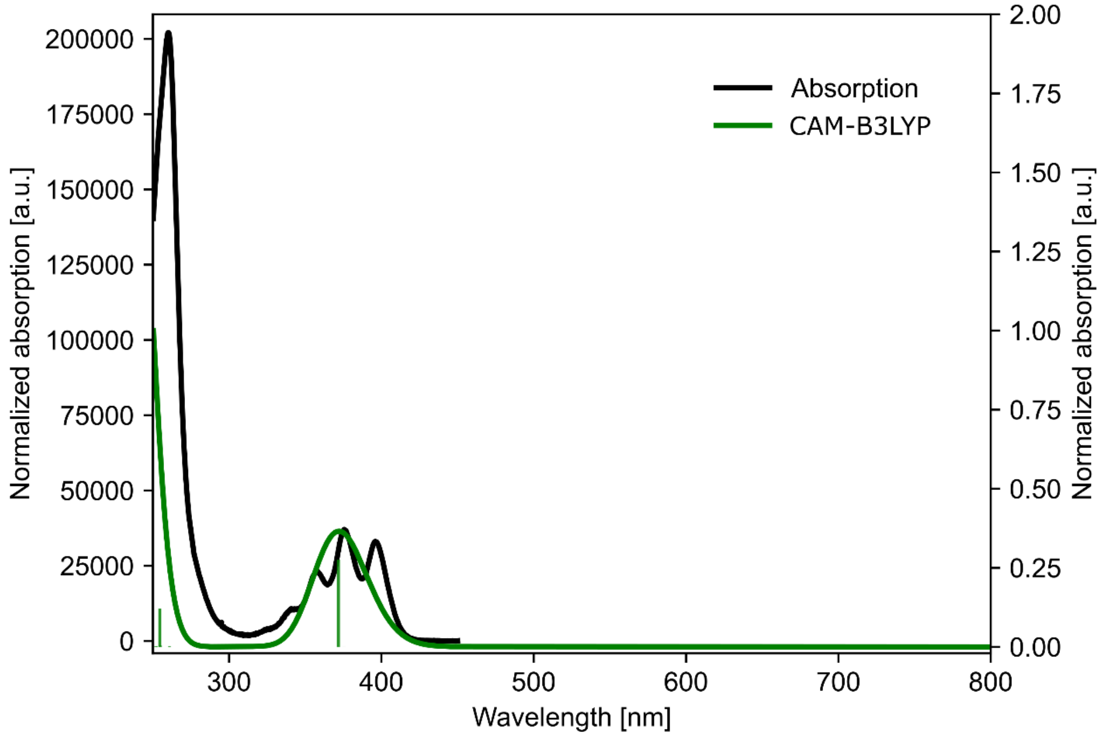

Figure S108: Experimental and TDDFT calculated (CAM-B3LYP-GD3BJ/6-311G(d) with THF solvation), with the corresponding osc. strengths as bar graph) UV/Vis spectra of **[6.6]CAPP-OH** in tetrahydrofuran.

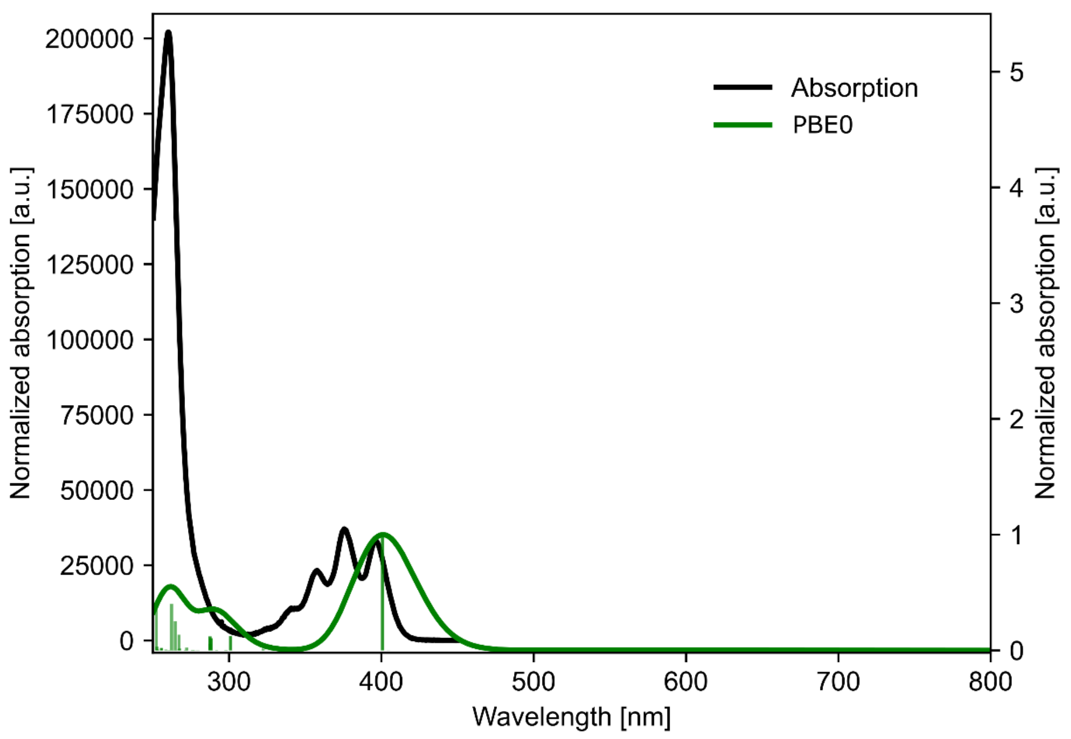

Figure S109: Experimental and TDDFT calculated (PBE0-GD3BJ/6-311G(d) with THF solvation), with the corresponding osc. strengths as bar graph) UV/Vis spectra of **[6.6]CAPP-OH** in tetrahydrofuran.

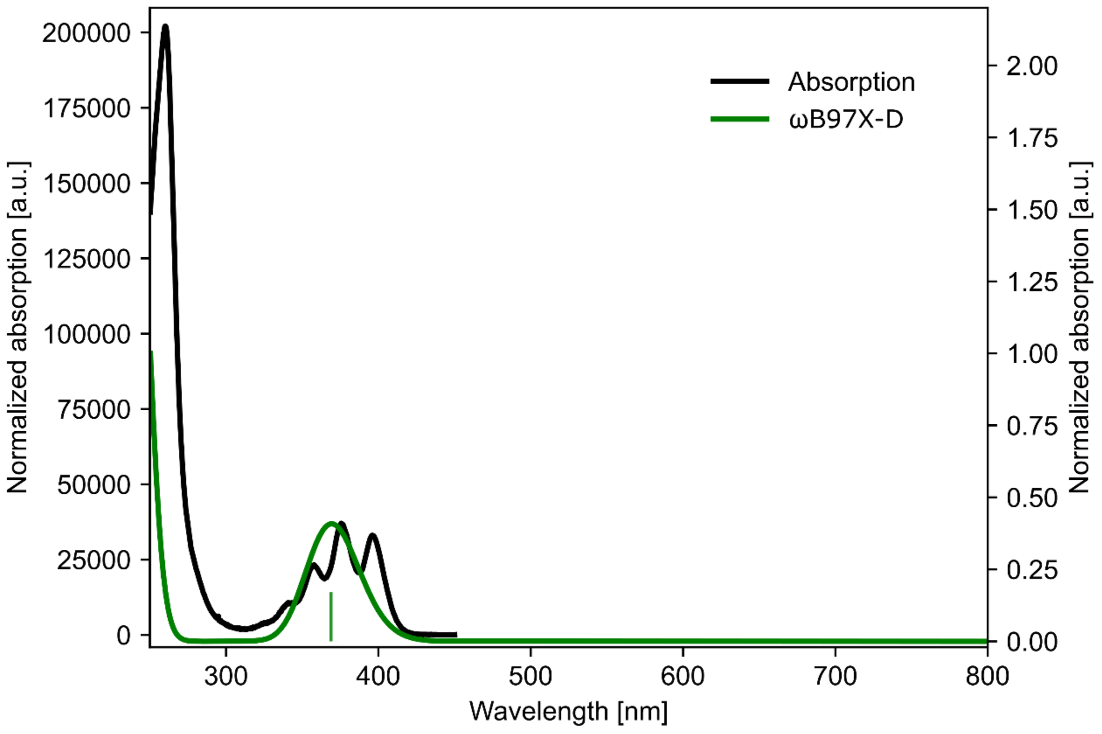

Figure S110: Experimental and TDDFT calculated (ωB97X-D/6-311G(d) with THF solvation), with the corresponding osc. strengths as bar graph) UV/Vis spectra of **[6.6]CAPP-OH** in tetrahydrofuran.

[6.6]CAPP:

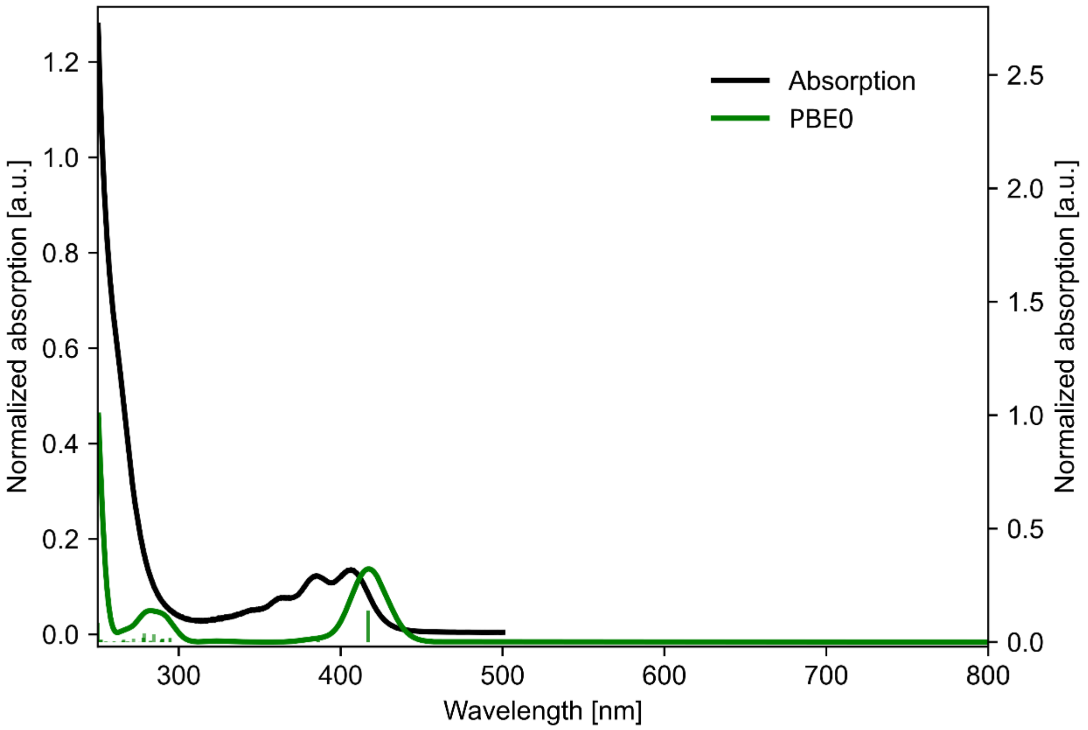

Figure S111: Experimental and TDDFT calculated (PBE0-GD3BJ/6-311G(d) with DCM solvation), with the corresponding osc. strengths as bar graph) UV/Vis spectra of **[6.6]CAPP** in dichloromethane.

5.3 XYZ-Coordinates

[6.6]CAPP-OH (B3LYP-GD3BJ/6-311G(d)):

Atom	x	y	z
C	-6.782114	1.454284	0.631907
C	-5.547436	2.094562	0.592475
C	-5.435679	3.406141	0.122700
C	-6.600563	4.060630	-0.289694
C	-7.832730	3.418630	-0.263053
C	-7.935123	2.099189	0.182199
C	-9.434996	0.435044	1.319590
C	-9.401790	-0.954175	1.207383
C	-9.415970	-0.745525	-1.319140
C	-9.428717	0.644034	-1.206934
C	-9.561460	-1.339271	-2.580784
C	-9.718926	-0.565429	-3.718089
C	-9.734405	0.827226	-3.607654
C	-9.591829	1.420084	-2.364113
C	-9.599548	1.023662	2.581295
C	-9.730854	0.245039	3.718660
C	-9.700269	-1.147367	3.608223
C	-9.538650	-1.735188	2.364623
C	-5.475440	-2.276082	-0.593191
C	-6.730551	-1.676825	-0.632346
C	-7.861579	-2.359342	-0.182315
C	-7.715675	-3.674691	0.262898
C	-6.463020	-4.275745	0.289278
C	-5.320416	-3.583226	-0.123345
C	-3.987674	-4.243569	-0.066523
C	-3.326615	-4.610215	-1.254703
C	-2.053115	-5.285776	-1.189218
C	-1.499843	-5.625483	0.061111
C	-2.159717	-5.256661	1.249307
C	-3.406176	-4.532436	1.183779
C	-1.641821	-5.597913	2.538349
C	-2.272950	-5.206621	3.684529
C	-3.468951	-4.443780	3.619839
C	-4.017707	-4.123814	2.410860
C	-3.890788	-4.352132	-2.543654
C	-3.225862	-4.682511	-3.689899
C	-1.946226	-5.294810	-3.625337
C	-1.383833	-5.590318	-2.416400
C	2.128516	-6.597152	0.628513
C	0.956537	-5.848344	0.588262
C	-0.234882	-6.407827	0.118196
C	-0.218653	-7.743994	-0.293836
C	0.953617	-8.489794	-0.266376
C	2.147076	-7.918341	0.179397
C	4.156367	-8.483346	-1.206899
C	5.354322	-7.778768	-1.316796
C	5.523771	-7.663174	1.210116
C	4.336456	-8.385527	1.320041
C	6.266537	-7.391681	2.368792
C	5.835518	-7.825159	3.611569
C	4.644180	-8.546942	3.719718
C	3.906205	-8.821985	2.580939
C	3.566860	-9.010943	-2.365370
C	4.153572	-8.837658	-3.607914
C	5.352950	-8.129308	-3.716020
C	5.943319	-7.607592	-2.577447
C	6.938943	-3.458938	0.293704
C	7.042935	-4.844476	0.267464
C	5.975977	-5.627096	-0.178302
C	4.820457	-4.987337	-0.628935
C	4.713662	-3.600659	-0.589898
C	5.769006	-2.814163	-0.119650
C	5.676060	-1.329714	-0.063236
C	5.636489	-0.681227	1.186848
C	5.640729	0.760368	1.251832
C	5.629278	1.515797	0.063368

C	5.611015	0.866361	-1.186719	C	-2.332045	5.183271	-1.249863
C	5.662632	-0.574321	-1.251704	C	-3.553968	4.418367	-1.184404
C	5.539302	1.597823	-2.414116	C	-4.125461	4.110111	0.065872
C	5.563786	0.962581	-3.622839	C	-3.476720	4.498036	1.254088
C	5.673284	-0.451790	-3.686956	C	-2.226190	5.215223	1.188653
C	5.720352	-1.192413	-2.540473	C	-4.031907	4.221116	2.543032
C	5.588904	-1.414657	2.414239	C	-3.378108	4.572962	3.689292
C	5.592557	-0.778959	3.622965	C	-2.119393	5.227195	3.624759
C	5.655522	0.638246	3.687081	C	-1.567194	5.541402	2.415839
C	5.678178	1.380020	2.540598	C	-1.825769	5.541702	-2.538859
C	4.653541	5.143224	0.628960	C	-2.443830	5.130200	-3.685081
C	4.592554	3.753773	0.589963	C	-3.614121	4.328459	-3.620477
C	5.673295	3.002503	0.119798	C	-4.151904	3.990249	-2.411533
C	6.821373	3.685504	-0.293457	H	-6.841817	0.447382	1.023960
C	6.879609	5.073717	-0.267230	H	-4.660041	1.572738	0.934306
C	5.787370	5.820730	0.178411	H	-6.535122	5.086878	-0.635035
C	5.268433	7.840930	-1.209837	H	-8.725455	3.947295	-0.568213
C	4.057958	8.523761	-1.319732	H	-9.556954	-2.420543	-2.643789
C	3.874619	8.615274	1.207226	H	-9.828210	-1.039746	-4.687617
C	5.095180	7.950620	1.317089	H	-9.857316	1.445276	-4.490840
C	3.267943	9.122914	2.365753	H	-9.615057	2.497960	-2.270675
C	3.859984	8.968893	3.608303	H	-9.630792	2.104492	2.644298
C	5.082100	8.300528	3.716369	H	-9.855384	0.715483	4.688236
C	5.689447	7.798791	2.577747	H	-9.802282	-1.769140	4.491457
C	6.019804	7.594209	-2.368505	H	-9.526223	-2.813243	2.271186
C	5.574773	8.013374	-3.611255	H	-4.605786	-1.725314	-0.935207
C	4.360276	8.695474	-3.719379	H	-6.823466	-0.672452	-1.024424
C	3.613590	8.945914	-2.580604	H	-8.590438	-4.232482	0.568227
C	0.763217	5.876822	-0.588561	H	-6.363728	-5.299267	0.634632
C	1.909960	6.663734	-0.628574	H	-0.730477	-6.178716	2.594707
C	1.885040	7.984774	-0.179324	H	-1.858343	-5.475751	4.650455
C	0.673381	8.516698	0.266259	H	-3.948842	-4.117137	4.536553
C	-0.473764	7.732779	0.293489	H	-4.930955	-3.544697	2.367904
C	-0.445999	6.396831	-0.118612	H	-4.865403	-3.885193	-2.599843
C	-1.684548	5.573293	-0.061637	H	-3.673997	-4.473710	-4.655777

H	-1.415911	-5.530631	-4.542130
H	-0.409534	-6.059518	-2.373568
H	3.030036	-6.145046	1.020878
H	0.964312	-4.818821	0.929786
H	-1.139848	-8.200848	-0.639397
H	0.942546	-9.527374	-0.571140
H	7.194525	-6.842554	2.277105
H	6.423436	-7.603037	4.495948
H	4.296878	-8.889685	4.688640
H	2.985194	-9.388676	2.642169
H	2.644115	-9.568832	-2.273726
H	3.680543	-9.251785	-4.492139
H	5.819945	-7.986581	-4.684755
H	6.878174	-7.064038	-2.638622
H	7.776280	-2.862280	0.639528
H	7.962525	-5.324369	0.573447
H	3.996587	-5.568929	-1.021467
H	3.802662	-3.121714	-0.932397
H	5.458559	2.676216	-2.371633
H	5.502251	1.539421	-4.539804
H	5.715918	-0.944641	-4.652681
H	5.803212	-2.269944	-2.596335
H	5.543644	-2.495122	2.371740
H	5.550048	-1.357511	4.539931
H	5.682002	1.132230	4.652804
H	5.725607	2.459691	2.596460
H	3.810931	5.697334	1.021474
H	3.697829	3.245057	0.932436
H	7.677976	3.116780	-0.639166
H	7.782917	5.583671	-0.573095
H	2.327282	9.650038	2.274149
H	3.373477	9.367006	4.492569
H	5.553496	8.173148	4.685112
H	6.641733	7.286385	2.638886
H	6.965398	7.075988	-2.276827
H	6.169737	7.810871	-4.495632
H	4.001896	9.026678	-4.688281
H	2.674400	9.481943	-2.641814
H	0.804840	4.848130	-0.930143
H	2.825917	6.241548	-1.020833
H	0.628171	9.553343	0.571050
H	-1.409523	8.159076	0.638963
H	-4.990569	3.722241	2.599207
H	-3.818998	4.349231	4.655161
H	-1.597056	5.480167	4.541560
H	-0.608931	6.042543	2.373019
H	-0.933990	6.152131	-2.595132
H	-2.038399	5.413064	-4.650978
H	-4.083147	3.986516	-4.537235
H	-5.045633	3.381433	-2.368643
C	3.162668	8.827228	-0.128970
C	5.825971	7.350573	0.126539
C	6.064911	-7.154843	-0.126285
C	3.451676	-8.718396	0.129276
C	-9.230139	-1.674568	-0.130088
C	-9.280399	1.369708	0.130472
O	-10.280656	2.403195	0.207790
H	-11.140434	1.974524	0.118674
O	-10.195780	-2.740499	-0.206990
H	-11.069205	-2.340443	-0.117529
O	3.056814	-10.101477	0.205317
H	3.858136	-10.631595	0.117146
O	7.470506	-7.460325	-0.200936
H	7.559126	-8.416912	-0.111486
O	7.220742	7.702110	0.201271
H	7.277844	8.661101	0.111890
O	2.722607	10.196617	-0.204858
H	3.506079	10.752756	-0.116560

[6.6]CAPP-OH (B3LYP/6-311G(d))**:**

Atom	x	y	z
C	1.740778	6.796616	0.594212
C	2.427380	5.585189	0.592922
C	3.752223	5.509044	0.151451
C	4.369276	6.688807	-0.275452
C	3.682778	7.900575	-0.279452
C	2.351845	7.969800	0.143321
C	0.673202	9.461169	1.307190
C	-0.722196	9.452511	1.230742
C	-0.576540	9.467210	-1.307325
C	0.818697	9.444497	-1.230870
C	-1.195292	9.643740	-2.555467
C	-0.447510	9.791593	-3.711762
C	0.947339	9.766420	-3.637725
C	1.566779	9.597113	-2.410212
C	1.293702	9.631706	2.555298
C	0.547446	9.787319	3.711562
C	-0.847586	9.776169	3.637535
C	-1.468703	9.612873	2.410055
C	-2.370071	5.609450	-0.592184
C	-1.671073	6.813764	-0.593456
C	-2.270314	7.993285	-0.143225
C	-3.602071	7.937831	0.278970
C	-4.300951	6.733157	0.274998
C	-3.695842	5.547026	-0.151338
C	-4.447559	4.252910	-0.140524
C	-5.229627	3.880223	-1.251860
C	-5.961957	2.634387	-1.234280
C	-5.891311	1.792158	-0.106989
C	-5.105165	2.162512	1.002651
C	-4.375228	3.409431	0.986212
C	-5.003560	1.333265	2.165630
C	-4.241198	1.700431	3.238310
C	-3.526627	2.928210	3.223693
C	-3.593383	3.752093	2.135864
C	-5.324966	4.705713	-2.417912
C	-6.087114	4.338221	-3.490662
C	-6.809779	3.115087	-3.471928
C	-6.747786	2.293915	-2.381750
C	-6.795725	-1.880636	-0.515571
C	-6.089643	-0.680994	-0.560206
C	-6.654326	0.504885	-0.080518
C	-7.956898	0.452314	0.425266
C	-8.662808	-0.746956	0.474678
C	-8.084736	-1.936791	0.021108
C	-8.584055	-4.167118	-1.066752
C	-7.875503	-5.369446	-0.993208
C	-7.832292	-5.181850	1.542481
C	-8.519191	-3.967123	1.467302
C	-7.605848	-5.769194	2.797843
C	-8.049265	-5.165263	3.962446
C	-8.736573	-3.951165	3.889588
C	-8.967253	-3.365763	2.655474
C	-9.098725	-3.746184	-2.303729
C	-8.912286	-4.498907	-3.451203
C	-8.201014	-5.699402	-3.379875
C	-7.693488	-6.125320	-2.163340
C	-3.701734	-7.145405	-0.128845
C	-5.093301	-7.130185	-0.064912
C	-5.779128	-5.966075	0.293057
C	-5.030077	-4.829116	0.608614
C	-3.640493	-4.844479	0.537131
C	-2.949974	-6.002343	0.160873
C	-1.456976	-6.016983	0.079670
C	-0.684566	-6.031692	1.259646
C	0.758432	-6.019283	1.179556
C	1.395518	-6.031411	-0.078860
C	0.623000	-6.038543	-1.258838
C	-0.819801	-6.011536	-1.178751

C	1.227533	-6.080338	-2.556539
C	0.474701	-6.058595	-3.696362
C	-0.941569	-5.988616	-3.617787
C	-1.563915	-5.968132	-2.401763
C	-1.289492	-6.067112	2.557351
C	-0.536477	-6.052753	3.697170
C	0.880430	-5.997127	3.618583
C	1.502951	-5.983178	2.402555
C	4.980528	-4.880067	-0.608082
C	3.590861	-4.881235	-0.536489
C	2.888585	-6.031945	-0.160058
C	3.628667	-7.182572	0.129803
C	5.020306	-7.181569	0.065761
C	5.717955	-6.024580	-0.292466
C	7.778795	-5.261720	-1.542452
C	8.478149	-4.054099	-1.467680
C	8.541536	-4.254226	1.066383
C	7.820642	-5.449223	0.993264
C	9.060862	-3.838382	2.303135
C	8.867024	-4.588940	3.450803
C	8.143423	-5.782067	3.379901
C	7.631205	-6.202971	2.163586
C	7.546070	-5.846986	-2.797636
C	7.995441	-5.247908	-3.962461
C	8.695240	-4.040941	-3.890008
C	8.932170	-3.457663	-2.656071
C	6.082470	-0.743130	0.560127
C	6.776340	-1.949877	0.515411
C	8.064488	-2.019195	-0.021798
C	8.654368	-0.835373	-0.475898
C	7.960677	0.370999	-0.426394
C	6.658943	0.436880	0.079991
C	5.909084	1.731862	0.106668
C	5.125464	2.109630	-1.002253
C	4.408335	3.363956	-0.985670
C	4.490610	4.207276	0.140511
C	5.270117	3.827207	1.251147
C	5.989643	2.573932	1.233418
C	5.375162	4.652269	2.416667
C	6.134710	4.277568	3.488767
C	6.844820	3.047106	3.469876
C	6.773221	2.226043	2.380203
C	5.014109	1.280854	-2.164672
C	4.254345	1.655218	-3.236706
C	3.552402	2.890257	-3.221969
C	3.628791	3.713995	-2.134663
H	0.724635	6.821948	0.969071
H	1.930310	4.687828	0.947396
H	5.402621	6.658683	-0.606663
H	4.189855	8.803535	-0.592908
H	-2.277687	9.675745	-2.594702
H	-0.944465	9.925975	-4.667177
H	1.546642	9.881067	-4.535329
H	2.647765	9.594768	-2.347501
H	2.376364	9.652795	2.594528
H	1.045731	9.916885	4.666950
H	-1.445698	9.897009	4.535121
H	-2.549659	9.621388	2.347337
H	-1.882070	4.706939	-0.946182
H	-0.654543	6.828589	-0.967837
H	-4.100009	8.846036	0.591923
H	-5.334713	6.713728	0.605709
H	-5.546570	0.396444	2.184648
H	-4.179355	1.052642	4.107055
H	-2.925098	3.210487	4.081983
H	-3.046932	4.687102	2.132269
H	-4.775785	5.638968	-2.439278
H	-6.142051	4.981704	-4.363120
H	-7.412506	2.833629	-4.329698
H	-7.300184	1.362314	-2.374469

H	-6.335924	-2.773920	-0.920699
H	-5.088290	-0.664584	-0.978552
H	-8.424912	1.363485	0.784593
H	-9.677554	-0.756888	0.850077
H	-7.085938	-6.719138	2.836861
H	-7.863689	-5.634700	4.923196
H	-9.091695	-3.466471	4.793449
H	-9.514267	-2.433371	2.595739
H	-9.660309	-2.820196	-2.340633
H	-9.316972	-4.155919	-4.398001
H	-8.046866	-6.299434	-4.271067
H	-7.156298	-7.063658	-2.100344
H	-3.190276	-8.062444	-0.404116
H	-5.650795	-8.033458	-0.274689
H	-5.527461	-3.922078	0.930291
H	-3.082466	-3.946676	0.782879
H	2.306818	-6.131585	-2.625426
H	0.959185	-6.091541	-4.667154
H	-1.530289	-5.952731	-4.529035
H	-2.644094	-5.915217	-2.351103
H	-2.369243	-6.107424	2.626241
H	-1.021268	-6.080602	4.667968
H	1.469483	-5.967017	4.529824
H	2.583610	-5.941204	2.351879
H	5.487112	-3.978193	-0.929926
H	3.042019	-3.977810	-0.782324
H	3.107900	-8.094311	0.405226
H	5.568563	-8.090465	0.275608
H	9.631944	-2.918208	2.339700
H	9.275490	-4.249959	4.397421
H	7.983366	-6.380319	4.271248
H	7.084401	-7.135762	2.100914
H	7.016402	-6.791537	-2.836334
H	7.804845	-5.715618	-4.923071
H	9.055165	-3.560135	-4.794046

H	9.488763	-2.530938	-2.596648
H	5.081527	-0.716489	0.978928
H	6.307695	-2.838385	0.920915
H	9.668802	-0.855642	-0.851724
H	8.437745	1.277309	-0.786110
H	4.835532	5.591075	2.438164
H	6.197162	4.920891	4.360837
H	7.445583	2.759940	4.327131
H	7.316036	1.288829	2.372822
H	5.547528	0.338541	-2.183786
H	4.184930	1.007644	-4.105040
H	2.952865	3.178234	-4.079760
H	3.091981	4.654570	-2.131009
C	8.812613	-3.359379	-0.140694
C	7.256305	-6.009705	-0.318000
C	-7.317257	-5.935426	0.318299
C	-8.846345	-3.269344	0.140092
C	-1.488283	9.318095	-0.091669
C	1.583382	9.302527	0.091567
O	2.602414	10.322856	0.137303
H	2.160395	11.176798	0.054611
O	-2.496834	10.348773	-0.137604
H	-2.046117	11.198172	-0.055076
O	-10.241333	-2.900926	0.152261
H	-10.752931	-3.709032	0.281497
O	-7.719679	-7.316840	0.422258
H	-8.684009	-7.339839	0.395131
O	7.644493	-7.395198	-0.421762
H	8.608543	-7.428086	-0.394890
O	10.211238	-3.005035	-0.153234
H	10.714649	-3.818271	-0.282455

[6.6]CAPP (B3LYP-GD3BJ/6-311G(d))**:**

Atom	x	y	z
C	3.053315	6.672362	-1.004412
C	4.251777	5.980429	-1.004412
C	5.290463	6.338119	-0.134063
C	5.136196	7.498107	0.629202
C	3.925453	8.197130	0.629202
C	2.843741	7.750735	-0.134063
C	1.424370	8.177777	0.039504
C	0.722564	7.726612	1.179770
C	-0.722564	7.726612	1.179770
C	-1.424370	8.177777	0.039504
C	-0.721257	8.765539	-1.027337
C	0.721257	8.765539	-1.027337
C	-1.400682	9.319426	-2.155801
C	-0.710547	9.850739	-3.208458
C	0.710547	9.850739	-3.208458
C	1.400682	9.319426	-2.155801
C	1.395494	7.159693	2.307917
C	0.709001	6.659832	3.376897
C	-0.709001	6.659832	3.376897
C	-1.395494	7.159693	2.307917
C	-4.251777	5.980429	-1.004412
C	-3.053315	6.672362	-1.004412
C	-2.843741	7.750735	-0.134063
C	-3.925453	8.197130	0.629202
C	-5.136196	7.498107	0.629202
C	-5.290463	6.338119	-0.134063
C	-6.369978	5.322429	0.039504
C	-7.230551	5.007396	-1.027337
C	-7.951808	3.758143	-1.027337
C	-7.794348	2.855348	0.039504
C	-7.052724	3.237547	1.179770
C	-6.330160	4.489065	1.179770
C	-6.898223	2.371313	2.307917
C	-6.122084	2.715903	3.376897
C	-5.413083	3.943929	3.376897
C	-5.502729	4.788380	2.307917
C	-7.370519	5.872739	-2.155801
C	-8.175717	5.540721	-3.208458
C	-8.886264	4.310018	-3.208458
C	-8.771201	3.446687	-2.155801
C	-7.305092	-0.691933	-1.004412
C	-7.305092	0.691933	-1.004412
C	-8.134204	1.412616	-0.134063
C	-9.061649	0.699023	0.629202
C	-9.061649	-0.699023	0.629202
C	-8.134204	-1.412616	-0.134063
C	-7.794348	-2.855348	0.039504
C	-7.951808	-3.758143	-1.027337
C	-7.230551	-5.007396	-1.027337
C	-6.369978	-5.322429	0.039504
C	-6.330160	-4.489065	1.179770
C	-7.052724	-3.237547	1.179770
C	-5.502729	-4.788380	2.307917
C	-5.413083	-3.943929	3.376897
C	-6.122084	-2.715903	3.376897
C	-6.898223	-2.371313	2.307917
C	-8.771201	-3.446687	-2.155801
C	-8.886264	-4.310018	-3.208458
C	-8.175717	-5.540721	-3.208458
C	-7.370519	-5.872739	-2.155801
C	-3.925453	-8.197130	0.629202
C	-5.136196	-7.498107	0.629202
C	-5.290463	-6.338119	-0.134063
C	-4.251777	-5.980429	-1.004412
C	-3.053315	-6.672362	-1.004412
C	-2.843741	-7.750735	-0.134063
C	-1.424370	-8.177777	0.039504
C	-0.722564	-7.726612	1.179770

C	0.722564	-7.726612	1.179770
C	1.424370	-8.177777	0.039504
C	0.721257	-8.765539	-1.027337
C	-0.721257	-8.765539	-1.027337
C	1.400682	-9.319426	-2.155801
C	0.710547	-9.850739	-3.208458
C	-0.710547	-9.850739	-3.208458
C	-1.400682	-9.319426	-2.155801
C	-1.395494	-7.159693	2.307917
C	-0.709001	-6.659832	3.376897
C	0.709001	-6.659832	3.376897
C	1.395494	-7.159693	2.307917
C	4.251777	-5.980429	-1.004412
C	3.053315	-6.672362	-1.004412
C	2.843741	-7.750735	-0.134063
C	3.925453	-8.197130	0.629202
C	5.136196	-7.498107	0.629202
C	5.290463	-6.338119	-0.134063
C	6.369978	-5.322429	0.039504
C	7.230551	-5.007396	-1.027337
C	7.951808	-3.758143	-1.027337
C	7.794348	-2.855348	0.039504
C	7.052724	-3.237547	1.179770
C	6.330160	-4.489065	1.179770
C	6.898223	-2.371313	2.307917
C	6.122084	-2.715903	3.376897
C	5.413083	-3.943929	3.376897
C	5.502729	-4.788380	2.307917
C	7.370519	-5.872739	-2.155801
C	8.175717	-5.540721	-3.208458
C	8.886264	-4.310018	-3.208458
C	8.771201	-3.446687	-2.155801
C	7.305092	0.691933	-1.004412
C	7.305092	-0.691933	-1.004412
C	8.134204	-1.412616	-0.134063
C	9.061649	-0.699023	0.629202
C	9.061649	0.699023	0.629202
C	8.134204	1.412616	-0.134063
C	7.794348	2.855348	0.039504
C	7.951808	3.758143	-1.027337
C	7.230551	5.007396	-1.027337
C	6.369978	5.322429	0.039504
C	6.330160	4.489065	1.179770
C	7.052724	3.237547	1.179770
C	5.502729	4.788380	2.307917
C	5.413083	3.943929	3.376897
C	6.122084	2.715903	3.376897
C	6.898223	2.371313	2.307917
C	8.771201	3.446687	-2.155801
C	8.886264	4.310018	-3.208458
C	8.175717	5.540721	-3.208458
C	7.370519	5.872739	-2.155801
H	2.232410	6.329416	-1.624389
H	4.365230	5.098032	-1.624389
H	5.942546	7.821529	1.279179
H	3.802370	9.057160	1.279179
H	-2.484048	9.324318	-2.153541
H	-1.246744	10.280162	-4.048212
H	1.246744	10.280162	-4.048212
H	2.484048	9.324318	-2.153541
H	2.475871	7.127007	2.306688
H	1.248841	6.243491	4.220970
H	-1.248841	6.243491	4.220970
H	-2.475871	7.127007	2.306688
H	-4.365230	5.098032	-1.624389
H	-2.232410	6.329416	-1.624389
H	-3.802370	9.057160	1.279179
H	-5.942546	7.821529	1.279179
H	-7.410105	1.419336	2.306688
H	-6.031442	2.040217	4.220970

H	-4.782601	4.203274	4.220970
H	-4.934234	5.707671	2.306688
H	-6.833072	6.813408	-2.153541
H	-8.279509	6.219793	-4.048212
H	-9.526253	4.060369	-4.048212
H	-9.317120	2.510910	-2.153541
H	-6.597640	-1.231384	-1.624389
H	-6.597640	1.231384	-1.624389
H	-9.744916	1.235631	1.279179
H	-9.744916	-1.235631	1.279179
H	-4.934234	-5.707671	2.306688
H	-4.782601	-4.203274	4.220970
H	-6.031442	-2.040217	4.220970
H	-7.410105	-1.419336	2.306688
H	-9.317120	-2.510910	-2.153541
H	-9.526253	-4.060369	-4.048212
H	-8.279509	-6.219793	-4.048212
H	-6.833072	-6.813408	-2.153541
H	-3.802370	-9.057160	1.279179
H	-5.942546	-7.821529	1.279179
H	-4.365230	-5.098032	-1.624389
H	-2.232410	-6.329416	-1.624389
H	2.484048	-9.324318	-2.153541
H	1.246744	-10.280162	-4.048212
H	-1.246744	-10.280162	-4.048212
H	-2.484048	-9.324318	-2.153541
H	-2.475871	-7.127007	2.306688
H	-1.248841	-6.243491	4.220970
H	1.248841	-6.243491	4.220970
H	2.475871	-7.127007	2.306688
H	4.365230	-5.098032	-1.624389
H	2.232410	-6.329416	-1.624389
H	3.802370	-9.057160	1.279179
H	5.942546	-7.821529	1.279179
H	7.410105	-1.419336	2.306688
H	6.031442	-2.040217	4.220970
H	4.782601	-4.203274	4.220970
H	4.934234	-5.707671	2.306688
H	6.833072	-6.813408	-2.153541
H	8.279509	-6.219793	-4.048212
H	9.526253	-4.060369	-4.048212
H	9.317120	-2.510910	-2.153541
H	6.597640	1.231384	-1.624389
H	6.597640	-1.231384	-1.624389
H	9.744916	-1.235631	1.279179
H	9.744916	1.235631	1.279179
H	4.934234	5.707671	2.306688
H	4.782601	4.203274	4.220970
H	6.031442	2.040217	4.220970
H	7.410105	1.419336	2.306688
H	9.317120	2.510910	-2.153541
H	9.526253	4.060369	-4.048212
H	8.279509	6.219793	-4.048212
H	6.833072	6.813408	-2.153541

[6.6]CAPP (B3LYP/6-311G(d))**:**

Atom	x	y	z
C	2.984127	6.552591	-0.827501
C	4.182647	5.860625	-0.827501
C	5.295641	6.331892	-0.114490
C	5.185507	7.580681	0.506158
C	3.972309	8.281122	0.506158
C	2.835759	7.752106	-0.114490
C	1.428029	8.247629	0.023061
C	0.723877	8.004833	1.225449
C	-0.723877	8.004833	1.225449
C	-1.428029	8.247629	0.023061
C	-0.722293	8.689021	-1.114804
C	0.722293	8.689021	-1.114804
C	-1.399722	9.092685	-2.308564

C	-0.710398	9.490034	-3.419948
C	0.710398	9.490034	-3.419948
C	1.399722	9.092685	-2.308564
C	1.395659	7.657612	2.440956
C	0.709194	7.369939	3.586359
C	-0.709194	7.369939	3.586359
C	-1.395659	7.657612	2.440956
C	-4.182647	5.860625	-0.827501
C	-2.984127	6.552591	-0.827501
C	-2.835759	7.752106	-0.114490
C	-3.972309	8.281122	0.506158
C	-5.185507	7.580681	0.506158
C	-5.295641	6.331892	-0.114490
C	-6.428642	5.360524	0.023061
C	-7.163767	4.970034	-1.114804
C	-7.886059	3.718987	-1.114804
C	-7.856671	2.887105	0.023061
C	-7.294327	3.375520	1.225449
C	-6.570450	4.629313	1.225449
C	-7.329516	2.620129	2.440956
C	-6.737151	3.070789	3.586359
C	-6.027957	4.299149	3.586359
C	-5.933857	5.037483	2.440956
C	-7.174635	5.758537	-2.308564
C	-7.863412	5.360240	-3.419948
C	-8.573810	4.129795	-3.419948
C	-8.574357	3.334148	-2.308564
C	-7.166773	-0.691966	-0.827501
C	-7.166773	0.691966	-0.827501
C	-8.131400	1.420214	-0.114490
C	-9.157816	0.700440	0.506158
C	-9.157816	-0.700440	0.506158
C	-8.131400	-1.420214	-0.114490
C	-7.856671	-2.887105	0.023061
C	-7.886059	-3.718987	-1.114804
C	-7.163767	-4.970034	-1.114804
C	-6.428642	-5.360524	0.023061
C	-6.570450	-4.629313	1.225449
C	-7.294327	-3.375520	1.225449
C	-5.933857	-5.037483	2.440956
C	-6.027957	-4.299149	3.586359
C	-6.737151	-3.070789	3.586359
C	-7.329516	-2.620129	2.440956
C	-8.574357	-3.334148	-2.308564
C	-8.573810	-4.129795	-3.419948
C	-7.863412	-5.360240	-3.419948
C	-7.174635	-5.758537	-2.308564
C	-3.972309	-8.281122	0.506158
C	-5.185507	-7.580681	0.506158
C	-5.295641	-6.331892	-0.114490
C	-4.182647	-5.860625	-0.827501
C	-2.984127	-6.552591	-0.827501
C	-2.835759	-7.752106	-0.114490
C	-1.428029	-8.247629	0.023061
C	-0.723877	-8.004833	1.225449
C	0.723877	-8.004833	1.225449
C	1.428029	-8.247629	0.023061
C	0.722293	-8.689021	-1.114804
C	-0.722293	-8.689021	-1.114804
C	1.399722	-9.092685	-2.308564
C	0.710398	-9.490034	-3.419948
C	-0.710398	-9.490034	-3.419948
C	-1.399722	-9.092685	-2.308564
C	-1.395659	-7.657612	2.440956
C	-0.709194	-7.369939	3.586359
C	0.709194	-7.369939	3.586359
C	1.395659	-7.657612	2.440956
C	4.182647	-5.860625	-0.827501
C	2.984127	-6.552591	-0.827501
C	2.835759	-7.752106	-0.114490

C	3.972309	-8.281122	0.506158
C	5.185507	-7.580681	0.506158
C	5.295641	-6.331892	-0.114490
C	6.428642	-5.360524	0.023061
C	7.163767	-4.970034	-1.114804
C	7.886059	-3.718987	-1.114804
C	7.856671	-2.887105	0.023061
C	7.294327	-3.375520	1.225449
C	6.570450	-4.629313	1.225449
C	7.329516	-2.620129	2.440956
C	6.737151	-3.070789	3.586359
C	6.027957	-4.299149	3.586359
C	5.933857	-5.037483	2.440956
C	7.174635	-5.758537	-2.308564
C	7.863412	-5.360240	-3.419948
C	8.573810	-4.129795	-3.419948
C	8.574357	-3.334148	-2.308564
C	7.166773	0.691966	-0.827501
C	7.166773	-0.691966	-0.827501
C	8.131400	-1.420214	-0.114490
C	9.157816	-0.700440	0.506158
C	9.157816	0.700440	0.506158
C	8.131400	1.420214	-0.114490
C	7.856671	2.887105	0.023061
C	7.886059	3.718987	-1.114804
C	7.163767	4.970034	-1.114804
C	6.428642	5.360524	0.023061
C	6.570450	4.629313	1.225449
C	7.294327	3.375520	1.225449
C	5.933857	5.037483	2.440956
C	6.027957	4.299149	3.586359
C	6.737151	3.070789	3.586359
C	7.329516	2.620129	2.440956
C	8.574357	3.334148	-2.308564
C	8.573810	4.129795	-3.419948
C	7.863412	5.360240	-3.419948
C	7.174635	5.758537	-2.308564
H	2.124277	6.131080	-1.337060
H	4.247532	4.905218	-1.337060
H	6.039974	7.997964	1.029995
H	3.906453	9.229753	1.029995
H	-2.483404	9.100169	-2.310268
H	-1.247794	9.812417	-4.306106
H	1.247794	9.812417	-4.306106
H	2.483404	9.100169	-2.310268
H	2.477094	7.630624	2.447667
H	1.249033	7.125624	4.495714
H	-1.249033	7.125624	4.495714
H	-2.477094	7.630624	2.447667
H	-4.247532	4.905218	-1.337060
H	-2.124277	6.131080	-1.337060
H	-3.906453	9.229753	1.029995
H	-6.039974	7.997964	1.029995
H	-7.846861	1.670086	2.447667
H	-6.795488	2.481118	4.495714
H	-5.546455	4.644507	4.495714
H	-5.369767	5.960538	2.447667
H	-6.639276	6.700775	-2.310268
H	-7.873906	5.986830	-4.306106
H	-9.121700	3.825588	-4.306106
H	-9.122679	2.399394	-2.310268
H	-6.371810	-1.225862	-1.337060
H	-6.371810	1.225862	-1.337060
H	-9.946427	1.231789	1.029995
H	-9.946427	-1.231789	1.029995
H	-5.369767	-5.960538	2.447667
H	-5.546455	-4.644507	4.495714
H	-6.795488	-2.481118	4.495714
H	-7.846861	-1.670086	2.447667
H	-9.122679	-2.399394	-2.310268

H	-9.121700	-3.825588	-4.306106
H	-7.873906	-5.986830	-4.306106
H	-6.639276	-6.700775	-2.310268
H	-3.906453	-9.229753	1.029995
H	-6.039974	-7.997964	1.029995
H	-4.247532	-4.905218	-1.337060
H	-2.124277	-6.131080	-1.337060
H	2.483404	-9.100169	-2.310268
H	1.247794	-9.812417	-4.306106
H	-1.247794	-9.812417	-4.306106
H	-2.483404	-9.100169	-2.310268
H	-2.477094	-7.630624	2.447667
H	-1.249033	-7.125624	4.495714
H	1.249033	-7.125624	4.495714
H	2.477094	-7.630624	2.447667
H	4.247532	-4.905218	-1.337060
H	2.124277	-6.131080	-1.337060
H	3.906453	-9.229753	1.029995
H	6.039974	-7.997964	1.029995
H	7.846861	-1.670086	2.447667
H	6.795488	-2.481118	4.495714
H	5.546455	-4.644507	4.495714
H	5.369767	-5.960538	2.447667
H	6.639276	-6.700775	-2.310268
H	7.873906	-5.986830	-4.306106
H	9.121700	-3.825588	-4.306106
H	9.122679	-2.399394	-2.310268
H	6.371810	1.225862	-1.337060
H	6.371810	-1.225862	-1.337060
H	9.946427	-1.231789	1.029995
H	9.946427	1.231789	1.029995
H	5.369767	5.960538	2.447667
H	5.546455	4.644507	4.495714
H	6.795488	2.481118	4.495714
H	7.846861	1.670086	2.447667
H	9.122679	2.399394	-2.310268
H	9.121700	3.825588	-4.306106
H	7.873906	5.986830	-4.306106
H	6.639276	6.700775	-2.310268

S1 (B3LYP/6-311G(d)):

Atom	x	y	z
C	-2.037245	1.218632	0.000001
C	-0.593329	1.224077	0.000027
C	-0.593285	-1.224089	-0.000051
C	-2.037201	-1.218697	-0.000066
C	0.069898	-2.492834	-0.000106
C	-0.637445	-3.662920	-0.000152
C	-2.060240	-3.652455	-0.000151
C	-2.735296	-2.465322	-0.000113
C	-2.735386	2.465230	0.000026
C	-2.060375	3.652389	0.000083
C	-0.637582	3.662908	0.000124
C	0.069805	2.492847	0.000097
H	1.152819	-2.516232	-0.000115
H	-0.111101	-4.612178	-0.000193
H	-2.603812	-4.591795	-0.000185
H	-3.821372	-2.448252	-0.000119
H	-3.821462	2.448119	-0.000001
H	-2.603984	4.591708	0.000101
H	-0.111273	4.612186	0.000180
H	1.152724	2.516288	0.000134
C	0.109528	0.000005	-0.000001
C	1.606873	0.000027	0.000024
C	2.321784	0.000331	-1.203520
C	2.321739	-0.000265	1.203598
C	3.715304	0.000350	-1.204292
H	1.778469	0.000554	-2.143173
C	3.715256	-0.000241	1.204424
H	1.778384	-0.000504	2.143229

C	4.416022	0.000066	0.000079
H	4.253152	0.000585	-2.147167
H	4.253070	-0.000462	2.147319
H	5.501376	0.000081	0.000102
C	-2.717574	-0.000045	-0.000041
H	-3.804503	-0.000064	-0.000056

S2 (B3LYP/6-311G(d))**:**

Atom	x	y	z
C	-4.009064	6.468831	-0.758359
C	-4.389652	5.182022	-0.387483
C	-5.348281	4.973023	0.608609
C	-5.913575	6.097014	1.219667
C	-5.529395	7.384759	0.854416
C	-4.563914	7.590145	-0.136603
C	-3.821284	9.115074	-2.013202
C	-2.524205	9.246341	-2.518703
C	-1.658870	9.658501	-0.171915
C	-2.950393	9.493895	0.337996
C	-0.641145	10.133163	0.669385
C	-0.893594	10.438745	1.997396
C	-2.181985	10.272986	2.509221
C	-3.194679	9.808308	1.684414
C	-4.906487	9.068350	-2.901466
C	-4.715200	9.149631	-4.271580
C	-3.421380	9.283096	-4.778818
C	-2.343489	9.332829	-3.908543
C	0.142051	5.834288	-0.885617
C	-0.609807	7.002115	-0.785240
C	-0.440529	8.047490	-1.696317
C	0.524083	7.896982	-2.698599
C	1.277117	6.730467	-2.795587
C	1.097316	5.675231	-1.894091
C	1.911144	4.424115	-1.987355
C	3.255683	4.424373	-1.561494
C	4.040554	3.214034	-1.647392
C	3.452199	2.024475	-2.123658
C	2.105080	2.021161	-2.539846
C	1.330485	3.240114	-2.487326
C	1.462651	0.830647	-3.009972
C	0.162960	0.838576	-3.431064
C	-0.586084	2.045345	-3.416199
C	-0.021231	3.202339	-2.958847
C	3.878816	5.597188	-1.025293
C	5.188072	5.595505	-0.634926
C	5.970597	4.416500	-0.756770
C	5.413462	3.267774	-1.243217
C	5.266189	-1.148247	-1.071985
C	4.515693	0.024619	-1.022861
C	4.257141	0.765282	-2.179674
C	4.768426	0.285141	-3.390675
C	5.521610	-0.883539	-3.440495
C	5.793163	-1.615070	-2.278357
C	8.153687	-2.545438	-2.327221
C	8.968366	-2.784169	-1.215995
C	7.095580	-4.099328	-0.121940
C	6.288371	-3.874871	-1.241824
C	6.664982	-5.010706	0.855330
C	5.460310	-5.684982	0.729051
C	4.657317	-5.464665	-0.391611
C	5.074088	-4.567632	-1.362875
C	8.727969	-1.986491	-3.480359
C	10.073672	-1.657271	-3.527757
C	10.882255	-1.886530	-2.413089
C	10.329513	-2.447862	-1.273186
C	8.659574	-1.874487	3.581177
C	8.735572	-2.784190	2.529594
C	8.409928	-2.398871	1.225254
C	8.029107	-1.072916	1.004519
C	7.948175	-0.166020	2.058145

C	-1.248438	-4.977960	-3.955160
C	-2.347280	-5.185671	-3.039514
C	-2.203468	-6.076137	-1.955388
C	-0.989537	-6.770516	-1.771543
C	0.098433	-6.583486	-2.704104
C	-0.788970	-7.663142	-0.669491
C	0.381894	-8.348673	-0.508916
C	1.439136	-8.190167	-1.444602
C	1.300950	-7.334930	-2.501862
C	-1.424969	-4.043128	-5.025605
C	-2.604279	-3.375713	-5.202154
C	-3.694847	-3.602763	-4.320478
C	-3.568435	-4.476591	-3.277833
C	-4.702764	-5.555793	0.866478
C	-3.654078	-5.344077	-0.025239
C	-3.337834	-6.291414	-1.004646
C	-4.105206	-7.460096	-1.054809
C	-5.155214	-7.672324	-0.165875
C	-5.477372	-6.717297	0.803734
C	-6.353987	-6.399548	3.154566
C	-7.012693	-5.299052	3.709415
C	-8.643615	-5.302263	1.759161
C	-7.961719	-6.378664	1.185247
C	-9.853081	-4.870588	1.191136
C	-10.380134	-5.490093	0.070296
C	-9.699265	-6.565072	-0.506203
C	-8.507678	-7.000830	0.049975
C	-5.357102	-7.051558	3.898226
C	-5.011928	-6.619280	5.167981
C	-5.665487	-5.515507	5.721595
C	-6.654321	-4.869222	4.997945
C	-6.632945	-1.538388	1.166023
C	-7.024054	-2.838202	1.473095
C	-7.737467	-3.116878	2.642163
C	-8.063876	-2.052130	3.486870
C	-7.675138	-0.750944	3.175220
C	-6.951413	-0.470954	2.012526
C	-6.548141	0.928329	1.668572
C	-5.381494	1.485820	2.228986
C	-4.990895	2.835095	1.889343
C	-5.768371	3.587503	0.986238
C	-6.935248	3.029747	0.425454
C	-7.334118	1.686073	0.776871
C	-7.748301	3.756620	-0.502614
C	-8.882926	3.212694	-1.035006
C	-9.285208	1.898744	-0.674974
C	-8.535257	1.163072	0.198532
C	-4.551139	0.747246	3.131958
C	-3.425803	1.297437	3.677343
C	-3.052069	2.630424	3.358317
C	-3.808565	3.370405	2.494190
H	-3.283713	6.590211	-1.553204
H	-3.944722	4.327818	-0.887727
H	-6.670290	5.962006	1.986197
H	-6.003433	8.237426	1.322472
H	0.350420	10.267120	0.253903
H	-0.092956	10.803990	2.632523
H	-2.394801	10.508621	3.547101
H	-4.199088	9.697749	2.073243
H	-5.905167	8.980148	-2.490747
H	-5.567277	9.111123	-4.942635
H	-3.255686	9.351070	-5.849297
H	-1.341783	9.457445	-4.299395
H	-0.007248	5.039615	-0.161784
H	-1.321544	7.095672	0.025496
H	0.707017	8.708686	-3.390284
H	2.021727	6.638935	-3.580089
H	2.027294	-0.093086	-3.026418
H	-0.300433	-0.079150	-3.778832
H	-1.612911	2.044949	-3.768053

H	-0.599183	4.117954	-2.951563	H	-10.100038	-7.061188	-1.384392
H	3.289924	6.500621	-0.929055	H	-7.988406	-7.845624	-0.384433
H	5.634816	6.498394	-0.230733	H	-4.867988	-7.913817	3.460747
H	7.014257	4.429966	-0.458825	H	-4.237989	-7.136029	5.726387
H	6.016283	2.372782	-1.332834	H	-5.404489	-5.164849	6.715029
H	5.425285	-1.704460	-0.156618	H	-7.176203	-4.024591	5.430557
H	4.116858	0.363130	-0.072049	H	-6.083061	-1.346938	0.250103
H	4.570288	0.833133	-4.306428	H	-6.785754	-3.634626	0.778261
H	5.882445	-1.240495	-4.396010	H	-8.646691	-2.239471	4.379027
H	7.299415	-5.194806	1.713295	H	-7.947266	0.062073	3.841079
H	5.147577	-6.383085	1.499064	H	-7.447848	4.758729	-0.782157
H	3.712855	-5.986625	-0.505729	H	-9.481734	3.785482	-1.736169
H	4.466485	-4.399168	-2.243232	H	-10.192312	1.478922	-1.098321
H	8.106005	-1.829463	-4.351935	H	-8.847589	0.161571	0.467102
H	10.493160	-1.225714	-4.430966	H	-4.830101	-0.269393	3.380058
H	11.937154	-1.632750	-2.437656	H	-2.812369	0.715469	4.357930
H	10.947848	-2.652995	-0.407464	H	-2.159824	3.059746	3.802953
H	8.921025	-2.194332	4.585052	H	-3.518080	4.386056	2.255653
H	9.073096	-3.794680	2.718332	C	-8.149488	-4.556616	2.996920
H	7.802370	-0.731366	0.002009	C	-6.659534	-6.948958	1.762963
H	7.646321	0.857667	1.860796	C	8.457846	-3.420791	0.075099
H	-1.589110	-7.789453	0.048868	C	6.656812	-2.889632	-2.348958
H	0.506315	-9.019329	0.335455	C	-1.284637	9.334055	-1.616637
H	2.359120	-8.752470	-1.318467	C	-4.130124	9.017098	-0.520594
H	2.111027	-7.218642	-3.210978	O	-5.273199	9.843455	-0.222382
H	-0.599415	-3.870253	-5.704813	H	-5.023212	10.758409	-0.402294
H	-2.712700	-2.673081	-6.022366	O	-0.421341	10.370362	-2.125814
H	-4.632097	-3.078553	-4.478161	H	-0.932093	11.189582	-2.143113
H	-4.405685	-4.645894	-2.612455	O	6.337847	-3.473183	-3.630993
H	-4.896588	-4.813128	1.631022	H	6.868008	-4.274811	-3.725770
H	-3.063423	-4.436720	0.050546	O	9.404113	-4.420134	0.505420
H	-3.872181	-8.217709	-1.796435	H	9.421932	-5.110440	-0.169082
H	-5.712812	-8.598583	-0.207717	O	-6.791321	-8.383644	1.846517
H	-10.377089	-4.045406	1.658709	H	-7.560465	-8.571670	2.398490
H	-11.315853	-5.141095	-0.354512	O	-9.246714	-4.415526	3.922640

H	-9.502843	-5.302170	4.204728
C	8.257909	-0.552443	3.365833
C	-0.042218	-5.685823	-3.781824
C	8.172466	0.421847	4.498450
C	6.980324	0.525079	5.247205
C	9.283028	1.234087	4.813613
C	5.824545	-0.272163	4.968050
C	6.901565	1.466904	6.338735
C	9.193793	2.173358	5.906608
C	10.516127	1.167041	4.089443
C	4.682268	-0.149834	5.709453
H	5.865174	-0.982885	4.151628
C	5.688005	1.560868	7.087131
C	8.008073	2.263492	6.637379
C	10.325987	2.989128	6.213860
H	10.601629	0.466282	3.267854
C	11.575958	1.966234	4.417491
C	4.610069	0.778603	6.785150
H	3.818900	-0.765114	5.476795
H	5.640526	2.273000	7.905978
H	7.944683	2.971874	7.459346
C	11.482497	2.891844	5.494018
H	10.246749	3.692589	7.037741
H	12.500457	1.896104	3.853050
H	3.694363	0.861609	7.361854
H	12.334530	3.517956	5.739083
C	1.090287	-5.489155	-4.740916
C	1.112060	-6.162613	-5.968103
C	2.156669	-4.638393	-4.425439
C	2.173568	-5.991443	-6.854709
H	0.292251	-6.826253	-6.224473
C	3.222538	-4.467994	-5.307941
H	2.147722	-4.105337	-3.479784
C	3.231297	-5.145928	-6.525925
H	2.174310	-6.522324	-7.801722
H	4.047380	-3.817968	-5.035294
H	4.059346	-5.015547	-7.215652

S3 (B3LYP/6-311G(d))**:**

Atom	x	y	z
C	0.038233	1.199927	12.398753
C	0.038766	1.199922	11.005799
C	-0.000000	0.000000	10.286905
C	-0.038766	-1.199922	11.005799
C	-0.038233	-1.199927	12.398753
C	-0.000000	0.000000	13.117646
C	-0.000000	0.000000	14.614649
C	-1.208584	0.172699	15.318976
C	-1.208087	0.174851	16.764157
C	-0.000000	0.000000	17.468296
C	1.208087	-0.174851	16.764157
C	1.208584	-0.172699	15.318976
C	2.456481	-0.355329	17.441678
C	3.624981	-0.518100	16.752832
C	3.626568	-0.509262	15.332291
C	2.459229	-0.341704	14.642528
C	-2.459229	0.341704	14.642528
C	-3.626568	0.509262	15.332291
C	-3.624981	0.518100	16.752832
C	-2.456481	0.355329	17.441678
C	0.176599	1.187489	21.077215
C	0.176719	1.187531	19.684166
C	-0.000000	0.000000	18.965580
C	-0.176719	-1.187531	19.684166
C	-0.176599	-1.187489	21.077215
C	-0.000000	0.000000	21.795930
C	-0.000000	0.000000	23.293209
C	1.205473	-0.192852	23.997245
C	1.205362	-0.192870	25.442401
C	-1.205362	0.192870	25.442401

C	-1.205473	0.192852	23.997245	C	-1.207165	-0.181390	-10.715986
C	-2.450888	0.391327	26.120352	C	-1.207146	-0.181517	-9.270824
C	-3.615790	0.577175	25.431124	C	0.000000	-0.000000	-8.566733
C	-3.616093	0.577037	24.010453	C	1.207146	0.181517	-9.270824
C	-2.451602	0.391153	23.320530	C	1.207165	0.181390	-10.715986
C	2.451602	-0.391153	23.320530	C	2.454810	0.368478	-8.593741
C	3.616093	-0.577037	24.010453	C	3.621334	0.543049	-9.283095
C	3.615790	-0.577175	25.431124	C	3.621442	0.542341	-10.703711
C	2.450888	-0.391327	26.120352	C	2.454976	0.367385	-11.393065
C	0.029702	1.200165	-22.314483	C	-2.454976	-0.367385	-11.393065
C	0.028955	1.200208	-23.707373	C	-3.621442	-0.542341	-10.703711
C	-0.028955	-1.200208	-23.707373	C	-3.621334	-0.543049	-9.283095
C	-0.029702	-1.200165	-22.314483	C	-2.454810	-0.368478	-8.593741
C	0.000000	-0.000000	-21.595528	C	-0.172588	1.188145	-4.957818
C	0.000000	-0.000000	-20.098636	C	-0.172597	1.188143	-6.350865
C	1.214294	0.126698	-19.394170	C	0.000000	-0.000000	-7.069449
C	1.213768	0.129867	-17.948987	C	0.172597	-1.188143	-6.350865
C	0.000000	-0.000000	-17.244900	C	0.172588	-1.188145	-4.957818
C	-1.213768	-0.129867	-17.948987	C	0.000000	-0.000000	-4.239235
C	-1.214294	-0.126698	-19.394170	C	0.000000	-0.000000	-2.741953
C	-2.467785	-0.265873	-17.271445	C	1.206535	0.185541	-2.037861
C	-3.641696	-0.383766	-17.960223	C	1.206573	0.185327	-0.592698
C	-3.643374	-0.371219	-19.380720	C	-0.000000	0.000000	0.111409
C	-2.470696	-0.246467	-20.070503	C	-1.206573	-0.185327	-0.592698
C	2.470696	0.246467	-20.070503	C	-1.206535	-0.185541	-2.037861
C	3.643374	0.371219	-19.380720	C	-2.453900	-0.374612	0.084360
C	3.641696	0.383766	-17.960223	C	-3.619816	-0.553171	-0.605006
C	2.467785	0.265873	-17.271445	C	-3.619638	-0.554259	-2.025618
C	0.158172	-1.190148	-13.635957	C	-2.453617	-0.376290	-2.714956
C	0.158372	-1.190115	-15.029001	C	2.453617	0.376290	-2.714956
C	0.000000	-0.000000	-15.747614	C	3.619638	0.554259	-2.025618
C	-0.158372	1.190115	-15.029001	C	3.619816	0.553171	-0.605006
C	-0.158172	1.190148	-13.635957	C	2.453900	0.374612	0.084360
C	0.000000	-0.000000	-12.917368	C	-0.151825	1.190967	3.720314
C	0.000000	-0.000000	-11.420091	C	-0.151575	1.191003	2.327275

C	-0.000000	0.000000	1.608672
C	0.151575	-1.191003	2.327275
C	0.151825	-1.190967	3.720314
C	-0.000000	0.000000	4.438937
C	-0.000000	0.000000	5.936208
C	1.215918	0.107967	6.640321
C	1.216364	0.104654	8.085505
C	-0.000000	0.000000	8.789922
C	-1.216364	-0.104654	8.085505
C	-1.215918	-0.107967	6.640321
C	-2.474596	-0.202508	8.761927
C	-3.649318	-0.306752	8.072229
C	-3.647889	-0.319724	6.651731
C	-2.472121	-0.222571	5.962873
C	2.472121	0.222571	5.962873
C	3.647889	0.319724	6.651731
C	3.649318	0.306752	8.072229
C	2.474596	0.202508	8.761927
H	0.068063	2.142045	12.936964
H	0.069249	2.142028	10.467598
H	-0.069249	-2.142028	10.467598
H	-0.068063	-2.142045	12.936964
H	2.462940	-0.360436	18.524605
H	4.558059	-0.653022	17.290655
H	4.561219	-0.635140	14.794994
H	2.468895	-0.334335	13.559663
H	-2.468895	0.334335	13.559663
H	-4.561219	0.635140	14.794994
H	-4.558059	0.653022	17.290655
H	-2.462940	0.360436	18.524605
H	0.315294	2.119726	21.615599
H	0.315560	2.119798	19.145838
H	-0.315560	-2.119798	19.145838
H	-0.315294	-2.119726	21.615599
H	-2.458006	0.392024	27.203322

H	-4.546850	0.725478	25.968926
H	-4.547535	0.725225	23.473200
H	-2.459747	0.391753	22.237561
H	2.459747	-0.391753	22.237561
H	4.547535	-0.725225	23.473200
H	4.546850	-0.725478	25.968926
H	2.458006	-0.392024	27.203322
H	0.053584	2.142489	-21.776326
H	0.052379	2.142533	-24.245518
H	-0.052379	-2.142533	-24.245518
H	-0.053584	-2.142489	-21.776326
H	-2.474159	-0.273541	-16.188532
H	-4.578957	-0.485591	-17.422382
H	-4.582252	-0.460247	-19.918004
H	-2.480460	-0.236281	-21.153318
H	2.480460	0.236281	-21.153318
H	4.582252	0.460247	-19.918004
H	4.578957	0.485591	-17.422382
H	2.474159	0.273541	-16.188532
H	0.282248	-2.124483	-13.097635
H	0.282703	-2.124409	-15.567330
H	-0.282703	2.124409	-15.567330
H	-0.282248	2.124483	-13.097635
H	2.462359	0.369536	-7.510797
H	4.553902	0.682753	-8.745605
H	4.554116	0.681321	-11.241202
H	2.462663	0.367503	-12.476006
H	-2.462663	-0.367503	-12.476006
H	-4.554116	-0.681321	-11.241202
H	-4.553902	-0.682753	-8.745605
H	-2.462359	-0.369536	-7.510797
H	-0.308179	2.120872	-4.419486
H	-0.308193	2.120869	-6.889196
H	0.308193	-2.120869	-6.889196
H	0.308179	-2.120872	-4.419486

H	-2.461699	-0.374259	1.167300
H	-4.552127	-0.694569	-0.067516
H	-4.551771	-0.696777	-2.563122
H	-2.461146	-0.377399	-3.797900
H	2.461146	0.377399	-3.797900
H	4.551771	0.696777	-2.563122
H	4.552127	0.694569	-0.067516
H	2.461699	0.374259	1.167300
H	-0.271001	2.125939	4.258632
H	-0.270431	2.126019	1.788959
H	0.270431	-2.126019	1.788959
H	0.271001	-2.125939	4.258632
H	-2.484007	-0.192190	9.844750
H	-4.589591	-0.379373	8.609541
H	-4.586777	-0.405596	6.113948
H	-2.478655	-0.230867	4.879969
H	2.478655	0.230867	4.879969
H	4.586777	0.405596	6.113948
H	4.589591	0.379373	8.609541
H	2.484007	0.192190	9.844750
C	0.000000	-0.000000	-24.426575
C	-0.000000	0.000000	26.146348
C	-0.000000	0.000000	27.643755
C	-0.181045	-1.189985	28.358410
C	0.181045	1.189985	28.358410
C	-0.181215	-1.190702	29.751926
H	-0.322441	-2.119012	27.815236
C	0.181215	1.190702	29.751926
H	0.322441	2.119012	27.815236
C	-0.000000	0.000000	30.452631
H	-0.323156	-2.122815	30.289880
H	0.323156	2.122815	30.289880
H	-0.000000	0.000000	31.537975
C	0.000000	-0.000000	-25.923026
C	1.201915	-0.234242	-26.626356
C	-1.201915	0.234242	-26.626356
C	2.451060	-0.463218	-25.965458
C	1.195565	-0.235701	-28.070268
C	-1.195565	0.235701	-28.070268
C	-2.451060	0.463218	-25.965458
C	3.599092	-0.686671	-26.673727
H	2.478659	-0.456203	-24.882721
C	2.418290	-0.475171	-28.769757
C	0.000000	-0.000000	-28.750534
C	-2.418290	0.475171	-28.769757
H	-2.478659	0.456203	-24.882721
C	-3.599092	0.686671	-26.673727
C	3.585551	-0.695955	-28.096271
H	4.533362	-0.857063	-26.148145
H	2.399479	-0.476524	-29.855765
H	0.000000	-0.000000	-29.837415
C	-3.585551	0.695955	-28.096271
H	-2.399479	0.476524	-29.855765
H	-4.533362	0.857063	-26.148145
H	4.506854	-0.876170	-28.640842
H	-4.506854	0.876170	-28.640842

6 References

[S1] K. Liu, R. A. Lalancette, F. Jakle, *J. Am. Chem. Soc.* **2017**, *139*, 18170–18173.

[S2] K. Iida, K. Endo, Y. Li, K. Okabe, M. Yabe (Mitsubishi Chemical Corp), EP2272894 B1, **2009**.

[S3] G. R. Fulmer, A. J. M. Miller, N. H. Sherden, H. E. Gottlieb, A. Nudelman, B. M. Stoltz, J. E. Bercaw, K. I. Goldberg, *Organometallics* **2010**, *29*, 2176–2179.

[S4] L. Krause, R. Herbst-Irmer, G. M. Sheldrick, D. Stalke, *J. Appl. Crystallogr.* **2015**, *48*, 3–10.

[S5] G. M. Sheldrick, *Acta Crystallogr., Sect. A:Found. Adv.* **2015**, *71*, 3–8.

[S6] G. M. Sheldrick, *Acta Crystallogr., Sect. C:Struct. Chem.* **2015**, *71*, 3–8.

[S7] M. J. Frisch, G. W. Trucks, H. B. Schlegel, G. E. Scuseria, M. A. Robb, J. R. Cheeseman, G. Scalmani, V. Barone, G. A. Petersson, H. Nakatsuji, X. Li, M. Caricato, A. V. Marenich, J. Bloino, B. G. Janesko, R. Gomperts, B. Mennucci, H. P. Hratchian, J. V. Ortiz, A. F. Izmaylov, J. L. Sonnenberg, Williams, F. Ding, F. Lipparini, F. Egidi, J. Goings, B. Peng, A. Petrone, T. Henderson, D. Ranasinghe, V. G. Zakrzewski, J. Gao, N. Rega, G. Zheng, W. Liang, M. Hada, M. Ehara, K. Toyota, R. Fukuda, J. Hasegawa, M. Ishida, T. Nakajima, Y. Honda, O. Kitao, H. Nakai, T. Vreven, K. Throssell, J. A. Montgomery Jr., J. E. Peralta, F. Ogliaro, M. J. Bearpark, J. J. Heyd, E. N. Brothers, K. N. Kudin, V. N. Staroverov, T. A. Keith, R. Kobayashi, J. Normand, K. Raghavachari, A. P. Rendell, J. C. Burant, S. S. Iyengar, J. Tomasi, M. Cossi, J. M. Millam, M. Klene, C. Adamo, R. Cammi, J. W. Ochterski, R. L. Martin, K. Morokuma, O. Farkas, J. B. Foresman, D. J. Fox, Wallingford, CT, **2016**.

[S8] P. Hohenberg, W. Kohn, *Phys. Rev.* **1964**, *136*, B864–B871.

[S9] W. Kohn, L. J. Sham, *Phys. Rev.* **1965**, *140*, A1133–A1138.

[S10] R. G. Parr, W. Yang, *Density-functional theory of atoms and molecules*, 1st ed., Oxford Univ. Press, New York, NY, **1994**.

[S11] W. Koch, *A Chemist's Guide to Density Functional Theory*, 2nd ed., Wiley-VCH, Weinheim, **2001**.

[S12] S. H. Vosko, L. Wilk, M. Nusair, *Can. J. Phys.* **1980**, *58*, 1200–1211.

[S13] C. Lee, W. Yang, R. G. Parr, *Phys. Rev. B: Condens. Matter Mater. Phys.* **1988**, *37*, 785–789.

[S14] A. D. Becke, *J. Chem. Phys.* **1993**, *98*, 5648–5652.

[S15] P. J. Stephens, F. J. Devlin, C. F. Chabalowski, M. J. Frisch, *J. Phys. Chem.* **1994**, *98*, 11623–11627.

[S16] S. Grimme, S. Ehrlich, L. Goerigk, *J. Comput. Chem.* **2011**, *32*, 1456–1465.

[S17] R. Krishnan, J. S. Binkley, R. Seeger, J. A. Pople, *J. Chem. Phys.* **1980**, *72*, 650–654.

[S18] A. D. McLean, G. S. Chandler, *J. Chem. Phys.* **1980**, *72*, 5639–5648.

[S19] M. M. Francl, W. J. Pietro, W. J. Hehre, J. S. Binkley, M. S. Gordon, D. J. DeFrees, J. A. Pople, *J. Chem. Phys.* **1982**, *77*, 3654–3665.

[S20] J. J. Stewart, *J. Mol. Model.* **2007**, *13*, 1173–1213.

[S21] R. Bauernschmitt, R. Ahlrichs, *Chem. Phys. Lett.* **1996**, *256*, 454–464.

[S22] M. E. Casida, C. Jamorski, K. C. Casida, D. R. Salahub, *J. Chem. Phys.* **1998**, *108*, 4439–4449.

[S23] R. E. Stratmann, G. E. Scuseria, M. J. Frisch, *J. Chem. Phys.* **1998**, *109*, 8218–8224.

[S24] C. van Caillie, R. D. Amos, *Chem. Phys. Lett.* **1999**, *308*, 249–255.

[S25] F. Furche, R. Ahlrichs, *J. Chem. Phys.* **2002**, *117*, 7433–7447.

[S26] G. Scalmani, M. J. Frisch, B. Mennucci, J. Tomasi, R. Cammi, V. Barone, *J. Chem. Phys.* **2006**, *124*, 94107.

[S27] T. Helgaker, P. Jørgensen, *J. Chem. Phys.* **1991**, *95*, 2595–2601.

[S28] K. L. Bak, P. Jørgensen, T. Helgaker, K. Ruud, H. J. r. A. Jensen, *J. Chem. Phys.* **1993**, *98*, 8873–8887.

[S29] K. L. Bak, A. E. Hansen, K. Ruud, T. Helgaker, J. Olsen, P. Jørgensen, *Theor. Chim. Acta* **1995**, *90*, 441–458.

[S30] J. Olsen, K. L. Bak, K. Ruud, T. Helgaker, P. Jørgensen, *Theor. Chim. Acta* **1995**, *90*, 421–439.
[S31] A. E. Hansen, K. L. Bak, *ENANTIOMER* **1999**, 455–476.
[S32] J. Autschbach, T. Ziegler, S. J. A. van Gisbergen, E. J. Baerends, *J. Chem. Phys.* **2002**, *116*, 6930–6940.
[S33] T. Yanai, D. P. Tew, N. C. Handy, *Chem. Phys. Lett.* **2004**, *393*, 51–57.
[S34] C. Adamo, V. Barone, *J. Chem. Phys.* **1999**, *110*, 6158–6170.
[S35] J. D. Chai, M. Head-Gordon, *Phys. Chem. Chem. Phys.* **2008**, *10*, 6615–6620.
[S36] S. Miertuš, E. Scrocco, J. Tomasi, *Chem. Phys.* **1981**, *55*, 117–129.
[S37] S. Miertuš, J. Tomasi, *Chem. Phys.* **1982**, *65*, 239–245.
[S38] J. L. Pascual-ahuir, E. Silla, I. Tuñon, *J. Comput. Chem.* **1994**, *15*, 1127–1138.
[S39] M. Cossi, V. Barone, R. Cammi, J. Tomasi, *Chem. Phys. Lett.* **1996**, *255*, 327–335.
[S40] V. Barone, M. Cossi, J. Tomasi, *J. Chem. Phys.* **1997**, *107*, 3210–3221.
[S41] E. Cancès, B. Mennucci, J. Tomasi, *J. Chem. Phys.* **1997**, *107*, 3032–3041.
[S42] B. Mennucci, E. Cancès, J. Tomasi, *J. Phys. Chem. B* **1997**, *101*, 10506–10517.
[S43] B. Mennucci, J. Tomasi, *J. Chem. Phys.* **1997**, *106*, 5151–5158.
[S44] V. Barone, M. Cossi, *J. Phys. Chem. A* **1998**, *102*, 1995–2001.
[S45] V. Barone, M. Cossi, J. Tomasi, *J. Comput. Chem.* **1998**, *19*, 404–417.
[S46] M. Cossi, V. Barone, B. Mennucci, J. Tomasi, *Chem. Phys. Lett.* **1998**, *286*, 253–260.
[S47] R. Cammi, B. Mennucci, J. Tomasi, *J. Phys. Chem. A* **1999**, *103*, 9100–9108.
[S48] M. Cossi, V. Barone, M. A. Robb, *J. Chem. Phys.* **1999**, *111*, 5295–5302.
[S49] J. Tomasi, B. Mennucci, E. Cancès, *J. Mol. Struct.:THEOCHEM* **1999**, *464*, 211–226.
[S50] R. Cammi, B. Mennucci, J. Tomasi, *J. Phys. Chem. A* **2000**, *104*, 5631–5637.
[S51] M. Cossi, V. Barone, *J. Chem. Phys.* **2000**, *112*, 2427–2435.
[S52] M. Cossi, V. Barone, *J. Chem. Phys.* **2001**, *115*, 4708–4717.
[S53] M. Cossi, N. Rega, G. Scalmani, V. Barone, *J. Chem. Phys.* **2001**, *114*, 5691–5701.
[S54] M. Cossi, G. Scalmani, N. Rega, V. Barone, *J. Chem. Phys.* **2002**, *117*, 43–54.
[S55] M. Cossi, N. Rega, G. Scalmani, V. Barone, *J. Comput. Chem.* **2003**, *24*, 669–681.
[S56] R. Cammi, *J. Chem. Phys.* **2009**, *131*, 164104.
[S57] R. Cammi, *Int. J. Quantum Chem.* **2010**, *110*, 3040–3052.
[S58] F. Lipparini, G. Scalmani, B. Mennucci, E. Cancès, M. Caricato, M. J. Frisch, *J. Chem. Phys.* **2010**, *133*, 014106.
[S59] G. Scalmani, M. J. Frisch, *J. Chem. Phys.* **2010**, *132*, 114110.
[S60] M. Caricato, *J. Chem. Theory Comput.* **2012**, *8*, 4494–4502.
[S61] N. M. O'Boyle, A. L. Tenderholt, K. M. Langner, *J. Comput. Chem.* **2008**, *29*, 839–845.
[S62] M. J. Turner, J. J. McKinnon, D. Jayatilaka, M. A. Spackman, *CrystEngComm* **2011**, *13*, 1804–1813.
[S63] P. R. Spackman, M. J. Turner, J. J. McKinnon, S. K. Wolff, D. J. Grimwood, D. Jayatilaka, M. A. Spackman, *J. Appl. Crystallogr.* **2021**, *54*, 1006–1011.
[S64] H. Takaba, H. Omachi, Y. Yamamoto, J. Bouffard, K. Itami, *Angew Chem Int Ed Engl* **2009**, *48*, 6112–6116.
[S65] Y. Segawa, H. Omachi, K. Itami, *Org. Lett.* **2010**, *12*, 2262–2265.
